# Sequence, genome organization, annotation and proteomics of the thermophilic, 47.7-kb *Geobacillus stearothermophilus* bacteriophage TP-84 and its classification in the new *Tp84virus* genus

**DOI:** 10.1371/journal.pone.0195449

**Published:** 2018-04-06

**Authors:** Piotr M. Skowron, Andrew M. Kropinski, Joanna Zebrowska, Lukasz Janus, Kasjan Szemiako, Edyta Czajkowska, Natalia Maciejewska, Malgorzata Skowron, Joanna Łoś, Marcin Łoś, Agnieszka Zylicz-Stachula

**Affiliations:** 1 Department of Molecular Biotechnology, Faculty of Chemistry, University of Gdansk, Gdansk, Poland; 2 BioVentures Institute Ltd., Poznan, Poland; 3 Departments of Food Science, Molecular and Cellular Biology; and Pathobiology University of Guelph, Guelph, ON, Canada; 4 Department of Molecular Biotechnology and Microbiology, Faculty of Chemistry, Gdansk University of Technology, Gdansk, Poland; 5 Phage Consultants, Gdansk, Poland; 6 Department of Bacterial Molecular Genetics, Faculty of Biology, University of Gdansk, Gdansk, Poland; Centro Nacional de Biotecnologia, SPAIN

## Abstract

Bacteriophage TP-84 is a well-characterized bacteriophage of historical interest. It is a member of the *Siphoviridae*, and infects a number of thermophilic *Geobacillus* (*Bacillus*) *stearothermophilus* strains. Its’ 47.7-kbp double-stranded DNA genome revealed the presence of 81 coding sequences (CDSs) coding for polypeptides of 4 kDa or larger. Interestingly, all CDSs are oriented in the same direction, pointing to a dominant transcription direction of one DNA strand. Based on a homology search, a hypothetical function could be assigned to 31 CDSs. No RNA or DNA polymerase-coding genes were found on the bacteriophage genome indicating that TP-84 relies on the host’s transcriptional and replication enzymes. The TP84 genome is tightly packed with CDSs, typically spaced by several-to-tens of bp or often overlapping. The genome contains five putative promoter-like sequences showing similarity to the host promoter consensus sequence and allowing for a 2-bp mismatch. In addition, ten putative rho-independent terminators were detected. Because the genome sequence shows essentially no similarity to any previously characterised bacteriophage, TP-84 should be considered a new species in an undefined genus within the *Siphoviridae* family. Thus a taxonomic proposal of a new *Tp84virus* genus has been accepted by the International Committee on Taxonomy of Viruses. The bioinformatics genome analysis was verified by confirmation of 33 TP-84 proteins, which included: a) cloning of a selected CDS in *Escherichia coli*, coding for a DNA single-stranded binding protein (SSB; gene TP84_63), b) purification and functional assays of the recombinant TP-84 SSB, which has been shown to improve PCR reactions, c) mass spectrometric (MS) analysis of TP-84 bacteriophage capsid proteins, d) purification of TP-84 endolysin activity, e) MS analysis of the host cells from infection time course.

## Introduction

Thermophilic bacteriophages are rarely studied and no life cycles have been deciphered to the extent approaching model *Escherichia coli* (*E*. *coli*) viruses, exemplified by λ, T4, T7 or M13. Nevertheless, they are interesting objects for thermophilicity determinants and for practical aspects in industrial microbial processes employing high temperatures. Thermophilic bacteriophages have been isolated from a variety of sources wherever environmental temperatures are increased by either natural processes or human activity. These sources include, amongst others, hot springs and their surrounding soils, hydrothermal vents, soils near volcanic activity, compost heaps, greenhouse soil, cooling units of power plants and wastewaters. Especially rich sources of thermophilic *Bacillus* (*Geobacillus* (*G*.) according to the current classification) bacteria infected with bacteriophages are found in active compost heaps and greenhouse soils due to the high humidity, active microbial metabolism and moderately increased temperature [1]. Older scientific literature includes a number of poorly characterized thermophilic “*Bacillus*” bacteriophages such as 24 podo- and siphoviruses of [*Bacillus*] *caldotenax* and *'B*. *caldovelox*' [2], temperate *Bacillus stearothermophilus* bacteriophages φμ-4 [3], and siphovirus Tφ3, which possesses a 125 nm long and 10 nm wide tail [4]. None of these bacteriophages has been sequenced. *Geobacillus kaustophilus* lytic bacteriophage GBK2 is a member of *Siphoviridae* with a 39.1 kb (43% G+C) circularly permuted genome [5]. It exhibits limited DNA sequence similarity to any other bacteriophage available in the NCBI database. The best-studied *Geobacillus* virus is a deep-sea temperate thermophilic siphovirus GVE2 (also known as E2), which has a 130-nm-long head and a 180-nm-long tail [6]. Its genome size is 40.9 kb (44.8 mol%G+C) and it reveals sequence similarity to the genomes of several *Geobacillus* species. A number of GVE2 proteins have been characterized in the laboratory. The *Geobacillus* bacteriophage D6E is a member of the *Myoviridae* family, possessing a 49.3 kb (46.0%GC) genome [7]. Morphologically, its capsid is 60 nm in diameter and the contractile tail’s size is 60 nm x 16 nm. Lastly, the temperate *Geobacillus* bacteriophage GBSV1 has a 34.7 kb genome, a GC-content of 44.4% and encodes 54 proteins [8].

An additional bacteriophage characterized in the past, TP-84, was discovered in 1952 in greenhouse soil using *G*. *stearothermophilus* strain 2184 as a host [9–12]. Early studies determined a number of microbiological and physical properties of this bacteriophage. It has a rather narrow host range, as it is lytic for only four out of 24 tested related thermophilic bacteria: *G*. *stearothermophilus* strains 4, 10, 2184 and, with 20-fold lower plating efficiency, *Geobacillus* strain T-27. Strains of *Bacillus* (*B*.) *subtilis*, *B*. *megaterium*, *B*. *pumilus*, *B*. *licheniformis*, and *B*. *coagulans*, as well as *Paenibacillus macerans*, were resistant to TP-84 infection. The bacteriophage requires nutrient-rich broth and is strongly dependent on the presence of Ca^2+^ ions since their omission causes a decrease in the bacteriophage yield of approx. 10,000-fold. Supplementation with glucose at the time of infection increases the bacteriophage yield by approx. 1,000-fold [13]. The temperature growth range matched that of the hosts growth range of 43–76°C, with a latent period of 22–24 min. Electron microscopic evaluation revealed an elongated head [14] with dimensions of 53 x 30 nm and a long, non-contractile tail (3–5 nm wide by 131 nm in length). TP-84’s double stranded (ds) genome contains 42% GC and is 13.9 μm long with a molecular weight of 22.4–27 MDa [10–12]. Further development of the TP-84 production process resulted in the determination that *G*. *stearothermophilus* strain 10 cultivation under conditions preventing spore formation, such as rich media, a highly aerobic process, pH 6.5, 10 mM MgCl_2_, 0.5% fructose (before infection) and a temperature of 58°C resulted in a bacteriophage titer of 6 x 10^11^ [11]. It was determined that the bacteriophage is sensitive to chelating agents, such as EDTA and phosphate, resulting in the dissociation of heads from tails and ghost structure formation. This points to the essential role of divalent cations in maintaining TP-84 integrity [15]. Based on the published characteristics, the TP-84 bacteriophage can be classified as a member of the order *Caudovirales*, family *Siphoviridae*.

In this manuscript, we describe the complete, annotated TP-84 genome sequence, show that the sequence is unrelated to other characterized bacteriophages and present an in-depth proteomics analysis.

## Results and discussion

### Basic characterization of the TP-84 genome

**Fig 1:**
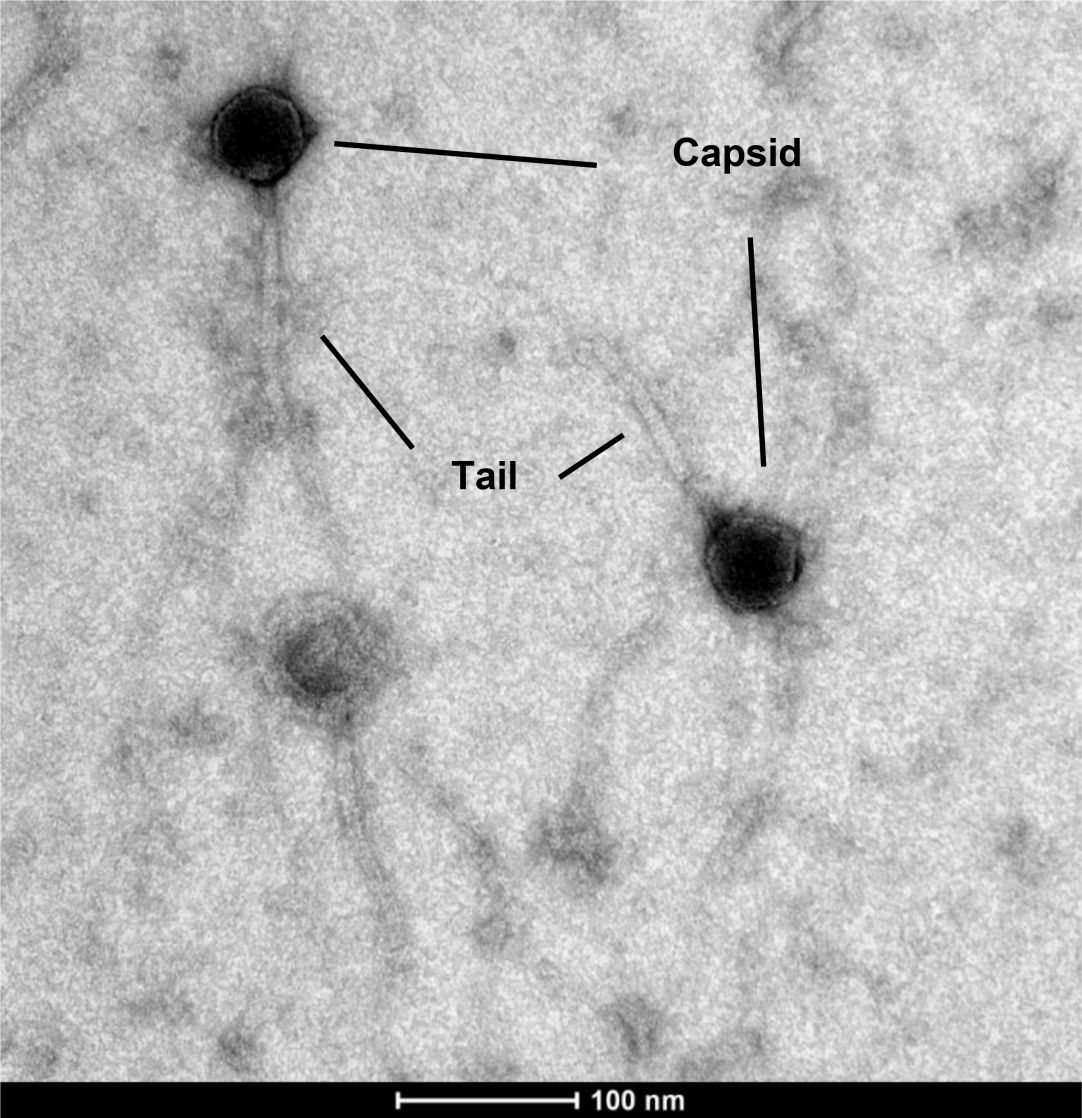
Electron microscopy image of the purified TP-84 bacteriophage. Purified TP-84 sample loaded onto 300 mesh copper grid (Sigma), covered with 2% collodion (Sigma), sprayed with carbon and stained with 2% uranyl acetate (BDH Chemicals). Visualised with a Tecnai G2 Spirit BioTWIN TEM set at 120 kV. Pictures were captured with a Veleta CCD camera.

The TP-84 bacteriophage was cultivated under modified conditions, purified and subjected to transmission electron microscopy (TEM) imaging, confirming the originally published siphoviral morphology (Fig 1) [14]. The isolated and sequenced genome was assembled into a major contig of 47,703 bp giving no evidence of terminal repeats. Its G+C content at 54.5 is close to that of the completely sequenced host strain (CP008934; 52.6 mol%G+C), but radically different from the 42.0%GC calculated from the buoyant density and melting temperature of the bacteriophage DNA [11]. This should indicate the presence of modified bases [16] but no sequence homology evidence was found for any protein that could contribute to this. Saunders and Campbell also measured the mass of the DNA on the basis of three physical parameters [10] giving an average value of 24x 10^6^ Da or 39.7 kb, again significantly less than the genome size derived from sequencing.

**Fig 2:**
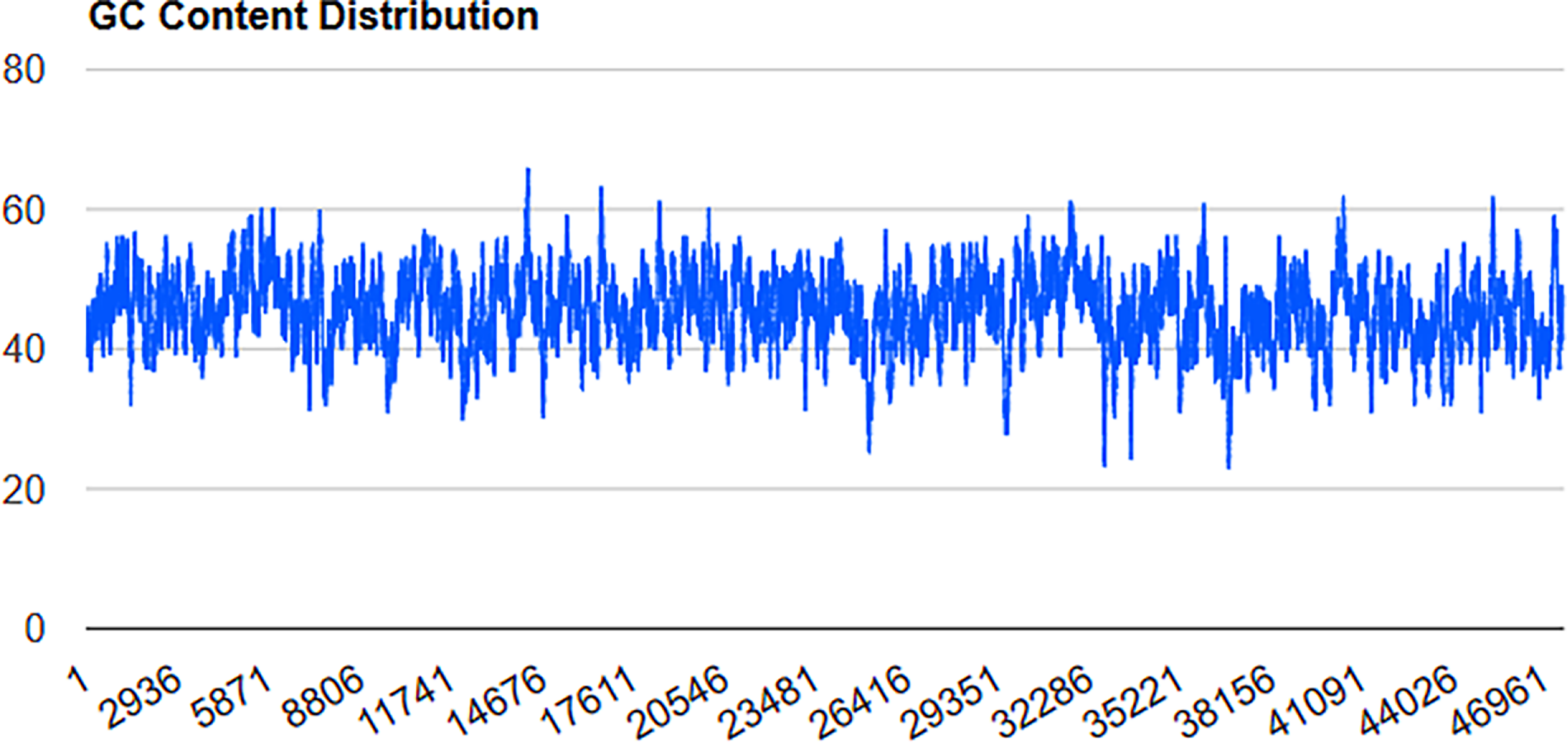
The GC-skew distribution over the genome of TP-84. The bacteriophage genome was analysed using the default settings of GC Content Calculator (Biologics International Corp, Indianapolis, USA; http://www.biologicscorp.com/tools/GCContent/; blue).

Using GC Content Calculator, we calculated the GC skew across the length of the genome (Fig 2), which indicates four regions where the GC-content was ≥62% at 14200, 16561, 40462 and 45274; and five regions where it is ≤ 28% (25196, 29575, 32760, 33623, and 36764). The significance of these regions of extreme base compositional divergence is not known.

Among the common restriction endonucleases there were no sites for BamHI, EcoRV, HpaI, KpnI, PstI, SacI, SalI and SmaI. Since its host, *G*. *stearothermophilus*, produces a number of characterized Type II restriction endonucleases [17], we screened the TP-84 genome to reveal: Gst1588I (CYCGRG, 0 sites), Gst1588II (GATC, 743 sites), GstI/GstGS18 (GGATCC, 0 sites), Gst4109 (CGATCG, 59 sites), and GsaI (CCCAGC, 0 sites). There is no evidence that this bacteriophage encodes a GATC-specific methyltransferase, but it seems likely.

**Fig 3:**
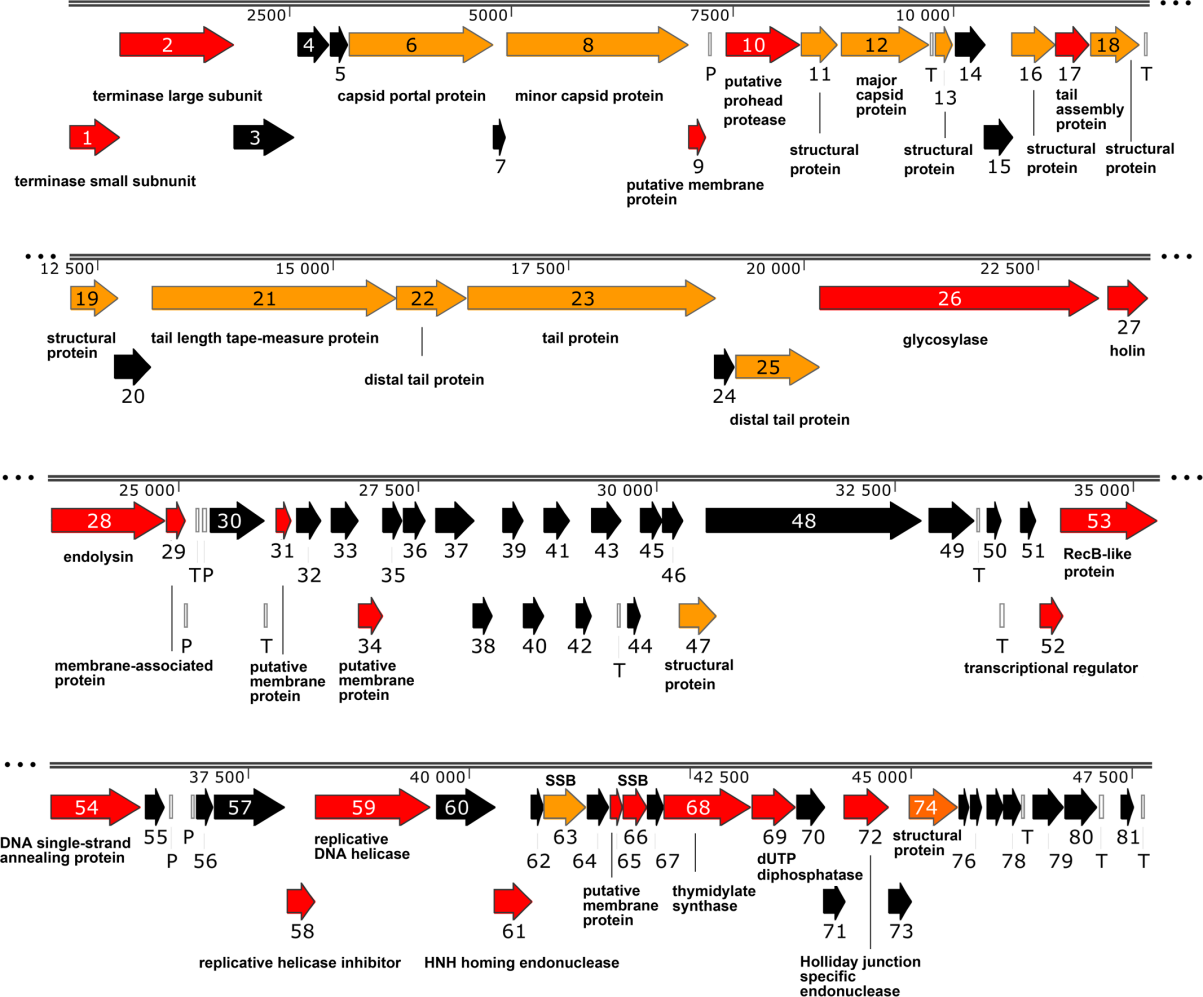
Genome organization of the thermophilic, 47.7-kb bacteriophage TP-84. Putative genes, encoding proteins with assigned biological function, are marked with red arrows. Genes with assigned function confirmed by proteomic analysis are marked with orange arrows. Genes without assigned biological function are marked with black arrows. P—putative host-dependent promoter, T–Rho-independent terminator, SSB–single-stranded DNA-binding protein. The scheme was created using SnapGene software (http://www.snapgene.com) and further modified.

The genome (Fig 3, S1 and S2 Files) also contains five putative promoter-like sequences showing similarity to the host promoter consensus sequence (TTGACA(N15-18)TATAAT) and allowing for a 2-bp mismatch. In addition, ten putative rho-independent terminators were discovered using ARNold. The data on these terminators are presented in S1 Table.

**Fig 4:**
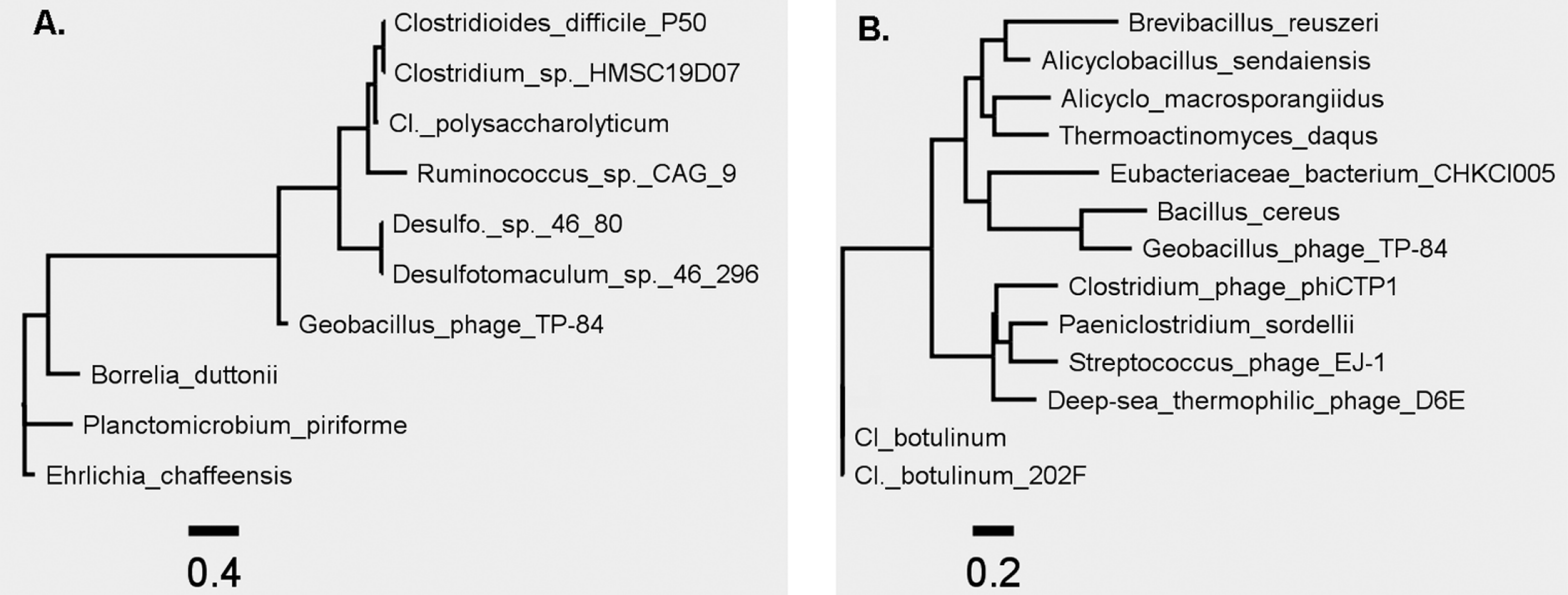
Phylogenetic trees for TP-84 bacteriophage. A) Phylogenetic tree constructed using the large subunit terminase (TerL). B) Phylogenetic tree constructed using thymidylate synthase (Ts) proteins. The trees do not include all *Geobacillus* phage proteins since the differences between all the sequences renders the trees unreliable.

BLASTN analysis against the NCBI viruses and non-redundant databases showed only limited sequence similarity to bacterial (presumably prophage) or viral genomes revealing TP-84 to be a genomic orphan. The relationships are such that TP-84 has been assigned as a new species (*Geobacillus virus TP84*) in a new genus (*Tp84virus*) within the *Siphoviridae* by the International Committee on Taxonomy of Viruses (Fig 4, S3 File) [18].

### Description of the bacteriophage TP-84 genes

**Fig 5:**
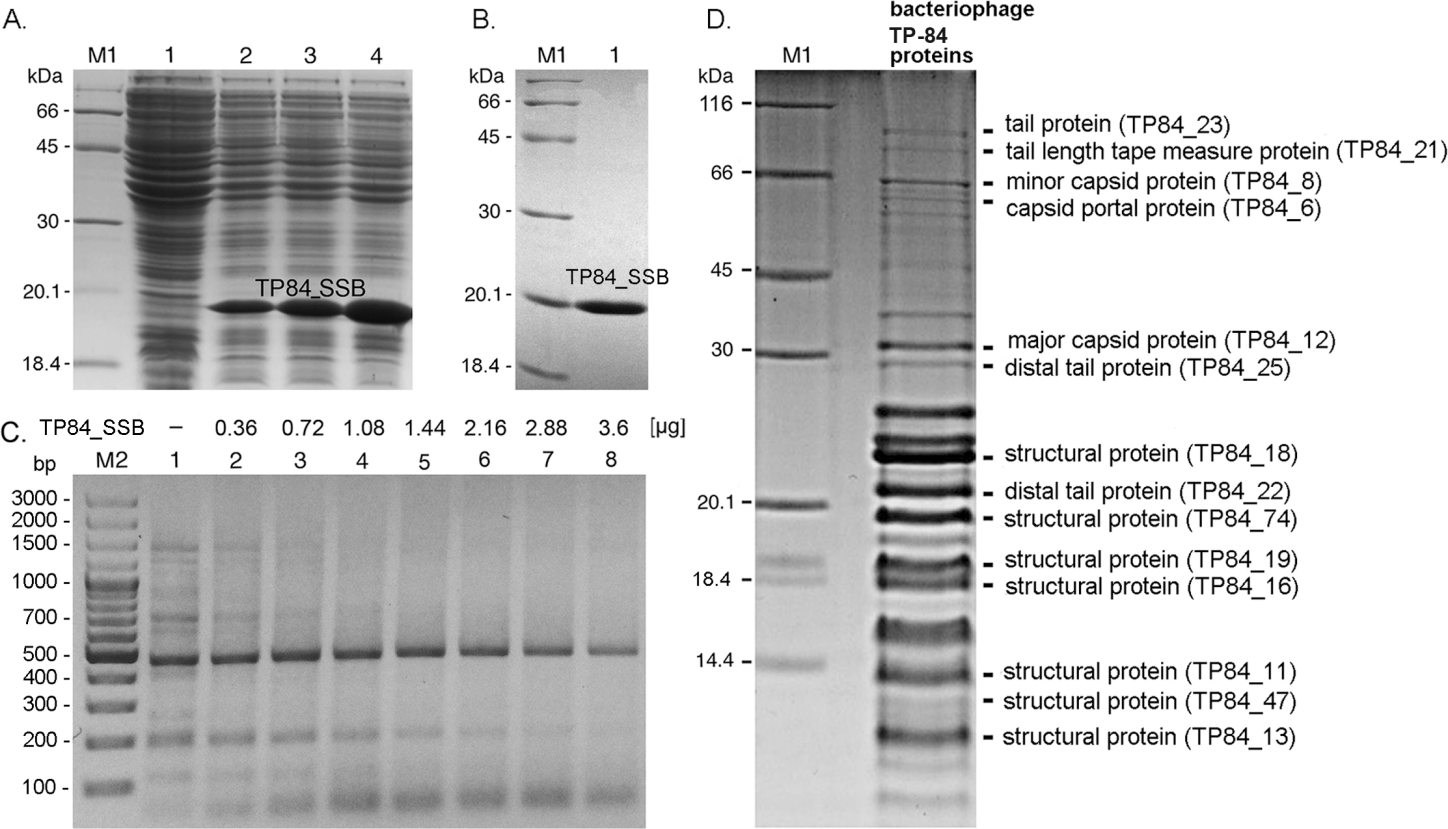
Experimental validation of TP-84 SSB and structural proteins. Panels A-C. Expression, purification and functional assay of TP-84 SSB-His_6_ protein. Lanes M1, molecular weight protein marker, LMW-SDS Marker (GE Healthcare). Panel A. SDS-PAGE analysis of the recombinant *E*. *coli* TOP10 [pBADMycHisA-TP-84_SSB] cells induction time course. Lane 1, *E*. *coli* TOP10 [pBADMycHisA-TP-84_SSB] cells prior to arabinose induction; lane 2, 2 h after induction; lane 3, 4 h after induction; lane 4, 16 h after induction. Panel B. Metal-affinity purification of TP-84 SSB-His_6_ protein. Lane 1, purified TP-84 SSB-His_6_ protein. Panel C. PCR assay of DNA-binding capabilities of TP-84 SSB-His_6_ protein. Lane M2, molecular weight DNA marker, 100-bp Plus (Thermo Scientific, USA); lane 1, PCR reaction without addition of TP-84 SSB-His_6_ protein; lane 2, 0.36 μg of TP-84 SSB-His_6_ protein added; lane 3, 0.72 μg; lane 4, 1.08 μg; lane 5, 1.44 μg; lane 6, 2.16 μg; lane 7, 2.88 μg; lane 8, 3.6 μg. Panel D. SDS-PAGE analysis of the proteins of purified TP-84 bacteriophage. Protein bands yielding MS results of high credibility are assigned to the matching TP-84 CDSs (S5 File).

**Fig 6:**
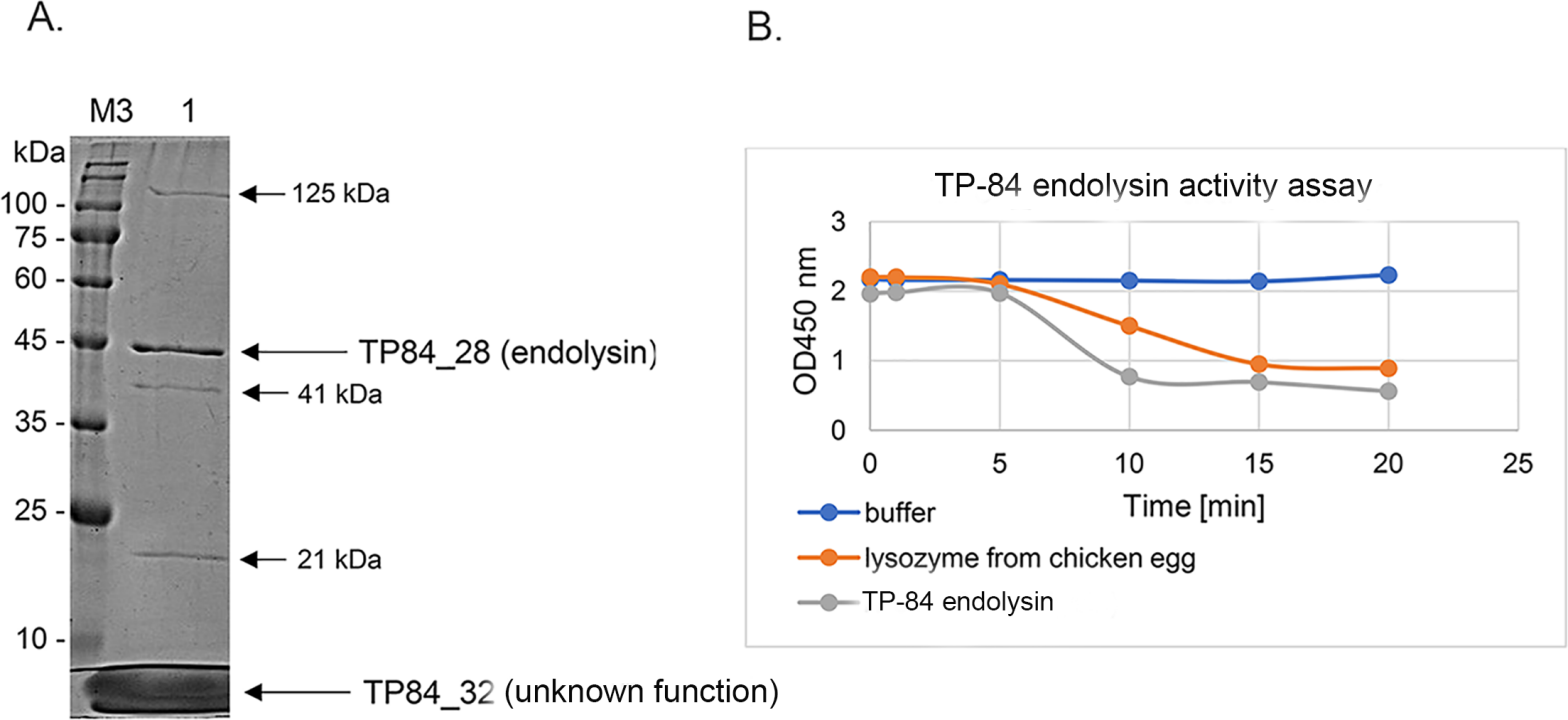
Experimental validation of TP-84 endolysin activity. Panel A. SDS-PAGE of combined peak fractions no 2–6 from CM-Sephadex chromatography (S4 File). The column was used to purify TP-84-infected, *G*. *stearothermophilus* cell lysate proteins. Lane M3, molecular weight protein marker, PM2500 (SMOBIO). Lane 1, cation exchange chromatography peak fractions from purification of the TP-84 lysate proteins. Panel B. Graph showing *E*. *coli* cell *in vitro* wall lysis as a decrease in turbidity measured at 450 nm. Blue line, control reaction buffer. Red line, hen egg lysozyme. Grey line, peak fractions 2–6 from purification of the TP-84 lysate proteins.

**Fig 7:**
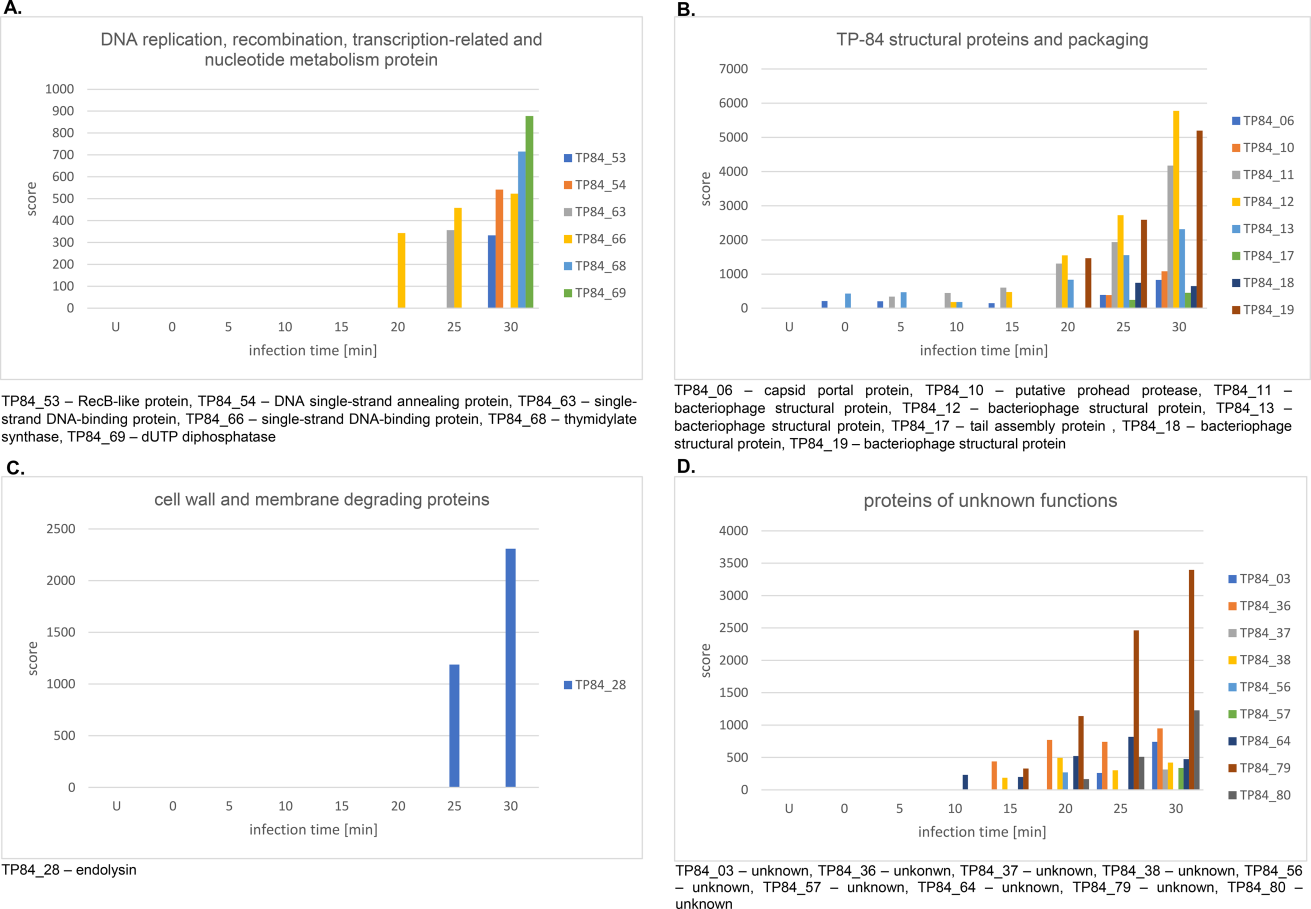
Experimental validation of TP-84 proteins biosynthesized during the infection time-course. Samples for MS analysis were taken from *G*. *stearothermophilus* cultures at time intervals: U, uninfected control prior to infection; 0, sample taken immediately upon TP-84 addition; 5, 5 min after infection; 10, 10 min; 15, 15 min; 20, 20 min; 25, 25 min; 30, 30 min. Panel A-D show graphs for each TP-84 protein detected in the culture samples, grouped according to their function. Panel A) DNA replication, recombination, transcription-related and nucleotide metabolism protein. Panel B). Structural proteins and packaging. Panel C) Cell wall and membrane degrading proteins. Panel D) Unknown function proteins.

Bacteriophage TP-84 does not encode any tRNAs but annotation reveals 81 CDSs coding for polypeptides of 4 kDa or larger. Interestingly, all CDSs are oriented in the same direction, pointing to the dominant transcription direction (Fig 3, S1 File). The genome is tightly packed with CDSs, typically spaced by several-to-tens bp or often overlapping, with the largest gap of 265 bp located between CDSs TP84_55 and TP84_56. There are two putative promoters in this gap (Fig 3, S1 Table). This phenomenon was also observed in other bacteriophages [19]. Based on a BLASTP homology search, a hypothetical function could be assigned to 31 CDSs. The annotated functions of 14 CDSs were assigned or confirmed by TP-84 particle proteomic analyses (Fig 5, S5 File), 1 CDS by gene cloning, expression and functional assay (Fig 5), 2 CDSs by purification from infected *G*. *stearothermophilus* cell lysates (Fig 6, S4 File) and 24 CDSs were confirmed to code for and produce proteins by MS of *G*. *stearothermophilus* cells during time-course infection analysis, which included proteins of unknown function (Fig 7, S6 File). Table 1 summarizes the locations of CDSs, their length, orientations, coded polypeptide length, molecular weights, isoelectric points and putative and/or confirmed functions. S1 File shows the entire TP-84 genome sequence with marked CDSs, amino acid (aa) sequences, promoters and terminators. S2 File containing TP-84 genome sequence is in txt format.

**Table 1:** Putative and experimentally confirmed CDSs of bacteriophage TP-84 genome and their functions.

CDS name	CDS length (bp)	Location in the genome (bp)	CDS arbitrary orientation	Polypeptide length (aa)	Predicted polypeptide molecular weight (kDa)	Experimentally determined polypeptide molecular weight (kDa)	Predicted isoelectric point	Hypothetical function (analysis)	Confirmed by proteomic analysis
TP84_01	567	13- 579	+	188	22.4	ND	7.76	terminase, small subunit	ND
TP84_02	1299	576- 1874	+	432	50.0	ND	7.74	terminase, large subunit	ND
**TP84_03** (S5 File)	687	1868- 2554	+	228	26.3	26.3 ^(T)^	9.17	unknown	unknown ^(T)^
TP84_04	354	2586- 2939	+	117	13.6	ND	4.36	unknown	ND
TP84_05	198	2957- 3154	+	65	7.5	ND	4.63	unknown	ND
**TP84_06** (S5 and S6 Files)	1629	3174- 4802	+	542	62.1	62.3 ^(S)^	4.96	capsid portal protein	bacteriophage structural protein ^(^^S^^,^^T^^)^
TP84_07	147	4799- 4945	+	48	5.3	ND	7.94	unknown	ND
**TP84_08** (S5 File)	2046	4962- 7007	+	681	78.0	ND	8.71	minor capsid protein	bacteriophage structural protein ^(S)^
TP84_09	210	7004- 7213	+	69	8.2	ND	4.89	putative membrane-associated protein	ND
**TP84_10** (S6 File)	828	7441- 8268	+	275	31.6	ND	4.70	putative prohead protease	putative prohead protease ^(T)^
**TP84_11** (S5 and S6 Files)	408	8283- 8690	+	135	14.0	14.0 ^(^^S^^,^^T^^)^	5.84	unknown	bacteriophage structural protein ^(^^S^^,^^T^^)^
**TP84_12** (S5 and S6 Files)	993	8736- 9728	+	330	37.8	37.8 ^(^^S^^,^^T^^)^	4.98	major capsid protein	bacteriophage structural protein ^(^^S^^,^^T^^)^
**TP84_13** (S5 and S6 Files)	195	9802- 9996	+	64	7.2	7.2 ^(T)^	9.19	unknown	bacteriophage structural protein ^(T)^
TP84_14	330	10032–10361	+	109	12.2	ND	5.16	unknown	ND
TP84_15	324	10358–10681	+	107	11.9	ND	6.26	unknown	ND
**TP84_16** (S5 File)	489	10671–11159	+	162	18.7	18.7 ^(S)^	6.26	unknown	bacteriophage structural protein ^(S)^
**TP84_17** (S6 File)	378	11162–11539	+	125	14.6	14.6 ^(T)^	5.35	tail assembly protein	tail assembly protein ^(T)^
**TP84_18** (S5 and S6 Files)	558	11554–12111	+	185	20.3	20.4 ^(^^S^^,^^T^^)^	4.38	unknown	bacteriophage structural protein ^(^^S^^,^^T^^)^
**TP84_19** (S5 and S6 Files)	507	12220–12726	+	168	19.3	19.3 ^(^^S^^,^^T^^)^	4.90	unknown	bacteriophage structural protein ^(^^S^^,^^T^^)^
TP84_20	375	12687–13061	+	124	14.7	ND	9.80	unknown	ND
**TP84_21** (S5 File)	2598	13081–15678	+	865	93.1	93.1 ^(S)^	9.70	tail length tape-measure protein	bacteriophage structural protein ^(S)^
**TP84_22** (S5 File)	738	15684–16421	+	245	27.8	27.9 ^(S)^	5.15	distal tail protein	bacteriophage structural protein ^(S)^
TP84_23	2634	16438–19071	+	877	99.3	99.4 ^(S)^	5.06	tail protein	bacteriophage structural protein ^(S)^
TP84_24	210	19061–19270	+	69	7.9	ND	4.59	unknown	ND
**TP84_25** (S5 File)	900	19283–20182	+	299	34.5	34.4 ^(S)^	5.35	distal tail protein	bacteriophage structural protein ^(S)^
TP84_26	2976	20179–23154	+	991	112.2	ND	5.49	glycosylase	ND
TP84_27	432	23240–23671	+	143	15.5	ND	6.20	holin	ND
**TP84_28** (S6 File)	1185	23676–24860	+	394	44.2	44.2 ^(^^T^^,^^P^^)^	9.67	endolysin	endolysin ^(^^T^^,^^P^^,^^B^^)^
TP84_29	201	24877–25077	+	66	7.2	ND	8.03	membrane-associated protein	ND
TP84_30	573	25329–25901	+	190	21.7	ND	4.98	unknown	ND
TP84_31	156	26023–26178	+	51	6.0	ND	6.15	putative membrane protein	ND
**TP84_32** (S4 File)	255	26235–26489	+	84	9.8	9.7 ^(P)^	9.70	unknown	unknown ^(P)^
TP84_33	291	26597–26887	+	96	11.0	ND	5.10	unknown	ND
TP84_34	255	26884–27138	+	84	9.6	ND	4.48	putative membrane protein	ND
TP84_35	207	27135–27341	+	68	7.9	ND	11.60	unknown	ND
**TP84_36** (S6 File)	237	27353–27589	+	78	9.3	9.3 ^(T)^	9.91	unknown	unknown ^(T)^
**TP84_37** (S6 File)	396	27698–28093	+	131	14.9	14.8 ^(T)^	6.41	unknown	unknown ^(T)^
**TP84_38** (S6 File)	201	28090–28290	+	66	7.4	7.38^(T)^	6.73	unknown	unknown ^(T)^
TP84_39	216	28403–28618	+	71	8,1	ND	7.96	unknown	ND
TP84_40	210	28615–28824	+	69	8.2	ND	9.39	unknown	ND
TP84_41	273	28824–29096	+	90	10.6	ND	9.69	unknown	ND
TP84_42	162	29169–29330	+	53	6.4	ND	5.71	unknown	ND
TP84_43	309	29330–29639	+	102	11.0	ND	9.81	unknown	ND
TP84_44	132	29708–29839	+	43	5.0	ND	10.62	unknown	ND
TP84_45	231	29839–30069	+	76	8.5	ND	9.75	unknown	ND
TP84_46	213	30071–30283	+	70	7.8	ND	10.01	unknown	ND
**TP84_47** (S5 File)	381	30252–30632	+	126	13.8	13.8 ^(S)^	7.85	putative membrane protein	bacteriophage structural protein ^(S)^
TP84_48	2265	30527–32791	+	754	86	ND	7.09	unknown	ND
TP84_49	474	32871–33344	+	157	18.0	ND	5.85	unknown	ND
TP84_50	159	33468–33626	+	52	5.8	ND	6.53	unknown	ND
TP84_51	159	33832–33990	+	52	6.1	ND	10.41	unknown	ND
TP84_52	237	34030–34266	+	78	8.5	ND	5.36	transcriptional regulator (HTH_XRE family)	ND
**TP84_53** (S6 File)	1017	34238–35254	+	338	38.5	ND	5.33	RecB-like protein	RecB-like protein ^(T)^
**TP84_54** (S6 File)	1020	35270–36289	+	339	38.8	ND	5.78	DNA single-strand annealing protein	DNA single-strand annealing protein ^(T)^
TP84_55	222	36337–36558	+	73	8.8	ND	4.54	unknown	ND
**TP84_56** (S6 File)	183	36924–37106	+	60	7.4	7.4 ^(T)^	9.25	unknown	unknown ^(T)^
**TP84_57** (S6 File)	783	37127–37909	+	260	30.3	30.4 ^(T)^	8.57	unknown	unknown ^(T)^
TP84_58	318	37942–38259	+	105	12.3	ND	4.92	replicative helicase inhibitor	ND
TP84_59	1305	38259–39563	+	434	49.1	ND	5.53	replicative DNA helicase	ND
TP84_60	672	39632–40303	+	223	26.4	ND	8.41	unknown	ND
TP84_61	435	40281–40715	+	144	16.7	ND	9.08	HNH homing endonuclease	ND
TP84_62	147	40699–40845	+	48	5.9	ND	9.10	unknown	ND
**TP84_63** (S6 File)	468	40851–41318	+	155	17.4 (18.3)*	17.4 ^(T)^ (18.4*)	5.16	single-stranded DNA-binding protein-	single-stranded DNA-binding protein ^(^^C^^,^^B^^,^^T^^)^
**TP84_64** (S6 File)	240	41343–41582	+	79	9.3	9.3 ^(T)^	6.89	unknown	unknown ^(T)^
TP84_65	150	41596–41745	+	49	5.5	ND	8.10	putative membrane protein	ND
**TP84_66** (S6 File)	270	41751–42020	+	89	10.4	ND	5.92	single-stranded DNA-binding protein	single-stranded DNA-binding protein ^(T)^
TP84_67	183	42023–42205	+	60	6.9	ND	4.58	unknown	ND
**TP84_68** (S6 File)	987	42209–43195	+	328	38.6	38.5 ^(T)^	6.91	thymidylate synthase	thymidylate synthase ^(T)^
**TP84_69** (S6 File)	501	43200–43700	+	166	20.0	20.1 ^(T)^	5.67	dUTP diphosphatase	dUTP diphosphatase ^(T)^
TP84_70	330	43704–44033	+	109	12.7	ND	9.47	unknown	ND
TP84_71	246	44014–44259	+	81	9.4	ND	10.21	unknown	ND
TP84_72	504	44246–44749	+	167	19.3	ND	7.70	Holliday junction-specific endonuclease	ND
TP84_73	264	44746–45009	+	87	10.4	ND	5.60	unknown	ND
**TP84_74** (S5 File)	546	44984–45529	+	181	21.4	21.3 ^(S)^	9.19	unknown	bacteriophage structural protein ^(S)^
TP84_75	114	45546–45659	+	37	4.0	ND	3.76	unknown	ND
TP84_76	126	45675–45800	+	41	4.8	ND	5.54	unknown	ND
TP84_77	186	45857–46042	+	61	7.2	ND	9.19	unknown	ND
TP84_78	204	46047–46250	+	67	7.5	ND	12.16	unknown	ND
**TP84_79** (S6 File)	354	46380–46733	+	117	13.4	13.4 ^(T)^	7.82	unknown	unknown ^(T)^
**TP84_80** (S6 File)	366	46741–47106	+	121	14.0	13.9 ^(T)^	6.82	unknown	unknown ^(T)^
TP84_81	141	47379–47519	+	46	5.3	ND	9.59	unknown	ND

(S)—TP-84 acetone precipitate was subjected to SDS-PAGE and individual protein bands were excised and in-gel trypsin-digested and analysed by MS.

(C)—CDS with assigned function, subjected to gene cloning and expression.

(B)–TP-84 proteins confirmed by a biochemical assay.

(P)–proteins purified from infected *G*. *stearothermophilus* cells lysates and subjected to MS.

(T)–TP-84 proteins detected by MS in the infected *G*. *stearothermophilus* cells.

ND–not determined

* SDS-PAGE molecular weight determination of the recombinant version of SSB-His_6_ protein, containing additional 7 aa residues at the N-terminus as based on calculations from Fig 5A.

Using Phobius, six putative membrane proteins were identified. Five of these (TP84_09, TP84_31, TP84_34, TP84_47 and TP84_65) exhibit one transmembrane domain (TMD), while TP84_27 contains two TMDs (Table 1, Fig 3).

In the sections below, we will discuss the categories of proteins encoded by the TP-84 genome.

### Nucleotide metabolism

Related to replication, precursor generating proteins include a thymidylate synthase (TP84_68) and a dUTP diphosphatase (TP84_69)–identified using pfam [20] motifs Thy1 (pfam02511) and dUTPase_2 (PF08761.7), respectively. Interestingly, the former protein shows greatest similarity to bacterial proteins from the genera *Desulfotomaculum*, *Clostridium* and *Clostridioides*. These genes are located within the DNA replication/recombination/transcription cluster (Table 1, Fig 3, S1 File).

### DNA replication, recombination- and transcription related proteins

No RNA or DNA polymerase-coding genes were found in the phage genome indicating that TP-84 relies on the host’s transcriptional and replication enzymes. Of interest is the observation that TP84_52 encodes a putative transcriptional regulator (TR) related to the HTH_XRE (cd00093) helix-turn-helix XRE-family of proteins. HHpred reveals a sequence similarity to *Salmonella* phage P22 C2 repressor (RCSB Protein Data Bank [21], accession number 1adr). Phylogenetic trees were constructed using the large subunit terminase (TerL) and thymidylate synthase (Ts) proteins (Fig 4). In both cases, the closest homologs were found amongst the order *Clostridiales* (TerL) and family *Bacillaceae* (Ts). This raises the interesting question as to whether TP-84 is a lytic variant of a temperate bacteriophage.

The accessory TP-84 replication proteins include a replicative helicase inhibitor (TP84_58) containing an inhibitor_G39P (pfam11417) domain. This 105-aa residue protein probably inhibits the replicative DNA helicase encoded by TP84_59. The latter protein, which shows closest homology to *Bacillus* helicases, possesses a DnaB-like helicase N terminal domain (pfam00772) and a C-terminal P-loop_NTPase (cl21455) domain containing Walker box motifs. Lastly, TP84_63 and TP84_66 encode single-stranded DNA-binding (SSB) proteins.

The recombination proteins include a RecB-like protein (TP84_53) and DNA single-strand annealing protein (TP84_54), which is a member of the ERF (pfam04404) superfamily. In addition, there is a RecU-related protein encoded by TP84_72, which functions as a Holliday junction-specific endonuclease. The SSB protein, encoded by TP84_63 CDS was selected for cloning and expression. An assumed benefit of this selection was the potential practical applicability of this thermostable SSB. The CDS were PCR amplified with forward primers introducing DNA sequences coding for a histidine tag incorporated at the N-terminus for the purpose of metal affinity purification. We cloned the TP84_63 CDS into the expression vector pBAD-MycHisA under an arabinose-inducible *araBAD* promoter control and expressed this protein in *E*. *coli*. The recombinant protein production was analysed by SDS-PAGE. The appearance of a suitable protein band correlated with induction time. The recombinant SSB-His_6_ protein with an apparent molecular weight of 17.4 kDa was observed (Fig 5A and 5B), which matches the predicted molecular weight for histidine-tagged TP-84 SSB very well (Table 1). The recombinant SSB-His_6_ protein was purified on a Ni^2+^ loaded affinity column and subjected to functional assays. The obtained results confirmed those from the bioinformatics CDS assignment. Fig 5C shows PCR amplification of a *Candida albicans* diagnostic, 470 bp genomic segment. In lane 1 there are 15 or more undesired bands in addition to the expected 470 bp fragment. As the amount of SSB-His_6_ protein added increases, the reaction is gradually purified. Thus, the SSB-His_6_ protein apparently interacts with DNA, facilitating correct annealing of PCR primers to single-stranded template DNA. As a result, SSB-His_6_ has the capability to improve problematic PCR reactions (Fig 5C) [22], similarly to other SSB proteins [23,24]. Detailed studies of the TP-84 SSB protein will be published elsewhere.

### TP-84 structural proteins and packaging

The assigned hypothetical functions of the identified CDSs were experimentally confirmed by MS analysis of the purified TP-84 bacteriophage particles. For that purpose whole TP-84 particles were denatured and resolved into individual proteins by SDS-PAGE, followed by MS analysis of the protein bands excised from the polyacrylamide gel (Table 1, S5 and S6 Files). Fourteen structural proteins were detected with at least two peptides sequence coverage: (TP84_06, TP84_08, TP84_11, TP84_12, TP84_13, TP84_16, TP84_18, TP84_19, TP84_21, TP84_22, TP84_23, TP84_25, TP84_47 and TP84_74). Eight of them matched the genes detected by bioinformatics analysis and six were previously assigned as ‘unknown’. Molecular weights of all the MS-detected proteins matched very well those predicted by bioinformatics analysis. Moreover, a few additional protein bands, not validated by MS, were detected in polyacrylamide gels after SDS-PAGE (Fig 5D). We speculate that non-validated proteins belong to the ‘unknown’ category.

We also suppose that the genes from TP84_01 to TP84_25–38.4% of the genome—encode proteins involved in the synthesis and assembly of the phage capsid and tail. The function of seventeen proteins encoded by this gene cluster was determined (TP84_01, TP84_02, TP84_06, TP84_08, TP84_09, TP84_10, TP84_11, TP84_12, TP84_13, TP84_16, TP84_17, TP84_18, TP84_19, TP84_21, TP84_22, TP84_23 and TP84_25) (Figs 3,5 and 7, Table 1). As with most bacteriophages, TP-84 encodes a pair of proteins involved in DNA packaging: TP-84_01 (putative terminase, small subunit) and TP84_02 (putative terminase, large subunit) (Fig 3, Table 1). Both mentioned genes are located within the described gene cluster. Following the gene order across the genome, TP84_06 encodes the portal protein, (HHpred relationship to *Bacillus* phage SSP1 portal protein 2jes); TP84_08, minor capsid protein (evidence: Pfam motifs PF06152.7 phage_min_cap2); TP84_09, a putative membrane protein, TP84_10, a putative prohead protease (HHpred homolog 2o8l (V8 protease, from *Staphylococcus aureus*)); TP84_11, a structural protein; TP84_12, major capsid protein (HHpred sequence similarity to 3bqw, a putative capsid protein from an *E*. *coli* CFT073 prophage); TP84_13, a structural protein; TP84_16, a structural protein; TP84_17, a tail assembly protein, detected as HHpred sequence similarity to 5a2 viral assembly, head-to-tail interface from *Bacillus* phage SPP1; TP84_18, a structural protein; TP84_19, a structural protein; TP84_21, a tail length tape-measure protein; TP84_22, distal tail protein, detected as HHpred sequence similarity to 2x8kt—the distal tail protein (19.1) from *Bacillus* phage SPP1; TP84_23, tail protein, detected as HHpred sequence similarity to 1wru—a 43 kDa tail protein from *Escherichia* phage Mu; TP84_25, distal tail protein, detected as HHpred sequence similarity to 4div—a distal tail protein (ORF46) from *Lactococcus* phage TP901-1 (Table 1, Figs 3 and 5; S1 File). The well-studied, from a genetics and proteomics standpoint, thermophilic bacteriophage offers a unique opportunity to construct a thermostable phage display system. Such a system can be of great value for the construction of a new generation of stable vaccines and application in regenerative medicine as a macromolecular vehicle for the delivery of bioactive peptides and polypeptides limited to a wound area, among others. The recombinant TP-84-based constructs are under development and will be published elsewhere.

In addition, six more genes, possibly coding for other structural or membrane proteins, have been identified outside of the cluster (TP84_29, TP84_31, TP84_34, TP84_47, TP84_65 and TP84_74) (Fig 3, Table 1).

Since TP-84 dissociates in the presence of EDTA [15], one could assume that divalent ions, particularly Ca^2+^, are involved in the stabilization of the virions. Furthermore, many bacteriophages require calcium ions for infectivity [25,26]. Since many calcium-binding proteins share an EF-hand motif [27], we screened the TP-84 putative structural proteins in Prosite [28] for the PDOC00018 signature sequence. None was found.

TP84_26 codes for a glycosylase, detected by HHpred sequence similarity to putative sporulation-specific glycosylase YDHD from *Bacillus subtilis* (3cz8; Probability = 100.00, E-value = 3.2e-33, Score = 307.02). Interestingly, it shows homology to a number of proteins from *Bacillus* and *Geobacillus* species, and one *Geobacillus* phage GBK2 protein (GenBank YP_009010491). Two interpretations can be made concerning the function of this protein; the first being is that it is a tail fibre protein possessing enzymatic activity. The alternate hypothesis is that like TP84_28 it is involved in lysis.

### Cell wall and membrane degrading proteins

The TP-84 bacteriophage produces proteins that include two hypothetical cell wall and cytoplasmic membrane lytic proteins. These proteins are grouped in a small cluster in the middle of the TP-84 genome: TP84_27, holin, with two transmembrane domains detected by Phobius and TP84_28 –endolysin, detected with HHpred sequence similarity to 4kru, which is an autolytic lysozyme from *Clostridium* phage PHISM101 (Table 1, Fig 3, S1 File).

TP-84 bacteriophage glycosylase functionality was evaluated by the purification of TP-84 encoded proteins from cleared *G*. *stearothermophilus* cell lysates using cation-exchange chromatography, followed by SDS-PAGE analysis, MS confirmation and a biochemical assay for cell wall degradation activity. Fig 6A shows the results of SDS-PAGE, where 5 protein bands are visible. For 2 dominant bands, MS analysis revealed that one band is TP84_28 endolysin and the second is TP84_32 of unknown function. Functional analysis of the chromatography peak fractions revealed strong lytic activity toward lyophilised and buffer-suspended *E*. *coli* cells compared to hen egg lysozyme solution. Since there are 3 additional protein bands present on the SDS-PAGE gel in the purified endolysin preparation (Fig 6, S4 File), one cannot exclude the rather remote possibility that the activity is attributed to a protein other than TP84_28, even though this protein should have such an activity based on bioinformatics analysis. Another possibility is that cell wall lysis is conducted cooperatively by more than one protein present in the preparation. One such candidate is TP84_26 since it is a putative glycosylase (Table 1), is present in the soluble fraction of TP-84-lysed *G*. *stearothermophilus* cells and has a molecular mass close to the band of the largest molecular mass (see below) present on the SDS-PAGE gel (Fig 6A). The thermostable TP-84 lysozyme is of potential biotechnology significance whenever a high temperature bacterial cell lysis process would be required. Detailed studies of the TP-84 lysozyme will be published elsewhere. The remaining 3 protein minor bands had SDS-PAGE-determined molecular weights of: 21 kDa, 41 kDa and 125 kDa. The closest matches compared to the molecular weights predicted by bioinformatics are: 21 kDa band–TP84_74 (structural protein), 41 kDa band–TP84_54 (DNA single-strand annealing protein), 125 kDa–TP84_26 (glycosylase). Nevertheless, one cannot exclude the possibility that these 3 protein bands are of host origin as their amounts were insufficient for MS confirmation.

### Time course of TP-84-coded proteins biosynthesis in infected *G*. *stearothermophilus* cells

To further validate the bioinformatics analysis and to obtain some insight into the TP-84 life cycle, a time-course experiment was conducted. Samples were taken every 5 min over approx. 30 min of the TP-84 growth cycle and analysed by MS. A total of 24 proteins were detected: (*i*) DNA replication, recombination, transcription-related and nucleotide metabolism protein: TP84_53 –RecB-like protein, TP83_54 –DNA single-strand annealing protein, TP84_63 –single-stranded DNA-binding protein, TP84_66 –single-stranded DNA-binding protein, TP84_68 –thymidylate synthase, TP84_69 –dUTP diphosphatase (Fig 7A); (*ii*) TP84 structural proteins and packaging: TP84_06 –capsid portal protein, TP83_10 –putative prohead protease, TP84_11 –bacteriophage structural protein, TP84_12 –structural protein, TP84_13 –structural protein, TP84_17 –tail assembly protein, TP84_18 –structural protein, TP84_19 –structural protein (Fig 7B); (*iii*) cell wall and membrane degrading proteins: TP84_28 –endolysin (Fig 7C); (*iv*) unknown function proteins: TP84_03, TP84_36, TP84_37, TP84_38, TP84_56, TP84_57, TP84_64, TP84_79, TP84_80 (Fig 7D). Including the findings shown in Figs 5 and 6, the total number of validated bioinformatics-predicted TP-84 proteins is 32. Furthermore, the general strength of the MS signal (score) depends on the protein amount, so one can trace TP-84 protein biosynthesis kinetics. The score-protein amount relationship is not precisely quantitative, as a number of factors affect the signal strength. Nevertheless, clear trends were observed for most of the structural proteins, which dominate the detected biosynthesis upwards of 20 min past infection. The TP84_28 endolysin starts to appear at 25 min, while TP84_68 –thymidylate synthase and TP84_69 –dUTP diphosphatase are detected only at 30 min. There is also strong biosynthesis of TP84_79 of unknown function, correlated with the biosynthesis of structural and packaging proteins, thus we hypothesize that it also belongs to this group.

## Conclusions

- The genome of TP-84 bacteriophage (*Caudovirales*, *Siphoviridae*), the thermophilic 47.7-kb bacteriophage infecting *G*. *stearothermophilus*, was sequenced and annotated. The sequence essentially shows no homology to other sequenced bacteriophage genomes. TP-84 should be considered a new species in the newly approved *Tp84virus* genus within the *Siphoviridae* (S3 File). The TP-84 morphology is confirmed by electron microscopy (Fig 1) and the presence of a typical siphoviral tail tape measure protein.
- The TP-84 bacteriophage shows only a peripheral relatedness to any of the other *Geobacillus* phages that have been sequenced.
- The genome organization shows a unidirectional organization of 81 ORFs.
- The functions of 37 ORFs have been assigned showing a clustering of DNA-metabolism-associated genes, cell wall/membrane lysing-associated genes and capsid-forming associated genes.
- The genome contains five putative promoter-like sequences showing similarity to the host promoter consensus sequence and ten putative rho-independent terminators.
- Proteomics analysis confirmed 32 CDSs/proteins out of 81 CDSs predicted by bioinformatics analysis.
- Purified TP-84 SSB protein was shown to improve problematic PCR reactions, while purified TP-84 lysozyme is a robust, thermostable, cell wall lysing enzyme. Both are suitable for biotechnology applications.

## Materials and methods

### Bacteriophage propagation, purification and TP-84 particle-associated proteins analysis

#### TP-84 cultivation

Bacteriophage TP-84 was cultivated by infection of bacterial host strain *G*. *stearothermophilus* strain 10, using 1 L portions of rich medium supplemented with calcium and magnesium ions, known to highly increase TP-84 yield as previously described [13], with minor modifications. Liquid TYM medium for the host cultivation and further infection contained (per 1000 ml): Pepton K (pancreatic casein hydrolysate) (BTL, Poland) 20 g, yeast extract (BTL) 4 g, MgCl_2_.6H_2_O 2 g, CaCl_2_.H_2_O 0.73 g, fructose 0.5%. Culturing was conducted at 58°C with vigorous aeration by shaking in 5 L flasks at 220 rpm. Infection was typically performed in 1 L cultures grown in TYM until OD_540_ reached 0.8–0.9, then an additional fructose portion was added to 0.5% and the culture was grown for an additional 30 min until an OD_540_ of approx. 1.2. Then it was infected with TP-84 at MOI of 0.01. For bacteriophage plating, TYM was supplemented with 20 g agar / 1000 ml (bottom agar) or 6 g agar / 1000 ml (top agar). Bacterial lawn was prepared using 50 μl of overnight, 20-h-old *G*. *stearothermophilus* strain 10 culture of OD_540_ = 4 mixed with 2.5 ml of top agar. Immediately before incubation with the bacteriophage TP-84 sample, the overnight host culture was supplemented with additional 0.5% fructose from a 10% stock.

#### TP-84 particle proteins analysis

The bacteriophage particles were purified using selective precipitation, followed by CsCl centrifugation as previously described [11,13]. At this purification stage, the TP-84 preparation was used for TEM imaging. For proteomics analysis, two methods for sample preparation were used: (*i*) TP-84 particles were acetone precipitated/trypsin digested and subjected to MS and (*ii*) acetone-precipitated TP-84 was subjected to SDS-PAGE on a 15% polyacrylamide gel run in Tris-tricine buffer. The individual gel slices, containing protein bands, were excised and subjected to in-gel trypsin digestion and MS analysis. The protein acetone precipitation was conducted as follows. Chilled acetone (400 μl) was added to 100 μl purified bacteriophage particles suspension (PFU = 1.8 10^10^). Then, the mixture was incubated at -20°C for 60 min. The resulting protein pellet was centrifuged at 13000 x g, 4°C for 20 min and suspended in 50 μl PBS buffer (1xPBS: 137 mM NaCl, 2.7 mM KCl, 10 mM Na_2_HPO_4_, 1.8 mM KH_2_PO_4_). Entire TP-84 particles were subjected to trypsin (52 ng trypsin) digestion at 37°C overnight. For individual protein analysis, 10 μl PBS suspension samples were subjected to SDS-PAGE in 15% polyacrylamide gel run in Tris-tricine buffer. Separated protein bands were cut out and 100 mM ammonium bicarbonate in HPLC-grade acetonitrile was added (1:1, vol/vol) and slices incubated with occasional mixing for up to 30 min, depending on protein band staining intensity. 500 μl of acetonitrile was added and the incubation continued at room temperature with occasional mixing, until the gel fragments become white and shrank, then the acetonitrile was removed. Trypsin buffer was added to gel fragments (50 μl) and samples were incubated with 52 ng trypsin overnight at 37˚C [29]. 50 μl of the digestion reactions were subjected to MS. Two MS instruments were used: HPLC—Shimadzu Nexera 2, MS—SCIEX TT5600+ on Phenomenex column with C-18 resin, flow rate 0,3 ml/min, buffer A: 0,02% HCOOH, buffer B: 80% acetonitrile with 0,02% HCOOH. The identity of the protein was confirmed based on score points and labelled with more than two peptide fragments (S4 and S5 Files).

### DNA extraction, genome sequencing and bioinformatics analysis

The genomic DNA was isolated by EDTA addition to the purified bacteriophage suspension, to the final concentration of 50 mM, triple extracted with phenol saturated with 20 mM phosphate buffer pH 7.0, and twice with chloroform. The DNA was precipitated with two volumes of ethyl alcohol, washed with 70% ethanol, dried and suspended in TE buffer (10 mM Tris-HCl, pH 8.0 at 25°C, 1 mM EDTA). Purified TP-84 bacteriophage DNA was subjected to Next Generation Sequencing using an Illumina HiSeq 2000 genomic sequencer in the PE250 mode. The genomic library was prepared containing short inserts of 100–600 bp. The reads were assembled and analysed using different programs: CutAdapt [30], CLC Genomic Workbench (https://www.qiagenbioinformatics.com/products/clc-genomics-workbench/) and PROKKA [31]. A total of 140,683,713 nt sequences were obtained, 626,934 paired sequenced, resulting in a final assembled contig of 47,703 bp (GenBank KY565347.1) S1 File. The obtained sequence was further analysed using software: MyRAST [32], Kodon (http://www.applied-maths.com/kodon), BLASTP (https://blast.ncbi.nlm.nih.gov, [33]), HHpred [34] and SnapGene (http://www.snapgene.com), as well as for visual corrections. Detection of genes encoding membrane-associated proteins was done using Phobius [35]. The GC content distribution was calculated with GC Content Calculator (Biologics International Corp, Indianapolis, USA; http://www.biologicscorp.com/tools/GCContent/). Putative rho-independent terminators were searched for using ARNold [36].

Homologs of the large subunit terminase (TerL) and thymidylate synthase (Ts) proteins were identified using BLASTP and Newick formatted trees were generated using the "One click" mode at phylogeny.fr [37]. The data was then massaged using FigTree (http://tree.bio.ed.ac.uk/software/figtree/).

### TP-84_63 CDS cloning, expression, recombinant SSB-His_6_ purification and functional assay

#### Cloning of the TP-84_63 CDS

The 468-bp CDS, assigned as coding for SSB protein, was PCR amplified. For that purpose, two primers were used. The mutagenic forward primer introduced a DNA segment coding for a His_6_-tag and NcoI restriction site (both underlined) in order to fuse the vector’s ATG codon: 5’-GGAGGACCATGGCTCACCATCATCATCATCATAACAATGTGACGTTAGTGGGAAGATTGACG-3’. The reverse primer introduced a BglII site (underlined) after the STOP codon: 5’- TTGAGATCTCTAAAATGGCAGATCGTCATCATTCACATAAATCGG-3’. The BglII site was introduced for ligation with the 3’ portion of the vector’s MSC. The PCR reaction was conducted in 50 μl volume, with 2 ng TP_84 genomic DNA as a template, 0.4 mM primers, 0.8 mM dNTPs, 2 mM MgCl_2_ in the manufacturer’s buffer (BLIRT, Poland). The cycling profile was as follows: 97°C for 4 min, 89°C for 20 sec (addition of DNA polymerase), 94°C for 3 min, the 30 cycles were conducted as follows: 94°C for 30 sec, 55°C for 30 sec, 72°C for 30 min and final extension at 72°C for 2 min. The obtained PCR product comprised of an extended 489 bp gene, coding for 18.3 kDa SSB-His_6_ protein of increased theoretical isoelectric point of 6.28. DNA was gel-purified (Gel Out Kit, A&A Biotechnology, Poland) and digested with NcoI and BglII. Further purification was carried out with the use of the Extract Me DNA Clean-up Kit (BLIRT) and ligated to the NcoI/BglII-digested pBAD-MycHisA vector. The ligation mixture was transformed into *E*. *coli* TOP10 cells.

#### Expression and purification of TP-84 SSB- His_6_ protein

Recombinant *E*. *coli* TOP10 [pBADMycHisA-TP-84_SSB] cells were grown in 50 ml of standard LB media at 37°C until OD_540_ = 0.4 and induced with the addition of arabinose to 0.02%. Culture samples were taken every 2 h and analysed by SDS-PAGE. The maximum expression level was obtained after 16 h, the cells were removed by centrifugation, suspended in 20 ml of ice-cold sonication buffer (50 mM Tris-HCl pH 8.0, 0.5 M NaCl, 0.01% Triton X100, 5 mM β-mercaptoethanol, 0.5 mg/ml hen egg lysozyme, 1/5 tablet of SIGMAFAST Protease Inhibitor Cocktail (Sigma, USA)) and subjected to sonication. Cell debris was spun down and supernatant was loaded onto 2 ml Ni Sepharose 6 Fast Flow (GE Healthcare, USA), equilibrated in buffer A (50 mM Tris-HCl pH 8.0, 0.5 M NaCl, 5 mM imidazole). The column was washed with 10 volumes of buffer A, 10 volumes of buffer B (50 mM Tris-HCl pH 8.0, 0.5 M NaCl, 5 mM imidazole) and the SSB- His_6_ was eluted with buffer C (50 mM Tris-HCl pH 8.0, 0.5 M NaCl, 300 mM imidazole). Fractions containing homogeneous SSB-His_6_ were dialysed against storage buffer S (20 mM Tris-HCl pH 8.0, 150 mM NaCl, 1 mM EDTA, 0.2 mM β-mercaptoethanol, 0.01% Triton X100, 50% glycerol) and stored at -20°C.

#### Functional assay of TP-84 SSB-His_6_ protein

The ability of the TP-84 SSB-His_6_ protein to enhance the specific primer extension capacity of TaqStoffel DNA Polymerase was investigated. For that purpose, a problematic PCR reaction, suffering from non-specific primer binding and unwanted product formation, was selected. In the absence of the TP-84 SSB-His_6_ protein, the PCR reaction yielded several non-specifics PCR products in addition to the specific 470-bp amplification product. The 25 μl PCR reaction contained 10 μM of both forward and reverse primers: 5’- AGAGAAGGTGAACAATTTGC-3’ and 5’-CCAACAGTATCGGCAATACCAACTCT-3’, 1x reaction buffer (10 mM Tris-HCl (pH 8.8 at 25°C), 50 mM KCl, 0.08% (v/v) Triton x-100), 1.5 mM MgCl_2_, 2 mM each dNTPs, 1 unit TaqStoffel DNA Polymerase (Innovabion, Poland), 20 ng *Candida albicans* genomic DNA and 0.36–3.6 μg TP-84 SSB-His_6_ protein.

### MS analysis

TP-84 purified and denatured particles, proteins, *G*. *stearothermophilus* cell culture were subjected to LC-MS-MS/MS analysis, which were performed at the Mass Spectrometry Laboratory IBB PAS, Warsaw and at the University of Gdansk MS Facility, Faculty of Chemistry. Gel slices containing proteins were subjected to a standard ‘in-gel digestion’ procedure. The protein disulphide bonds were reduced with 100 mM DTT (30 min at 56°C), proteins alkylated with iodoacetamide (45 min in a darkroom at room temperature) and digested with trypsin. The resulting peptides were eluted from the gel with 0.1% trifluoroacetic acid (TFA) and 2% acetonitrile (ACN) and measured by LC-MS. HPLC separation parameters: (*i*) pre-column: RP-18 (nanoACQUITY Symmetry R ® C18,Waters) and 0.1% TFA as a mobile phase; (*ii*) Nano-HPLC parameters: RP-18 column (nanoACQUITY BEH C18, Waters), flow rate 250 nl/min, gradient: 0–35% B for 70 min, solvent A: 0.05% formic acid in water, solvent B: 0.05% formic acid in ACN (IBB PAS, Warsaw) and (*iii*) HPLC—Shimadzu Nexera 2, MS—SCIEX TT5600+ on Phenomenex column with C-18 resin, flow rate 0.3 ml/min, buffer A: 0.02% HCOOH, buffer B: 80% acetonitrile with 0.02% HCOOH (University of Gdansk, Faculty of Chemistry). The column outlet was directly coupled to the ion source of the spectrometer working in the regime of data dependent MS to MS/MS switch (Orbitrap Velos mass spectrometer-Thermo Electron Corp.). The raw data were processed using Mascot Distiller followed by Mascot Search against the TP-84 proteins database. Peptides with a Mascot Score exceeding the 5% False Positive Rate threshold and with a Mascot Score exceeding 148 were considered to be positively identified. The analysis was conducted toward the determination of the molecular masses of TP-84 proteins, as well as sequence coverage of the obtained peptides after trypsin digestion for precise protein identification.

### TP-84 cell wall lysis activity purification, functional assay and analysis

Bacteriophage TP-84 was cultivated by infection of the host *G*. *stearothermophilus* strain 10, grown in 2 x 1 L in TYM medium in 5 L flasks at 58°C with vigorous aeration (220 rpm). Infection was performed in cultures that reached an OD_540_ of 0.7, were supplemented with additional fructose portion added to 0.5% and grown for 30 min until an OD_540_ of approx. 1.0. Then, the culture was infected with CsCl–purified TP-84 at MOI of 0.01 and further grown for 4 h. Bacterial debris was centrifuged from completely lysed culture—twice at 7000 x g, 4°C, 10 min. The cleared lysate was loaded onto 4 ml CM-Sephadex C-25 column, washed with 40 ml of buffer D (50 mM K/P0_4_ pH 6.5, 0.1 mM EDTA) and eluted with 12 ml of buffer E (50 mM K/P0_4_ pH 6.5, 0.1 mM EDTA, 1 M NaCl), collecting 2 ml fractions (S4 File). Collected fraction samples were analysed by MS to determine the molecular mass of SDS-PAGE isolated proteins (Fig 6, S4 File). The mixed peak column fractions 2–6 (S4 File) sample was subjected to functional assay and MS sequenced coverage of obtained peptides after trypsin digestion. To evaluate TP-84 endolysin activity, the *in vitro* lyophilised *E*. *coli* cells lysis assay was used, based on spectrophotometric quantitation of a decrease of the cells suspension turbidity [38]. For that purpose the substrate *E*. *coli* cells were prepared as follows: *E*. *coli* were grown in 100 ml LB medium with aeration to a concentration of about 10^8^ CFU/ml (OD_600_ = 0.75) then chilled, washed by centrifugation with 0.05 M Tris-HCl pH 7.4, and suspended in 1 ml of the same buffer. The cell slurry was rapidly frozen at liquid nitrogen temperatures and then lyophilized for 4 days. The dried powder was stored tightly closed at room temperature. For the endolysin assay the lyophilised cells were suspended in 0.9 ml of reaction buffer F (50 mM Tris-HCl pH 7.4), incubated with 0.1 ml of the endolysin sample for 20 min at 37°C, and the absorbance at 450 nm was measured. Changes in OD during the incubation were plotted against time using a recording spectrophotometer. Purified chicken lysozyme was used as a control to generate a standard curve: 1 U of the enzyme activity corresponding to a 0.001 decrease in OD. The incubation temperature of 37°C was used for comparative purposes, however, the TP-84 endolysin is expected to be fully active at 58°C as this is the optimal growth temperature for TP-84.

### *G*. *stearothermophilus* TP-84 infection time course analysis

Infection time-course experiments were conducted in in 10 ml of TYM medium, inoculated with *G*. *stearothermophilus* and grown at 58°C with vigorous aeration (220 rpm) until OD_540_ = 0.55. At this point fructose was added to 0.5%, the culture was further grown until OD_540_ = 0.9, infected with TP-84 bacteriophage at MOI = 1 and further cultivated for 30 min. 1 ml samples were taken at: just prior to infection, immediately after infection and then every 5 min, spun down and cell pellets frozen at -20°C. For MS analysis, cell pellets were suspended in 20 μl of glycine buffer for SDS-PAGE, 5 μl of 5x lysis/gel loading SDS-PAGE buffer was added, samples denatured for 10 min at 100°C and loaded onto 12.5% SDS-PAGE gels. Immediately after electrophoresis started, the loading dye entered the gel, the SDS-PAGE was stopped and the top gel fragment was excised, which contained all unresolved proteins present in the sample. Gel fragments were subjected to the MS analysis toward determination of the molecular masses of TP-84 proteins as well as for sequence coverage of the obtained peptides after trypsin digestion for precise protein identification, as described in the *TP-84 particle proteins analysis* section above. For further analysis, the detected TP-84 proteins that yielded scores of 146 or higher were selected.

### Nucleotide sequence accession number

The whole-genome sequence generated has been deposited at GenBank under the accession no. GenBank KY565347.1.

## Supporting information

S1 File TP-84 genes arrangements and sequences. Complete nucleotide sequence of TP-84 bacteriophage with marked ORFs and *cis*-regulatory regions. Putative genes, encoding proteins with assigned biological function are marked with red arrows. Genes with function confirmed by proteomic analysis are marked with orange arrows. Genes without assigned biological function are marked with black arrows. Regulatory regions, including putative promoters and terminators are shown as white boxes. The scheme was created using SnapGene software (http://www.snapgene.com) and further modified.
(PDF)

S2 File Complete nucleotide sequence of TP-84 bacteriophage. (TXT)

S3 File Taxonomic proposal of a new *Tp84virus* genus submitted to the International Committee on Taxonomy of Viruses. (PDF)

S4 File Purification and analysis of TP-84 proteins present in infected *G*. *stearothermophilus* cells lysates. (PDF)

S5 File MS results for TP-84 bacteriophage particle proteins: TP84_06, TP84_08, TP84_11, TP84_12, TP84_13, TP84_16, TP84_18, TP84_19, TP84_21, TP84_22, TP84_23, TP84_25, TP84_47 and TP84_74. (PDF)

S6 File MS results for TP-84 bacteriophage proteins present in infected *G*. *stearothermophilus* cells: TP84_03, TP84_06, TP84_10, TP84_11, TP84_12, TP84_13, TP84_17, TP84_18, TP84_19, TP84_28, TP84_36, TP84_37, TP84_38, TP84_53, TP84_54, TP84_56, TP84_57, TP84_63, TP84_64, TP84_66, TP84_68, TP84_69, TP84_79, TP84_80. (PDF)

S1 Table Putative regulatory elements in TP-84 DNA. (DOC)
