# Correction: Sequence, genome organization, annotation and proteomics of the thermophilic, 47.7-kb *Geobacillus stearothermophilus* bacteriophage TP-84 and its classification in the new *Tp84virus* genus

**DOI:** 10.1371/journal.pone.0196798

**Published:** 2018-04-26

**Authors:** Piotr M. Skowron, Andrew M. Kropinski, Joanna Zebrowska, Lukasz Janus, Kasjan Szemiako, Edyta Czajkowska, Natalia Maciejewska, Malgorzata Skowron, Joanna Łoś, Marcin Łoś, Agnieszka Zylicz-Stachula

There is an error in the Corresponding Author’s email address. The correct email address is: piotr.skowron@ug.edu.pl

There are errors in the Funding section. The correct funding information is as follows: The project has been sponsored in part by the National Center for Development (Poland) grant no POIG.01.04.00-22-140/12 issued to BioVentures Institute Ltd., in which the University of Gdansk has been a scientific partner. The BioVentures Institute Ltd. provided support in the form of salaries for the following authors: Piotr M. Skowron, Joanna Zebrowska, Lukasz Janus, Kasjan Szemiako, Edyta Czajkowska, Natalia Maciejewska, Malgorzata Skowron and Agnieszka Zylicz-Stachula. These authors are (or have been) also University of Gdansk employees or students. The specific roles of these authors are articulated in the ‘author contributions’ section. During the grant realisation, BioVentures Institute Ltd. also provided materials, reagents and equipment. BioVentures Institute Ltd. has played a predominant role in this study during all of its stages: the concept development, experiments design and realisation, as well as the decision to publish and the manuscript preparation. The project has also been sponsored in part by: University of Gdansk, Polish Ministry of Science and Higher Education fund no DS 530–8645-D691-17; National Centre for Research and Development (Poland) grant no STRATEGMED1/235077/9/NCBR/2014 and Gdansk University, Chemistry Department fund no BMN 538-8370-B817-17.
