# Supplementary material for: Sequence, genome organization, annotation and proteomics of the thermophilic, 47.7-kb Geobacillus stearothermophilus bacteriophage TP-84 and its classification in the new Tp84virus genus
Source: PLoS One. 2018 Apr 6;13(4):e0195449. doi: 10.1371/journal.pone.0195449 (PMC5889276; doi:10.1371/journal.pone.0195449)
Supplement: S1 File — Complete nucleotide sequence of TP-84 bacteriophage with marked ORFs and cis-regulatory regions. Putative genes, encoding proteins with assigned biological function are marked with red arrows. Genes with function confirmed by proteomic analysis are marked with orange arrows. Genes without assigned biological function are marked with black arrows. Regulatory regions, including putative promoters and terminators are shown as white boxes. The scheme was created using SnapGene software (http://www.snapgene.com) and further modified. (PDF) [file pone.0195449.s001.pdf]

Sequence: S1 File_061017.dna (Linear / 47 703 bp)
Features: 96 visible, 97 total

```
5' aaggaacacatgatgttcaattcgtatcaaaacgatacaacgatgtttcattcggaaggggtgaaaccaagcgtg   75
3' ttccttgtgtactacaagttaagcatagttttgctatgttgctacaaagtaagccttccccactttggttcgcac
          1   5   10   15   20
          M F N S Y Q N D T T M F H S E G V K P S V
          TP84_01 >

   gcaaagaaaaagaaagataaacatgtaccacatgcggaaaaacagcgaattgaaacgccggagcaaaaacaggcg   150
   cgtttctttttctttctatttgtacatggtgtacgcctttttgtcgcttaactttgcggcctcgtttttgtccgc
   25   30   35   40   45
   A K K K K D K H V P H A E K Q R I E T P E Q K Q A
   TP84_01 >

   ttcgaaacttatttcatgatgggtgaagatcgatcgtatcgaaaactagcaaaaatgttgaataaaggtgtgacg   225
   aagctttgaataaagtactacccacttctagctagcatagcttttgatcgtttttacaacttatttccacactgc
   50   55   60   65   70
   F E T Y F M M G E D R S Y R K L A K M L N K G V T
   TP84_01 >

   acaatctacaactgggcgcagtggtttaactggcaagaacgcatcgaagaacgtgaaaaacaagtccagaagatt   300
   tgttagatgttgacccgcgtcaccaaattgaccgttcttgcgtagcttcttgcactttttgttcaggtcttctaa
   75   80   85   90   95
   T I Y N W A Q W F N W Q E R I E E R E K Q V Q K I
   TP84_01 >

   gtcgaagaacgcaacaacaaaacgatggctgaactgaaactggaacaagccagactgatcgatgcaacactgtca   375
   cagcttcttgcgttgttgttttgctaccgacttgactttgaccttgttcggtctgactagctacgttgtgacagt
   100   105   110   115   120
   V E E R N N K T M A E L K L E Q A R L I D A T L S
   TP84_01 >

   cgattctgggaaaaagtgaaggctggaaaaatcgagatcgaaagctggcaagattatgaacgattatggcgaatt   450
   gctaagacccttttttcacttccgacctttttagctctagctttcgaccgttctaatacttgctaataccgcttaa
   125   130   135   140   145
   R F W E K V K A G K I E I E S W Q D Y E R L W R I
   TP84_01 >

   cgttgtgaaatcggcggtgaagcggatcgcaaacagtctggcgcactcactgaattgacgaacatgatcgtgaaa   525
   gcaacactttagccgccacttcgcctagcgtttgtcagaccgcgtgagtgacttaactgcttgtactagcacttt
   150   155   160   165   170
   R C E I G G E A D R K Q S G A L T E L T N M I V K
   TP84_01 >

   gtgggtgaacagatcatggccaaacaacaagaaaaggcggatgatgaagaatgacaaaatatgtgccgccatctg   600
   cacccacttgtctagtaccggtttgttgttcttttccgcctactacttcttactgttttatacacggcggtagac
                                                          1   5
                                                          M T K Y V P P S
                                                          TP84_02 >
   175   180   185
   V G E Q I M A K Q Q E K A D D E E *
   TP84_01 >

   aaaaccaagtgatggccattctgggatcaaacaaaccatttaatctatgggaaggatcgatccgaagcggcaaga   675
   ttttggttcactaccggtaagaccctagtttgtttggtaaattagatacccttcctagctaggcttcgccgttct
   10   15   20   25   30
   E N Q V M A I L G S N K P F N L W E G S I R S G K
   TP84_02 >
```

cattctggtcgatggtctggctatatcacaagatccaaacactgccgccaggggatggaatgatcctgggccaaa 750
gtaagaccagctaccagaccgatatagtgttctaggtttgtgacggcggtcccctaccttactaggacccggttt
35 40 45 50 55
T F W S M V W L Y H K I Q T L P P G D G M I L G Q
TP84_02

caccagaaacaatctatcgaaactttatgaatgaattccttttctggctggatgacgaaggtgcgaattatcgcc 825
gtggtctttgttagatagctttgaaatacttacttaaggaaaagaccgacctactgcttccacgcttaatagcgg
60 65 70 75 80
T P E T I Y R N F M N E F L F W L D D E G A N Y R
TP84_02

atgtgcaaaacagccacatcgacatcgaatatatacacaatggcacgccagagaaacgccgaatgtatatagtcg 900
tacacgttttgtcggtgtagctgtagcttatatatgtgttaccgtgcggtctctttgcggcttacatatatcagc
85 90 95 100 105
H V Q N S H I D I E Y I H N G T P E K R R M Y I V
TP84_02

gcgcaaaagacaaaggagcaatcaagcggattcgtggatcgacactgatgattgcatacattgacgaattgacga 975
cgcgttttctgtttcctcgttagttcgcctaagcacctagctgtgactactaacgtatgtaactgcttaactgct
110 115 120 125 130
G A K D K G A I K R I R G S T L M I A Y I D E L T
TP84_02

tgatgccgcaggtcgtgtttgacgaattggtgggccgcttgtcatacaaagaatcgatcctgctggccacaacga 1050
actacggcgtccagcacaaactgcttaaccacccggcgaacagtatgtttcttagctaggacgaccggtgttgct
135 140 145 150 155
M M P Q V V F D E L V G R L S Y K E S I L L A T T
TP84_02

acccggacagtccacatcactgggtgctgaaacaatatgtcgaacacccggaaaagaaacaggactggtccagat 1125
tgggcctgtcaggtgtagtgacccacgactttgttatacagcttgtgggcctttcttttgtcctgaccaggtcta
160 165 170 175 180
N P D S P H H W V L K Q Y V E H P E K K Q D W S R
TP84_02

ggcgattcacaatggatgacaacctggcactatcggaagaatacaaagatcgtgtgaagcggcaatattccggca 1200
ccgctaagtgttacctactgttggaccgtgatagccttcttatgtttctagcacacttcgccgttataaggccgt
185 190 195 200 205
W R F T M D D N L A L S E E Y K D R V K R Q Y S G
TP84_02

tcccggcacgctatcagcggatgatcctgggtcgatgggtgatggccgatggcttgatttacgatgtgttcgatc 1275
agggccgtgcgatagtcgcctactaggacccagctacccactaccggctaccgaactaaatgctacacaagctag
210 215 220 225 230
I P A R Y Q R M I L G R W V M A D G L I Y D V F D
TP84_02

cgaaaaaacacgtcatcaccagaaagcaaatggaatcgatggtcaaaggaccgccgatgaaatggcacgtgtcag 1350
gcttttttgtgcagtagtggtctttcgtttaccttagctaccagtttcctggcggctactttaccgtgcacagtc
235 240 245 250 255
P K K H V I T R K Q M E S M V K G P P M K W H V S
TP84_02

tggactatggaacgaaaaacccgacagtgttcggattgatcgggcagtggccacatccgaatccgacaagggatc 1425
acctgataccttgcttttttgggctgtcacaagcctaactagcccgtcaccggtgtaggcttaggctgttccctag
260 265 270 275 280
V D Y G T K N P T V F G L I G Q W P H P N P T R D
TP84_02

```
gaaaatatttatatatcttggtcaaggaatattattacgatggccgcaaaacaggtgtgtcaaaaacgacatccg   1500
cttttataaatatatagaaccagttccttataataatgctaccggcgttttgtccacacagtttttgctgtaggc
   285         290         295         300         305
R  K  Y  L  Y  I  L  V  K  E  Y  Y  D  G  R  K  T  G  V  S  K  T  T  S
TP84_02 >

cttatctgcaagatttagaaaatttattggtcagaagcggataagcacgatcacgatcgatccgtctgccacgc   1575
gaatagacgttctaaatcttttaaataaccagtcttcgcctattcgtgctagtgctagctaggcagacggtgcg
   310         315         320         325         330
A  Y  L  Q  D  F  R  K  F  I  G  Q  K  R  I  S  T  I  T  I  D  P  S  A  T
TP84_02 >

cgctgatcgccgaattcgaaaatgcaggattgagagtgacagaagcagacaacgaagtcctgggcggcatcgcat   1650
gcgactagcggcttaagcttttacgtcctaactctcactgtcttcgtctgttgcttcaggacccgccgtagcgta
   335         340         345         350         355
P  L  I  A  E  F  E  N  A  G  L  R  V  T  E  A  D  N  E  V  L  G  G  I  A
TP84_02 >

tggtggcgaatgcactggccgaaggatacttctatgtgctggatgaatgtgaacacaccattgaggaattcggct   1725
accaccgcttacgtgaccggcttcctatgaagatacacgacctacttacacttgtgtggtaactccttaagccga
   360         365         370         375         380
L  V  A  N  A  L  A  E  G  Y  F  Y  V  L  D  E  C  E  H  T  I  E  E  F  G
TP84_02 >

tatacatctgggatgaagcggctggcttgaaaggaatcgacaggccagtgaaggaaaacgatcactgcatggaca   1800
atatgtagaccctacttcgccgaccgaactttccttagctgtccggtcacttccttttgctagtgacgtacctgt
   385         390         395         400         405
L  Y  I  W  D  E  A  A  G  L  K  G  I  D  R  P  V  K  E  N  D  H  C  M  D
TP84_02 >

tgatcagatatttcttcaaaacacacgccgatccatcgaagcgcggcggtgttctgggcgtgtctggttggtaaa   1875
actagtctataaagaagttttgtgtgcggctaggtagcttcgcgccgccacaagacccgcacagaccaaccattt
   410         415         420         425         430
M  I  R  Y  F  F  K  T  H  A  D  P  S  K  R  G  G  V  L  G  V  S  G  W  *
TP84_02 >
                                                                   1
                                                                   M  V  K
                                                                   TP84_03 >

aaccgaacaaaaacagatgtcatcggtccaaaatattcggtgtttcaccatcaaaatgtcagctgatcgtatagg   1950
ttggcttgtttttgtctacagtagccaggttttataagccacaaagtggtagttttacagtcgactagcatatcc
      5          10          15          20          25
   T  E  Q  K  Q  M  S  S  V  Q  N  I  R  C  F  T  I  K  M  S  A  D  R  I  G
TP84_03 >

tcagcatccgaaaataccgaacaatcatcattttatccattcgaaggggggaatcattcatgggattgaaaatgtg   2025
agtcgtaggcttttatggcttgttagtagtaaaataggtaagcttccccccttagtaagtaccctaacttttacac
      30          35          40          45          50
   Q  H  P  K  I  P  N  N  H  H  F  I  H  S  K  G  E  S  F  M  G  L  K  M  W
TP84_03 >

gatcaaaaagaaactgggcctggtctatccatcggatgtgttaagggataagccaggggggtttcattgacacgac   2100
ctagtttttctttgacccggaccagataggtagcctacacaattccctattcggtcccccaaagtaactgtgctg
      55          60          65          70          75
   I  K  K  K  L  G  L  V  Y  P  S  D  V  L  R  D  K  P  G  G  F  I  D  T  T
TP84_03 >
```

agcgttcggcacgatgccagtcaatcaaatcaaaccaaaacaagggggatcaaacatggcagacacatatgcaga 2175
tcgcaagccgtgctacggtcagttagtttagtttggttttgttccccctagtttgtaccgtctgtgtatacgtct
80 85 90 95 100
A F G T M P V N Q I K P K Q G G S N M A D T Y A E
TP84_03

agcaatgataaacaaaacaaactatccagatgctgatccagaaatgacatttgaggtgcaacgatccacgcttga 2250
tcgttactatttgttttgtttgataggtctacgactaggtctttactgtaaactccacgttgctaggtgcgaact
105 110 115 120 125
A M I N K T N Y P D A D P E M T F E V Q R S T L D
TP84_03

caaactagcggaagaatacaaaaaaacaatggacaaagtggacaaaaacgaaaatgtccacgatgtccaccagga 2325
gtttgatcgccttcttatgtttttttgttacctgtttcacctgtttttgcttttacaggtgctacaggtggtcct
130 135 140 145 150
K L A E E Y K K T M D K V D K N E N V H D V H Q D
TP84_03

tgtgatcaacaaaccacggcactatcaccagggcggattcgatgcgctttatgtgatcgaaagaaaattcggtcg 2400
acactagttgtttggtgccgtgatagtggtcccgcctaagctacgcgaaatacactagctttcttttaagccagc
155 160 165 170 175
V I N K P R H Y H Q G G F D A L Y V I E R K F G R
TP84_03

tgtggtgttaaggggattctacatcggcaacatcatcaaatacatcctgcgattcgaacagaaaaacggtgtcga 2475
acaccacaattcccctaagatgtagccgttgtagtagtttatgtaggacgctaagcttgtctttttgccacagct
180 185 190 195 200
V V L R G F Y I G N I I K Y I L R F E Q K N G V E
TP84_03

agatctgaaaaaagcacgctttttatctggacaagctgatcgaacttgaagaaggaagcgcaccagatcaaagggg 2550
tctagactttttttcgtgcgaaaatagacctgttcgactagcttgaacttcttccttcgcgtggtctagtttcccc
205 210 215 220 225
D L K K A R F Y L D K L I E L E E G S A P D Q R G
TP84_03

gtgatggataaaagcggcggaaaggatggatcacggtgaaaaagtggatcatcggtgcggcaatcggcacgctga 2625
cactacctattttcgccgcctttcctacctagtgccactttttcacctagtagccacgccgttagccgtgcgact
*
TP84_03
1 5 10
M K K W I I G A A I G T L
TP84_04

tcggctacatggccggaaaattgatcagcaaactggacaacatggacaaacggatcagacagattgaagaagaaa 2700
agccgatgtaccggccttttaactagtcgtttgacctgttgtacctgtttgcctagtctgtctaacttcttcttt
15 20 25 30 35
I G Y M A G K L I S K L D N M D K R I R Q I E E E
TP84_04

tcgaaagtgatgatggatggatcgatgcagtcgaaacagatgacgacatcgccgtgaaatatccaccgaaaatgg 2775
agctttcactactacctacctagctacgtcagctttgtctactgctgtagcggcactttataggtggcttttacc
40 45 50 55 60
I E S D D G W I D A V E T D D D I A V K Y P P K M
TP84_04

cattcatctgtccaaaatgtaccgcactggtgatgtttcatggcacagacgaagcggcacagcaagagatcgaag 2850
gtaagtagacaggttttacatggcgtgaccactacaaagtaccgtgtctgcttcgccgtgtcgttctctagcttc

65 70 75 80 85
A F I C P K C T A L V M F H G T D E A A Q Q E I E
TP84_04

catggcggcagtacgaatttatttactgtccatactgtgatgaagaaatagaaatcgatgactggaccagacaca 2925
gtaccgccgtcatgcttaaataaatgacaggtatgacactacttctttatctttagctactgacctggtctgtgt

90 95 100 105 110
A W R Q Y E F I Y C P Y C D E E I E I D D W T R H
TP84_04

tcctggatcagtagaatgaaaggggaacagcatgaacgacagcaagatacaaccgattccacacatcgtggtcgc 3000
aggacctagtcatcttactttccccttgtcgtacttgctgtcgttctatgttggctaaggtgtgtagcaccagcg

115
I L D Q *
TP84_04

1 5 10 15
M N D S K I Q P I P H I V V A
TP84_05

cgaaaacggtgatggattccatgtgatcctggaagtggccatcaccgatcgtgtttttatgcgattcacggtgga 3075
gcttttgccactacctaaggtacactaggaccttcaccggtagtggctagcacaaaaatacgctaagtgccacct

20 25 30 35 40
E N G D G F H V I L E V A I T D R V F M R F T V D
TP84_05

cattgtggacaacgaggaaaaagcatatgaactggcacatgaaatccaggatcgcttgtcggtgatctggaaaga 3150
gtaacacctgttgctcctttttcgtatacttgaccgtgtactttaggtcctagcgaacagccactagacctttct

45 50 55 60 65
I V D N E E K A Y E L A H E I Q D R L S V I W K E
TP84_05

ataacgaaaggagaggggaagatatgggcatcaagtggacaaaatggtcaacaaacgtgatcaagaaataccacg 3225
tattgctttcctctccccttctatacccgtagttcacctgttttaccagttgtttgcactagttctttatggtgc

*
TP84_05

1 5 10 15
M G I K W T K W S T N V I K K Y H
TP84_06

gcaacatccaaaaatatcgcaaattgtatgacggtgatcatgccaaactgttcgaacgagcaaaacggctgatcc 3300
cgttgtaggtttttatagcgtttaacatactgccactagtacggtttgacaagcttgctcgttttgccgactagg

20 25 30 35 40
G N I Q K Y R K L Y D G D H A K L F E R A K R L I
TP84_06

aggaaggggaaatcaccgatcagatcatcgaaggtgcagaagtggccagaaacgtcaaaacaccgtacatcgtgg 3375
tccttcccctttagtggctagtctagtagcttccacgtcttcaccggtctttgcagttttgtggcatgtagcacc

45 50 55 60 65
Q E G E I T D Q I I E G A E V A R N V K T P Y I V
TP84_06

ccaatgtctgcaagatgatcgtggacatcccggccatgttggtgtccagggcaattggtcaagtgacgacatcga 3450
ggttacagacgttctactagcacctgtagggccggtacaaccacaggtcccgttaaccagttcactgctgtagct

70 75 80 85 90
A N V C K M I V D I P A M L V S R A I G Q V T T S
TP84_06

```
tgtcaccagatgattttgctggaatggtcactgatcaaggtgatggtacggtgatcagtctatacgaaaaacaaa  3525
acagtggtctactaaaacgaccttaccagtgactagttccactaccatgccactagtcagatatgctttttgttt
     95             100            105            110            115
M  S  P  D  D  F  A  G  M  V  T  D  Q  G  D  G  T  V  I  S  L  Y  E  K  Q
TP84_06 >
```

```
aagaactgatcaaagggatcgccaaacggtcgaatctgcaattcgaacacaagacaaacatcatccatcatcaaa  3600
ttcttgactagtttccctagcggtttgccagcttagacgttaagcttgtgttctgtttgtagtaggtagtagttt
     120            125            130            135            140
K  E  L  I  K  G  I  A  K  R  S  N  L  Q  F  E  H  K  T  N  I  I  H  H  Q
TP84_06 >
```

```
tggatggcggcattgtaggaatgccattcgatgatgaaaacggtctgcgaattgaattcaagtcgcgtgatgtct  3675
acctaccgccgtaacatccttacggtaagctactactttttgccagacgcttaacttaagttcagcgcactacaga
     145            150            155            160            165
M  D  G  G  I  V  G  M  P  F  D  D  E  N  G  L  R  I  E  F  K  S  R  D  V
TP84_06 >
```

```
attatccgcatccagatggccgtggatgtgatctggtgtatcaacttgaaattgaagatgaagaaacagaagaag  3750
taataggcgtaggtctaccggcacctacactagaccacatagttgaactttaacttctacttctttgtcttcttc
     170            175            180            185            190
Y  Y  P  H  P  D  G  R  G  C  D  L  V  Y  Q  L  E  I  E  D  E  E  T  E  E
TP84_06 >
```

```
cgatcaaatatttgcatgtgtaccgggaaagagtggaagaacaaaaactggtgacacaacacatgctttataaaa  3825
gctagtttataaacgtacacatggccctttctcaccttcttgtttttgaccactgtgttgtgtacgaaatatttt
     195            200            205            210            215
A  I  K  Y  L  H  V  Y  R  E  R  V  E  E  Q  K  L  V  T  Q  H  M  L  Y  K
TP84_06 >
```

```
tcggtgaatccgggatgttggaagaaatcgaggatgaagcggaagtcaaagaaatcctgggtattgaaaaaacat  3900
agccacttaggccctacaaccttctttagctcctacttcgccttcagtttctttaggacccataactttttgta
     220            225            230            235            240
I  G  E  S  G  M  L  E  E  I  E  D  E  A  E  V  K  E  I  L  G  I  E  K  T
TP84_06 >
```

```
accgggaattcgaaggccgcgacaaaccattcgtggtgtactggccaaacaataaaacattcacgcatccgcttg  3975
tggcccttaagcttccggcgctgtttggtaagcaccacatgaccggtttgttattttgtaagtgcgtaggcgaac
     245            250            255            260            265
Y  R  E  F  E  G  R  D  K  P  F  V  V  Y  W  P  N  N  K  T  F  T  H  P  L
TP84_06 >
```

```
gtcggtctgaattatacaacctggccggaaaacaggatgaaatcaactggacattgacacgaaatgcgattgttt  4050
cagccagacttaatatgttggaccggccttttgtcctactttagttgacctgtaactgtgctttacgctaacaaa
     270            275            280            285            290
G  R  S  E  L  Y  N  L  A  G  K  Q  D  E  I  N  W  T  L  T  R  N  A  I  V
TP84_06 >
```

```
atgaacgaaacggcaagccgcgaatcgccgtatcgaaagagatattccaggcgttgcaggacaaagcgtttgaac  4125
tacttgctttgccgttcggcgcttagcggcatagctttctctataaggtccgcaacgtcctgtttcgcaaacttg
     295            300            305            310            315
Y  E  R  N  G  K  P  R  I  A  V  S  K  E  I  F  Q  A  L  Q  D  K  A  F  E
TP84_06 >
```

```
gatatggcgatgaaaacaagatcgatcatcgcgatctggaaattgtcacgtttgacgaaaacggaaaagcgatgg  4200
ctataccgctacttttgttctagctagtagcgctagacctttaacagtgcaaactgcttttgccttttcgctacc
     320            325            330            335            340
R  Y  G  D  E  N  K  I  D  H  R  D  L  E  I  V  T  F  D  E  N  G  K  A  M
TP84_06 >
```

aagtcattcagatcgatgtcagcaagatcggtgacatcaaatgggtaaaagatctgatgaaactgatgctgatgg 4275
ttcagtaagtctagctacagtcgttctagccactgtagtttacccattttctagactactttgactacgactacc
345 350 355 360 365
E V I Q I D V S K I G D I K W V K D L M K L M L M
TP84_06

aaacacacacatcggaaaaagcggtcgatttttacctggaaggcaacacatcggcacagtctgggatcgcaaaat 4350
tttgtgtgtgtagcctttttcgccagctaaaaatggaccttccgttgtgtagccgtgtcagaccctagcgtttta
370 375 380 385 390
E T H T S E K A V D F Y L E G N T S A Q S G I A K
TP84_06

tttatgatctgttcgtatcgatcatgaaggcggaacaaatcgccacagaatatgtccatttcttgcaagaactat 4425
aaatactagacaagcatagctagtacttccgccttgtttagcggtgtcttatacaggtaaagaacgttcttgata
395 400 405 410 415
F Y D L F V S I M K A E Q I A T E Y V H F L Q E L
TP84_06

tcgaaaactgcctgtggatcgcacaccaggatgatccagacatcgtcatcgaagaaccacgcattcagatcaaag 4500
agcttttgacggacacctagcgtgtggtcctactaggtctgtagcagtagcttcttggtgcgtaagtctagtttc
420 425 430 435 440
F E N C L W I A H Q D D P D I V I E E P R I Q I K
TP84_06

acatgatcccgatcagccgcagagaattgatcgaacaagaaagcacggcatacaaaaacggcacacagtcgcttg 4575
tgtactagggctagtcggcgtctcttaactagcttgttctttcgtgccgtatgtttttgccgtgtgtcagcgaac
445 450 455 460 465
D M I P I S R R E L I E Q E S T A Y K N G T Q S L
TP84_06

aaacaacagtacgcaaccaaaatccgactgcgacagaagattggatcgaagatgaactggcggccatcgaggaaa 4650
tttgttgtcatgcgttggttttaggctgacgctgtcttctaacctagcttctacttgaccgccggtagctccttt
470 475 480 485 490
E T T V R N Q N P T A T E D W I E D E L A A I E E
TP84_06

gtcagcaatcgacagacacaacatcgatcctgatggggcggcagacgttatcgaatctattagacaatcgaaatc 4725
cagtcgttagctgtctgtgttgtagctaggactaccccgccgtctgcaatagcttagataatctgttagctttag
495 500 505 510 515
S Q Q S T D T T S I L M G R Q T L S N L L D N R N
TP84_06

ctaacggcacgccgatcggagcggcacagcaacagccacaacaaggcacaccgcaaacagggggtggccaggcgt 4800
gattgccgtgcggctagcctcgccgtgtcgttgtcggtgttgttccgtgtggcgtttgtcccccaccggtccgca
520 525 530 535 540
P N G T P I G A A Q Q Q P Q Q G T P Q T G G G Q A
TP84_06

1
M
TP84_07

gatgatgaataaaaatggacaagttggacaaaatgaacgcagtgtgatcgtggacaaaacggacaaacgagaaac 4875
ctactacttatttttacctgttcaacctgttttacttgcgtcacactagcacctgttttgcctgtttgctctttg

*
TP84_06

5 10 15 20 25
M M N K N G Q V G Q N E R S V I V D K T D K R E T
TP84_07

ggcaaatctggtcatatcttattgtctgatcatcgccgggatcatcatcacgattgtcactcatttatgaaaagg 4950
ccgtttagaccagtatagaataacagactagtagcggccctagtagtagtgctaacagtgagtaaatacttttcc

30 35 40 45
A N L V I S Y C L I I A G I I I T I V T H L *
TP84_07

ggtgatcctggatggccaacatggaccagatcgaacgacaaattgtggaactatgcaggcaagctattatcgaca 5025
ccactaggacctaccggttgtacctggtctagcttgctgtttaacaccttgatacgtccgttcgataatagctgt

1 5 10 15 20
M A N M D Q I E R Q I V E L C R Q A I I D
TP84_08

ttatcgcatcggtcggtggtgtcgataacttactggatgatgaacggcgaatggcactggtcaacgccatcgccg 5100
aatagcgtagccagccaccacagctattgaatgacctactacttgccgcttaccgtgaccagttgcggtagcggc

25 30 35 40 45
I I A S V G G V D N L L D D E R R M A L V N A I A
TP84_08

cacgaatgcaacaactgggcgcagacatttcggccatcctgcctgccgaacttgcaaaagcctattatcaaggca 5175
gtgcttacgttgttgacccgcgtctgtaaagccggtaggacggacggcttgaacgttttcggataatagttccgt

50 55 60 65 70
A R M Q Q L G A D I S A I L P A E L A K A Y Y Q G
TP84_08

taaccgatgccgatgcattcttgcatcaagaaggcacggaagtcacggtccagggtgtgaataaacaaatccacc 5250
attggctacggctacgtaagaacgtagttcttccgtgccttcagtgccaggtcccacacttatttgtttaggtgg

75 80 85 90 95
I T D A D A F L H Q E G T E V T V Q G V N K Q I H
TP84_08

tggccgggatcgaatcgatcgtgtctgacacaatgatggatatgcaagcggcaatccgaaccgcagtggcgatgg 5325
accggccctagcttagctagcacagactgtgttactacctatacgttcgccgttaggcttggcgtcaccgctacc

100 105 110 115 120
L A G I E S I V S D T M M D M Q A A I R T A V A M
TP84_08

ccatcgccgacatcgatcgcatcctggtcgatgtacggaacgacatcgcaaaaggaatgatcctgggcgatccat 5400
ggtagcggctgtagctagcgtaggaccagctacatgccttgctgtagcgttttccttactaggacccgctaggta

125 130 135 140 145
A I A D I D R I L V D V R N D I A K G M I L G D P
TP84_08

cacgtgaaatcacaaaacgtgtcatgcagacatttgccaacagcggcatgacagcattcatcacgaaagataata 5475
gtgcactttagtgttttgcacagtacgtctgtaaacggttgtcgccgtactgtcgtaagtagtgctttctattat

150 155 160 165 170
S R E I T K R V M Q T F A N S G M T A F I T K D N
TP84_08

agcgactgccgcttgattttatgcgatgactgtcaccagaacaaaaatgcgccaggcacacacagacggtgcag 5550
tcgctgacggcgaactaaaaatacgctactgacagtggtcttgtttttacgcggtccgtgtgtgtctgccacgtc
175 180 185 190 195
K R L P L D F Y A M T V T R T K M R Q A H T D G A
TP84_08

tcaatcgatacaaagaaaatggcgtttatcacgtcaagatcagcgaacacggtattacatgcaacaaatgtgcaa 5625
agttagctatgtttcttttaccgcaaatagtgcagttctagtcgcttgtgccataatgtacgttgtttacacgtt
200 205 210 215 220
V N R Y K E N G V Y H V K I S E H G I T C N K C A
TP84_08

gataccagggattagtcgtggcacttgatcccgaacatgcggaaggctttccagtggccgggatcgatgtgccac 5700
ctatggtccctaatcagcaccgtgaactagggcttgtacgccttccgaaaggtcaccggccctagctacacggtg
225 230 235 240 245
R Y Q G L V V A L D P E H A E G F P V A G I D V P
TP84_08

tgccgccatatcatccaaactgccgccatacagtgcggccattcgtgatggaatatcacagtcaaaacgacatcc 5775
acggcggtatagtaggtttgacggcggtatgtcacgccggtaagcactaccttatagtgtcagttttgctgtagg
250 255 260 265 270
L P P Y H P N C R H T V R P F V M E Y H S Q N D I
TP84_08

gaaacgaaaagcggaagtggaaatcgttcgatccagaaggtgatccgcggaccgatgcacaaaaacgactataca 5850
ctttgcttttcgccttcacctttagcaagctaggtcttccactaggcgcctggctacgtgtttttgctgatatgt
275 280 285 290 295
R N E K R K W K S F D P E G D P R T D A Q K R L Y
TP84_08

aggccgaacaagacattcgccgcaaagcgcggcaggaaatgaaagaatacatgctgatgaaagccacactgccgc 5925
tccggcttgttctgtaagcggcgtttcgcgccgtcctttactttcttatgtacgactactttcggtgtgacggcg
300 305 310 315 320
K A E Q D I R R K A R Q E M K E Y M L M K A T L P
TP84_08

cggatcaagtgccgaaaacactggccgcttatcgccggatgaagcggaagaacgatgcacagtggcaaaaattac 6000
gcctagttcacggcttttgtgaccggcgaatagcggcctacttcgccttcttgctacgtgtcaccgtttttaatg
325 330 335 340 345
P D Q V P K T L A A Y R R M K R K N D A Q W Q K L
TP84_08

aggcacaattcaaacaagcactgggcgaagtggatctgcaagggccgccgccaggaacgccacgaaagcggaaag 6075
tccgtgttaagtttgttcgtgacccgcttcacctagacgttcccggcggcggtccttgcggtgctttcgcctttc
350 355 360 365 370
Q A Q F K Q A L G E V D L Q G P P P G T P R K R K
TP84_08

gggcaaaaactggtcctggatcatcgacaccgaaaacggacaaaacggacaaaaaacaaaacgtgtccactttgt 6150
cccgtttttgaccaggacctagtagctgtggcttttgcctgttttgcctgttttttgttttgcacaggtgaaaca
375 380 385 390 395
G A K T G P G S S T P K T D K T D K K Q N V S T L
TP84_08

ccacactgccaaaaggaccgaaactgacggaagcggatctggcggcacacaaagagtttgacaaacgcatcgatg 6225
ggtgtgacggttttcctggctttgactgccttcgcctagaccgccgtgtgtttctcaaactgtttgcgtagctac
400 405 410 415 420
S T L P K G P K L T E A D L A A H K E F D K R I D
TP84_08

gcatcgaattcgctgatcgcagacagacagcaagattcttacttgatcagatccctggcggcaaccagatcaaag 6300
cgtagcttaagcgactagcgtctgtctgtcgttctaagaatgaactagtctagggaccgccgttggtctagtttc
425 430 435 440 445
G I E F A D R R Q T A R F L L D Q I P G G N Q I K
TP84_08

tcagcatccgaaaaattgaagcgaatggccacgtgggaaaattcagcgtggttggcggaaaattcataatcggtg 6375
agtcgtaggctttttaacttcgcttaccggtgcacccttttaagtcgcaccaaccgccttttaagtattagccac
450 455 460 465 470
V S I R K I E A N G H V G K F S V V G G K F I I G
TP84_08

aatatgcattgttgaaagatgatccgcggccgccgcagtatcgatggaaaacggtctttcatgaatactaccatg 6450
ttatacgtaacaactttctactaggcgccggcggcgtcatagctaccttttgccagaaagtacttatgatggtac
475 480 485 490 495
E Y A L L K D D P R P P Q Y R W K T V F H E Y Y H
TP84_08

cacagatgcatggcatggattatgacgatcattttcagcgtgataaccaggaatggactatctgggaagaaacag 6525
gtgtctacgtaccgtacctaatactgctagtaaaagtcgcactattggtccttacctgatagacccttctttgtc
500 505 510 515 520
A Q M H G M D Y D D H F Q R D N Q E W T I W E E T
TP84_08

ccaccgaatgtgcggcattcttcatggcgcaaagggccggatggaatgtgtccacgatcctaccgtcttattcag 6600
ggtggcttacacgccgtaagaagtaccgcgtttcccggcctaccttacacaggtgctaggatggcagaataagtc
525 530 535 540 545
A T E C A A F F M A Q R A G W N V S T I L P S Y S
TP84_08

aatatctgatccgcacactgccgatgctgaaacaactgccagaatacgccgattgtgaaacgatggtcgattttg 6675
ttatagactaggcgtgtgacggctacgactttgttgacggtcttatgcggctaacactttgctaccagctaaaac
550 555 560 565 570
E Y L I R T L P M L K Q L P E Y A D C E T M V D F
TP84_08

gtgcaaagtttatgaagtatcgatttaatgaagaacataaaacgatggaatggcggtggctagtcagacgtgtgg 6750
cacgtttcaaatacttcatagctaaattacttcttgtattttgctaccttaccgccaccgatcagtctgcacacc
575 580 585 590 595
G A K F M K Y R F N E E H K T M E W R W L V R R V
TP84_08

aacaaaatgcagatgtgttcgacatcaacgaatatttccgggaacactacctggaacatatcaaagcgaacaaag 6825
ttgttttacgtctacacaagctgtagttgcttataaaggcccttgtgatggaccttgtatagtttcgcttgtttc
600 605 610 615 620
E Q N A D V F D I N E Y F R E H Y L E H I K A N K
TP84_08

accgatatgccgatatgatttacgattcactgtcacagtcgaacaaatttacacacgaatccgtgatccggcaga 6900
tggctatacggctatactaaatgctaagtgacagtgtcagcttgtttaaatgtgtgcttaggcactaggccgtct
625 630 635 640 645
D R Y A D M I Y D S L S Q S N K F T H E S V I R Q
TP84_08

tgatcaaagaaggcctggaatctgggatcagattcggcacggtgaaccgggaaattcgcttatgtctgccagtcg 6975
actagtttcttccggaccttagaccctagtctaagccgtgccacttggccctttaagcgaatacagacggtcagc
650 655 660 665 670
M I K E G L E S G I R F G T V N R E I R L C L P V
TP84_08

cgatgaatgaactgggggtgaagaagccatgatcgcgatctatgatctgatcggaaaaattcaaaacatggacaa 7050
gctacttacttgacccccacttcttcggtactagcgctagatactagactagcctttttaagttttgtacctgtt

675 680
A M N E L G V K K P *
TP84_08

1 5 10 15
M I A I Y D L I G K I Q N M D N
TP84_09

catggacaaactggatgaaatcatcagtcgatggtactttcaaacatccgtatggacaccacaagtgaaagaaga 7125
gtacctgtttgacctactttagtagtcagctaccatgaaagtttgtaggcatacctgtggtgttcactttcttct

20 25 30 35 40
M D K L D E I I S R W Y F Q T S V W T P Q V K E E
TP84_09

agtcaaaaaagcggcggtcgaaatcctgcaactaggcgaaccgaaagtgtaccagtttattgtggatatgtacgg 7200
tcagttttttcgccgccagctttaggacgttgatccgcttggctttcacatggtcaaataacacctatacatgcc

45 50 55 60 65
V K K A A V E I L Q L G E P K V Y Q F I V D M Y G
TP84_09

ccttaaaatgtgaacaatgtggacaactgaatggaaaaatattcacttttttagccgaatgtttggtataataacg 7275
ggaattttacacttgttacacctgttgacttaccttttataagtgaaaaatcggcttacaaaccatattattgc

70
L K M *
TP84_09

putative host-dependent promoter

ataaagacaagcaatcgggccacaaaccgtgtgagtggcccggcctgggatcattggattgtctgatcctgggtg 7350
tatttctgttcgttagcccggtgtttggcacactcaccgggccggaccctagtaacctaacagactaggacccac

gtttcatcacgtgaacgtgtttttttcggcctttttcctggtgacgggccttaaacgaaaccagagatcgtgcaag 7425
caaagtagtgcacttgcacaaaaaaagccggaaaaggaccactgcccggaatttgctttggtctctagcacgttc

ggggaagaaagtaaaatgcaagacaaacaaatcacggatgaattcatccaaaaagcgaataaagcatccgaattc 7500
ccccttctttcattttacgttctgtttgtttagtgcctacttaagtaggtttttcgcttatttcgtaggcttaag

1 5 10 15 20
M Q D K Q I T D E F I Q K A N K A S E F
TP84_10

ggattcctgcaatttttgccggagagggtggacagcaaggcggcgaacctactgatccaaccgatccaactgat 7575
cctaaggacgttaaaaacggcctctcccacctgtcgttccgccgcttggatgactaggttggctaggttgacta

25 30 35 40 45
G F L Q F F A G E G G Q Q G G E P T D P T D P T D
TP84_10

ccgcttgacgatccagaaggcggcgaaccaaacgatccgactgatccaactgatccagtcgatgatcaagatgat 7650
ggcgaactgctaggtcttccgccgcttggtttgctaggctgactaggttgactaggtcagctactagttctacta

50 55 60 65 70
P L D D P E G G E P N D P T D P T D P V D D Q D D
TP84_10

aacgatgacgatactgggatcacatatgaaaaaggcttgattgatcgcattttaaaagcaaataacatcgatttt 7725
ttgctactgctatgaccctagtgtatactttttccgaactaactagcgtaaaattttcgtttattgtagctaaaa

75 80 85 90 95
N D D D T G I T Y E K G L I D R I L K A N N I D F
TP84_10

ggtcaattgttgaaggacaatccagaactgaaacggcaatatcaagcaagattcaacaaaaatatgtccaaacgc 7800
ccagttaacaacttcctgttaggtcttgactttgccgttatagttcgttctaagttgtttttatacaggtttgcg
100 105 110 115 120
G Q L L K D N P E L K R Q Y Q A R F N K N M S K R
TP84_10

cttgaaaaataccagggtgtcgatgtcgatgaatattttgaactgaaaaaacgtgcagaaagcggcaatctcgaa 7875
gaactttttatggtcccacagctacagctacttataaaacttgacttttttgcacgtctttcgccgttagagctt
125 130 135 140 145
L E K Y Q G V D V D E Y F E L K K R A E S G N L E
TP84_10

ggtgacgcaaagacttggaaagaaaaatacgaacaattgaaagcagaaatggaaacaacaacaaagaaaacagcc 7950
ccactgcgtttctgaacctttcttttatgcttgttaactttcgtctttaccttgttgttgtttcttttgtcgg
150 155 160 165 170
G D A K T W K E K Y E Q L K A E M E T T T K K T A
TP84_10

attcaacagtatgcgatcgaaaacggattcgattccgaacagatcgcattcatcacgtcaatgatcgacatgaac 8025
taagttgtcatacgctagcttttgcctaagctaaggcttgtctagcgtaagtagtgcagttactagctgtacttg
175 180 185 190 195
I Q Q Y A I E N G F D S E Q I A F I T S M I D M N
TP84_10

aagctggaacgtgatgacgaaggtgaatggatgggcattgacgaggaagtcgaacgaatcaaagaaaaatttcca 8100
ttcgaccttgcactactgcttccacttacctacccgtaactgctccttcagcttgcttagtttctttttaaaggt
200 205 210 215 220
K L E R D D E G E W M G I D E E V E R I K E K F P
TP84_10

cggatgttcgaaccacgtgacggtcaagtcggcggcacaccgaaaaaagaatcgaaatataatcctggcacgaaa 8175
gcctacaagcttggtgcactgccagttcagccgccgtgtggctttttttcttagctttatattaggaccgtgcttt
225 230 235 240 245
R M F E P R D G Q V G G T P K K E S K Y N P G T K
TP84_10

aagcacaatcaagacacgaaaccgactgatccacgagaactgggccgacaaatcgcattacagcggcataaaaac 8250
ttcgtgttagttctgtgctttggctgactaggtgctcttgacccggctgtttagcgtaatgtcgccgtatttttg
250 255 260 265 270
K H N Q D T K P T D P R E L G R Q I A L Q R H K N
TP84_10

cgcttgaaacaacaataaggaggtaaaaacacatgaacttgcaaccgaaaattgtatcgatcgcaggccagaaag 8325
gcgaactttgttgttattcctccatttttgtgtacttgaacgttggcttttaacatagctagcgtccggtctttc
275 1 5 10
R L K Q Q * M N L Q P K I V S I A G Q K
TP84_10 TP84_11

aattcctggccactacacaaggccttgtccacaaagtgggtggcgtgacgcttgacgcttcaaaattcacgccag 8400
ttaaggaccggtgatgtgttccggaacaggtgtttcacccaccgcactgcgaactgcgaagttttaagtgcggtc
15 20 25 30 35
E F L A T T Q G L V H K V G G V T L D A S K F T P
TP84_11

atgaaaatggattcatcaaggccggatctgcacttgcattgacagcaagcggaaaagctgaaccattcaacgttt 8475
tactttacctaagtagttccggcctagacgtgaacgtaactgtcgttcgccttttcgacttggtaagttgcaaa
40 45 50 55 60
D E N G F I K A G S A L A L T A S G K A E P F N V
TP84_11

```
caacacctggcgatccatccactgcaaacggaacgccatacattcttgcacacgatgtgcaaatcaaagacggca    8550
gttgtggaccgctaggtaggtgacgtttgccttgcggtatgtaagaacgtgtgctacacgtttagtttctgccgt
65        70        75        80        85
S T P G D P S T A N G T P Y I L A H D V Q I K D G
TP84_11

caaccaacatcgatgctgtcgccggagtgttagaagccgcttatttaaaatcgtcagtggtcacaaccgctgaac    8625
gttggttgtagctacgacagcggcctcacaatcttcggcgaataaattttagcagtcaccagtgttggcgacttg
90        95        100       105       110
T T N I D A V A G V L E A A Y L K S S V V T T A E
TP84_11

ctggtcgtgtcgtggtgacacaagattttatcgatgcatcgaacggtcgattccaccttcgataatggtggacaa    8700
gaccagcacagcaccactgtgttctaaaatagctacgtagcttgccagctaaggtggaagctattaccacctgtt
115       120       125       130       135
P G R V V V T Q D F I D A S N G R F H L R *
TP84_11

gccggacaatattcatttaaggaggtaaaaaacgatgcctttacatttagaacaattccaacgtgaagcattcc    8775
cggcctgttataagtaaaattcctccattttttgctacggaaatgtaaatcttgttaaggttgcacttcgtaagg
                                    1         5         10
                                    M P L H L E Q F Q R E A F
                                    TP84_12

aggggtacgttgaaaacgtgccgccaaaacgtgaatatgcactggcaaaattcatgccgaatcagccagtctatg    8850
tccccatgcaacttttgcacggcggttttgcacttatacgtgaccgttttaagtacggcttagtcggtcagatac
  15        20        25        30        35
Q G Y V E N V P P K R E Y A L A K F M P N Q P V Y
TP84_12

acattgaattcacatacaacatcatcaatggcggatatggacaaatggcatccatcaccgcatgggattctggcg    8925
tgtaacttaagtgtatgttgtagtagttaccgcctatacctgtttaccgtaggtagtggcgtaccctaagaccgc
  40        45        50        55        60
D I E F T Y N I I N G G Y G Q M A S I T A W D S G
TP84_12

cgccgttgcgtgacaaagatgtgattcaacgcttgacagcgcaaatcgccaaagtgcaacacgcatatcgcctta    9000
gcggcaacgcactgtttctacactaagttgcgaactgtcgcgtttagcggtttcacgttgtgcgtatagcggaat
  65        70        75        80        85
A P L R D K D V I Q R L T A Q I A K V Q H A Y R L
TP84_12

ctgaaaaagaactgttaatgtttcaccggccacgtatggatgaagaacagcaacaagtcattcaagcgatctaca    9075
gactttttcttgacaattacaaagtggccggtgcatacctacttcttgtcgttgttcagtaagttcgctagatgt
  90        95        100       105       110
T E K E L L M F H R P R M D E E Q Q Q V I Q A I Y
TP84_12

acaacacagacaagcttgtctggggtgttcaagaccgtgaagaatggttgcgcgctaaagctgtttatgtcggac    9150
tgttgtgtctgttcgaacagaccccacaagttctggcacttcttaccaacgcgcgatttcgacaaatacagcctg
  115       120       125       130       135
N N T D K L V W G V Q D R E E W L R A K A V Y V G
TP84_12

aattacaatacagcgaaaacgatgtgcaattgaacatcgacttcttgattccggcagaaaacaaattgactgccg    9225
ttaatgttatgtcgcttttgctacacgttaacttgtagctgaagaactaaggccgtcttttgtttaactgacggc
  140       145       150       155       160
Q L Q Y S E N D V Q L N I D F L I P A E N K L T A
TP84_12
```

```
atgtggactggtctgatccgactgcgccagtgatccaacacttacaaagcgcagtgcaacgattcaaagaagcaa   9300
tacacctgaccagactaggctgacgcggtcactaggttgtgaatgtttcgcgtcacgttgctaagtttcttcgtt
  165      170      175      180      185
D V D W S D P T A P V I Q H L Q S A V Q R F K E A
TP84_12

acaacggcgaaaagccagtcgaaatgcatatgtctagccgtgtagaaacatggttattacaaaacgagcaagtga   9375
tgttgccgcttttcggtcagctttacgtatacagatcggcacatctttgtaccaataatgttttgctcgttcact
  190      195      200      205      210
N N G E K P V E M H M S S R V E T W L L Q N E Q V
TP84_12

aagcacacatctatggaaacacgactgatccgcgcatcgtgacaagcgaacaattacaacaattattcagcgcgt   9450
ttcgtgtgtagatacctttgtgctgactaggcgcgtagcactgttcgcttgttaatgttgttaataagtcgcgca
  215      220      225      230      235
K A H I Y G N T T D P R I V T S E Q L Q Q L F S A
TP84_12

tgtcgttgccgccatatcgtgtgatcgatgaacaagtgattggcgaaaacggcgcggaagcactcatgccagaag   9525
acagcaacggcggtatagcacactagctacttgttcactaaccgcttttgccgcgccttcgtgagtacggtcttc
  240      245      250      255      260
L S L P P Y R V I D E Q V I G E N G A E A L M P E
TP84_12

atcgtgtcgtcttacttggtgaagaacttggacacacaatggaaggaccgacagtcgaaaacaactacaagcctg   9600
tagcacagcagaatgaaccacttcttgaacctgtgtgttaccttcctggctgtcagcttttgttgatgttcggac
  265      270      275      280      285
D R V V L L G E E L G H T M E G P T V E N N Y K P
TP84_12

gcatttatgtcattccagaaatcaaagaaacaaatccaccacgccaggaagtatatgtcggaaaatctgtatttc   9675
cgtaaatacagtaaggtctttagtttctttgtttaggtggtgcggtccttcatatacagccttttagacataaag
  290      295      300      305      310
G I Y V I P E I K E T N P P R Q E V Y V G K S V F
TP84_12

cggcattagaacggccacaagcagtcgttcatttaatcgttgcacaatcctaatgacaaaaaagggatagacaca   9750
gccgtaatcttgccggtgttcgtcagcaagtaaattagcaacgtgttaggattactgttttttccctatctgtgt
  315      320      325      330
P A L E R P Q A V V H L I V A Q S *
TP84_12
rho-independent terminator

tgatctagtctatcccttttttaaaaaatacattcaaagggggattttttcatgccgaaatacatcgcaaaacgt   9825
actagatcagatagggaaaaaattttttatgtaagtttccccctaaaaaagtacggctttatgtagcgttttgca
                                              1       5
                                              M P K Y I A K R
rho-independent terminator                    TP84_13

catttagtgacacgcactggaatcaaaaaacctggtgatgtgatcgaatacacgaaagaacaagcgcaaaaatta   9900
gtaaatcactgtgcgtgaccttagttttttggaccactacactagcttatgtgctttcttgttcgcgtttttaat
  10       15       20       25       30
H L V T R T G I K K P G D V I E Y T K E Q A Q K L
TP84_13
```

ttggccgcaggattcattgaagaagcggaagaagaaaagaaaacaacaagcaaaggaaaacaaaaagctgatgaa 9975
aaccggcgtcctaagtaacttcttcgccttcttcttttcttttgttgttcgtttccttttgtttttcgactactt

35 40 45 50 55
L A A G F I E E A E E E K K T T S K G K Q K A D E
TP84_13

gcggacaaagtggacaaataaaaatgtccactatgtccacataagaggtgaaggccatgacagtcaaagaatatt 10 050
cgcctgtttcacctgtttatttttacaggtgatacaggtgtattctccacttccggtactgtcagtttcttataa

60 65
A D K V D K *
TP84_13

1 5
M T V K E Y
TP84_14

tggccaaaattccaggtgcagatgaattcctggcactgccggaagcagaacaagaaaaatggatctttatggccg 10 125
accggttttaaggtccacgtctacttaaggaccgtgacggccttcgtcttgttctttttacctagaaataccggc

10 15 20 25 30
L A K I P G A D E F L A L P E A E Q E K W I F M A
TP84_14

aagaaacactgcgtgatcattttcatccgcgtgtgatcacgccgcgtgtggttgcactgcaagtcatgtatatgt 10 200
ttctttgtgacgcactagtaaaagtaggcgcacactagtgcggcgcacaccaacgtgacgttcagtacatataca

35 40 45 50 55
E E T L R D H F H P R V I T P R V V A L Q V M Y M
TP84_14

tcgaaggcgaatccgaagatttgccatgctaaaacgacagggcgtggattcgttcagcacagaaggactgtctg 10 275
agcttccgcttaggcttctaaacggtacgattttgctgtcccgcacctaagcaagtcgtgtcttcctgacagac

60 65 70 75 80
F E G E S E D F A M L K R Q G V D S F S T E G L S
TP84_14

tttcattcaaatctggatcaatcgcgccgatggtcctggaaatcgttaatcgaagcggatcggcaaaggtgggcc 10 350
aaagtaagtttagacctagttagcgcggctaccaggacctttagcaattagcttcgcctagccgtttccacccgg

85 90 95 100 105
V S F K S G S I A P M V L E I V N R S G S A K V G
TP84_14

gtttgatatgaaaccaccgatgaatgatccagtcgtgctgaaacagcccaaaaaagtcgatggcaagatccaaaa 10 425
caaactatactttggtggctacttactaggtcagcacgactttgtcgggttttttcagctaccgttctaggtttt

110
R L I *
TP84_14

1 5 10 15 20
M K P P M N D P V V L K Q P K K V D G K I Q K
TP84_15

agacgaaaagaggattcgccgaaatgatcgaaatcccggtcatggccagggtgatcaacgaaaccaccgaagtgat 10 500
tctgcttttctcctaagcggctttactagctttagggccagtaccggtcccactagttgctttggtggcttcacta

25 30 35 40 45
D E R G F A E M I E I P V M A R V I N E T T E V I
TP84_15

cgatgagaaaggacaaacacgtcaagctgtcatgactgtcatcctcccaccatctgtgacaccatccctgggcga 10 575
gctactctttcctgtttgtgcagttcgacagtactgacagtaggagggtggtagacactgtggtagggacccgct

50 55 60 65 70
D E K G Q T R Q A V M T V I L P P S V T P S L G D
TP84_15

```
tgaaatacaagtcggatcagacaccgtggtgatcctacaagtcgaaccacgaaagtcctggtctggcaaaaagac   10 650
actttatgttcagcctagtctgtggcaccactaggatgttcagcttggtgctttcaggaccagaccgtttttctg
   75        80        85        90        95
 E  I  Q  V  G  S  D  T  V  V  I  L  Q  V  E  P  R  K  S  W  S  G  K  K  T
TP84_15 >

atattactgggtgacgaactgtggcacgtgatcacatcttttcattcgagtgggaaggtattgacgaactggtcg   10 725
tataatgacccactgcttgacaccgtgcactagtgtagaaaagtaagctcacccttccataactgcttgaccagc
                        1       5        10        15
                        M  A  R  D  H  I  F  S  F  E  W  E  G  I  D  E  L  V
                        TP84_16 >
  100       105
 Y  Y  W  V  T  N  C  G  T  *
TP84_15 >

agttattggaaaacatggacagaaaaacaagacggataatggtccaggaatacacaaaattcggcctattggttg   10 800
tcaataaccttttgtacctgtctttttgttctgcctattaccaggtccttatgtgttttaagccggataaccaac
   20        25        30        35        40
E  L  L  E  N  M  D  R  K  T  R  R  I  M  V  Q  E  Y  T  K  F  G  L  L  V
TP84_16 >

aagaaggtgcgagggcacttgcaccgaaagacgaaggcaacctggaagattcgatcaacgcagaaaaagcgaaaa   10 875
ttcttccacgctcccgtgaacgtggctttctgcttccgttggaccttctaagctagttgcgtctttttcgctttt
   45        50        55        60        65
E  E  G  A  R  A  L  A  P  K  D  E  G  N  L  E  D  S  I  N  A  E  K  A  K
TP84_16 >

tcatcggtgaaggcgtggaagtggaagtcggtgtcggatcggtctatggactgcggcgacacgaagaaccaccac   10 950
agtagccacttccgcaccttcaccttcagccacagcctagccagatacctgacgccgctgtgcttcttggtggtg
   70        75        80        85        90
I  I  G  E  G  V  E  V  E  V  G  V  G  S  V  Y  G  L  R  R  H  E  E  P  P
TP84_16 >

gcatcggaaaatacccgaaatatgagcgcggcgcaaaattcccgaactactatgtgaacggtctgggtgcgcgga   11 025
cgtagccttttatgggctttatactcgcgccgcgttttaagggcttgatgatacacttgccagacccacgcgcct
   95        100       105       110       115
R  I  G  K  Y  P  K  Y  E  R  G  A  K  F  P  N  Y  Y  V  N  G  L  G  A  R
TP84_16 >

ccagatcaaaaccaggatggcgcggcgaaaagccaggtcgaaaatatctgcaaagggccgtggaattggttgtgg   11 100
ggtctagttttggtcctaccgcgccgcttttcggtccagcttttatagacgtttcccggcaccttaaccaacacc
   120       125       130       135       140
T  R  S  K  P  G  W  R  G  E  K  P  G  R  K  Y  L  Q  R  A  V  E  L  V  V
TP84_16 >

atgattttgatcaaatgaacgagcgcatccttgaacggatcatggaaggcaggcgataaacatgatccagaagtg   11 175
tactaaaactagtttacttgctcgcgtaggaacttgcctagtaccttccgtccgctatttgtactaggtcttcac
   145       150       155       160                  1        5
D  D  F  D  Q  M  N  E  R  I  L  E  R  I  M  E  G  R  R  *    M  I  Q  K  W
TP84_16 >                                                      TP84_17 >

gatcaaagatcaattggaagcggcgatcccgaacctggaatggacatatgactacaaaacaggcaaggatcacac   11 250
ctagtttctagttaaccttcgccgctagggcttggaccttacctgtatactgatgttttgtccgttcctagtgtg
            10        15        20        25       30
 I  K  D  Q  L  E  A  A  I  P  N  L  E  W  T  Y  D  Y  K  T  G  K  D  H  T
TP84_17 >
```

tggcgtggtctatcacgaaacgccagggcagatcagccgggatgattttgagatcatcacaccgtcatattcggt 11 325
accgcaccagatagtgctttgcggtcccgtctagtcggccctactaaaactctagtagtgtggcagtataagcca
35 40 45 50 55
G V V Y H E T P G Q I S R D D F E I I T P S Y S V
TP84_17

gtatatcgaaacatccgacatgaaaaacgccgaaaagtgggcctggatcgtttatgacactatgaacaagcggcg 11 400
catatagctttgtaggctgtacttttgcggcttttcacccggacctagcaaatactgtgatacttgttcgccgc
60 65 70 75 80
Y I E T S D M K N A E K W A W I V Y D T M N K R R
TP84_17

gcaggaagtggccaccatcgatgatcggtcattccaggtcatatttatcgaagtcacgacaccaccgatcctggt 11 475
cgtccttcaccggtggtagctactagccagtaaggtccagtataaatagcttcagtgctgtggtggctaggacca
85 90 95 100 105
Q E V A T I D D R S F Q V I F I E V T T P P I L V
TP84_17

ggggatcgttgacaaaaaaatgacatactcaatcaatctacaagcgacaatccgcaaaatataaggaggtagaaa 11 550
cccctagcaactgtttttttactgtatgagttagttagatgttcgctgttaggcgttttatattcctccatcttt
110 115 120 125
G I V D K K M T Y S I N L Q A T I R K I *
TP84_17

ggtatgccaggggtaaacattccattcggtcttgcaacgatcacagtgggtgatccacaagatccgaataaaatc 11 625
ccatacggtccccatttgtaaggtaagccagaacgttgctagtgtcacccactaggtgttctaggcttattttag
1 5 10 15 20
M P G V N I P F G L A T I T V G D P Q D P N K I
TP84_18

gtgttcgatggcgtgaattattttcaagcagaaggcggcgaacttaacattgagccggaacttgaaccgatcgaa 11 700
cacaagctaccgcacttaataaaagttcgtcttccgccgcttgaattgtaactcggccttgaacttggctagctt
25 30 35 40 45
V F D G V N Y F Q A E G G E L N I E P E L E P I E
TP84_18

ctgcaagattttggtgcgacaccgtatgatgaacgcattaatggatatacatgtgaattgaccatcgttgttggt 11 775
gacgttctaaaaccacgctgtggcatactacttgcgtaattacctatatgtacacttaactggtagcaacaacca
50 55 60 65 70
L Q D F G A T P Y D E R I N G Y T C E L T I V V G
TP84_18

caaaacgacttgaaactgatgcgtaaactattcgcatatcattcggaaatcgtggatgaaggaacaggcgaagtc 11 850
gttttgctgaactttgactacgcatttgataagcgtatagtaagcctttagcacctacttccttgtccgcttcag
75 80 85 90 95
Q N D L K L M R K L F A Y H S E I V D E G T G E V
TP84_18

atcggcctaactgacgaaaagatcggcgcatcgatgcgtgacaaagcagtgccagtcacgatccatccacgtgaa 11 925
tagccggattgactgcttttctagccgcgtagctacgcactgtttcgtcacggtcagtgctaggtaggtgcactt
100 105 110 115 120
I G L T D E K I G A S M R D K A V P V T I H P R E
TP84_18

atgggatcagacacaagtctggacatccacatttataaaatgtctggtgttggtgcgttcaaccgcacatatgaa 12 000
taccctagtctgtgttcagacctgtaggtgtaaatattttacagaccacaaccacgcaagttggcgtgtatactt
125 130 135 140 145
M G S D T S L D I H I Y K M S G V G A F N R T Y E
TP84_18

```
aacgcacagggatcatatgaagtcacactgcgtgcatatccacgtgatggcgcagatccaacgaaaccaggcaat   12 075
ttgcgtgtccctagtatacttcagtgtgacgcacgtataggtgcactaccgcgtctaggttgctttggtccgtta
150       155       160       165       170
N A Q G S Y E V T L R A Y P R D G A D P T K P G N
TP84_18 >

ttctattacatcggcgacactgatccaaacgcataatatcgacatttttcatcgccgatatgacgaaaatctg   12 150
aagataatgtagccgctgtgactaggtttgcgtattatagctgtaaaaagtagcggctatactgcttttagac
175       180       185
F Y Y I G D T D P N A *
TP84_18 >

gtaaaatagggatagggaacataatgatccctatcctttttaataaaaaaatcaagggggaataaataatggca   12 225
cattttatccctatcccttgtattactagggataggaaaaattatttttttagttcccccttatttattaccgt
                                                                    1
                                                                    M A
rho-independent terminator                                          TP84_19 >

aaagtgacactgacaatcaaaaacggaaacgtgaaggaatcacaacaattcgagatcgacaaaatcacaactttc   12 300
tttcactgtgactgttagtttttgcctttgcacttccttagtgttgttaagctctagctgttttagtgttgaaag
    5         10        15        20        25
K V T L T I K N G N V K E S Q Q F E I D K I T T F
TP84_19 >

caggcattgaaactgaaaaatgaaatccatgcaatcttgaaagacttgaaaaacaatggcgaactgaaagaagtc   12 375
gtccgtaactttgactttttactttaggtacgttagaactttctgaactttttgttaccgcttgactttcttcag
    30        35        40        45        50
Q A L K L K N E I H A I L K D L K N N G E L K E V
TP84_19 >

atggaaggtctattcagcggcgaattcgatgtggacaacatggacattaaaaacatcacagcggatcaactggaa   12 450
taccttccagataagtcgccgcttaagctacacctgttgtacctgtaatttttgtagtgtcgcctagttgacctt
    55        60        65        70        75
M E G L F S G E F D V D N M D I K N I T A D Q L E
TP84_19 >

caaatgaaagacgaaaaattcatcacaagcctggcaggggcattcgatcgattgcttgaaactgtgccggaacgt   12 525
gtttactttctgctttttaagtagtgttcggaccgtccccgtaagctagctaacgaactttgacacggccttgca
    80        85        90        95        100
Q M K D E K F I T S L A G A F D R L L E T V P E R
TP84_19 >

gcgatgaatctattgtccatcatgtctggcatcgatcgtgaagtgttagaaaaagcgtatctggaagaattattc   12 600
cgctacttagataacaggtagtacagaccgtagctagcacttcacaatctttttcgcatagaccttcttaataag
    105       110       115       120       125
A M N L L S I M S G I D R E V L E K A Y L E E L F
TP84_19 >

gatgtctatgatgctgttatggaagaaaacgacatcatcaaactaattgaccgaatgaaacgatctttttttacg   12 675
ctacagatactacgacaataccttcttttgctgtagtagtttgattaactggcttactttgctagaaaaaaatgc
    130       135       140       145       150
D V Y D A V M E E N D I I K L I D R M K R S F F T
TP84_19 >
```

acaaagggccaatggtcgcaggcacttcgaacatttctggccaacaaataaacgatgacaccgatctattcgaaa 12 750
tgtttcccggttaccagcgtccgtgaagcttgtaaagaccggttgtttatttgctactgtggctagataagcttt

155 160 165
T K G Q W S Q A L R T F L A N K *
TP84_19

1 5 10 15 20
M V A G T S N I S G Q Q I N D D T D L F E
TP84_20

tgatcatatacacattgtcaccagtcctgggcggcagatccgaagtcctggccacgccgtttgtcgagtgtttga 12 825
actagtatatgtgtaacagtggtcaggacccgccgtctaggcttcaggaccggtgcggcaaacagctcacaaact

25 30 35 40 45
M I I Y T L S P V L G G R S E V L A T P F V E C L
TP84_20

aatacatcgaactagaacgacagcggaaaaaggcggatcgatggaacagatttatggatctgttttatgcacatc 12 900
ttatgtagcttgatcttgctgtcgcctttttccgcctagctaccttgtctaaatacctagacaaaatacgtgtag

50 55 60 65 70
K Y I E L E R Q R K K A D R W N R F M D L F Y A H
TP84_20

ctatggtggataagaacaaacggcaaagatatgtcgaactgatccaaccagaaagaccaagacgaaaactgacaa 12 975
gataccacctattcttgtttgccgtttctatacagcttgactaggttggtctttctggttctgcttttgactgtt

75 80 85 90 95
P M V D K N K R Q R Y V E L I Q P E R P R R K L T
TP84_20

tgcggacaaaccttgaacaattaaaggcactaaaagaacaacaagaaagagaacgtgccagaaaatcaatcagac 13 050
acgcctgtttggaacttgttaatttccgtgattttcttgttgttctttctcttgcacggtcttttagttagtctg

100 105 110 115 120
M R T N L E Q L K A L K E Q Q E R E R A R K S I R
TP84_20

aggggggatgattgaaagggggcacgataagtggcgacaatccgtgaactgcgtgcaaaattcactgcgactgcg 13 125
tcccccctactaactttcccccgtgctattcaccgctgttaggcacttgacgcacgttttaagtgacgctgacgc

125
Q G G *
TP84_20

1 5 10 15
M A T I R E L R A K F T A T A
TP84_21

aatggattcaaatcagctatacaaggcataaaaaaagatattcaaagtctgaatgatgcaagcaataaggccgcc 13 200
ttacctaagtttagtcgatatgttccgtatttttttctataagtttcagacttactacgttcgttattccggcgg

20 25 30 35 40
N G F K S A I Q G I K K D I Q S L N D A S N K A A
TP84_21

gacaacatgaacagtcgatttagcaatctgaaatcgacactggtgggattcgccggggcatatctaggattcgaa 13 275
ctgttgtacttgtcagctaaatcgttagactttagctgtgaccaccctaagcggccccgtatagatcctaagctt

45 50 55 60 65
D N M N S R F S N L K S T L V G F A G A Y L G F E
TP84_21

gcgttaaaaggcggcatcaccagtgtggcaacggcgatgatccagggaaacgccaatatggaacaataccatgcc 13 350
cgcaatttttccgccgtagtggtcacaccgttgccgctactaggtccctttgcggttataccttgttatggtacgg

70 75 80 85 90
A L K G G I T S V A T A M I Q G N A N M E Q Y H A
TP84_21

```
acactgacgactgttctgaaatcgtcagaaaaagcgacagaaatgctggcgtgggccgaaaaattcgccgcatcc   13 425
tgtgactgctgacaagactttagcagtctttttcgctgtctttacgaccgcacccggctttttaagcggcgtagg
        95        100       105       110       115
T L T T V L K S S E K A T E M L A W A E K F A A S
TP84_21

acaccatttgagatcccggacatcgttgaagcgacaacaaaactggaagtgtatgggatcagtgcgaaagaaaca   13 500
tgtggtaaactctagggcctgtagcaacttcgctgttgttttgaccttcacataccctagtcacgctttctttgt
        120       125       130       135       140
T P F E I P D I V E A T T K L E V Y G I S A K E T
TP84_21

ctgaaagacatcggtgacatggcggcgatcaccgggaaaccgttaatgcaagcggtcgaagcgatcgccgatgcc   13 575
gactttctgtagccactgtaccgccgctagtggccctttggcaattacgttcgccagcttcgctagcggctacgg
        145       150       155       160       165
L K D I G D M A A I T G K P L M Q A V E A I A D A
TP84_21

caaacaggtgaattggaacgcttgaaggaattcggtatcaccaagcagatgttgatcgacaaggcccaagaactt   13 650
gtttgtccacttaaccttgcgaacttccttaagccatagtggttcgtctacaactagctgttccgggttcttgaa
        170       175       180       185       190
Q T G E L E R L K E F G I T K Q M L I D K A Q E L
TP84_21

tatggaaaagagatcgtcaacgcaaagggccagatcactgacatggaaacaatgaacaaagcattattcgccatc   13 725
ataccttttctctagcagttgcgtttcccggtctagtgactgtacctttgttacttgtttcgtaataagcggtag
        195       200       205       210       215
Y G K E I V N A K G Q I T D M E T M N K A L F A I
TP84_21

atgcgtgaacgttatagcggtggtatggaatatatatcaaaaacgttcaatggtatgttggcgaacatcaaagat   13 800
tacgcacttgcaatatcgccaccataccttatatatagtttttgcaagttaccatacaaccgcttgtagtttcta
        220       225       230       235       240
M R E R Y S G G M E Y I S K T F N G M L A N I K D
TP84_21

tcgatgggcacaatcgccgccgaactaggaaaaccgatatttgaaaagttaaaaggtcaaatggaagatttagtg   13 875
agctacccgtgttagcggcggcttgatccttttggctataaacttttcaattttccagtttaccttctaaatcac
        245       250       255       260       265
S M G T I A A E L G K P I F E K L K G Q M E D L V
TP84_21

ccgatcatgtcggcatttacatcgttcattcgcggcgacatgtcgggtgcgatgaaaacactgacggaagcattc   13 950
ggctagtacagccgtaaatgtagcaagtaagcgccgctgtacagcccacgctacttttgtgactgccttcgtaag
        270       275       280       285       290
P I M S A F T S F I R G D M S G A M K T L T E A F
TP84_21

ggcgcaaataaagcacagcagatcatgtcctttttccagacgatcaaaaacgctggaatgggcataaaagacttt   14 025
ccgcgtttatttcgtgtcgtctagtacaggaaaaaggtctgctagtttttgcgaccttacccgtattttctgaaa
        295       300       305       310       315
G A N K A Q Q I M S F F Q T I K N A G M G I K D F
TP84_21

ttcgtgtcacttgcaccgacagtgcaaaacatcggaacgatcctggggaacatcgcaccgatcatcatcggtcca   14 100
aagcacagtgaacgtggctgtcacgttttgtagccttgctaggaccccttgtagcgtggctagtagtagccaggt
        320       325       330       335       340
F V S L A P T V Q N I G T I L G N I A P I I I G P
TP84_21
```

cttgtgattgcgtttaaagctatcgccgcggtacttccaccagttttaaacacgatcactggaatcgtggccaag 14 175
gaacactaacgcaaatttcgatagcggcgccatgaaggtggtcaaaatttgtgctagtgaccttagcaccggttc
345 350 355 360 365
L V I A F K A I A A V L P P V L N T I T G I V A K
TP84_21

tttactgaatgggaaggattcatcccactggtgacaggcctggcggcggcgatggccgtattcaaggcgcagacg 14 250
aaatgacttacccttcctaagtagggtgaccactgtccggaccgccgccgctaccggcataagttccgcgtctgc
370 375 380 385 390
F T E W E G F I P L V T G L A A A M A V F K A Q T
TP84_21

gtcgccacacagatcgtgacaaccgtggcaactagggcgacacaaatgtgggcggcggcacaaagattattgaac 14 325
cagcggtgtgtctagcactgttggcaccgttgatcccgctgtgtttacacccgccgccgtgtttctaataacttg
395 400 405 410 415
V A T Q I V T T V A T R A T Q M W A A A Q R L L N
TP84_21

ctggcgatgtctatgaatccgatgacgttgatcatcgggctgatcgttggtttggtggccgcactggttatggct 14 400
gaccgctacagatacttaggctactgcaactagtagcccgactagcaaccaaaccaccggcgtgaccaataccga
420 425 430 435 440
L A M S M N P M T L I I G L I V G L V A A L V M A
TP84_21

tatcaacggtctgaaacattccgcaacattgtcaataatgcatggcagtcgatcaaatccgtggtgtctgcggtt 14 475
atagttgccagactttgtaaggcgttgtaacagttattacgtaccgtcagctagtttaggcaccacagacgccaa
445 450 455 460 465
Y Q R S E T F R N I V N N A W Q S I K S V V S A V
TP84_21

atcaactggtttgtgacgacaatcccacaatgggtggaaaatgtcaaaaactggtttgtcggcctgggtcaacga 14 550
tagttgaccaaacactgctgttagggtgttacccaccttttacagtttttgaccaaacagccggacccagttgct
470 475 480 485 490
I N W F V T T I P Q W V E N V K N W F V G L G Q R
TP84_21

atttctgaaacgtggtcaaatatcaaaaacggcatcgtcagcatctggcagaatatcatcggaacggtcgtgccg 14 625
taaagactttgcaccagtttatagtttttgccgtagcagtcgtagaccgtcttatagtagccttgccagcacggc
495 500 505 510 515
I S E T W S N I K N G I V S I W Q N I I G T V V P
TP84_21

attgtccagtcttttgtccaggctatcgttgaacgagccaaacagatcgccacaaatgtgatgaactttattcgg 14 700
taacaggtcagaaaacaggtccgatagcaacttgctcggtttgtctagcggtgtttacactacttgaaataagcc
520 525 530 535 540
I V Q S F V Q A I V E R A K Q I A T N V M N F I R
TP84_21

ccgctgatcgactttttcaaaaacacctggaataatatcaaattgcttgtcctttcaattgttggtgtttttttg 14 775
ggcgactagctgaaaaagtttttgtggaccttattatagtttaacgaacaggaaagttaacaaccacaaaaaaac
545 550 555 560 565
P L I D F F K N T W N N I K L L V L S I V G V F L
TP84_21

aatctgctagtcgggaatttttgaaggtttgaaaatttctcttttgggaatctggacagcgatcaaaaaccaggtc 14 850
ttagacgatcagcccttaaaaacttccaaacttttaaagagaaaacccttagacctgtcgctagtttttggtccag
570 575 580 585 590
N L L V G N F E G L K I S L L G I W T A I K N Q V
TP84_21

atcaatatcgccacgacaatcaagtcaatggccgtcaacattttcacggctttgaaaaatggtgtacttgcagtg 14 925
tagttatagcggtgctgttagttcagttaccggcagttgtaaaagtgccgaaactttttaccacatgaacgtcac
595 600 605 610 615
I N I A T T I K S M A V N I F T A L K N G V L A V
TP84_21

atcaacggtttgaagtcgctggcgatctcggcgtggaacggtttgaagtcggcggcgatctcggtgtggacaggt 15 000
tagttgccaaacttcagcgaccgctagagccgcaccttgccaaacttcagccgccgctagagccacacctgtcca
620 625 630 635 640
I N G L K S L A I S A W N G L K S A A I S V W T G
TP84_21

ttgaaaaatggtgtcgtaaatacggtacaggctttgaaaaacacagcgatcaacatcgtcaacagtgtcaagtct 15 075
aactttttaccacagcatttatgccatgtccgaaactttttgtgtcgctagttgtagcagttgtcacagttcaga
645 650 655 660 665
L K N G V V N T V Q A L K N T A I N I V N S V K S
TP84_21

ggggtagtcaatgcgtttaattcagcgaaaaacctggcaatttcagcctggaacgcattgaaatctggtgtatcg 15 150
ccccatcagttacgcaaattaagtcgctttttggaccgttaaagtcggaccttgcgtaactttagaccacatagc
670 675 680 685 690
G V V N A F N S A K N L A I S A W N A L K S G V S
TP84_21

aacgcgatcaacagcgtgaaatccctggtcagcaacatgaaaaacaacattatcagcacgatcaagggaatcaac 15 225
ttgcgctagttgtcgcactttagggaccagtcgttgtactttttgttgtaatagtcgtgctagttcccttagttg
695 700 705 710 715
N A I N S V K S L V S N M K N N I I S T I K G I N
TP84_21

ttgttcgaaatgggcaaaaacgtaatccagggattcatcaaagggatcaaatcgatggtcggtgcggttggaaaa 15 300
aacaagctttacccgtttttgcattaggtccctaagtagtttccctagtttagctaccagccacgccaacctttt
720 725 730 735 740
L F E M G K N V I Q G F I K G I K S M V G A V G K
TP84_21

gccatcaaagaagtcgcatcgaacgtcacgaacaagatcaaatcggcactgggcatccattcgccatcacgtgtg 15 375
cggtagtttcttcagcgtagcttgcagtgcttgttctagtttagccgtgacccgtaggtaagcggtagtgcacac
745 750 755 760 765
A I K E V A S N V T N K I K S A L G I H S P S R V
TP84_21

ctgatggaaatcggcgcatacactggtcaaggattcgctatcggtattgagaacatgaaaaaagcggtcgtcaat 15 450
gactacctttagccgcgtatgtgaccagttcctaagcgatagccataactcttgtacttttttcgccagcagtta
770 775 780 785 790
L M E I G A Y T G Q G F A I G I E N M K K A V V N
TP84_21

gcaacacaatccctggccgatgcaacgatcggaacgatcagcagtgcagaattgaatccaaccgaaccacaagtt 15 525
cgttgtgttagggaccggctacgttgctagccttgctagtcgtcacgtcttaacttaggttggcttggtgttcaa
795 800 805 810 815
A T Q S L A D A T I G T I S S A E L N P T E P Q V
TP84_21

gcggtggcggcggccacaggagcgcagacgaactataacgcaccgctgatgtatgtcgaaaaccaatacgtgaac 15 600
cgccaccgccgccggtgtcctcgcgtctgcttgatattgcgtggcgactacatacagcttttggttatgcacttg
820 825 830 835 840
A V A A A T G A Q T N Y N A P L M Y V E N Q Y V N
TP84_21

```
gacaatacagatgtgcgtgacatttcacatggtctgtataacttgcaacgcagatcagatcgcaaaaaagggtgg   15 675
ctgttatgtctacacgcactgtaaagtgtaccagacatattgaacgttgcgtctagtctagcgttttttcccacc
      845                850                855                860               865
D  N  T  D  V  R  D  I  S  H  G  L  Y  N  L  Q  R  R  S  D  R  K  K  G  W
TP84_21 >
```

```
tgatgtagatgcctatgggctatacattcggcggtcggcacattcatgaattcctggtcgaaatgactgggaaaa   15 750
actacatctacggatacccgatatgtaagccgccagccgtgtaagtacttaggaccagctttactgaccctttt
   *         1            5              10             15             20
             M  P  M  G  Y  T  F  G  G  R  H  I  H  E  F  L  V  E  M  T  G  K
TP84_21      TP84_22 >
```

```
atgtgccgatgacaccgccgatcaaaaacctatcagaagaacaaggcggcattgacggcggatgggatttggga   15 825
tacacggctactgtggcggctagtttttggatagtcttcttgttccgccgtaactgccgcctaccctaaaccct
      25             30             35             40             45
N  V  P  M  T  P  P  I  K  N  L  S  E  E  Q  G  G  I  D  G  G  W  D  F  G
TP84_22 >
```

```
ttcaatacgaaccgaaaattatcaccatcgatcattacatcctggcgaaaaccagggaagaacggcaaaacatga   15 900
aagttatgcttggcttttaatagtggtagctagtaatgtaggaccgcttttggtcccttcttgccgttttgtact
      50             55             60             65             70
I  Q  Y  E  P  K  I  I  T  I  D  H  Y  I  L  A  K  T  R  E  E  R  Q  N  M
TP84_22 >
```

```
tccgtgaactggcaggatggctgaatccacgactgggtgcaagggaattgatattcgatgacgaaccagacaaaa   15 975
aggcacttgaccgtcctaccgacttaggtgctgacccacgttcccttaactataagctactgcttggtctgtttt
      75             80             85             90             95
I  R  E  L  A  G  W  L  N  P  R  L  G  A  R  E  L  I  F  D  D  E  P  D  K
TP84_22 >
```

```
tgtattatgcaagattatccgaacaattcgcgctggaaaaggttatcggaacatatagcgacttttcactgaatt   16 050
acataatacgttctaataggcttgttaagcgcgaccttttccaatagccttgtatatcgctgaaaagtgacttaa
      100            105            110            115            120
M  Y  Y  A  R  L  S  E  Q  F  A  L  E  K  V  I  G  T  Y  S  D  F  S  L  N
TP84_22 >
```

```
tcatctgttatgatccatttacttattcggtccaggaatacacgcaaaacatcacaggcagtggacaaatcgaac   16 125
agtagacaatactaggtaaatgaataagccaggtccttatgtgcgttttgtagtgtccgtcacctgtttagcttg
      125            130            135            140            145
F  I  C  Y  D  P  F  T  Y  S  V  Q  E  Y  T  Q  N  I  T  G  S  G  Q  I  E
TP84_22 >
```

```
acctgggaacacacgtgtcgaaaccgatcttgattgtggatcatcgcggcggatcagcaacgatcacgaatcaga   16 200
tggacccttgtgtgcacagctttggctagaactaacacctagtagcgccgcctagtcgttgctagtgcttagtct
      150            155            160            165            170
H  L  G  T  H  V  S  K  P  I  L  I  V  D  H  R  G  G  S  A  T  I  T  N  Q
TP84_22 >
```

```
cacaagacggacaaacacagacggtgacattcgcatcgactacaccgccaggtatattcacaatcgatatgaagg   16 275
gtgttctgcctgtttgtgtctgccactgtaagcgtagctgatgtggcggtccatataagtgttagctatacttcc
      175            180            185            190            195
T  Q  D  G  Q  T  Q  T  V  T  F  A  S  T  T  P  P  G  I  F  T  I  D  M  K
TP84_22 >
```

aaggaacggtcaaactgggaacgcaaagcggcgaaaaatacatcgattcaattgaatggtttgaattagaacaag 16 350
ttccttgccagtttgacccttgcgtttcgccgctttttatgtagctaagttaacttaccaaacttaatcttgttc
200 205 210 215 220
E G T V K L G T Q S G E K Y I D S I E W F E L E Q
TP84_22

ggatgaacacgatcacgcacagcggcaacatcaatcaaattacagtgaaatatcggcatacatggctatgaaaaa 16 425
cctacttgtgctagtgcgtgtcgccgttgtagttagtttaatgtcactttatagccgtatgtaccgatactttttt
225 230 235 240 245
G M N T I T H S G N I N Q I T V K Y R H T W L *
TP84_22

ggggtgatcggcatggcgattcaaaaagcgttattcacgaatcagtcagattttattcaaataggatcgaaccag 16 500
ccccactagccgtaccgctaagtttttcgcaataagtgcttagtcagtctaaaataagtttatcctagcttggtc
1 5 10 15 20
M A I Q K A L F T N Q S D F I Q I G S N Q
TP84_23

gtcggtgtcgatccttttctgtattataacggcgatcaatatcaacaaaacaaagtggccatccagatgcaacga 16 575
cagccacagctaggaaaagacataatattgccgctagttatagttgttttgtttcaccggtaggtctacgttgct
25 30 35 40 45
V G V D P F L Y Y N G D Q Y Q Q N K V A I Q M Q R
TP84_23

ttcggcagtcctgcggccatcggtccaggaatcagagggaacgcggcggcgatggatggtggatgtaccatctgg 16 650
aagccgtcaggacgccggtagccaggtccttagtctcccttgcgccgccgctacctaccacctacatggtagacc
50 55 60 65 70
F G S P A A I G P G I R G N A A A M D G G C T I W
TP84_23

cgaaatgtgccgaatccggcggctagaaaacgcatgatcagtctatggatcaagccgacagcggcggatctatca 16 725
gctttacacggcttaggccgccgatcttttgcgtactagtcagatacctagttcggctgtcgccgcctagatagt
75 80 85 90 95
R N V P N P A A R K R M I S L W I K P T A A D L S
TP84_23

cgtttttttcagtgtgatctgcacagaccgtggcgatggatggactgatcatgggatgcatctggcattgaacttc 16 800
gcaaaaaagtcacactagacgtgtctggcaccgctacctacctgactagtaccctacgtagaccgtaacttgaag
100 105 110 115 120
R F F S V I C T D R G D G W T D H G M H L A L N F
TP84_23

gatagtgcaaagcaaaaaacattgttgaatgcgcggattatcagatcaggatcgggaacatcacaatggtacaca 16 875
ctatcacgtttcgttttttgtaacaacttacgcgcctaatagtctagtcctagcccttgtagtgttaccatgtgt
125 130 135 140 145
D S A K Q K T L L N A R I I R S G S G T S Q W Y T
TP84_23

catgatccagatcaaggaacgatagaattccaggccgatcaatggtatcacatttgcttcttttatgatgcagat 16 950
gtactaggtctagttccttgctatcttaaggtccggctagttaccatagtgtaaacgaagaaaatactacgtcta
150 155 160 165 170
H D P D Q G T I E F Q A D Q W Y H I C F F Y D A D
TP84_23

ggcggcctgcctggcggtcgttatgttgcagtgtacgtgaataagacactgcaactgcaatcgttcgttgcgcca 17 025
ccgccggacggaccgccagcaatacaacgtcacatgcacttattctgtgacgttgacgttagcaagcaacgcggt
175 180 185 190 195
G G L P G G R Y V A V Y V N K T L Q L Q S F V A P
TP84_23

tttgatattactgcggcatataccagatcgttcacacttggcgacataccaaacaacggatcatatagcggcaca 17 100

aaactataatgacgccgtatatggtctagcaagtgtgaaccgctgtatggtttgttgcctagtatatcgccgtgt

200 205 210 215 220

F D I T A A Y T R S F T L G D I P N N G S Y S G T

TP84_23

tatcgattcagcggcatgattgacgaagtgatccgattgcacggtgatgatgtgtggaccattcaagaaatgcaa 17 175

atagctaagtcgccgtactaactgcttcactaggctaacgtgccactactacacacctggtaagttctttacgtt

225 230 235 240 245

Y R F S G M I D E V I R L H G D D V W T I Q E M Q

TP84_23

caatattatgacgacatcatggccggaaattacatcgatgcagaaaccgaaccaggaacgatgaaagtcggaaaa 17 250

gttataatactgctgtagtaccggcctttaatgtagctacgtctttggcttggtccttgctactttcagcctttt

250 255 260 265 270

Q Y Y D D I M A G N Y I D A E T E P G T M K V G K

TP84_23

aattttgtgactggacaatataatacgaatctgatgacatggacatcaccgacaatcgatttaggccaggatgga 17 325

ttaaaacactgacctgttatattatgcttagactactgtacctgtagtggctgttagctaaatccggtcctacct

275 280 285 290 295

N F V T G Q Y N T N L M T W T S P T I D L G Q D G

TP84_23

ttcgatgatttggtcgtgtgcagttaaacttcgaacagccgcctggtacattcatcaacatttacaccagaaca 17 400

aagctactaaaccagcacacgtcaatttgaagcttgtcggcggaccatgtaagtagttgtaaatgtggtcttgt

300 305 310 315 320

F D D F G R V Q L N F E Q P P G T F I N I Y T R T

TP84_23

tcggatgatggccaaaattgggatgcctgggtgaaaacaagtgtcgatggaacaatcaacagcagtgataagcgt 17 475

agcctactaccggttttaaccctacggacccacttttgttcacagctaccttgttagttgtcgtcactattcgca

325 330 335 340 345

S D D G Q N W D A W V K T S V D G T I N S S D K R

TP84_23

tatttgcaaatcaaaatcgaattccaaacgacaaacggtgcaataacaccaaagatcatggaagtccaggtcctg 17 550

ataaacgtttagttttagcttaaggtttgctgtttgccacgttattgtggtttctagtaccttcaggtccaggac

350 355 360 365 370

Y L Q I K I E F Q T T N G A I T P K I M E V Q V L

TP84_23

gattatcaaaaaattaagcgtctgacactgacatccgaaccattgattatttataaggatctggaaagcggcctg 17 625

ctaatagtttttaattcgcagactgtgactgtaggcttggtaactaataaatattcctagacctttcgccggac

375 380 385 390 395

D Y Q K I K R L T L T S E P L I I Y K D L E S G L

TP84_23

gaacgcatcggcgaattgaaaaacgcttatgacgttatcatcacagaagaaatcaacggtgaagaagtcatcgaa 17 700

cttgcgtagccgcttaactttttgcgaatactgcaatagtagtgtcttctttagttgccacttcttcagtagctt

400 405 410 415 420

E R I G E L K N A Y D V I I T E E I N G E E V I E

TP84_23

ttcaaaatggcatcgaacgatccgaaacgaatcgaactgggcgcagaaccagtcgaactgatcgcacggatcggt 17 775

aagttttaccgtagcttgctaggctttgcttagcttgacccgcgtcttggtcagcttgactagcgtgcctagcca

425 430 435 440 445

F K M A S N D P K R I E L G A E P V E L I A R I G

TP84_23

gataaacaatttatcattcgaaacgcgatcgataaacgtgacgaaaacggcaagaaatacacacaattttcggt 17 850
ctatttgttaaatagtaagctttgcgctagctatttgcactgcttttgccgttctttatgtgtgttaaaagcca
450 455 460 465 470
D K Q F I I R N A I D K R D E N G K K Y T Q F F G
TP84_23

gaagcgttatggtatgaattgcgcgatgctaaagtgatcaactatgaacaggtggaaaagacggcatacgaacac 17 925
cttcgcaataccatacttaacgcgctacgatttcactagttgatacttgtccaccttttctgccgtatgcttgtg
475 480 485 490 495
E A L W Y E L R D A K V I N Y E Q V E K T A Y E H
TP84_23

atccaggccatcttgaattcagcggtcgtgccgactgggtggaccatttacaaagtcgaatccgatggccgaaaa 18 000
taggtccggtagaacttaagtcgccagcacggctgacccacctggtaaatgtttcagcttaggctaccggctttt
500 505 510 515 520
I Q A I L N S A V V P T G W T I Y K V E S D G R K
TP84_23

cgaacgattcgcggcgaatggaaatctgtcctggaattattacgcgaagtcgttgatcagtttggcggcgaactg 18 075
gcttgctaagcgccgcttacctttagacaggaccttaataatgcgcttcagcaactagtcaaaccgccgcttgac
525 530 535 540 545
R T I R G E W K S V L E L L R E V V D Q F G G E L
TP84_23

caattcgacaccattaatcgcacaatcagcctggtgaaccgcatcggtgaagataacggcgttcgattctactac 18 150
gttaagctgtggtaattagcgtgttagtcggaccacttggcgtagccacttctattgccgcaagctaagatgatg
550 555 560 565 570
Q F D T I N R T I S L V N R I G E D N G V R F Y Y
TP84_23

aataagaatctgaaaacgatcgaacggtcggtggacacgtacaatctgatcacacgtctatatctatatggcaaa 18 225
ttattcttagacttttgctagcttgccagccacctgtgcatgttagactagtgtgcagatatagatataccgttt
575 580 585 590 595
N K N L K T I E R S V D T Y N L I T R L Y L Y G K
TP84_23

aacggcatgactgtccaatctgtccatccacaaggcctggaatacattgaggacttaacatgggtaaacgcgttg 18 300
ttgccgtactgacaggttagacaggtaggtgttccggaccttatgtaactcctgaattgtacccatttgcgcaac
600 605 610 615 620
N G M T V Q S V H P Q G L E Y I E D L T W V N A L
TP84_23

aatctgcgaaataagattcgcatcggtgtctggaaagatgaacgatacacgattccgcagaacctttatgatgat 18 375
ttagacgctttattctaagcgtagccacagacctttctacttgctatgtgctaaggcgtcttggaaatactacta
625 630 635 640 645
N L R N K I R I G V W K D E R Y T I P Q N L Y D D
TP84_23

ggcatgaaaatgttgcaagaaatggcgaaaccgaatgtgtcttatgcgatgactattgcggatctgtccatgttg 18 450
ccgtacttttacaacgttctttaccgctttggcttacacagaatacgctactgataacgcctagacaggtacaac
650 655 660 665 670
G M K M L Q E M A K P N V S Y A M T I A D L S M L
TP84_23

tccggccatgaacacgaatcgatcggcctgggcgacaccgtctgggtagtggacaccgaactgatgaatctgctt 18 525
aggccggtacttgtgcttagctagccggacccgctgtggcagacccatcacctgtggcttgactacttagacgaa
675 680 685 690 695
S G H E H E S I G L G D T V W V V D T E L M N L L
TP84_23

```
gtggaagcgcgcatcgtgcggcgaaaatacaacgtgcggcaaccgtggaaaacagaagtcgaattgaatcaaccg   18 600
caccttcgcgcgtagcacgccgcttttatgttgcacgccgttggcaccttttgtcttcagcttaacttagttggc
         700                      705                      710                      715                      720
V    E    A    R    I    V    R    R    K    Y    N    V    R    Q    P    W    K    T    E    V    E    L    N    Q    P
TP84_23

aaaaaggaactggccgatgcgaatcggcgtgctattgacgatgcaatcgaaacgctagtggaaagcgatccactg   18 675
ttttccttgaccggctacgcttagccgcacgataactgctacgttagctttgcgatcacctttcgctaggtgac
         725                      730                      735                      740                      745
K    K    E    L    A    D    A    N    R    R    A    I    D    D    A    I    E    T    L    V    E    S    D    P    L
TP84_23

gacacaagtgacgttcaacagatgactgtgttcaatcatttattgaattcacgtgcagaagatggcacgaaatac   18 750
ctgtgttcactgcaagttgtctactgacacaagttagtaaataacttaagtgcacgtcttctaccgtgctttatg
         750                      755                      760                      765                      770
D    T    S    D    V    Q    Q    M    T    V    F    N    H    L    L    N    S    R    A    E    D    G    T    K    Y
TP84_23

tgggaagtgactggatcagacatcacgatcgaaccgtctggattcagtggtaatgcatcttggaaaatacaaagc   18 825
acccttcactgacctagtctgtagtgctagcttggcagacctaagtcaccattacgtagaaccttttatgtttcg
         775                      780                      785                      790                      795
W    E    V    T    G    S    D    I    T    I    E    P    S    G    F    S    G    N    A    S    W    K    I    Q    S
TP84_23

ggctatggaaaaacaaataaactgaaacaatccatttatggtgtgtcacatcgttcggcgtacactatcagtgca   18 900
ccgataccttttgtttatttgactttgttaggtaaataccacacagtgtagcaagccgcatgtgatagtcacgt
         800                      805                      810                      815                      820
G    Y    G    K    T    N    K    L    K    Q    S    I    Y    G    V    S    H    R    S    A    Y    T    I    S    A
TP84_23

tatgtggccacagaaggaacgatcaccaggggaacatcacaggatgcgttcgtggggatcaaggtgacgattcac   18 975
atacaccggtgtcttccttgctagtggtccccttgtagtgtcctacgcaagcacccctagttccactgctaagtg
         825                      830                      835                      840                      845
Y    V    A    T    E    G    T    I    T    R    G    T    S    Q    D    A    F    V    G    I    K    V    T    I    H
TP84_23

tacactgaaccagacagcgatggcaaaacatatgaagaacacttcctggcgatcccggacatcacacaacaaggc   19 050
atgtgacttggtctgtcgctaccgttttgtatacttcttgtgaaggaccgctagggcctgtagtgtgttgttccg
         850                      855                      860                      865                      870
Y    T    E    P    D    S    D    G    K    T    Y    E    E    H    F    L    A    I    P    D    I    T    Q    Q    G
TP84_23

ggtgaagataatggcggttaattcgctgaattttgaactgatcgtgaagaagatagaaacagacacatcgcggaa   19 125
ccacttctattaccgccaattaagcgacttaaaacttgactagcacttcttctatctttgtctgtgtagcgcctt
         875
G    E    D    N    G    G    *
TP84_23
                 1                   5                   10                  15                  20
                 M    A    V    N    S    L    N    F    E    L    I    V    K    K    I    E    T    D    T    S    R    K
                 TP84_24

agtggatcacatcgatgtcgagatcatcgccaatgactacacaggaacgttttatttttacggatataatgtttca   19 200
tcacctagtgtagctacagctctagtagcggttactgatgtgtccttgcaaaataaaaatgcctatattacaaagt
         25                       30                       35                       40                       45
 V    D    H    I    D    V    E    I    I    A    N    D    Y    T    G    T    F    Y    F    T    D    I    M    F    Q
TP84_24
```

atcgggaacggtggccacatcctgggtgggccacgtgtctgaaatcagatggtcattcgataacgcatagggggg 19 275
tagcccttgccaccggtgtaggacccacccggtgcacagactttagtctaccagtaagctattgcgtatcccccc
50 55 60 65 70
S G T V A T S W V G H V S E I R W S F D N A *
TP84_24

gatcgtgatgccaaagatcacagactggaaagtgtttggcggcacggtcgaactggaacggaaacacatgaaagt 19 350
ctagcactacggtttctagtgtctgacctttcacaaaccgccgtgccagcttgaccttgcctttgtgtactttca
1 5 10 15 20
M P K I T D W K V F G G T V E L E R K H M K V
TP84_25

ggaccacgtggatctggaaatcgtggtccacgacaccgtttcatggaaggatgggccacaaccaatctatttcac 19 425
cctggtgcacctagacctttagcaccaggtgctgtggcaaagtaccttcctacccggtgttggttagataaagtg
25 30 35 40 45
D H V D L E I V V H D T V S W K D G P Q P I Y F T
TP84_25

tgatctacaattccaaccaggccaccaaaagacagggtggattccaaacacacaagaattcctggatcgtgttga 19 500
actagatgttaaggttggtccggtggttttctgtcccacctaaggtttgtgtgttcttaaggacctagcacaact
50 55 60 65 70
D L Q F Q P G H Q K T G W I P N T Q E F L D R V E
TP84_25

attcactgtcgatgaactgcggcgataccgactggcagatggatcggtcgatccttattttcaatttccgccagg 19 575
taagtgacagctacttgacgccgctatggctgaccgtctacctagccagctaggaataaaagttaaaggcggtcc
75 80 85 90 95
F T V D E L R R Y R L A D G S V D P Y F Q F P P G
TP84_25

tgtgacaccgcgaacctacacaccagaagaacttggctatcagcggcttttcaatattatgggccgcggccatga 19 650
acactgtggcgcttggatgtgtggtcttcttgaaccgatagtcgccgaaaagttataatacccggcgccggtact
100 105 110 115 120
V T P R T Y T P E E L G Y Q R L F N I M G R G H E
TP84_25

agttattgtcctgccgaatgatctgccagaaccagaattctgggatcttgatctgattgcacaaaaaggcctgga 19 725
tcaataacaggacggcttactagacggtcttggtcttaagaccctagaactagactaacgtgtttttccggacct
125 130 135 140 145
V I V L P N D L P E P E F W D L D L I A Q K G L E
TP84_25

acggccagttgagattctttccactgggattgacttcacgatcatcccaaaagatgacttcgaactgatgcgact 19 800
tgccggtcaactctaagaaaggtgaccctaactgaagtgctagtagggttttctactgaagcttgactacgctga
150 155 160 165 170
R P V E I L S T G I D F T I I P K D D F E L M R L
TP84_25

ttcaaacaatatcggtgcattgttgccggaggaagaacagaaatatccagacgatcccgaacatccactgaatta 19 875
aagtttgttatagccacgtaacaacggcctccttcttgtctttataggtctgctagggcttgtaggtgacttaat
175 180 185 190 195
S N N I G A L L P E E E Q K Y P D D P E H P L N Y
TP84_25

tcgatataccagggaattttggatcggaagcggccacgcaggggatgtcatcgagatcaacgccaccaccatgac 19 950
agctatatggtcccttaaaacctagccttcgccggtgcgtcccctacagtagctctagttgcggtggtggtactg
200 205 210 215 220
R Y T R E F W I G S G H A G D V I E I N A T T M T
TP84_25

ggccaaagtgaatggcatcaccatcaacacacaagggatcaaacaaatcacgactggcagtgatacaataaaaat 20 025
ccggtttcacttaccgtagtggtagttgtgtgttccctagtttgtttagtgctgaccgtcactatgttattttta
225 230 235 240 245
A K V N G I T I N T Q G I K Q I T T G S D T I K I
TP84_25

atacaaaaacaaatttcatctgatgccgcgtggatcggtccgattccgtgtcgaattttatggccgggatgcaaa 20 100
tatgtttttgtttaaagtagactacggcgcacctagccaggctaaggcacagcttaaaataccggccctacgttt
250 255 260 265 270
Y K N K F H L M P R G S V R F R V E F Y G R D A N
TP84_25

tggccgcctggccgacactgggatcggttatcgcggcacagcaacatttaaacaatggacatatggggtggaaag 20 175
accggcggaccggctgtgaccctagccaatagcgccgtgtcgttgtaaatttgttacctgtataccccacctttc
275 280 285 290 295
G R L A D T G I G Y R G T A T F K Q W T Y G V E R
TP84_25

actatgacgaaacaaaaacgtgtgatgacatggtcactgcggattcaaaacgatgactatcgacacgacatcgaa 20 250
tgatactgctttgtttttgcacactactgtaccagtgacgcctaagttttgctactgatagctgtgctgtagctt
1 5 10 15 20
M T K Q K R V M T W S L R I Q N D D Y R H D I E
TP84_26

300
L *
TP84_25

cgttatggaaacaagatcacatcgatcggattccatgaattcaatgtcgatgcacagggccggatcgccatctat 20 325
gcaataccttgttctagtgtagctagcctaaggtacttaagttacagctacgtgtcccggcctagcggtagata
25 30 35 40 45
R Y G N K I T S I G F H E F N V D A Q G R I A I Y
TP84_26

cgtctagggccacaaaaggatgcacagggatcgttcacatccttcacagatgccaccacatatgatcgttatgcg 20 400
gcagatcccggtgttttcctacgtgtccctagcaagtgtaggaagtgtctacggtggtgtatactagcaatacgc
50 55 60 65 70
R L G P Q K D A Q G S F T S F T D A T T Y D R Y A
TP84_26

ccagggcggacattatggccgaattacatcgaaaaagacatgaagcggtggccgcatatcgaatattacatgcag 20 475
ggtcccgcctgtaataccggcttaatgtagctttttctgtacttcgccaccggcgtatagcttataatgtacgtc
75 80 85 90 95
P G R T L W P N Y I E K D M K R W P H I E Y Y M Q
TP84_26

tttgttatattcggtccagaaacggtgtcacgactgctggattcacaaacggcgcaggataactttatcaacaat 20 550
aaacaatataagccaggtctttgccacagtgctgacgacctaagtgtttgccgcgtcctattgaaatagttgtta
100 105 110 115 120
F V I F G P E T V S R L L D S Q T A Q D N F I N N
TP84_26

ctgaaaacggtcgtggccagattccgaaaggatcagaacggaaatgatctgggatacaccgggatcgaaatcgac 20 625
gacttttgccagcaccggtctaaggctttcctagtcttgcctttactagaccctatgtggccctagctttagctg
125 130 135 140 145
L K T V V A R F R K D Q N G N D L G Y T G I E I D
TP84_26

tgcgaaggatcattcagtgattcgaaatgggatacacgcgccggggatgatgtgaagtatatcaatctattgaaa 20 700
acgcttcctagtaagtcactaagctttaccctatgtgcgcggcccctactacacttcatatagttagataacttt
150 155 160 165 170
C E G S F S D S K W D T R A G D D V K Y I N L L K
TP84_26

cggattaaaaatgaagtgatcatcgatgcgaatccagattttaaattacggatcaacgcacatgcgatgtggggc 20 775
gcctaattttacttcactagtagctacgcttaggtctaaaatttaatgcctagttgcgtgtacgctacaccccg
175 180 185 190 195
R I K N E V I I D A N P D F K L R I N A H A M W G
TP84_26

gatggaatcccggattattatcgattccataactataaattgtttgccgaatccaccgataaaaacggaaatccg 20 850
ctaccttagggcctaataatagctaaggtattgatatttaacaaacggcttaggtggctatttttgcctttaggc
200 205 210 215 220
D G I P D Y Y R F H N Y K L F A E S T D K N G N P
TP84_26

ttgctggatgaagtgcagatcatgacgtatgacttttcctggtctggatctgcgccagggccatcgacaccattg 20 925
aacgacctacttcacgtctagtactgcatactgaaaaggaccagacctagacgcggtcccggtagctgtggtaac
225 230 235 240 245
L L D E V Q I M T Y D F S W S G S A P G P S T P L
TP84_26

tggtggatgcgaaatgtggccgaatgggtgaaacagtgttttgatccatcggtgaatccgaacgcaaaatgcacg 21 000
accacctacgctttacaccggcttacccactttgtcacaaaactaggtagccacttaggcttgcgttttacgtgc
250 255 260 265 270
W W M R N V A E W V K Q C F D P S V N P N A K C T
TP84_26

attgacaatgtgtatctgggcggcgcagggtatggccgcagatggccgattcattcggatgataactggggatcg 21 075
taactgttacacatagacccgccgcgtcccataccggcgtctaccggctaagtaagcctactattgacccctagc
275 280 285 290 295
I D N V Y L G G A G Y G R R W P I H S D D N W G S
TP84_26

acagtgacatatcgggatctcgttgactggcaaaacggttatttgatccaccacctgggcggcggccaactggca 21 150
tgtcactgtatagccctagagcaactgaccgttttgccaataaactaggtggtggacccgccgccggttgaccgt
300 305 310 315 320
T V T Y R D L V D W Q N G Y L I H H L G G G Q L A
TP84_26

gaccaggattttatccctttaaatgcgtttaatgatccaaacagtgacaaccagatcatgctgatgcatcaatat 21 225
ctggtcctaaaatagggaaatttacgcaaattactaggtttgtcactgttggtctagtacgactacgtagttata
325 330 335 340 345
D Q D F I P L N A F N D P N S D N Q I M L M H Q Y
TP84_26

gactatttcaaagcgcgtcacatgaagcgatatgattcgatggggcagacgacagtgcgtgtatccgaatataac 21 300
ctgataaagtttcgcgcagtgtacttcgctatactaagctaccccgtctgctgtcacgcacataggcttatattg
350 355 360 365 370
D Y F K A R H M K R Y D S M G Q T T V R V S E Y N
TP84_26

ggcatcgaatatgcaacggcctattcaaagacgcaacatgctaaaattagcggtctggcaggggtgacaggggtg 21 375
ccgtagcttatacgttgccggataagtttctgcgttgtacgatttaatcgccagaccgtccccactgtccccac
375 380 385 390 395
G I E Y A T A Y S K T Q H A K I S G L A G V T G V
TP84_26

aaaggtgtcagcgaaccgacacacaacctttcaccatatgataatccgaaacgcggtgaaacagtcaccattgat 21 450
tttccacagtcgcttggctgtgtgttggaaagtggtatactattaggctttgcgccactttgtcagtggtaacta
400 405 410 415 420
K G V S E P T H N L S P Y D N P K R G E T V T I D
TP84_26

tttgggaatggccagtcatacacattccaggcataccaaacgaaacgtgtgccgtatgtaccgacagcaatcaaa 21 525
aaacccttaccggtcagtatgtgtaaggtccgtatggtttgctttgcacacggcatacatggctgtcgttagttt
425 430 435 440 445
F G N G Q S Y T F Q A Y Q T K R V P Y V P T A I K
TP84_26

gatgcaaacggaaaaataacaggatatgcgtgtcagttatccgaacagccagaaactgttttgacatacacgtta 21 600
ctacgtttgccttttattgtcctatacgcacagtcaataggcttgtcggtctttgacaaaactgtatgtgcaat
450 455 460 465 470
D A N G K I T G Y A C Q L S E Q P E T V L T Y T L
TP84_26

aatgtgccgcaagctggaacatacaagatcggcgcaattgtgtcatttccattttataactatacgaaactaggt 21 675
ttacacggcgttcgaccttgtatgttctagccgcgttaacacagtaaaggtaaaatattgatatgctttgatcca
475 480 485 490 495
N V P Q A G T Y K I G A I V S F P F Y N Y T K L G
TP84_26

ggacacataaatggcgcaccattcacgatcggcggcgacaatttaccagactattatccgatgatgttcaaagca 21 750
cctgtgtatttaccgcgtggtaagtgctagccgccgctgttaaatggtctgataataggctactacaagtttcgt
500 505 510 515 520
G H I N G A P F T I G G D N L P D Y Y P M M F K A
TP84_26

tgtcacatctgggatcttggcacacattcattgaacgccggagcaaacaccatcacgatcgaagggcctatgtct 21 825
acagtgtagaccctagaaccgtgtgtaagtaacttgcggcctcgtttgtggtagtgctagcttcccggatacaga
525 530 535 540 545
C H I W D L G T H S L N A G A N T I T I E G P M S
TP84_26

gcacatggcacgatcattttcggcttttttgcgtgtcaatcactgacactggaagtgatcggcggatatatggac 21 900
cgtgtaccgtgctagtaaaagccgaaaaaacgcacagttagtgactgtgaccttcactagccgcctatatacctg
550 555 560 565 570
A H G T I I F G F F A C Q S L T L E V I G G Y M D
TP84_26

tgcgaatcgacaatatatccttacaaaaaacgtgatggatcagatgccagactgcctgcacaattcgcactgaca 21 975
acgcttagctgttatataggaatgttttttgcactacctagtctacggtctgacggacgtgttaagcgtgactgt
575 580 585 590 595
C E S T I Y P Y K K R D G S D A R L P A Q F A L T
TP84_26

tcggaagtattgcaacaatcgccgcggccagtgatcatgtgggaagatgtattcagacagtatttgaatgatgaa 22 050
agccttcataacgttgttagcggcgccggtcactagtacacccttctacataagtctgtcataaacttactactt
600 605 610 615 620
S E V L Q Q S P R P V I M W E D V F R Q Y L N D E
TP84_26

tttgttcaacaatacggcctggaagctacaacctattacagattgacaggacagaaaaaagactatggcggcggc 22 125
aaacaagttgttatgccggaccttcgatgttggataatgtctaactgtcctgtcttttttctgataccgccgccg
625 630 635 640 645
F V Q Q Y G L E A T T Y Y R L T G Q K K D Y G G G
TP84_26

acgattgaagaatggacagatgacgtgctgactggatgttatgcgctagtggacaacggattcacacaaggtcac 22 200
tgctaacttcttacctgtctactgcacgactgacctacaatacgcgatcacctgttgcctaagtgtgttccagtg
650 655 660 665 670
T I E E W T D D V L T G C Y A L V D N G F T Q G H
TP84_26

tggccagtggaaacagacgaaaacggcacagcgtgtgcaagattccggcctaatgatccgaaaaacaacggtgtg 22 275
accggtcacctttgtctgcttttgccgtgtcgcacacgttctaaggccggattactaggctttttgttgccacac
675 680 685 690 695
W P V E T D E N G T A C A R F R P N D P K N N G V
TP84_26

acatctggacagttagttttgaattacacatataaaaccacgaacatttcctgcgaagtgcaattcaaagtgaaa 22 350
tgtagacctgtcaatcaaaacttaatgtgtatattttggtgcttgtaaaggacgcttcacgttaagtttcacttt
700 705 710 715 720
T S G Q L V L N Y T Y K T T N I S C E V Q F K V K
TP84_26

tcgggtagacgcgccgggatcagattcgcatccactggaccaggggatggatatgtctttttaatcgattatcaa 22 425
agcccatctgcgcggccctagtctaagcgtaggtgacctggtcccctacctatacagaaaaattagctaatagtt
725 730 735 740 745
S G R R A G I R F A S T G P G D G Y V F L I D Y Q
TP84_26

acacaggaagccatgatgttctatgaaaccgctggatcatcgcaactggtggccagtgcatccctgggggataga 22 500
tgtgtccttcggtactacaagatactttggcgacctagtagcgttgaccaccggtcacgtagggaccccctatct
750 755 760 765 770
T Q E A M M F Y E T A G S S Q L V A S A S L G D R
TP84_26

agggccgactatgatgaattgatcacattgaaagtcctggtcaataacggaaaatgtcggtgctatttcggcaac 22 575
tcccggctgatactacttaactagtgtaactttcaggaccagttattgccttttacagccacgataaagccgttg
775 780 785 790 795
R A D Y D E L I T L K V L V N N G K C R C Y F G N
TP84_26

gtgatgttctttatggatatgaatctgccgcacatgtcgccaggcggcatcggattcgtggcaacaaattgcgat 22 650
cactacaagaaatacctatacttagacggcgtgtacagcggtccgccgtagcctaagcaccgttgtttaacgcta
800 805 810 815 820
V M F F M D M N L P H M S P G G I G F V A T N C D
TP84_26

gcctatctatataaactatccatcggaacgactgaacgatgggaaacacttgaacgattcagtgtcatcatcgat 22 725
cggatagatatatttgataggtagccttgctgacttgctaccctttgtgaacttgctaagtcacagtagtagcta
825 830 835 840 845
A Y L Y K L S I G T T E R W E T L E R F S V I I D
TP84_26

ggccaggaatataagatgggcgaaatcagcagaccgggaatcaaccgggatcagtggggatttttgatatattct 22 800
ccggtccttatattctacccgctttagtcgtctggcccttagttggccctagtcacccctaaaaactatataaga
850 855 860 865 870
G Q E Y K M G E I S R P G I N R D Q W G F L I Y S
TP84_26

ggattcaatgaatacaacaccagggaagtcctgccggatggatcgcggccagaaatcagtctggactatgtattt 22 875
cctaagttacttatgttgtggtcccttcaggacggcctacctagcgccggtctttagtcagacctgatacataaa
875 880 885 890 895
G F N E Y N T R E V L P D G S R P E I S L D Y V F
TP84_26

aaaccgatccatgtgccatcctggaccgggaaaaagaagatcacgatcaagctgatcgatgccgggctatggtat 22 950
tttggctaggtacacggtaggacctggccctttttcttctagtgctagttcgactagctacggcccgataccata
900 905 910 915 920
K P I H V P S W T G K K K I T I K L I D A G L W Y
TP84_26

aaacagctatacatcggcgatgcaaacggcatgtcgatcgcatacgcaggggatgaagaatcgttcgacagggcg 23 025
tttgtcgatatgtagccgctacgtttgccgtacagctagcgtatgcgtcccctacttcttagcaagctgtcccgc
925 930 935 940 945
K Q L Y I G D A N G M S I A Y A G D E E S F D R A
TP84_26

atgaacatcgcggtgcatgaatatggatgcaaagggatcggtttgtgggtattaggtcaagcagatccgcgcatc 23 100
tacttgtagcgccacgtacttatacctacgtttccctagccaaacacccataatccagttcgtctaggcgcgtag
950 955 960 965 970
M N I A V H E Y G C K G I G L W V L G Q A D P R I
TP84_26

tttgaaacattgccggatgtagtgccgtggcatccagatccgaacgaaaactaaaaaaacacggccaaatgatca 23 175
aaactttgtaacggcctacatcacggcaccgtaggtctaggcttgcttttgatttttttgtgccggtttactagt
975 980 985 990
F E T L P D V V P W H P D P N E N *
TP84_26

gcaaaatatggtaaaatttaaacaagaacaggggggtgaataaaaataaaggaggtacaacgttttgaaaacgcc 23 250
cgttttataccattttaaatttgttcttgtccccccacttatttttatttcctccatgttgcaaaacttttgcgg
1
M K T P
TP84_27

gcaacaaaccgacacgctattcactgcgatcgccggaggatttacatcgacagtggcttatttgatcggcggtgt 23 325
cgttgtttggctgtgcgataagtgacgctagcggcctcctaaatgtagctgtcaccgaataaactagccgccaca
5 10 15 20 25
Q Q T D T L F T A I A G G F T S T V A Y L I G G V
TP84_27

ggacaacctttcaaacgcgctgacggtgttcatgattttggattatttcactggcatcctatccgctttctatac 23 400
cctgttggaaagtttgcgcgactgccacaagtactaaaacctaataaagtgaccgtaggataggcgaaagatatg
30 35 40 45 50
D N L S N A L T V F M I L D Y F T G I L S A F Y T
TP84_27

gcgccaggtgaacagctacctggcatatagaggactggcaaaaaaagcagggatgatcgcattcgttattgtggc 23 475
cgcggtccacttgtcgatggaccgtatatctcctgaccgtttttttcgtccctactagcgtaagcaataacaccg
55 60 65 70 75
R Q V N S Y L A Y R G L A K K A G M I A F V I V A
TP84_27

gaatcaattggacatcatcactggcaacacggaagggtttctgcgtgatgccatgatgatgtttctgatcgggat 23 550
cttagttaacctgtagtagtgaccgttgtgccttcccaaagacgcactacggtactactacaaagactagcccta
80 85 90 95 100
N Q L D I I T G N T E G F L R D A M M M F L I G M
TP84_27

ggaaggcatttcgatcaaagagaatgtcgagaaaatgggattcaatgcaccaggattcattgtggaagcattgaa 23 625
ccttccgtaaagctagtttctcttacagctcttttaccctaagttacgtggtcctaagtaacaccttcgtaactt
105 110 115 120 125
E G I S I K E N V E K M G F N A P G F I V E A L K
TP84_27

```
aaagctgatggggaatgataaaaacgattcatcgaagggggcttgataacatgcaagcaagatccgcgaacaata   23 700
tttcgactaccccttactatttttgctaagtagcttcccccgaactattgtacgttcgttctaggcgcttgttat
130  135  140                1  5
K L M G N D K N D S S K G A *   M Q A R S A N N
TP84_27                         TP84_28
```

```
tcaaagggatcgatgtgtcgcactggcaaggaaagattgactgggcgaaagtgaaagccgctggcatccaggtcg   23 775
agtttccctagctacacagcgtgaccgttcctttctaactgacccgctttcactttcggcgaccgtaggtccagc
10  15  20  25  30
I K G I D V S H W Q G K I D W A K V K A A G I Q V
TP84_28
```

```
cttatttgaaagcgacagaaggaacgacacatgtggataaaatgttaaaaacaaactatcagaacgcaaaaaaag   23 850
gaataaactttcgctgtcttccttgctgtgtacacctattttacaatttttgtttgatagtcttgcgtttttttc
35  40  45  50  55
A Y L K A T E G T T H V D K M L K T N Y Q N A K K
TP84_28
```

```
ccgggatcaaagtgggatttatcactttttccgggcaaaaaacgaacaaaacgcacgtgaacaagccagacatt   23 925
ggccctagtttcaccctaaaatagtgaaaaaggcccgttttttgcttgttttgcgtgcacttgttcggtctgtaa
60  65  70  75  80
A G I K V G F Y H F F R A K N E Q N A R E Q A R H
TP84_28
```

```
tcgtgaacacagtcaaagggatgccgaacgatctgaaacacgcgctggacatcgaaaccaccgaaggcctatcga   24 000
agcacttgtgtcagtttccctacggcttgctagactttgtgcgcgacctgtagctttggtggcttccggatagct
85  90  95  100  105
F V N T V K G M P N D L K H A L D I E T T E G L S
TP84_28
```

```
atgaagcgttgacgaaatgcgcgattgcattcctggaagaagtgaaaaagctgactggtcaagatccgatcgttt   24 075
tacttcgcaactgctttacgcgctaacgtaaggaccttcttcacttttttcgactgaccagttctaggctagcaaa
110  115  120  125  130
N E A L T K C A I A F L E E V K K L T G Q D P I V
TP84_28
```

```
acacatacacatcattcgcaagatcccgattaacagcggctatcgcaaaatatccagtgtggatcgcacattatg   24 150
tgtgtatgtgtagtaagcgttctagggctaattgtcgccgatagcgttttataggtcacacctagcgtgtaatac
135  140  145  150  155
Y T Y T S F A R S R L T A A I A K Y P V W I A H Y
TP84_28
```

```
gcgtggacaagcctggcgacaatccgatctgggatcgatggatcggattccagtacactgacaaagggaaagtca   24 225
cgcacctgttcggaccgctgttaggctagaccctagctacctagcctaaggtcatgtgactgtttccctttcagt
160  165  170  175  180
G V D K P G D N P I W D R W I G F Q Y T D K G K V
TP84_28
```

```
gcggcatcgctggaaatgtggacatgaatgaatttacgtcagacatttttgtcgatgcggccaaagtggaacagc   24 300
cgccgtagcgacctttacacctgtacttacttaaatgcagtctgtaaaaacagctacgccggtttcaccttgtcg
185  190  195  200  205
S G I A G N V D M N E F T S D I F V D A A K V E Q
TP84_28
```

```
cgaaacaaaaggtcgatgctgtccaatccacgccatcggccactggcacatatacgatcaaaagcggcgacacgt   24 375
gctttgttttccagctacgacaggttaggtgcggtagccggtgaccgtgtatatgctagttttcgccgctgtgca
210  215  220  225  230
P K Q K V D A V Q S T P S A T G T Y T I K S G D T
TP84_28
```

```
tctgggaactagaagaaaagtataactggccacatggcacactgcaaagattgaatccgtcagtgaatccgaacg   24 450
agacccttgatcttcttttcatattgaccggtgtaccgtgtgacgtttctaacttaggcagtcacttaggcttgc
    235       240       245       250       255
F W E L E E K Y N W P H G T L Q R L N P S V N P N
TP84_28 >

cattgaaagtcggccaggtgataaaagtaccaaaatccgaacagccgaaacaaaatgcatcatcagtcactggca   24 525
gtaactttcagccggtccactattttcatggttttaggcttgtcggctttgttttacgtagtagtcagtgaccgt
    260       265       270       275       280
A L K V G Q V I K V P K S E Q P K Q N A S S V T G
TP84_28 >

catatacgatcaaaagcggcgacacgttctgggatctggaacaaaaaaacggatggccacacggcacgttacaaa   24 600
gtatatgctagttttcgccgctgtgcaagaccctagaccttgtttttttgcctaccggtgtgccgtgcaatgttt
    285       290       295       300       305
T Y T I K S G D T F W D L E Q K N G W P H G T L Q
TP84_28 >

aattgaatccaggcgtgaatccgaacaaattgaaagtcggccaggtgataaaagtaccaaaatccgaacaaaaga   24 675
ttaacttaggtccgcacttaggcttgtttaactttcagccggtccactattttcatggttttaggcttgttttct
    310       315       320       325       330
K L N P G V N P N K L K V G Q V I K V P K S E Q K
TP84_28 >

atgtgcagcggaccgtcaaaaaccaccaaaaaccgaactatcgaacgtataaaataaagaaaggtgacacgttct   24 750
tacacgtcgcctggcagtttttggtggtttttggcttgatagcttgcatattttatttctttccactgtgcaaga
    335       340       345       350       355
N V Q R T V K N H Q K P N Y R T Y K I K K G D T F
TP84_28 >

gggaacttgaaaagaaaaatggctggccacatggaacgttgcagaaattgaatccaggcgtgaatccggcgaaac   24 825
cccttgaacttttcttttaccgaccggtgtaccttgcaacgtctttaacttaggtccgcacttaggccgctttg
    360       365       370       375       380
W E L E K K N G W P H G T L Q K L N P G V N P A K
TP84_28 >

tgcaaatcggccaaacgatcaaaattccaaactaaagagaggggaagccgcatgaagaacttgaaggcaaaagac   24 900
acgtttagccggtttgctagttttaaggtttgatttctctccccttcggcgtacttcttgaacttccgttttctg
    385       390       395                 1         5
L Q I G Q T I K I P N *                   M K N L K A K D
TP84_28 >                                 TP84_29 >

tggatcagcatcattaaatatttacttcctgtcatcgcactggtgatcgcaaagatcgcagatgcaggcgatcca   24 975
acctagtcgtagtaatttataaatgaaggacagtagcgtgaccactagcgtttctagcgtctacgtccgctaggt
     10        15        20        25        30
W I S I I K Y L L P V I A L V I A K I A D A G D P
TP84_29 >

accaagattcaaaacgcactggaaatcttccttactgctttactgggattacttggcgcaattggtatcattaaa   25 050
tggttctaagttttgcgtgacctttagaaggaatgacgaaatgaccctaatgaaccgcgttaaccatagtaattt
     35        40        45        50        55
T K I Q N A L E I F L T A L L G L L G A I G I I K
TP84_29 >
```

agtcatgacaaaactgacgatcagtgatattatatgaagtgccggcctatgttctattttcctgtgaaccttcga 25 125
tcagtactgttttgactgctagtcactataatatacttcacggccggatacaagataaaaggacacttggaagct
60 65
S H D K T D D Q *
TP84_29
putative host-dependent promoter

cacgtttttcgaaatgtgcaagtgttctgcatcgatattcttcattctcctatgatccgccatccaaacggata 25 200
gtgcaaaaagctttacacgttcacaagacgtagctataagaagtaagaggatactaggcggtaggtttgcctat
rho-independent terminator

gcggatctttttttgttcgtttacattatatttttatatttttaaaaatcatcgttgacattgtccacgatcgca 25 275
cgcctagaaaaaaacaagcaaatgtaatataaaaatataaaaatttttagtagcaactgtaacaggtgctagcgt
rho-independent terminator
putative host-dependent promoter

tgatagtataaaggcaacaaaggaaaatatttcattaacagggggaatgttaaatggtgaatatcgaaacaaaaa 25 350
actatcatatttccgttgtttccttttataaagtaattgtcccccttacaatttaccacttatagctttgttttt
1 5
M V N I E T K
TP84_30
putative host-dependent promoter

caagaaaaacagccgctaaaaatagattgcgtgcgaaacatgtaatcatcacagcggtgttgtcaatggtgttga 25 425
gttctttttgtcggcgatttttatctaacgcacgctttgtacattagtagtgtcgccacaacagttaccacaact
10 15 20 25 30
T R K T A A K N R L R A K H V I I T A V L S M V L
TP84_30

ttttggcatcttgatcggtctgaaaaacctggtggccgatctaatcaatgcgaatgaagcagtggtcgatgatc 25 500
aaaaccgtagaactagccagactttttggaccaccggctagattagttacgcttacttcgtcaccagctactag
35 40 45 50 55
I F G I L I G L K N L V A D L I N A N E A V V D D
TP84_30

aaacgacagaaacgctggatcgtgtatatgaattggcgacaagcgatgatccagaacttgacgtgacagtcgaat 25 575
tttgctgtctttgcgacctagcacatatacttaaccgctgttcgctactaggtcttgaactgcactgtcagctta
60 65 70 75 80
Q T T E T L D R V Y E L A T S D D P E L D V T V E
TP84_30

ggaacgatgtgcaagtgcaaaacgcgatccacaaaatgtcgcaccaaaaagtcttggcagatgtgaaatggggtg 25 650
ccttgctacacgttcacgttttgcgctaggtgttttacagcgtggtttttcagaaccgtctacactttaccccac
85 90 95 100 105
W N D V Q V Q N A I H K M S H Q K V L A D V K W G
TP84_30

cgatcaagatcacgaaaaaacgtctagaaacattgtcgatgatcatcgatcaaaacaaagaccatctggaacact 25 725
gctagttctagtgcttttttgcagatctttgtaacagctactagtagctagttttgtttctggtagaccttgtga
110 115 120 125 130
A I K I T K K R L E T L S M I I D Q N K D H L E H
TP84_30

atgatctatatcgcgacatcttggatcgctggatggaaggtgacttcagtcgcgcagacgaagatcacaacgcga 25 800
tactagatatagcgctgtagaacctagcgacctaccttccactgaagtcagcgcgtctgcttctagtgttgcgct
135 140 145 150 155
Y D L Y R D I L D R W M E G D F S R A D E D H N A
TP84_30

tctgggaactgcaaggcggcacgatcgggaaagcctatggattacaaaccaaagaagaagaacaagaattcatca
agacccttgacgttccgccgtgctagccctttcggatacctaatgtttggtttcttcttcttgttcttaagtagt 25 875

160 165 170 175 180
I W E L Q G G T I G K A Y G L Q T K E E E Q E F I
TP84_30

aaaacacattcggtgatggcgaataaaaaaattcgccatttttctgtggacttgttgacatcgtcaacaaaaaa
tttgtgtaagccactaccgcttatttttttaagcggtaaaaagacacctgaacaactgtagcagttgtttttt 25 950

185 190
K N T F G D G E *
TP84_30

rho-independent terminator

tggtacattataagtgtaaggcaatcacgaaaattcaaggtgaagcgatatgaaaaatgggatcaagtggtgatg
accatgtaatattcacattccgttagtgcttttaagttccacttcgctatactttttaccctagttcaccactac 26 025

1
M
TP84_31

aaaaaggcggaagccaacaaactggggatctttccagaactgcaagtgatcgaggattcgggcgaattcctcaca
tttttccgccttcggttgtttgacccctagaaaggtcttgacgttcactagctcctaagcccgcttaaggagtgt 26 100

5 10 15 20 25
K K A E A N K L G I F P E L Q V I E D S G E F L T
TP84_31

gtgtacgcgacaacgaaagatttaatacgaaaatgatcatgatcgaacgcgatcaactgcttaaaaagtaccaa
cacatgcgctgttgctttctaaattatgcttttactagtactagcttgcgctagttgacgaatttttcatggtt 26 175

30 35 40 45 50
V Y A T T K D F N T K M I M I E R D Q L L K K Y Q
TP84_31

taagatccgggccgctgatggcggcctggcacaaataaatcaaacaggggggaatgaacgatggaaatcaaaacac
attctaggcccggcgactaccgccggaccgtgtttatttagtttgtccccccttacttgctacctttagttttgtg 26 250

*
TP84_31

1 5
M E I K T
TP84_32

tgccgatcaagtggtctgtgccgactaattacatcacaggggttgtgatcgaaacaagaacgatgatttatgtga
acggctagttcaccagacacggctgattaatgtagtgtccccaacactagctttgttcttgctactaaatacact 26 325

10 15 20 25 30
L P I K W S V P T N Y I T G V V I E T R T M I Y V
TP84_32

tccgcaaaaacggatctgatgaattccaggtcgatcgaatggataaaaaccacaaatggaatcgatactggacaa
aggcgtttttgcctagactacttaaggtccagctagcttacctatttttggtgtttaccttagctatgacctgtt 26 400

35 40 45 50 55
I R K N G S D E F Q V D R M D K N H K W N R Y W T
TP84_32

ttgatcgattcacgatgaaaggatcagccggcttgccgaaagcggtgatcgaagcgatccaaacgatcaaagaaa
aactagctaagtgctactttcctagtcggccgaacggctttcgccactagcttcgctaggtttgctagtttcttt 26 475

60 65 70 75 80
I D R F T M K G S A G L P K A V I E A I Q T I K E
TP84_32

```
aaggcatcctgtgaaaagccgaaacggtctggggattcctggaccgtctggccagatgggatctggtcactgatg    26 550
ttccgtaggacacttttcggctttgccagaccccctaaggacctggcagaccggtctaccctagaccagtgactac
         85
K  G  I  L  *
TP84_32

atggcaaccatcacaggccaaaaaaatcaaacaggggggaatgaaggatgttcaaaactgaaaaacaaatcaatga    26 625
taccgttggtagtgtccggtttttttagtttgtccccccttacttcctacaagttttgacttttgtttagttact
                                                   1           5              10
                                                   M  F  K  T  E  K  Q  I  N  E
                                                   TP84_33

agcgaaagccgcattgaaaaaaggtgtgatcgtctattacttcaatgaagcggcgatgaatgcacacatcgggat     26 700
tcgctttcggcgtaacttttttccacactagcagataatgaagttacttcgccgctacttacgtgtgtagccccta
            15             20             25             30             35
 A  K  A  A  L  K  K  G  V  I  V  Y  Y  F  N  E  A  A  M  N  A  H  I  G  I
TP84_33

cgtggtcgatactgatccagaatattattccggcgaaggcgtatcagtcatgctagtcgatcgaaacttgaaacc     26 775
gcaccagctatgactaggtcttataataaggccgcttccgcatagtcagtacgatcagctagctttgaactttgg
            40             45             50             55             60
 V  V  D  T  D  P  E  Y  Y  S  G  E  G  V  S  V  M  L  V  D  R  N  L  K  P
TP84_33

aaaacaagatgtgttcggccggatcatattcgacatcgtgttttaccacaacgaaatatatcggatctgggaaac     26 850
ttttgttctacacaagccggcctagtataagctgtagcacaaaatggtgttgctttatatagcctagaccctttg
            65             70             75             80             85
 K  Q  D  V  F  G  R  I  I  F  D  I  V  F  Y  H  N  E  I  Y  R  I  W  E  T
TP84_33

agtcggcgaatttttttgcaggggaggaaggaaaatgaaacgattcgaaatcatgttgaaagacggattggtgtat    26 925
tcagccgcttaaaaaaacgtcccctccttccttttactttgctaagctttagtacaactttctgcctaaccacata
            90             95
 V  G  E  F  F  A  G  E  E  G  K  *
TP84_33
                    1           5              10
                    M  K  R  F  E  I  M  L  K  D  G  L  V  Y
                    TP84_34

gcagtttttccgatattgaatccaaactatcaaggcaacggcattgacgaaatcatcgaagctgaaagttacgat     27 000
cgtcaaaaaggctataacttaggtttgatagttccgttgccgtaactgctttagtagcttcgactttcaatgcta
15             20             25             30             35
A  V  F  P  I  L  N  P  N  Y  Q  G  N  G  I  D  E  I  I  E  A  E  S  Y  D
TP84_34

gacgcagtccaaaaagcggaaatgaaatatggcggatggtatcaatcgccaggatcgcaaaacgaatcaacattc     27 075
ctgcgtcaggtttttcgcctttactttataccgcctaccatagttagcggtcctagcgttttgcttagttgtaag
40             45             50             55             60
D  A  V  Q  K  A  E  M  K  Y  G  G  W  Y  Q  S  P  G  S  Q  N  E  S  T  F
TP84_34

aaagaggaagaaaacgcaagatattcgccggaacaagtttatggcatcaaaggtcttttatgaaaggggatcg      27 150
tttctccttcttttgcgttctataagcggccttgttcaaataccgtagtttccagaaaatacttttcccctagc
65             70             75             80             85
K  E  E  E  N  A  R  Y  S  P  E  Q  V  Y  G  I  K  G  L  L  *
TP84_34
                                                     1           5
                                                     M  K  R  G  I
                                                     TP84_35
```

atggtgatcacaaatccaaaatggaccgtcacagacgttctatgggccactctaggcgttttgatcatcggggca 27 225
taccactagtgtttaggttttacctggcagtgtctgcaagatacccggtgagatccgcaaaactagtagccccgt
10 15 20 25 30
D G D H K S K M D R H R R S M G H S R R F D H R G
TP84_35

atagtttatatcatgtcggtctgaaaagccgaaacaggaccgctttaaacggtcctgtccggccagatgggatct 27 300
tatcaaatatagtacagccagacttttcggctttgtcctggcgaaatttgccaggacaggccggtctaccctaga
35 40 45 50 55
N S L Y H V G L K S R N R T A L N G P V R P D G I
TP84_35

ggtcgctgacgatggcaaccgtcagaccaaaaatcgaatagggggaattcagatggtgaaacaaatgacagatgc 27 375
ccagcgactgctaccgttggcagtctggtttttagcttatcccccttaagtctaccactttgtttactgtctacg
60 65 — 1 5
W S L T M A T V R P K I E * — M V K Q M T D A
TP84_35 — TP84_36

acaaagaaaggcgatcgaatgggcggtcaaaatggcggaaagccagatcaaatcaagtcaaagacggataaaaag 27 450
tgtttctttccgctagcttacccgccagttttaccgcctttcggtctagtttagttcagtttctgcctatttttc
10 15 20 25 30
Q R K A I E W A V K M A E S Q I K S S Q R R I K R
TP84_36

ggcggaacgatcactggaattgttcaagggcatggatgaattagatgataagctgaaaagacgacaaatcaaaca 27 525
ccgccttgctagtgaccttaacaagttcccgtacctacttaatctactattcgacttttctgctgtttagtttgt
35 40 45 50 55
A E R S L E L F K G M D E L D D K L K R R Q I K H
TP84_36

tcaacaaaatcggatcgaaagtctgcaaaactacatcgatgcacttaaaagtctgcttgattgacgaaagccgaa 27 600
agttgttttagcctagctttcagacgttttgatgtagctacgtgaattttcagacgaactaactgctttcggctt
60 65 70 75
Q Q N R I E S L Q N Y I D A L K S L L D *
TP84_36

acagtccaggatcatccctggactgtctggcgtgatgggatcacgtcactgacgatggcaaccgcgtcagatcaa 27 675
tgtcaggtcctagtagggacctgacagaccgcactaccctagtgcagtgactgctaccgttggcgcagtctagtt

aaaacacgaaaggggatcgaaaatgcaagtcagaaaaatccacaaagtcacgtatgaatcagaaacaatgccgct 27 750
ttttgtgctttcccctagcttttacgttcagtctttttaggtgtttcagtgcatacttagtctttgttacggcga
1 5 10 15
M Q V R K I H K V T Y E S E T M P L
TP84_37

ggaacaagcggtggaagaaatcgcgcaaagcactggatcgcctgtggaccgtgttcgcggactgatcattcaatt 27 825
ccttgttcgccaccttctttagcgcgtttcgtgacctagcggacacctggcacaagcgcctgactagtaagttaa
20 25 30 35 40
E Q A V E E I A Q S T G S P V D R V R G L I I Q F
TP84_37

cggcaaagtgatgacagtccatcatgtctatcacatcgtggatgccagtgggatcgggctggtgaaggatgcaga 27 900
gccgtttcactactgtcaggtagtacagatagtgtagcacctacggtcaccctagcccgaccacttcctacgtct
45 50 55 60 65
G K V M T V H H V Y H I V D A S G I G L V K D A E
TP84_37

aaaattcctgggccaatcgatggtgtttcaactgaaaggcggcgaaaccgtcattttgaaagtcatggaagtgac 27 975
ttttaaggacccggttagctaccacaaagttgactttccgccgctttggcagtaaaactttcagtaccttcactg

70 75 80 85 90
K F L G Q S M V F Q L K G G E T V I L K V M E V T
TP84_37

acgatggatgaatggggattattttatcaaaggatatgatgatgaagggatgtggcgcacgatcgatgtcgattc 28 050
tgctacctacttaccccctaataaaatagtttcctatactactacttccctacaccgcgtgctagctacagctaag

95 100 105 110 115
R W M N G D Y F I K G Y D D E G M W R T I D V D S
TP84_37

gattaaagcatatttcacgccaggaaaggggatgatccgatgaaaccatatgatccgatcccgatggcgatcaca 28 125
ctaatttcgtataaagtgcggtcctttcccctactaggctactttggtatactaggctagggctaccgctagtgt

120 125 130
I K A Y F T P G K G M I R *
TP84_37

1 5 10
M K P Y D P I P M A I T
TP84_38

ttgaaaccacaccaaacgatccgaaaaggtgaagtcatccaggaaaccatcggtggcaaagtggtcaaatttaaa 28 200
aactttggtgtggtttgctaggcttttccacttcagtaggtcctttggtagccaccgtttcaccagtttaaattt

15 20 25 30 35
L K P H Q T I R K G E V I Q E T I G G K V V K F K
TP84_38

gtgatcgacatccgaatggtggaaatacgcgaaacacacgcactggtcggtgtaatggccagggaaatcggggaa 28 275
cactagctgtaggcttaccacctttatgcgctttgtgtgcgtgaccagccacattaccggtccctttagcccctt

40 45 50 55 60
V I D I R M V E I R E T H A L V G V M A R E I G E
TP84_38

ggtgatgccgaataaatgaaaaagaccaggcgatctgcctggccaccaaacaggggaattcaatggtgaattcc 28 350
ccactacggcttatttactttttctggtccgctagacggaccggtggtttgtccccttaagttaccacttaagg

65
G D A E *
TP84_38

tctatattataccaagaaacacgatattcaacaacatgaggaggtggacaaaatggataaaattccgaaactagt 28 425
agatataatatggttctttgtgctataagttgttgtactcctccacctgttttacctattttaaggctttgatca

1 5
M D K I P K L V
TP84_39

gatggcatcggatatggctgaaaaatggggtgtggatcgccgtgtggtgaataactggtccaggcgtgatccgtc 28 500
ctaccgtagcctataccgactttttaccccacacctagcggcacaccacttattgaccaggtccgcactaggcag

10 15 20 25 30
M A S D M A E K W G V D R R V V N N W S R R D P S
TP84_39

attcccgaaaccagtgatggttgtcggcaacgggaaatatccgctttacctggaagaagatatgatcgcttatgg 28 575
taagggctttggtcactaccaacagccgttgccctttataggcgaaatggaccttcttctatactagcgaatacc

35 40 45 50 55
F P K P V M V V G N G K Y P L Y L E E D M I A Y G
TP84_39

```
caaacaaaaaggtctggaaaaaggggatgatacacaaacatgatcagatcgaaggattactgggaaggatacaat   28 650
gtttgtttttccagacctttttcccctactatgtgtttgtactagtctagcttcctaatgacccttcctatgtta
 60 65 70
K Q K G L E K G D D T Q T *
TP84_39
 1 5 10
M I R S K D Y W E G Y N
TP84_40
```

```
cacgcacagaaggaagcggaaaagcggtgggaacaagcacggtctgaactcaccgccagatttattgagatcatc   28 725
gtgcgtgtcttccttcgccttttcgccacccttgttcgtgccagacttgagtggcggtctaaataactctagtag
 15 20 25 30 35
H A Q K E A E K R W E Q A R S E L T A R F I E I I
TP84_40
```

```
gaagattccaggcgtgacgtgaaggggatcgggccaaaagtacacaaagcactggtcgaatacgtgatcgaacga   28 800
cttctaaggtccgcactgcacttcccctagcccggttttcatgtgtttcgtgaccagcttatgcactagcttgct
 40 45 50 55 60
E D S R R D V K G I G P K V H K A L V E Y V I E R
TP84_40
```

```
atgatgaatggggggcggaaataatgtggctaggattcgaaacacggcaacaatatctggactggcttgaatcgt   28 875
tactacttaccccccgcctttattacaccgatcctaagctttgtgccgttgttatagacctgaccgaacttagca
 1 5 10 15
M W L G F E T R Q Q Y L D W L E S
TP84_41
 65 70
M M N G G R K *
TP84_40
```

```
tgcaaccaggaaacgaagtggcagtgggattccgcattcgatatggaagggatgtgcgttatgagatccgaacag   28 950
acgttggtcctttgcttcaccgtcaccctaaggcgtaagctataccttccctacacgcaatactctaggcttgtc
 20 25 30 35 40
L Q P G N E V A V G F R I R Y G R D V R Y E I R T
TP84_41
```

```
tcacaagggtgacaaatcggcaaatcatcctggatgatggtgcaatccgcttcaatcgcggcacagggaagcagt   29 025
agtgttcccactgtttagccgtttagtaggacctactaccacgttaggcgaagttagcgccgtgtcccttcgtca
 45 50 55 60 65
V T R V T N R Q I I L D D G A I R F N R G T G K Q
TP84_41
```

```
ggggcggatatgcatggatcgagccagtcaccgaaaaaattctatcgcaaaaatacggtaaacaggcataattcc   29 100
ccccgcctatacgtacctagctcggtcagtggcttttttaagatagcgtttttatgccatttgtccgtattaagg
 70 75 80 85 90
W G G Y A W I E P V T E K I L S Q K Y G K Q A *
TP84_41
```

```
gctgatccctgcatacactggattcatccagtgtttgtggggatactctacacaccggggggattcagatggcat   29 175
cgactagggacgtatgtgacctaagtaggtcacaaacacccctatgagatgtgtggcccccctaagtctaccgta
 1
M A
TP84_42
```

```
tgaaagtggttaaaaagcagatgacatgcaaagagatcatgatcctttatttctgcgatgactgcggcatccagg   29 250
actttcaccaatttttcgtctactgtacgtttctctagtactaggaaataaagacgctactgacgccgtaggtcc
 5 10 15 20 25
L K V V K K Q M T C K E I M I L Y F C D D C G I Q
TP84_42
```

aatggtcggatcggatcattccgaaaaagcgatgcgaaaagtgtggactgtttatggatcacgaagaatgggaag 29 325
ttaccagcctagcctagtaaggctttttcgctacgcttttcacacctgacaaatacctagtgcttcttacccttc
30 35 40 45 50
E W S D R I I P K K R C E K C G L F M D H E E W E
TP84_42

ggtgatggtgaatgcatatcacaaaaaaactgatcccggccgatcaatccgaaatatatcaaatcccaaagcatc 29 400
ccactaccacttacgtatagtgtttttttgactagggccggctagttaggctttatatagtttagggtttcgtag
1 5 10 15 20
M V N A Y H K K T D P G R S I R N I S N P K A S
TP84_43

G *
TP84_42

acgcactggatgtcctggcgaaagagatcaccaaacaacaaagacacatcgaacggatcaagatggacatccagg 29 475
tgcgtgacctacaggaccgctttctctagtggtttgttgtttctgtgtagcttgcctagttctacctgtaggtcc
25 30 35 40 45
R T G C P G E R D H Q T T K T H R T D Q D G H P G
TP84_43

cggtgaaggatcagccgaaaaaggtggcacgatggcaaaaacagatcgagattcacgaaaagcgaatccaggaat 29 550
gccacttcctagtcggctttttccaccgtgctaccgtttttgtctagctctaagtgcttttcgcttaggtcctta
50 55 60 65 70
G E G S A E K G G T M A K T D R D S R K A N P G M
TP84_43

gtcgccaggcgatcaaatggatcatggaaaatctggtgtaaaggcactgaaaaaaatcagtgcctttttttatttt 29 625
cagcggtccgctagtttacctagtacctttagaccacatttccgtgactttttttagtcacggaaaaaaataaaa
75 80 85 90 95
S P G D Q M D H G K S G V K A L K K I S A F F I F
TP84_43
rho-independent terminator

ttatggaatatagacaggcacacatgaaatatgtatgtaatgcaaagcaaatcaaaactaaatgaaaaggggatg 29 700
aataccttatatctgtccgtgtgtactttatacatacattacgtttcgtttagttttgatttacttttcccctac
100
Y G I *
TP84_43

rho-independent terminator

gaatgacatgaaaaaaatgaaaaaatacactgtggtgatcagaaaatttcaggcagaaaggaaaaatggggatt 29 775
cttactgtactttttttacttttttatgtgacaccactagtcttttaaagtccgtctttcctttttacccctaa
1 5 10 15 20
M K K M K K Y T V V I R K F S G R K E K W G F
TP84_44

tttagcgcagaacgaacagatcgcaaaagctattgcagatgcaatcagaaagggtggcaagtaatgtcaatgaaa 29 850
aaatcgcgtcttgcttgtctagcgttttcgataacgtctacgttagtctttcccaccgttcattacagttacttt
1
M S M K
TP84_45

25 30 35 40
L A Q N E Q I A K A I A D A I R K G G K *
TP84_44

acgaaaggggtggcattcaatctggccgatcccgatcaaaaggcattatatgaatatgccatgacacgcaccaat 29 925
tgctttccccaccgtaagttagaccggctagggctagttttccgtaatatacttatacggtactgtgcgtggtta
5 10 15 20 25
T K G V A F N L A D P D Q K A L Y E Y A M T R T N
TP84_45

ttcagcgcatacgtcaaacggctgatccaggctgatatgcaacggcaacaagcaaaaaccgcagggcgacaacat 30 000
aagtcgcgtatgcagtttgccgactaggtccgactatacgttgccgttgttcgtttttggcgtcccgctgttgta
30 35 40 45 50
F S A Y V K R L I Q A D M Q R Q Q A K T A G R Q H
TP84_45

cctgcggtccattccgctgaatatggcggcattaagatccgaattggcgaaaagtcactgggtgaatagcttgac 30 075
ggacgccaggtaaggcgacttataccgccgtaattctaggcttaaccgcttttcagtgacccacttatcgaactg
55 60 65 70 75 1
P A V H S A E Y G G I K I R I G E K S L G E * M T
TP84_45 TP84_46

gatgatggccgccgtgccagtggtcacaagcacatatccgattccgacaaggatcggcatcgatgatcacatcct 30 150
ctactaccggcggcacggtcaccagtgttcgtgtataggctaaggctgttcctagccgtagctactagtgtagga
5 10 15 20 25
M M A A V P V V T S T Y P I P T R I G I D D H I L
TP84_46

tttccaaagtgtcgatcgtgtttatagtatgaacaaaaaacgaaagggtgatacatgtgttcacacgaaaaaagg 30 225
aaaggtttcacagctagcacaaatatcatacttgttttttgctttcccactatgtacacaagtgtgctttttttcc
30 35 40 45 50
F Q S V D R V Y S M N K K R K G D T C V H T K K G
TP84_46

tggaaaccattccatttcgcgaattcatggatggcaactggcgcgaaaagaaggctgaacgccgcaaagaaagcc 30 300
acctttggtaaggtaaagcgcttaagtacctaccgttgaccgcgcttttcttccgacttgcggcgtttctttcgg
1 5 10 15
M D G N W R E K K A E R R K E S
TP84_47
55 60 65 70
G N H S I S R I H G W Q L A R K E G *
TP84_46

tggatgaagtgatcaccagactggcggcggttggaactggcgcacatatggtgatccatccgatcgatgcacacg 30 375
acctacttcactagtggtctgaccgccgccaaccttgaccgcgtgtataccactaggtaggctagctacgtgtgc
20 25 30 35 40
L D E V I T R L A A V G T G A H M V I H P I D A H
TP84_47

ccgctgacatgggcgacatcattatgaaggcgtgccaaccgatcattgatttattgcaaggcatttcgtatccag 30 450
ggcgactgtacccgctgtagtaatacttccgcacggttggctagtaactaaataacgttccgtaaagcataggtc
45 50 55 60 65
A A D M G D I I M K A C Q P I I D L L Q G I S Y P
TP84_47

tcgctttcatcatgatcactggcggattcctgctgatcatgactgggcagacatccaggggggatgcatttcatca 30 525
agcgaaagtagtactagtgaccgcctaaggacgactagtactgacccgtctgtaggtcccccctacgtaaagtagt
70 75 80 85 90
V A F I M I T G G F L L I M T G Q T S R G M H F I
TP84_47

```
aatgggcgtgcctgggatatttaggattgcaatttgcgcctgcactgatgcagattgtgatccagatcggtcaaa   30 600
ttacccgcacggaccctataaatcctaacgttaaacgcggacgtgactacgtctaacactaggtctagccagttt
1   5   10   15   20   25
M G V P G I F R I A I C A C T D A D C D P D R S K
TP84_48
95   100   105   110   115
K W A C L G Y L G L Q F A P A L M Q I V I Q I G Q
TP84_47

acattcaagcacaagtgggggtggtacgatgaagcatttccgcattatcccggatcaaaagattgacaaccgtca   30 675
tgtaagttcgtgttcacccccaccatgctacttcgtaaaggcgtaatagggcctagttttctaactgttggcagt
30   35   40   45   50
H S S T S G G G T M K H F R I I P D Q K I D N R H
TP84_48
120   125
N I Q A Q V G V V R *
TP84_47

tgtggatcagatgctaaagacgctgaacatatataaaagtccaatacagcggtggaatggtcggggattcgatcg   30 750
acacctagtctacgatttctgcgacttgtatatattttcaggttatgtcgccaccttaccagcccctaagctagc
55   60   65   70   75
V D Q M L K T L N I Y K S P I Q R W N G R G F D R
TP84_48

aatcccttttatttcatacgaaatacacatccaaaaggatcacaccacattccacatgacggtggccgatgaaat   30 825
ttagggaaaataaagtatgctttatgtgtaggttttcctagtgtggtgtaaggtgtactgccaccggctacttta
80   85   90   95   100
I P F I S Y E I H I Q K D H T T F H M T V A D E I
TP84_48

cgaaacgatcgcgaaaaaagcactggaaaccgcctggccgaatgcatcgatcgatctggccgctgatccattcca   30 900
gctttgctagcgctttttttcgtgacctttggcggaccggcttacgtagctagctagaccggcgactaggtaaggt
105   110   115   120   125
E T I A K K A L E T A W P N A S I D L A A D P F Q
TP84_48

gggattcgaaccgaacgcaatcagccgtctggaattgaaacatcattccatgtttgccattcgggtggatcgccg   30 975
ccctaagcttggcttgcgttagtcggcagaccttaactttgtagtaaggtacaaacggtaagcccacctagcggc
130   135   140   145   150
G F E P N A I S R L E L K H H S M F A I R V D R R
TP84_48

ggaaaatggattcatgcacaatttgttcgaatcgttgaaggtgatgggccaggatgatcggatcttcattcagat   31 050
cctttttacctaagtacgtgttaaacaagcttagcaacttccactacccggtcctactagcctagaagtaagtcta
155   160   165   170   175
E N G F M H N L F E S L K V M G Q D D R I F I Q I
TP84_48

cctttgcatccctgcatccctggactggtacacatccgccgctgatgcctacaaacgttttaaggaaggcaagat   31 125
ggaaacgtagggacgtagggacctgaccatgtgtaggcggcgactacggatgtttgcaaaattccttccgttcta
180   185   190   195   200
L C I P A S L D W Y T S A A D A Y K R F K E G K M
TP84_48

gccgaaaaagattcacatcgatggcaaagaactggccagggaagggatcaaactggtgacacgtggtgtcctggg   31 200
cggctttttctaagtgtagctaccgtttcttgaccggtcccttccctagtttgaccactgtgcaccacaggaccc
205   210   215   220   225
P K K I H I D G K E L A R E G I K L V T R G V L G
TP84_48
```

cgtgatcgacacgattgtggtcctaacaggcggcacaccagaaaagatcaatcttgacaatgcagatcgggccat 31 275
gcactagctgtgctaacaccaggattgtccgccgtgtggtcttttctagttagaactgttacgtctagcccggta
230 235 240 245 250
V I D T I V V L T G G T P E K I N L D N A D R A M
TP84_48

gatgatgcgtgatggtggcctgcgactggaaacgatcaacaagacaaaacacgatgcgttcgatgtcacgatccg 31 350
ctactacgcactaccaccggacgctgacctttgctagttgttctgttttgtgctacgcaagctacagtgctaggc
255 260 265 270 275
M M R D G G L R L E T I N K T K H D A F D V T I R
TP84_48

tgtggcgatccagtcaaagaatcacgaaaacatcatgaaaatggtggccgcttcattccgtgaattcgatggcga 31 425
acaccgctaggtcagtttcttagtgcttttgtagtactttaccaccggcgaagtaaggcacttaagctaccgct
280 285 290 295 300
V A I Q S K N H E N I M K M V A A S F R E F D G D
TP84_48

caatcaattcacatattatccactggatgtgaataagatgacgatcggatggatcgccgatcggaaaccagggat 31 500
gttagttaagtgtataataggtgacctacacttattctactgctagcctacctagcggctagcctttggtcccta
305 310 315 320 325
N Q F T Y Y P L D V N K M T I G W I A D R K P G I
TP84_48

caagctgtcaaaggactatctatcgacaccagaactggccaggatcattcatatgccgactggaccgatccagat 31 575
gttcgacagtttcctgatagatagctgtggtcttgaccggtcctagtaagtatacggctgacctggctaggtcta
330 335 340 345 350
K L S K D Y L S T P E L A R I I H M P T G P I Q M
TP84_48

gaaatggaacatcgaacggatcgagaaaacacagacagccgtgccatccaggatcacgactggtcctggcatctt 31 650
ctttaccttgtagcttgcctagctcttttgtgtctgtcggcacggtaggtcctagtgctgaccaggaccgtagaa
355 360 365 370 375
K W N I E R I E K T Q T A V P S R I T T G P G I L
TP84_48

gatcggtgtgcatacacacaaaggagtggaacaaacggtgttcatgccgatcgatgactgggatgagttgtgtct 31 725
ctagccacacgtatgtgtgtttcctcaccttgtttgccacaagtacggctagctactgaccctactcaacacaga
380 385 390 395 400
I G V H T H K G V E Q T V F M P I D D W D E L C L
TP84_48

gccgcgtgtggcgatcggcggcatgggccagggcaagacaagaggattcggcgcaaactggttatatcaagcggt 31 800
cggcgcacaccgctagccgccgtacccggtcccgttctgttctcctaagccgcgtttgaccaatatagttcgcca
405 410 415 420 425
P R V A I G G M G Q G K T R G F G A N W L Y Q A V
TP84_48

caaaaatggattcggcggcctggcgatcgatccggcaaaaggggaaatcggcaacgaactggcggcggtcctgga 31 875
gtttttacctaagccgccggaccgctagctaggccgtttttcccctttagccgttgcttgaccgccgccaggacct
430 435 440 445 450
K N G F G G L A I D P A K G E I G N E L A A V L D
TP84_48

tgaagatgaattcattcggatcaacatcgcgcaaaatccgatctgcctggactggtgtgaaacaaaatacagtga 31 950
acttctacttaagtaagcctagttgtagcgcgttttaggctagacggacctgaccacactttgttttatgtcact
455 460 465 470 475
E D E F I R I N I A Q N P I C L D W C E T K Y S E
TP84_48

```
actggccaggggcagactggcaaatacgatgatcagctttttcaattcgaatgccgatgatgcaggagtgcagac   32 025
tgaccggtccccgtctgaccgtttatgctactagtcgaaaaagttaagcttacggctactacgtcctcacgtctg
        480        485        490        495        500
 L  A  R  G  R  L  A  N  T  M  I  S  F  F  N  S  N  A  D  D  A  G  V  Q  T
TP84_48 >

acagcgatatatccgggcgatggttatggggatgcaggggaacaaactggatgaattgatccgcatgatgaatga   32 100
tgtcgctatataggcccgctaccaatacccctacgtccccttgtttgacctacttaactaggcgtactacttact
        505        510        515        520        525
 Q  R  Y  I  R  A  M  V  M  G  M  Q  G  N  K  L  D  E  L  I  R  M  M  N  D
TP84_48 >

tatggcctatttgcaaaaatgcgtggaaaagatgccgccaggattccaccgggccacactggaagaactgatcaa   32 175
ataccggataaacgtttttacgcaccttttctacggcggtcctaaggtggcccggtgtgaccttcttgactagtt
        530        535        540        545        550
 M  A  Y  L  Q  K  C  V  E  K  M  P  P  G  F  H  R  A  T  L  E  E  L  I  N
TP84_48 >

ctattccgatgccagacgaatgcagatcctatcgccgatcttgaatcgattagacatgatcctgggcgatccatt   32 250
gataaggctacggtctgcttacgtctaggatagcggctagaacttagctaatctgtactaggacccgctaggtaa
        555        560        565        570        575
 Y  S  D  A  R  R  M  Q  I  L  S  P  I  L  N  R  L  D  M  I  L  G  D  P  F
TP84_48 >

cctggcgaaatgcatggaatccgatcgatccctggacatggtggacattctgcggcaacgaaaagcgatcgtgat   32 325
ggaccgctttacgtaccttaggctagctagggacctgtaccacctgtaagacgccgttgcttttcgctagcacta
        580        585        590        595        600
 L  A  K  C  M  E  S  D  R  S  L  D  M  V  D  I  L  R  Q  R  K  A  I  V  I
TP84_48 >

cgatgtaccgaaaaaggatgtcggtccagaaggcgtggacatcattgtgaatcttctatccaccaaaatcgatct   32 400
gctacatggctttttcctacagccaggtcttccgcacctgtagtaacacttagaagataggtggttttagctaga
        605        610        615        620        625
 D  V  P  K  K  D  V  G  P  E  G  V  D  I  I  V  N  L  L  S  T  K  I  D  L
TP84_48 >

ggcgatgacactgcggccagacgatgaacaatttccgtttttcatcgtgttcgatgaaccgcaccaatacatgag   32 475
ccgctactgtgacgccggtctgctacttgttaaaggcaaaaagtagcacaagctacttggcgtggttatgtactc
        630        635        640        645        650
 A  M  T  L  R  P  D  D  E  Q  F  P  F  F  I  V  F  D  E  P  H  Q  Y  M  R
TP84_48 >

aagccacacgatctggaaatcagcatgtgtggaatccaggaaatggcgtgtcggctatgtctggatgtttcacga   32 550
ttcggtgtgctagacctttagtcgtacacaccttaggtcctttaccgcacagccgatacagacctacaaagtgct
        655        660        665        670        675
 S  H  T  I  W  K  S  A  C  V  E  S  R  K  W  R  V  G  Y  V  W  M  F  H  E
TP84_48 >

atggacacaaatcgatgacaaactgcggaagatcatgaaatccgcgttgccgcactatcacgtttatccatcgtc   32 625
tacctgtgtttagctactgtttgacgccttctagtactttaggcgcaacggcgtgatagtgcaaataggtagcag
        680        685        690        695        700
 W  T  Q  I  D  D  K  L  R  K  I  M  K  S  A  L  P  H  Y  H  V  Y  P  S  S
TP84_48 >

aaaaaacacattcctggatctgaaagaagaactgcaaccattcgaactggatgactttctaaaactggaacgctg   32 700
ttttttgtgtaaggacctagactttcttcttgacgttggtaagcttgacctactgaaagattttgaccttgcgac
        705        710        715        720        725
 K  N  T  F  L  D  L  K  E  E  L  Q  P  F  E  L  D  D  F  L  K  L  E  R  W
TP84_48 >
```

```
gcacgccatcaacgtgatccgatccggcggccaaacgatcacaccattcatcgccaggatgacaccgccgccaac   32 775
cgtgcggtagttgcactaggctaggccgccggtttgctagtgtggtaagtagcggtcctactgtggcggcggttg
    730 735 740 745 750
 H  A  I  N  V  I  R  S  G  G  Q  T  I  T  P  F  I  A  R  M  T  P  P  P  T
TP84_48 >

aaaaagagaaatctagttttttgttgtaatttgcataatttttgttacaattgaatgtgtaaaatattacaattc   32 850
tttttctctttagatcaaaaaacaacattaaacgtattaaaaacaatgttaacttacacattttataatgttaag
    755
 K  R  E  I  *
TP84_48 >

aaagaaagggtggacaatagatgaaaaaggtattaaaagctattttggccggggcgttgacattcggtgtgatgg   32 925
tttctttcccacctgttatctactttttccataattttcgataaaaccggccccgcaactgtaagccacactacc
    1 5 10 15
                    M  K  K  V  L  K  A  I  L  A  G  A  L  T  F  G  V  M
                    TP84_49 >

gaactgtgtttgcagtatcaccaaaaaccgaagtccaagcggctgtcgtggaagattggagcggatacaaagaaa   33 000
cttgacacaaacgtcatagtggtttttggcttcaggttcgccgacagcaccttctaacctcgcctatgtttcttt
    20 25 30 35 40
G  T  V  F  A  V  S  P  K  T  E  V  Q  A  A  V  V  E  D  W  S  G  Y  K  E
TP84_49 >

cagatctgcaattacctattgacgcaagtctcgctgacgctgatcgggattataattatggcgacacatacaatg   33 075
gtctagacgttaatggataactgcgttcagagcgactgcgactagccctaatattaataccgctgtgtatgttac
    45 50 55 60 65
T  D  L  Q  L  P  I  D  A  S  L  A  D  A  D  R  D  Y  N  Y  G  D  T  Y  N
TP84_49 >

tattaaaaacggtcgaatggcgtttttactccgaaacacattaaaatctatcgtgtgaacgaagataaaacgctac   33 150
ataatttttgccagcttaccgcaaaaatgaggctttgtgtaattttagatagcacacttgcttctattttgcgatg
    70 75 80 85 90
V  L  K  T  V  E  W  R  F  T  P  K  H  I  K  I  Y  R  V  N  E  D  K  T  L
TP84_49 >

aaagatataagactatttacccggaacttataaatgttatcgatggcatagaatatcagtatagattccagacac   33 225
tttctatattctgataaatgggccttgaatatttacaatagctaccgtatcttatagtcatatctaaggtctgtg
    95 100 105 110 115
Q  R  Y  K  T  I  Y  P  E  L  I  N  V  I  D  G  I  E  Y  Q  Y  R  F  Q  T
TP84_49 >

cgatcacgactggtttccctgctggaaactattatgcagtactaacatacacatatgaatatgcaggcggaaact   33 300
gctagtgctgaccaaagggacgacctttgataatacgtcatgattgtatgtgtatacttatacgtccgcctttga
    120 125 130 135 140
P  I  T  T  G  F  P  A  G  N  Y  Y  A  V  L  T  Y  T  Y  E  Y  A  G  G  N
TP84_49 >

tcgtgacagaagcctatcgctcgtatcgtttcacaatcaattgatactgaaacaattcgaaaggtcgttcttgga   33 375
agcactgtcttcggatagcgagcatagcaaagtgttagttaactatgactttgttaagctttccagcaagaacct
    145 150 155
F  V  T  E  A  Y  R  S  Y  R  F  T  I  N  *
TP84_49 >
                                                        rho-independent terminator

tcagacaggaacggccttttttgttgcccaataagtgccagtgacacacgtggtgcaaaagtgatatgatgggcg   33 450
agtctgtccttgccggaaaaaacaacgggttattcacggtcactgtgtgcaccacgttttcactatactacccgc
rho-independent terminator
```

```
tgagaaaggggaatgagtggtgaacaaaatcgatctgatcaaacaactactggaagcaggcaaactgccgatgc   33 525
actctttccccttactcaccacttgttttagctagactagtttgttgatgaccttcgtccgtttgacggctacg
          1   5   10   15
          M V N K I D L I K Q L L E A G K L P M
          TP84_50 >

ttgacaaagacaaagattatacgatccaggaatatatgaagctgaaacacgcatcgatcaaagcgatcgcagatg   33 600
aactgtttctgtttctaatatgctaggtccttatatacttcgactttgtgcgtagctagtttcgctagcgtctac
20   25   30   35   40
L D K D K D Y T I Q E Y M K L K H A S I K A I A D
TP84_50 >

cgcttgaacaagcatgccagggatgatccctggctttttattgcgtaaataccattatatgcagtgatcgaaaa   33 675
gcgaacttgttcgtacggtccctactagggaccgaaaaataacgcatttatggtaatatacgtcactagctttt
45   50
A L E Q A C Q G *
TP84_50
rho-indepenndent terminator

aatatcacaaacatgatcaaaaaaatattgcaaagtgatcatgatcatgataatataaagacgtggacatgatgg   33 750
ttatagtgtttgtactagtttttttataacgtttcactagtactagtactattatatttctgcacctgtactacc

acatgtccgccgcccatcaaagggtgtggccgtcatatggactagaaaaggcggtcaatataaaaagatcggggg   33 825
tgtacaggcggcgggtagtttcccacaccggcagtatacctgatcttttccgccagttatatttttctagccccc

gattcaatggggaaaaggttgcaaaaactaatcgaacaacaacgaaagaaaatgatcgatctgggaatgacgaaa   33 900
ctaagttacccctttttccaacgtttttgattagcttgttgttgctttcttttactagctagacccttactgcttt
  1   5   10   15   20
  M G K R L Q K L I E Q Q R K K M I D L G M T K
  TP84_51 >

ggattcaatcatccagatacgatccgggcttcacaagaattggacaagctaatcaacaaagcaatgaaaaagcaa   33 975
cctaagttagtaggtctatgctaggcccgaagtgttcttaacctgttcgattagttgtttcgttactttttcgtt
  25   30   35   40   45
G F N H P D T I R A S Q E L D K L I N K A M K K Q
TP84_51 >

aagccagggaaatgatccctggcacactacacaacaagaaggggtgatcaccaagtgtctatcgctgaaaattta   34 050
ttcggtccctttactagggaccgtgtgatgtgttgttcttccccactagtggttcacagatagcgacttttaaat
  50                         1   5
K P G K *                    M S I A E N L
TP84_51                      TP84_52 >

aagcggcacagggaagcgaaaaatctgtcgcaacgaaagctggctgaattggctggcgtgccgcaatcattgatc   34 125
ttcgccgtgtcccttcgctttttagacagcgttgctttcgaccgacttaaccgaccgcacggcgttagtaactag
    10   15   20   25   30
K R H R E A K N L S Q R K L A E L A G V P Q S L I
TP84_52 >

agtcacatcgaaaatggcgaaaagaaaaatccaggtgtgatcggcattaaaaaactggccgatgcactggggatc   34 200
tcagtgtagcttttaccgcttttctttttaggtccacactagccgtaatttttttgaccggctacgtgacccctag
    35   40   45   50   55
S H I E N G E K K N P G V I G I K K L A D A L G I
TP84_52 >
```

```
acaatggaagaactgatcgaaagcaagggggatgaggatgaggaaatgggcggtcttgaccgatgatctaaaatt   34 275
tgttaccttcttgactagctttcgttccccctactcctactcctttacccgccagaactggctactagattttaa
                                             1       5         10
                                             M R K W A V L T D D L K F
                                             TP84_53 >
    60        65        70        75
T M E E L I E S K G D E D E E M G G L D R *
TP84_52 >

ttgcgaaatcacattcgatggcgaatggtatcgtgtaagaatcaccgatgaattcggtcaggaattaatgacaaa   34 350
aacgctttagtgtaagctaccgcttaccatagcacattcttagtggctacttaagccagtccttaattactgttt
  15        20        25        30        35
C E I T F D G E W Y R V R I T D E F G Q E L M T N
TP84_53 >

cgaatcgatcagcttacaaggggcgaaaaaacaagtcaaacgattcatcggcactggccaaacgttgaaatggca   34 425
gcttagctagtcgaatgttccccgcttttttgttcagtttgctaagtagccgtgaccggtttgcaactttaccgt
  40        45        50        55        60
E S I S L Q G A K K Q V K R F I G T G Q T L K W Q
TP84_53 >

ggaagtcgaaaccgacaagatcgaaacaccatccaaactgcatctgccattcggttatctgtctgtttcacaagt   34 500
ccttcagctttggctgttctagctttgtggtaggtttgacgtagacggtaagccaatagacagacaaagtgttca
  65        70        75        80        85
E V E T D K I E T P S K L H L P F G Y L S V S Q V
TP84_53 >

tagaaaatatctgacatgtccacgtgcttatgaattcaaatatgtcaacaaactgaatgagccgatcggatcgac   34 575
atcttttatagactgtacaggtgcacgaatacttaagtttatacagttgtttgacttactcggctagcctagctg
  90        95        100       105       110
R K Y L T C P R A Y E F K Y V N K L N E P I G S T
TP84_53 >

actggtgatgggccgggcattccacaaagggatgcagatggcatcgatcaaaaaggtggtcgatggcgaaatctt   34 650
tgaccactacccggcccgtaaggtgtttccctacgtctaccgtagctagtttttccaccagctaccgctttagaa
  115       120       125       130       135
L V M G R A F H K G M Q M A S I K K V V D G E I L
TP84_53 >

atccactgacgatgtgctggatgtatattcggatgcgttcgatcaagaacgtgaaaacaatgatgtggactgggc   34 725
taggtgactgctacacgacctacatataagcctacgcaagctagttcttgcacttttgttactacacctgacccg
  140       145       150       155       160
S T D D V L D V Y S D A F D Q E R E N N D V D W A
TP84_53 >

cgaagatgatccggcaaaggtgaaagatgatggcgcgaagctgatgcagaaatactatgaggaaatggggatcaa   34 800
gcttctactaggccgtttccactttctactaccgcgcttcgactacgtctttatgatactcctttacccctagtt
  165       170       175       180       185
E D D P A K V K D D G A K L M Q K Y Y E E M G I N
TP84_53 >

tgcgattccgatggtcgatgatcgtggcctgccgcttgtcgaacgtgaacacgcattcgagatcgtgccagggct   34 875
acgctaaggctaccagctactagcaccggacggcgaacagcttgcacttgtgcgtaagctctagcacggtcccga
  190       195       200       205       210
A I P M V D D R G L P L V E R E H A F E I V P G L
TP84_53 >
```

```
gaaagcgaaagcagtcatcgacatcatcgaacaagacggatcaatccgggattacaaaacaagtaagcgatcgcc   34 950
ctttcgctttcgtcagtagctgtagtagcttgttctgcctagttaggccctaatgttttgttcattcgctagcgg
    215       220       225       230       235
K A K A V I D I I E Q D G S I R D Y K T S K R S P
TP84_53

atcgcaaacgatcatcgatgaaacgatccagatgccagtctatgcgcttgcatatcgtgacatcactggccaggt   35 025
tagcgtttgctagtagctactttgctaggtctacggtcagatacgcgaacgtatagcactgtagtgaccggtcca
    240       245       250       255       260
S Q T I I D E T I Q M P V Y A L A Y R D I T G Q V
TP84_53

cgaaaaaacggtgggcctggattatgcagtgaacctaaagaaagaaaaaaagatcatgcgcctggaaaccgatgg   35 100
gcttttttgccacccggacctaatacgtcacttggatttctttctttttttctagtacgcggacctttggctacc
    265       270       275       280       285
E K T V G L D Y A V N L K K E K K I M R L E T D G
TP84_53

gccagtcgatgatggccgcatcgaacgtgtaaaacaaaccttcgtgggtgtggcaaaggcgatcagtgccgggat   35 175
cggtcagctactaccggcgtagcttgcacattttgtttggaagcacccacaccgtttccgctagtcacggcccta
    290       295       300       305       310
P V D D G R I E R V K Q T F V G V A K A I S A G I
TP84_53

cttttacccgaatgaagaatcgaacgcctgcggctattgttcatttaaggacatctgcaaaaaatcaaaaacatt   35 250
gaaaatgggcttacttcttagcttgcggacgccgataacaagtaaattcctgtagacgttttttagtttttgtaa
    315       320       325       330       335
F Y P N E E S N A C G Y C S F K D I C K K S K T F
TP84_53

ttaagaaaggggaattcaaatgggattcgatatgcaatatttcagcgaaaaagaagcacagcaatatcaagatgc   35 325
aattctttccccttaagtttaccctaagctatacgttataaagtcgctttttcttcgtgtcgttatagttctacg
                1       5         10        15
*               M G F D M Q Y F S E K E A Q Q Y Q D A
TP84_53         TP84_54

agtggaaaaactcactgaacaaaacacgatccaggtggtttcacttaacgacatcaaacaaaaactactggaaga   35 400
tcacctttttgagtgacttgttttgtgctaggtccaccaaagtgaattgctgtagtttgtttttgatgaccttct
20        25        30        35        40
V E K L T E Q N T I Q V V S L N D I K Q K L L E E
TP84_54

agtgaaggaattcgaattcagtccactggaatatgtgccagggccagatgaaacgcaaaaaaccatctttttaaa   35 475
tcacttccttaagcttaagtcaggtgaccttatacacggtcccggtctactttgcgttttttggtagaaaaattt
45        50        55        60        65
V K E F E F S P L E Y V P G P D E T Q K T I F L K
TP84_54

gatcgctaacatcatgggtgaactggaccgaattccgaaatcgggatacaacaagcaattcgattactattatgt   35 550
ctagcgattgtagtacccacttgacctggcttaaggctttagccctatgttgttcgttaagctaatgataataca
70        75        80        85        90
I A N I M G E L D R I P K S G Y N K Q F D Y Y Y V
TP84_54
```

```
gactgaatctgatgtggtcggcgcggtgcggccgctgatggcgaaatataaacttgtcatgatgccatcgctgaa   35 625
ctgacttagactacaccagccgcgccacgccggcgactaccgctttatatttgaacagtactacggtagcgactt
95        100       105       110       115
T E S D V V G A V R P L M A K Y K L V M M P S L K
TP84_54

aaaatacaaaacagaaacgatccagggcaaatacaacaagatgatcatgggaacggtcgaaatcgaatggatcgt   35 700
ttttatgttttgtctttgctaggtcccgtttatgttgttctactagtacccttgccagctttagcttacctagca
120       125       130       135       140
K Y K T E T I Q G K Y N K M I M G T V E I E W I V
TP84_54

gcgtgatgccgaatcctatgaacagataaaattcacgatgttaggcaagggcctggacaatctggaaaaagacat   35 775
cgcactacggcttaggatacttgtctattttaagtgctacaatccgttcccggacctgttagacctttttctgta
145       150       155       160       165
R D A E S Y E Q I K F T M L G K G L D N L E K D I
TP84_54

ttacaaagcaatcaccgggaataaaaaatatgcgctgatcacactgtttatgatcgacagcggtgacgatccaga   35 850
aatgtttcgttagtggcccttattttttatacgcgactagtgtgacaaatactagctgtcgccactgctaggtct
170       175       180       185       190
Y K A I T G N K K Y A L I T L F M I D S G D D P E
TP84_54

acggaacgacacaccgactggtgaagaagggccacaaaacgcacagaatgggccagaaaacacgaaaaagacaca   35 925
tgccttgctgtgtggctgaccacttcttcccggtgttttgcgtgtcttacccggtcttttgtgctttttctgtgt
195       200       205       210       215
R N D T P T G E E G P Q N A Q N G P E N T K K T Q
TP84_54

agggaatagtaaaacaccagggcaaccgaaaaacggccaggaagtgccaaaaaatgcgtcacaagggcaaccgca   36 000
tcccttatcattttgtggtcccgttggctttttgccggtccttcacggttttttacgcagtgttcccgttggcgt
220       225       230       235       240
G N S K T P G Q P K N G Q E V P K N A S Q G Q P Q
TP84_54

acaaccgccgcaacagccgccagtcaaacaaccgacaaaaggtgatctattgacacggtgggtggtcctggcagg   36 075
tgttggcggcgttgtcggcggtcagtttgttggctgttttccactagataactgtgccacccaccaggaccgtcc
245       250       255       260       265
Q P P Q Q P P V K Q P T K G D L L T R W V V L A G
TP84_54

gccggatcaaaagaaacaggaagtgcggaaggcgttcgatgaatggtatgaaaagaaaaaggccgaaggatggga   36 150
cggcctagttttctttgtccttcacgccttccgcaagctacttaccatacttttcttttttccggcttcctaccct
270       275       280       285       290
P D Q K K Q E V R K A F D E W Y E K K K A E G W D
TP84_54

tcacatggccatgatgcaagcattgacgaaaaaactgcatgaaaagaaccaaaaagaaaagcaggcacagcaaca   36 225
agtgtaccggtactacgttcgtaactgcttttttgacgtacttttcttggtttttcttttcgtccgtgtcgttgt
295       300       305       310       315
H M A M M Q A L T K K L H E K N Q K E K Q A Q Q Q
TP84_54

gcaagcagatggacaaagtggacaagcacagcaacagcaaactgatgacaagtcgaaagaataacaatctggcca   36 300
cgttcgtctacctgtttcacctgttcgtgtcgttgtcgtttgactactgttcagctttcttattgttagaccggt
320       325       330       335       340
Q A D G Q S G Q A Q Q Q Q T D D K S K E *
TP84_54
```

ggatcatcctggcctatccaaaaaagcaggggggatatggtggcaaactggatctatcaataccatgtgtataac 36 375
cctagtaggaccggataggttttttcgtcccccctataccaccgtttgacctagatagttatggtacacatattg

1 5 10
M V A N W I Y Q Y H V Y N
TP84_55

gatcatatcagttttatcgtgtctattccgatggaatcagaagaatatgcgatcgattttgcaaaacgtgtcgca 36 450
ctagtatagtcaaaatagcacagataaggctaccttagtcttcttatacgctagctaaaacgttttgcacagcgt

15 20 25 30 35
D H I S F I V S I P M E S E E Y A I D F A K R V A
TP84_55

ctggattatcttgattatccgctttaccaactggatgacgtgaaatgtgaattcatcgtcagatttttttgcgaa 36 525
gacctaatagaactaataggcgaaatggttgacctactgcactttacacttaagtagcagtctaaaaaaacgctt

40 45 50 55 60
L D Y L D Y P L Y Q L D D V K C E F I V R F F C E
TP84_55

tcagtcatcgatgtgaacaaactgcaaaaatagcggcgtgatcaaagcgatgtgccgtatgatcaagatcctggg 36 600
agtcagtagctacacttgtttgacgtttttatcgccgcactagtttcgctacacggcatactagttctaggaccc

65 70
S V I D V N K L Q K *
TP84_55

aacaaaagaggggtgacaaatctgacatccctaaattataaaaaataccgtaaaaataatactttataagatcat 36 675
ttgttttctccccactgtttagactgtagggatttaatatttttatggcatttttattatgaaatattctagta

putative host-dependent promoter

aggggtgacactggtgtcactcctaatcaaaaaggggtgacactggtgacatccctggggtgacactggtgtcac 36 750
tccccactgtgaccacagtgaggattagtttttccccactgtgaccactgtagggaccccactgtgaccacagtg

tccgggggggtgacaaatctggcacataacaattactatatatacaaatactaattacaaatagttattatatgt 36 825
aggcccccccactgtttagaccgtgtattgttaatgatatatatgtttatgattaatgtttatcaataatataca

cacgatcctgtggatcatgacaatttttttcagtttttttgtttacagtttataaacaataagctataatgaaagta 36 900
gtgctaggacacctagtactgttaaaaaagtcaaaaaaacaaatgtcaaatatttgttattcgatattactttcat

putative host-dependent promoter

ccaaacaggaaaggggtgcaacgatggagaagaaacaaaaattcaatccgatcctttcatttcagaccgatgaat 36 975
ggtttgtcctttccccacgttgctacctcttctttgtttttaagttaggctaggaaagtaaagtctggctactta

1 5 10 15
M E K K Q K F N P I L S F Q T D E
TP84_56

cggtgtgcgaattcatccagaaagtgatgatcaaacgaatggaattcaacagatcgaatatcgtgcgtgagattt 37 050
gccacacgcttaagtaggtctttcactactagtttgcttaccttaagttgtctagcttatagcacgcactctaaa

20 25 30 35 40
S V C E F I Q K V M I K R M E F N R S N I V R E I
TP84_56

ttatgattggcctggaagaattcaaaaaacgtcatccagagattgacaagaagtgaagtgaatgggggaatgatc 37 125
aatactaaccggaccttcttaagttttttgcagtaggtctctaactgttcttcacttcacttacccccttactag

45 50 55 60
F M I G L E E F K K R H P E I D K K *
TP84_56

tatgtcagatcaagaaaaacaagaatatctgatgtcggatgacatgtgggtgtatggatatagtgtcatcgacaa 37 200
atacagtctagttctttttgttcttatagactacagcctactgtacacccacatacctatatcacagtagctgtt
1 5 10 15 20 25
M S D Q E K Q E Y L M S D D M W V Y G Y S V I D N
TP84_57

tggtgtgatttattcgaaagaaatcactgattccgaattccgcacatattgtgtcatccgatcactggtgaatga 37 275
accacactaaataagctttctttagtgactaaggcttaaggcgtgtataacacagtaggctagtgaccacttact
30 35 40 45 50
G V I Y S K E I T D S E F R T Y C V I R S L V N E
TP84_57

acgaaaagcagtggcctggccatcctatgaaactattgcagaattatccgggcattccaaacgaactgcgatgcg 37 350
tgcttttcgtcaccggaccggtaggatactttgataacgtcttaataggcccgtaaggtttgcttgacgctacgc
55 60 65 70 75
R K A V A W P S Y E T I A E L S G H S K R T A M R
TP84_57

aaatgtggccagattgatcgaactggatctgatcgaaaaacggccacgatctggaacgtcaaacgaatttgtggt 37 425
tttacaccggtctaactagcttgacctagactagctttttgccggtgctagaccttgcagtttgcttaaacacca
80 85 90 95 100
N V A R L I E L D L I E K R P R S G T S N E F V V
TP84_57

gaaaaaactgcaaaattccaaagtgttaaaaaacaaacaagacatcctggattatatagagaaatgccgggatga 37 500
ctttttgacgttttaaggtttcacaattttttgtttgttctgtaggacctaatatatctctttacggccctact
105 110 115 120 125
K K L Q N S K V L K N K Q D I L D Y I E K C R D D
TP84_57

tgaaccgaaaaaagccactgatcctggtgaaaaagtggacaagccggaaaaagccgatccgatcccttacaaaga 37 575
acttggcttttttcggtgactaggaccactttttcacctgttcggcctttttcggctaggctagggaatgtttct
130 135 140 145 150
E P K K A T D P G E K V D K P E K A D P I P Y K E
TP84_57

gatcatcgactatctaaacgaaaaagctggaacgaaatacagccacactggatcggcgaatcaaaaactaatcaa 37 650
ctagtagctgatagatttgctttttcgaccttgctttatgtcggtgtgacctagccgcttagtttttgattagtt
155 160 165 170 175
I I D Y L N E K A G T K Y S H T G S A N Q K L I K
TP84_57

ggcacgatggaacgaaatggccaaaataaacaaagaccgtgactggatcgtggcgcagtttaaacacgtgatcga 37 725
ccgtgctaccttgctttaccggttttatttgtttctggcactgacctagcaccgcgtcaaatttgtgcactagct
180 185 190 195 200
A R W N E M A K I N K D R D W I V A Q F K H V I D
TP84_57

tgtcaaaacagcacaatggaagggtacagaatgggaaaaatatctgcggccatccacgttattcggcaataaatt 37 800
acagttttgtcgtgttaccttcccatgtcttaccctttttatagacgccggtaggtgcaataagccgttatttaa
205 210 215 220 225
V K T A Q W K G T E W E K Y L R P S T L F G N K F
TP84_57

tgatcaatacagaaacgaatcgccgaatcacaagccagtcggtggacaaaacagaccaaaagaggacatccgaac 37 875
actagttatgtctttgcttagcggcttagtgttcggtcagccacctgttttgtctggttttctcctgtaggcttg
230 235 240 245 250
D Q Y R N E S P N H K P V G G Q N R P K E D I R T
TP84_57

```
aagactggcaggattcctggctgatgaagattaaagagatataaaggggaaaggggcgatcgatcatgacaaaa   37 950
ttctgaccgtcctaaggaccgactacttctaatttctctatattttcccctttccccgctagctagtactgtttt
    255       260                                              1
 R  L  A  G  F  L  A  D  E  D  *                               M  T  K
 TP84_57                                                       TP84_58

acagaaatgatcactttgcttgaacagatcgaatcgtttttccaggccgcttcacagcagacgaaaaacagcg   38 025
tgtctttactagtgaaacgaacttgtctagcttagcaaaaaggtccggcgaagtgtcgtctgctttttgtcgc
    5         10        15        20        25
 T  E  M  I  T  L  L  E  Q  I  E  S  F  F  P  G  R  F  T  A  D  E  K  T  A
 TP84_58

gcggtctggtgggaagtgttaaaggattatgattttgaacactgcaaaaagaatctgatcaatcacgtcaagtca   38 100
cgccagaccacccttcacaatttcctaatactaaaacttgtgacgtttttcttagactagttagtgcagttcagt
    30        35        40        45        50
 A  V  W  W  E  V  L  K  D  Y  D  F  E  H  C  K  K  N  L  I  N  H  V  K  S
 TP84_58

tccgaatttcccccaacgatcgccaatctgatcgcaggatcaaaagacacaagccggaactattcagatgatctg   38 175
aggcttaaagggggttgctagcggttagactagcgtcctagttttctgtgttcggccttgataagtctactagac
    55        60        65        70        75
 S  E  F  P  P  T  I  A  N  L  I  A  G  S  K  D  T  S  R  N  Y  S  D  D  L
 TP84_58

atcgaaaagatcgaaaatgtaccagaaatagatgaaatcgttgcagaaatagaacggcgcacaggaagaaaggtg   38 250
tagcttttctagcttttacatggtctttatctactttagcaacgtctttatcttgccgcgtgtccttctttccac
    80        85        90        95        100
 I  E  K  I  E  N  V  P  E  I  D  E  I  V  A  E  I  E  R  R  T  G  R  K  V
 TP84_58

atgtggtaatgatctataatatcgatgcagaacaatattttcttggatgtttaatgcttgaaccagggctggtca   38 325
tacaccattactagatattatagctacgtcttgttataaaagaacctacaaattacgaacttggtcccgaccagt
          1         5         10        15        20
          M  I  Y  N  I  D  A  E  Q  Y  F  L  G  C  L  M  L  E  P  G  L  V
          TP84_59
    105
 M  W  *
 TP84_58

aagaaacaaagataagaccagtgcatctatctgaacgacatcgaatcatcctggatgcgattctaaaagcagatg   38 400
ttctttgtttctattctggtcacgtagatagacttgctgtagcttagtaggacctacgctaagattttcgtctac
      25        30        35        40        45
 K  E  T  K  I  R  P  V  H  L  S  E  R  H  R  I  I  L  D  A  I  L  K  A  D
 TP84_59

aaaagggaaacacgccggacatcgtggccattgtcactgaaatgggcgatgataaagtggcggcgatcggcggcg   38 475
ttttccctttgtgcggcctgtagcaccggtaacagtgactttacccgctactatttcaccgccgctagccgccgc
      50        55        60        65        70
 E  K  G  N  T  P  D  I  V  A  I  V  T  E  M  G  D  D  K  V  A  A  I  G  G
 TP84_59

tgtcctatcttactgaattgatcggtgcagtgcctaccacatcaaatgtgaagatgtatgaacaatttattatcg   38 550
acaggatagaatgacttaactagccacgtcacggatggtgtagtttacacttctacatacttgttaaataatagc
      75        80        85        90        95
 V  S  Y  L  T  E  L  I  G  A  V  P  T  T  S  N  V  K  M  Y  E  Q  F  I  I
 TP84_59
```

atgcatacaaactgcgtctggcaaccgaagcggcgcaaagatttatcgaggatgtaagaggaagccgcgatccga 38 625
tacgtatgtttgacgcagaccgttggcttcgccgcgtttctaaatagctcctacattctccttcggcgctaggct
100 105 110 115 120
D A Y K L R L A T E A A Q R F I E D V R G S R D P
TP84_59

tcctggtgaaccaactggcacaaaaaatggtcgagatccaggaatatggaagtattgtgaaaagaagatcctggc 38 700
aggaccacttggttgaccgtgttttttaccagctctaggtccttataccttcataacacttttcttctaggaccg
125 130 135 140 145
I L V N Q L A Q K M V E I Q E Y G S I V K R R S W
TP84_59

gtgacatccttgtcgatctggtggaagaatcatatcaagaaaaaatcggattgactggaatcaacactggctttg 38 775
cactgtaggaacagctagaccaccttcttagtatagttcttttttagcctaactgaccttagttgtgaccgaaac
150 155 160 165 170
R D I L V D L V E E S Y Q E K I G L T G I N T G F
TP84_59

ctggattgaatcggatgacagacggtttgcaagggaacaatctgatcatcatcggtgcgcggccatcgatgggta 38 850
gacctaacttagcctactgtctgccaaacgttcccttgttagactagtagtagccacgcgccggtagctacccat
175 180 185 190 195
A G L N R M T D G L Q G N N L I I I G A R P S M G
TP84_59

aaacggcattcgcattgaacatcggcacaaatgcttgcacattagatgatgcaagggtggacatcttttcactgg 38 925
tttgccgtaagcgtaacttgtagccgtgtttacgaacgtgtaatctactacgttcccacctgtagaaaagtgacc
200 205 210 215 220
K T A F A L N I G T N A C T L D D A R V D I F S L
TP84_59

aaacgccggaaaaacgtctggccaaacggatcatcgcatcggttggcaatatcagtgctgaacgactgcggcgaa 39 000
tttgcggcctttttgcagaccggtttgcctagtagcgtagccaaccgttatagtcacgacttgctgacgccgctt
225 230 235 240 245
E T P E K R L A K R I I A S V G N I S A E R L R R
TP84_59

tggattttgacgataaaacacgtgaacgatttattcaaaaggtcggcctggtggattcgtttgacttgcacattc 39 075
acctaaaactgctattttgtgcacttgctaaataagttttccagccggaccacctaagcaaactgaacgtgtaag
250 255 260 265 270
M D F D D K T R E R F I Q K V G L V D S F D L H I
TP84_59

atgatcaatcgacaatcactgtcgaggaaatccgatcgatcgtggccgaaagcaatcgccaggcacggaaagaag 39 150
tactagttagctgttagtgacagctcctttaggctagctagcaccggctttcgttagcggtccgtgcctttcttc
275 280 285 290 295
H D Q S T I T V E E I R S I V A E S N R Q A R K E
TP84_59

gcaaaaaacatctggtcatcatcgactatctgcaactgatcacatatcgtgggccgctacaaaacaaggtccaac 39 225
cgttttttgtagaccagtagtagctgatagacgttgactagtgtatagcacccggcgatgttttgttccaggttg
300 305 310 315 320
G K K H L V I I D Y L Q L I T Y R G P L Q N K V Q
TP84_59

aaattggccatatatccagacaattgaaactgatggccggggatttttgacatcccgatcatcgcactgtcgcaat 39 300
tttaaccggtatataggtctgttaactttgactaccggcccctaaaaactgtagggctagtagcgtgacagcgtta
325 330 335 340 345
Q I G H I S R Q L K L M A G D F D I P I I A L S Q
TP84_59

tatcgcgcggtgttgaacaacgacaagacaaacggcctgtgatggctgatctgcgtgaatcgggtgacatcgaac 39 375
atagcgcgccacaacttgttgctgttctgtttgccggacactaccgactagacgcacttagcccactgtagcttg

350 355 360 365 370
L S R G V E Q R Q D K R P V M A D L R E S G D I E
TP84_59

aagatgccgacatcatcgctttttctttaccgggatgactattatgacaaagaatctgacaaaaagaacatcacgg 39 450
ttctacggctgtagtagcgaaaaagaaatggccctactgataatactgtttcttagactgtttttcttgtagtgcc

375 380 385 390 395
Q D A D I I A F L Y R D D Y Y D K E S D K K N I T
TP84_59

aagtcatcatcgctaaaaaccgggatggcgcagttggtacagtggaactggcgttcattaaagaatacaacaaat 39 525
ttcagtagtagcgatttttggccctaccgcgtcaaccatgtcaccttgaccgcaagtaatttcttatgttgttta

400 405 410 415 420
E V I I A K N R D G A V G T V E L A F I K E Y N K
TP84_59

tcttggatctggaacgccgattcgatgaagcatcatgattctacatacagcttatccaaacaagcccaaaaatat 39 600
agaacctagaccttgcggctaagctacttcgtagtactaagatgtatgtcgaataggtttgttcgggtttttata

425 430 435
F L D L E R R F D E A S *
TP84_59

tcataaaaaataaagaaagggagaatgaagaatgcaagtgatctcgaaacgattcagattcaatcaatactggga 39 675
agtatttttttatttctttccctcttacttcttacgttcactagagctttgctaagtctaagttagttatgaccct

1 5 10 15
M Q V I S K R F R F N Q Y W E
TP84_60

attcgtgatggtcgatgggcaattctacatcttggcgaaagacatcctggggtatttcggatataaacggaccag 39 750
taagcactaccagctacccgttaagatgtagaaccgctttctgtaggaccccataaagcctatatttgcctggtc

20 25 30 35 40
F V M V D G Q F Y I L A K D I L G Y F G Y K R T S
TP84_60

tcacaaagtgatcaccgaattactcactaatgatgatcgattgatcatcaaaggcaaagcaacacacgacatgaa 39 825
agtgtttcactagtggcttaatgagtgattactactagctaactagtagtttccgtttcgttgtgtgctgtactt

45 50 55 60 65
H K V I T E L L T N D D R L I I K G K A T H D M K
TP84_60

gcatgtcgagaaaggacaacatgcgttattgatcacaaaggccggcctgatgacgtttgtttacaaccgattaaa 39 900
cgtacagctctttcctgttgtacgcaataactagtgtttccggcccgactactgcaaacaaatgttggctaattt

70 75 80 85 90
H V E K G Q H A L L I T K A G L M T F V Y N R L K
TP84_60

agatgacaagcgagaaaaagggatcgaattgatccactggatcaacgaaaaagcaatcccggaaatgaaagagga 39 975
tctactgttcgctctttttccctagcttaactaggtgacctagttgctttttcgttagggcctttactttctcct

95 100 105 110 115
D D K R E K G I E L I H W I N E K A I P E M K E E
TP84_60

atgggaaaaatttttaaaaatcaatggaaaagacagcggtttcgaggaacggtttagcacggatgatcaaaaaca 40 050
taccctttttaaaaatttttagttaccttttctgtcgccaaagctccttgccaaatcgtgcctactagtttttgt

120 125 130 135 140
W E K F L K I N G K D S G F E E R F S T D D Q K H
TP84_60

tccagatttatttgatatggcactggaacaacaaattgaagaattgaaatgcattcaagaagtacaagaaaaaac 40 125
aggtctaaataaactataccgtgaccttgttgtttaacttcttaactttacgtaagttcttcatgttcttttttg
145 150 155 160 165
P D L F D M A L E Q Q I E E L K C I Q E V Q E K T
TP84_60

gatccgccgcttgcaagaaaaactggcgatggccgaaaagcgcatcgaagcactggaaaaggcactgaaacaaga 40 200
ctaggcggcgaacgttctttttgaccgctaccggcttttcgcgtagcttcgtgaccttttccgtgactttgttct
170 175 180 185 190
I R R L Q E K L A M A E K R I E A L E K A L K Q E
TP84_60

gaagttagaaaaagcggtcgtgaatggcgaacgcaagctgatcgatgtggaccgattgatcaactatctgaaagg 40 275
cttcaatctttttcgccagcacttaccgcttgcgttcgactagctacacctggctaactagttgatagactttcc
195 200 205 210 215
K L E K A V V N G E R K L I D V D R L I N Y L K G
TP84_60

tgtggatgcaagtgggcatattcgatgaagtgaacgcagtgcccaaaccaaaacacaagcggcggacaccaaagc 40 350
acacctacgttcacccgtataagctacttcacttgcgtcacgggtttggttttgtgttcgccgcctgtggtttcg
220
V D A S G H I R *
TP84_60
1 5 10 15 20
M Q V G I F D E V N A V P K P K H K R R T P K
TP84_61

ggaaatccaggggcgaattcagccaggaaacgattcgggcgatctgggaacgtgacggctatcgatgcgtgaaat 40 425
cctttaggtccccgcttaagtcggtcctttgctaagcccgctagacccttgcactgccgatagctacgcacttta
25 30 35 40 45
R K S R G E F S Q E T I R A I W E R D G Y R C V K
TP84_61

gcggatcatatcacctggaaaaagtgccgcaccatgtgatctataaaagccagggcggtcgtggcacaaagcgga 40 500
cgcctagtatagtggacctttttcacggcgtggtacactagatattttcggtcccgccagcaccgtgtttcgcct
50 55 60 65 70
C G S Y H L E K V P H H V I Y K S Q G G R G T K R
TP84_61

acggtgtgactgtttgccgtggatgccacgactgggcacacggtttgaaaccaggtccattcggagaaccgccag 40 575
tgccacactgacaaacggcacctacggtgctgacccgtgtgccaaactttggtccaggtaagcctcttggcggtc
75 80 85 90 95
N G V T V C R G C H D W A H G L K P G P F G E P P
TP84_61

aagatggccgcaaatggtttgaaaactgggtggaaacatacttggatgaaaacggtgatctgatccgaaaggggg 40 650
ttctaccggcgtttaccaaacttttgacccacctttgtatgaacctacttttgccactagactaggctttccccc
100 105 110 115 120
E D G R K W F E N W V E T Y L D E N G D L I R K G
TP84_61

atcatcgtggcaagattcgtgactttttcctgggagaatgggaaacaggtgatcacgatctatgaggaagggatg 40 725
tagtagcaccgttctaagcactgaaaaaggaccctcttaccctttgtccactagtgctagatactccttccctac
125 130 135 140 145
D H R G K I R D F F L G E W E T G D H D L *
TP84_61
1 5
M I T I Y E E G M
TP84_62

aaacgaaagcggatcatccaggaacaagatcggccgtttcttatccaggcgatgcataaaacaaatgatggccgc
tttgctttcgcctagtaggtccttgttctagccggcaaagaataggtccgctacgtattttgtttactaccggcg
10 15 20 25 30
K R K R I I Q E Q D R P F L I Q A M H K T N D G R
TP84_62
40 800

tatgagtgggccaacgaaaaaccaaaatacatgaaaggggaataagaacaatgaacaatgtgacgttagtgggaa
atactcacccggttgcttttttggttttatgtactttccccttattcttgttacttgttacactgcaatcaccctt
35 40 45 1 5
Y E W A N E K P K Y M K G E * M N N V T L V G
TP84_62 TP84_63
40 875

gattgacgaacgatgtggaattgagatacacacaacaagggaaagcagtggccacattcaacctggcagtgcaaa
ctaactgcttgctacaccttaactctatgtgtgttgttccctttcgtcaccggtgtaagttggaccgtcacgttt
10 15 20 25 30
R L T N D V E L R Y T Q Q G K A V A T F N L A V Q
TP84_63
40 950

gagaattcaaaaaccaggataatgtttatgaagtggactttccacagattgtcgtctggggcaagcctgcggaaa
ctcttaagtttttggtcctattacaaatacttcacctgaaaggtgtctaacagcagaccccgttcggacgcctttt
35 40 45 50 55
R E F K N Q D N V Y E V D F P Q I V V W G K P A E
TP84_63
41 025

cgttggccaactacaccaaaaaaggcagtttaatcgggatcacaggtcgattgcaaacacggtcctatgaaagac
gcaaccggttgatgtggttttttccgtcaaattagccctagtgtccagctaacgtttgtgccaggatactttctg
60 65 70 75 80
T L A N Y T K K G S L I G I T G R L Q T R S Y E R
TP84_63
41 100

aggatggcagtcgtgtttatgtgacggaagtggtggctaacaatgtacggatctatcaatggaaagatagtggac
tcctaccgtcagcacaaatacactgccttcaccaccgattgttacatgcctagatagttacctttctatcacctg
85 90 95 100 105
Q D G S R V Y V T E V V A N N V R I Y Q W K D S G
TP84_63
41 175

aaggtggacaaagcagacagggcggatcacaaccacaaggccgtggatcacgttatccagatccttatggtccat
ttccacctgtttcgtctgtcccgcctagtgttggtgttccggcacctagtgcaataggtctaggaataccaggta
110 115 120 125 130
Q G G Q S R Q G G S Q P Q G R G S R Y P D P Y G P
TP84_63
41 250

atgacggcgatccatttgcaaacgatggcgaaccgatttatgtgaatgatgacgatctgccattttaggcagtga
tactgccgctaggtaaacgtttgctaccgcttggctaaatacacttactactgctagacggtaaaatccgtcact
135 140 145 150 155
Y D G D P F A N D G E P I Y V N D D D L P F *
TP84_63
41 325

atgaacagggggtgatcatggtgaaatattttgtcggcagtgatgttcattttatcgcattcgatcaaaaccttt
tacttgtcccccactagtaccactttataaaacagccgtcactacaagtaaaatagcgtaagctagttttggaaa
1 5 10 15
M V K Y F V G S D V H F I A F D Q N L
TP84_64
41 400

tcaaacacgacaaatgttttcacatcaaacgatctgatcaaatttatagcggcataacgatttataaagtcgccg
agtttgtgctgtttacaaaagtgtagtttgctagactagtttaaatatcgccgtattgctaaatatttcagcggc
20 25 30 35 40
F K H D K C F H I K R S D Q I Y S G I T I Y K V A
TP84_64
41 475

```
gatcacaaatcgccgactatctgcacgacaggatcatgaacatgtatatcaaaggtgacatcaatctggaagtta   41 550
ctagtgtttagcggctgatagacgtgctgtcctagtacttgtacatatagtttccactgtagttagaccttcaat
45        50        55        60        65
G S Q I A D Y L H D R I M N M Y I K G D I N L E V
TP84_64

tgccggaaagcctactttacaaaaccatataggccagggatcatcctggcctggaaaggggtggacaacatggac   41 625
acggcctttcggatgaaatgttttggtatatccggtccctagtaggaccggacctttccccacctgttgtacctg
70        75        80                  1         5         10
M P E S L L Y K T I *                   M A W K G V D N M D
TP84_64                                 TP84_65

aacgaacgcaaaaaaggaccaatcgaagtggcgttcgattggacattggcgatcattgcactggccacgatcatc   41 700
ttgcttgcgttttttcctggttagcttcaccgcaagctaacctgtaaccgctagtaacgtgaccggtgctagtag
          15        20        25        30        35
N E R K K G P I E V A F D W T L A I I A L A T I I
TP84_65

ggcctggcctggaaatacattttaaaaccattattcctggggtgataagcatggatattcgtgaagaaatctatc   41 775
ccggaccggacctttatgtaaaattttggtaataaggaccccactattcgtacctataagcacttctttagatag
          40        45        50              1         5
G L A W K Y I L K P L F L G *                 M D I R E E I Y
TP84_65                                       TP84_66

aagcgcaaaaggaacgtgaagaattcgaaaaagcgcgcgccgcatcgatgccggatgaagtcgaaagaaaaatcg   41 850
ttcgcgttttccttgcacttcttaagctttttcgcgcgcggcgtagctacggcctacttcagctttctttttagc
  10        15        20        25        30
Q A Q K E R E E F E K A R A A S M P D E V E R K I
TP84_66

tggaactgatgaatgatccacgcattcaagcggaagtgatggcaatcattattcatctaaaaaaacaaaaacgaa   41 925
accttgactacttactaggtgcgtaagttcgccttcactaccgttagtaataagtagatttttttgtttttgctt
  35        40        45        50        55
V E L M N D P R I Q A E V M A I I I H L K K Q K R
TP84_66

aagacatcgagatcttgacgatcggtctgatggtcacgaacatggcacgaagcgtcatggaacaagcggcgcaaa   42 000
ttctgtagctctagaactgctagccagactaccagtgcttgtaccgtgcttcgcagtaccttgttcgccgcgttt
  60        65        70        75        80
K D I E I L T I G L M V T N M A R S V M E Q A A Q
TP84_66

ccaaacagaaaggggaatgatcatgggtgtgcatttgccgaaacacgccacagtgacgatcgatgacatcgtgat   42 075
ggtttgtctttccccttactagtacccacacgtaaacggctttgtgcggtgtcactgctagctactgtagcacta
  85        90      1         5         10        15
T K Q K G E *       M G V H L P K H A T V T I D D I V I
TP84_66             TP84_67

cgatttaccattcgaaatcgaactggattatcaaaaaagcaaagcggaagatccagaaatcaccgaactgaatct   42 150
gctaaatggtaagctttagcttgacctaatagttttttcgtttcgccttctaggtctttagtggcttgacttaga
    20        25        30        35        40
  D L P F E I E L D Y Q K S K A E D P E I T E L N L
TP84_67

gccgaaatcgatcgaaaaagaatacaacgtcataaagatcaaggaaggggaatgaacaatggaattcaaactgta   42 225
cggctttagctagctttttcttatgttgcagtatttctagttccttccccttacttgttaccttaagtttgacat
    45        50        55        60            1         5
  P K S I E K E Y N V I K I K E G E *           M E F K L Y
TP84_67                                         TP84_68
```

taaggtcgcaggatatgaagaagcgatcatgtcactgcggatgtcgaaaggcaaatatttttcctgggaacgagc 42 300
attccagcgtcctatacttcttcgctagtacagtgacgcctacagctttccgtttataaaaaggacccttgctcg
10 15 20 25 30
K V A G Y E E A I M S L R M S K G K Y F S W E R A
TP84_68

caaaaaaattcagcatcttgtctatgcggtgacagatcaccgcggattcattgcgccgccgcaagtttatatcaa 42 375
gtttttttaagtcgtagaacagatacgccactgtctagtggcgcctaagtaacgcggcggcgttcaaatatagtt
35 40 45 50 55
K K I Q H L V Y A V T D H R G F I A P P Q V Y I N
TP84_68

taacatgcgaaacctggcggcgatcgaggaagatataaaagtcgatcaccagggcaaaaaaggcgaaatcagcgg 42 450
attgtacgctttggaccgccgctagctccttctatattttcagctagtggtcccgttttttccgctttagtcgcc
60 65 70 75 80
N M R N L A A I E E D I K V D H Q G K K G E I S G
TP84_68

caactatatccgggatgtggatgaattcaaacgattgttggcactcacactgaacaatgcgatgggcgaacacaa 42 525
gttgatataggccctacacctacttaagtttgctaacaaccgtgagtgtgacttgttacgctacccgcttgtgtt
85 90 95 100 105
N Y I R D V D E F K R L L A L T L N N A M G E H K
TP84_68

acaccacacactgatgaaatacatcgacatttcattctttacgatcggactacatagaggggcgcaggatgatct 42 600
tgtggtgtgtgactactttatgtagctgtaaagtaagaaatgctagcctgatgtatctccccgcgtcctactaga
110 115 120 125 130
H H T L M K Y I D I S F F T I G L H R G A Q D D L
TP84_68

ggatgcacatgccatcgcattcaataaccgcatcacacgatattcaacacggctggccaacatccaggaaactgt 42 675
cctacgtgtacggtagcgtaagttattggcgtagtgtgctataagttgtgccgaccggttgtaggtcctttgaca
135 140 145 150 155
D A H A I A F N N R I T R Y S T R L A N I Q E T V
TP84_68

cttgtctgaatggtatcaagacaagatcattccattcgaacatgcacagcattttgttgggatcgaatggccgat 42 750
gaacagacttaccatagttctgttctagtaaggtaagcttgtacgtgtcgtaaaacaaccctagcttaccggcta
160 165 170 175 180
L S E W Y Q D K I I P F E H A Q H F V G I E W P M
TP84_68

ggtgattgaaacagacattggaaattttgaatacacaccattcggatacatccatgaaaatttcaacagaatcag 42 825
ccactaactttgtctgtaacctttaaaacttatgtgtggtaagcctatgtaggtacttttaaagttgtcttagtc
185 190 195 200 205
V I E T D I G N F E Y T P F G Y I H E N F N R I S
TP84_68

cgatgaaaatggattgaaaaaagatgtgaaacgcggtctgatcccactgtcaatggcatcgaacgcactatggaa 42 900
gctacttttacctaacttttttctacactttgcgccagactagggtgacagttaccgtagcttgcgtgatacctt
210 215 220 225 230
D E N G L K K D V K R G L I P L S M A S N A L W K
TP84_68

aattgatctgtttaatctgcgatatgtctataaaatgcggtcgaagctgacaaaagcgaatccagaactgaaaca 42 975
ttaactagacaaattagacgctatacagatattttacgccagcttcgactgttttcgcttaggtcttgactttgt
235 240 245 250 255
I D L F N L R Y V Y K M R S K L T K A N P E L K Q
TP84_68

agggatggaaatgctggcggatcagatcgaaaaacatgtgcctgtattcggtccatatttccgttatgaattgac 43 050
tccctacctttacgaccgcctagtctagcttttttgtacacggacataagccaggtataaaggcaatacttaactg

260 265 270 275 280
G M E M L A D Q I E K H V P V F G P Y F R Y E L T
TP84_68

agacactggcgaatgggaacacatgaacaaagtcaagaccgtcaccagggaagaatatgaactattgaaaaagat 43 125
tctgtgaccgcttacccttgtgtacttgtttcagttctggcagtggtcccttcttatacttgataactttttcta

285 290 295 300 305
D T G E W E H M N K V K T V T R E E Y E L L K K I
TP84_68

caaaaagcagatgcaacaacaaggcacagtcgatgtggatctttaccgtctgcaacaggaaggggaatgaaaaaa 43 200
gtttttcgtctacgttgttgttccgtgtcagctacacctagaaatggcagacgttgtccttccccttactttttt

310 315 320 325
K K Q M Q Q Q G T V D V D L Y R L Q Q E G E *
TP84_68
TP84_69

tggtgaaggtggatgaaagaacgatcaatttatcagatctattccaaaaacagaaaatgctgacggattatgtgt 43 275
accacttccacctactttcttgctagttaaatagtctagataaggtttttgtcttttacgactgcctaatacaca

1 5 10 15 20 25
M V K V D E R T I N L S D L F Q K Q K M L T D Y V
TP84_69

ggcaaaaacacaacatcaaaaaagcggatctggaaaaagaaaaagcggcgatcatctgcgaattgtgggaaaccg 43 350
ccgtttttgtgttgtagttttttcgcctagacctttttctttttcgccgctagtagacgcttaacaccctttggc

30 35 40 45 50
W Q K H N I K K A D L E K E K A A I I C E L W E T
TP84_69

caaacgaattgaaatccgatggcttcaaatattggaccgacaaaaaatgcgacagggaaaaaacgctggaagaaa 43 425
gtttgcttaactttaggctaccgaagtttataacctggctgttttttacgctgtcccttttttgcgaccttcttt

55 60 65 70 75
A N E L K S D G F K Y W T D K K C D R E K T L E E
TP84_69

tcgtggacatgttgcatttctatttgcagatcgggaacatcctgggtgtcgtttatgaacattactggatcgaaa 43 500
agcacctgtacaacgtaaagataaacgtctagcccttgtaggacccacagcaaatacttgtaatgacctagcttt

80 85 90 95 100
I V D M L H F Y L Q I G N I L G V V Y E H Y W I E
TP84_69

gacgtgacacgattctggatcagatcatggcgatcaactggtcattattaatgatggatgggccgctgacatggg 43 575
ctgcactgtgctaagacctagtctagtaccgctagttgaccagtaataattactacctacccggcgactgtaccc

105 110 115 120 125
R R D T I L D Q I M A I N W S L L M M D G P L T W
TP84_69

cggtgtcattcgcacaatatcgtgggcttgttcggatgcttggattcgactgggatcaagatattatcccggcat 43 650
gccacagtaagcgtgttatagcacccgaacaagcctacgaacctaagctgaccctagttctataatagggccgta

130 135 140 145 150
A V S F A Q Y R G L V R M L G F D W D Q D I I P A
TP84_69

```
ataaccgaaaattccaggaaaacatcgccagacagcagaggggatattaatcgatgtttattaaaattccatgtc   43 725
tattggcttttaaggtccttttgtagcggtctgtcgtctcccctataattagctacaaataattttaaggtacag
          155       160       165              1       5
Y N R K F Q E N I A R Q Q R G Y *              M F I K I P C
TP84_69                                        TP84_70

cagggatgtatctggaatttaaaaacaaaaacccgaaactgtttgtggaatattgcaaagcgtacatcaaacata   43 800
gtccctacatagaccttaaatttttgtttttgggctttgacaaacaccttataacgtttcgcatgtagtttgtat
  10        15        20        25        30
P G M Y L E F K N K N P K L F V E Y C K A Y I K H
TP84_70

gccactatggcttgaaggcggtcggggtggaaacatcccgatccggccaagtctacatcctaacaatcccggatt   43 875
cggtgataccgaacttccgccagccccacctttgtagggctaggccggttcagatgtaggattgttagggcctaa
  35        40        45        50        55
S H Y G L K A V G V E T S R S G Q V Y I L T I P D
TP84_70

atgatgcaaaacggccagtcgcaaaaggtagaggaaaaagcagacaagtcatattagaaccgatgtataaagtgc   43 950
tactacgttttgccggtcagcgttttccatctcctttttcgtctgttcagtataatcttggctacatatttcacg
  60        65        70        75        80
Y D A K R P V A K G R G K S R Q V I L E P M Y K V
TP84_70

cgatcgagaattttttgagtgaagaagaaatcgaatatctaaaaaggaaagggatggacaaatatggcaataaaa   44 025
gctagctcttaaaaaactcacttcttctttagcttatagatttttcctttccctacctgtttataccgttatttt
  85        90        95        100       105
P I E N F L S E E E I E Y L K R K G M D K Y G N K
TP84_70
                                                   1
                                                   M A I K
                                                   TP84_71

tcacgtgaagcgtacatgatcatgagaaatccagaaacgcacatggaaatcaaaatcgatctgacgaaagaaaac   44 100
agtgcacttcgcatgtactagtactctttaggtctttgcgtgtacctttagttttagctagactgctttcttttg
  110
I T *
TP84_70
5         10        15        20        25
S R E A Y M I M R N P E T H M E I K I D L T K E N
TP84_71

gccggggatctggcgatcaaagcgcatgaaaaaggattcgtgccgatggatcatgaactgacacgaaatcagtac   44 175
cggcccctagaccgctagtttcgcgtactttttcctaagcacggctacctagtacttgactgtgctttagtcatg
30        35        40        45        50
A G D L A I K A H E K G F V P M D H E L T R N Q Y
TP84_71

cacaaacaattcaaatcactatcgaaggcggtcaatccgaaaccgcgcaaacggaaaggaagccgcaaaaatggc   44 250
gtgtttgttaagtttagtgatagcttccgccagttaggctttggcgcgtttgcctttccttcggcgtttttaccg
                                                        1
                                                        M A
                                                        TP84_72
55        60        65        70        75
H K Q F K S L S K A V N P K P R K R K G S R K N G
TP84_71
```

aaaaagtaagggcaaggaactggaagatcgaatcatcaagaaaaacaaagaatatctggaacaagggattgctac 44 325
tttttcattcccgttccttgaccttctagcttagtagttctttttgtttcttatagaccttgttccctaacgatg

5 10 15 20 25
K S K G K E L E D R I I K K N K E Y L E Q G I A T
TP84_72

80
K K *
TP84_71

gatcaagaagatcccgaataaatgggcaatcagacggaaagggccgcacattgtcggggcgaacccgattccgtc 44 400
ctagttcttctagggcttatttacccgttagtctgcctttcccggcgtgtaacagccccgcttgggctaaggcag

30 35 40 45 50
I K K I P N K W A I R R K G P H I V G A N P I P S
TP84_72

tggtctatgcgattttattggcacatccaaactggtcagcggtcgtttgatcgtgttcgatgcgaaggaatgcaa 44 475
accagatacgctaaaataaccgtgtaggtttgaccagtcgccagcaaactagcacaagctacgcttccttacgtt

55 60 65 70 75
G L C D F I G T S K L V S G R L I V F D A K E C K
TP84_72

actgaaaacacgttttccactgaaaaatatcaagccggaacaaatggatcacatgcaggaaacggtcgaacacgg 44 550
tgacttttgtgcaaaaggtgactttttatagttcggccttgtttacctagtgtacgtcctttgccagcttgtgcc

80 85 90 95 100
L K T R F P L K N I K P E Q M D H M Q E T V E H G
TP84_72

cggcatcgcttttctgatcgttcattttactgaactggatgaaatgtatttcctaccatatgaattcgtgaaacc 44 625
gccgtagcgaaaagactagcaagtaaaatgacttgacctactttacataaaggatggtatacttaagcactttgg

105 110 115 120 125
G I A F L I V H F T E L D E M Y F L P Y E F V K P
TP84_72

atattgggaagcggctgaaaaggaagaagggccatcatcgattccgatagcggacatccgggaatcttgcacgca 44 700
tataacccttcgccgacttttccttcttcccggtagtagctaaggctatcgcctgtaggcccttagaacgtgcgt

130 135 140 145 150
Y W E A A E K E E G P S S I P I A D I R E S C T Q
TP84_72

gatcatcgacatggattatttcacgcatatccagatgttttacaaatgaaaccgaacatcaccgatcgatccacc 44 775
ctagtagctgtacctaataaagtgcgtataggtctacaaaatgtttactttggcttgtagtggctagctaggtgg

155 160 165
I I D M D Y F T H I Q M F Y K *
TP84_72

1 5 10
M K P N I T D R S T
TP84_73

tggacatctgggtggatcgtggaagatccagacaaccacattttgatctgtgtcgatgaatgcggcaacgtggta 44 850
acctgtagacccacctagcaccttctaggtctgttggtgtaaaactagacacagctacttacgccgttgcaccat

15 20 25 30 35
W T S G W I V E D P D N H I L I C V D E C G N V V
TP84_73

ggaacatgtgacgcaatgatgccgccagtcatggtcaaacggtggatcgtgaaggtgggggaattcaaaaaaatc 44 925
ccttgtacactgcgttactacggcggtcagtaccagtttgccacctagcacttcccccccttaagtttttttag

40 45 50 55 60
G T C D A M M P P V M V K R W I V K V G E F K K I
TP84_73

```
aacattcgaaaccagatgcactatatacagaaatggaacgaaagaatggtgaatgatgatgacgaaaaatatcga   45 000
ttgtaagctttggtctacgtgatatatgtctttaccttgctttcttaccacttactactactgctttttatagct

                                                            1       5
                                                            M  T  K  N  I  D
                                                            TP84_74 >

        65              70              75              80              85
N  I  R  N  Q  M  H  Y  I  Q  K  W  N  E  R  M  V  N  D  D  D  E  K  Y  R
TP84_73 >

ttcgaataaacaagcggcaaccacgaaaccgccgcagggaaaaatatatgatcagcggtggcaggattatcaaag   45 075
aagcttatttgttcgccgttggtgctttggcggcgtccctttttatatactagtcgccaccgtcctaatagtttc

           10              15              20              25              30
S  N  K  Q  A  A  T  T  K  P  P  Q  G  K  I  Y  D  Q  R  W  Q  D  Y  Q  R
TP84_74 >

F  E  *
TP84_73

acggatcggctttacaaaaagacggtggcaaaatacaacgccattgtgaaacggatcgaggaattatataaaat   45 150
tgcctagccgaaaatgtttttctgccaccgttttatgttgcggtaacactttgcctagctccttaatatatttta

           35              40              45              50              55
R  I  G  F  Y  K  K  T  V  A  K  Y  N  A  I  V  K  R  I  E  E  L  Y  K  I
TP84_74 >

cacggaacaactggatgacaacaatcccgatgatgtactgaaaaaagccgaaatatacggcatgatcctggaaga   45 225
gtgccttgttgacctactgttgttagggctactacatgacttttttcggctttatatgccgtactaggaccttct

           60              65              70              75              80
T  E  Q  L  D  D  N  N  P  D  D  V  L  K  K  A  E  I  Y  G  M  I  L  E  D
TP84_74 >

tgtcggcgatctgcattatatttcgatcactgcctggaaatacgccgatgcactgaaaaaggaagcctatgcact   45 300
acagccgctagacgtaatataaagctagtgacggacctttatgcggctacgtgactttttccttcggatacgtga

           85              90              95              100             105
V  G  D  L  H  Y  I  S  I  T  A  W  K  Y  A  D  A  L  K  K  E  A  Y  A  L
TP84_74 >

ggcgatcatccgggaaaggccaaacggccgcacagtcgaagcgcaccgggaaatggcggtcctggaatcgcagga   45 375
ccgctagtaggccctttccggtttgccggcgtgtcagcttcgcgtggccctttaccgccaggaccttagcgtcct

           110             115             120             125             130
A  I  I  R  E  R  P  N  G  R  T  V  E  A  H  R  E  M  A  V  L  E  S  Q  E
TP84_74 >

atggcgatggaaaatggctgaatgggaaggattgacgaaacggtgggaaaacgcaaagacaacaattgaagaaca   45 450
taccgctaccttttaccgacttacccttcctaactgctttgccacccttttgcgtttctgttgttaacttcttgt

           135             140             145             150             155
W  R  W  K  M  A  E  W  E  G  L  T  K  R  W  E  N  A  K  T  T  I  E  E  Q
TP84_74 >

gatcaaaatcatgaagtggaagatcaaatggacactggcgaacatgcaacaagcaggaatggcgaatccgaatgc   45 525
ctagttttagtacttcaccttctagtttacctgtgaccgcttgtacgttgttcgtccttaccgcttaggcttacg

           160             165             170             175             180
I  K  I  M  K  W  K  I  K  W  T  L  A  N  M  Q  Q  A  G  M  A  N  P  N  A
TP84_74 >

ataacgaaggggggtgttcagtggtgaaatgtgctatctgcggctatgaattgacaatcgatgaagaaatcgaag   45 600
tattgcttccccccacaagtcaccactttacacgatagacgccgatacttaactgttagctacttctttagcttc

   *                      1           5               10              15
TP84_74                   M  V  K  C  A  I  C  G  Y  E  L  T  I  D  E  E  I  E
                          TP84_75 >
```

tcatgctggccgatgatgatgaatgcccaaaatgcggcggcggattgactgtcgaatgaaaaggaggtaaaggaa 45 675
agtacgaccggctactactacttacgggttttacgccgccgcctaactgacagcttacttttcctccatttcctt

20 25 30 35
V M L A D D D E C P K C G G G L T V E *
TP84_75
TP84_76

tgaagcggcacggaaaatgctattcgtgccagacaacaggggaaacgatatacagccagacgatggccgaatggt 45 750
acttcgccgtgccttttacgataagcacggtctgttgtcccctttgctatatgtcggtctgctaccggcttacca

1 5 10 15 20 25
M K R H G K C Y S C Q T T G E T I Y S Q T M A E W
TP84_76

tgtgtatggaatgctacaaagcatttctggatcaaatcatcgatggataaaaagtggacaaagtggacatgtcca 45 825
acacataccttacgatgtttcgtaaagacctagtttagtagctacctatttttcacctgtttcacctgtacaggt

30 35 40
L C M E C Y K A F L D Q I I D G *
TP84_76

cgatgtccacatcaaaaaaagggggatgatcatggccaaactgaatggatactggatcagcaaaactgatccgaa 45 900
gctacaggtgtagtttttttccccctactagtaccggtttgacttacctatgacctagtcgttttgactaggctt

1 5 10 15
M A K L N G Y W I S K T D P N
TP84_77

cgtgacggtgtatgtcgaaaaggtctacaaaaaaggatacgtcactggattcaaatatatgaaggttgcagacgg 45 975
gcactgccacatacagcttttccagatgttttttcctatgcagtgacctaagtttatatacttccaacgtctgcc

20 25 30 35 40
V T V Y V E K V Y K K G Y V T G F K Y M K V A D G
TP84_77

tgaaatccattccagattcaaagcgacattcgatgaattgctggatcaatacgaacgaaaaggatgataaaatat 46 050
actttaggtaaggtctaagtttcgctgtaagctacttaacgacctagttatgcttgcttttcctactattttata

45 50 55 60 1
E I H S R F K A T F D E L L D Q Y E R K G * M
TP84_77
TP84_78

ggatcgatgggaacgaatggcgaacgaacgcggccaggcgatccagaaagcgatccaaaaggaaagggggatgca 46 125
cctagctacccttgcttaccgcttgcttgcgccggtccgctaggtctttcgctaggttttcctttccccctacgt

5 10 15 20 25
W I D G N E W R T N A A R R S R K R S K R K G G C
TP84_78

gaatgggtggcaaaccatcgaagggaacgccgaaagatatgcgattgaaacggaacaggaaagcggcaggtctgc 46 200
cttacccaccgtttggtagcttcccttgcggctttctatacgctaactttgccttgtcctttcgccgtccagacg

30 35 40 45 50
R M G G K P S K G T P K D M R L K R N R K A A G L
TP84_78

cagtcggtccaggatcgagatcgaaaaagacaacaagtaaacgaaaataaggccaggggaaacctggtctttttt 46 275
gtcagccaggtcctagctctagctttttctgttgttcatttgcttttattccggtcccctttggaccagaaaaaa

55 60 65
P V G P G S R S K K T T S K R K *
TP84_78
rho-independent terminator

gcggacaaaacggacattttagaagaatataacaaaagcgcattcggctatggtaaaatatagacaagcctggcc 46 350
cgcctgttttgcctgtaaaatcttcttatattgttttcgcgtaagccgataccattttatatctgttcggaccgg

```
aggtaaaaaagaggaaaggggaagatcgaatggcaaaaacgacaacacaattccagtaccattccggcgtggggg    46 425
tccattttttctcctttccccttctagcttaccgtttttgctgttgtgttaaggtcatggtaaggccgcaccccc
                                1    5    10    15
                                M A K T T T Q F Q Y H S G V G
                                TP84_79 >

atgcaacatcctacattcacacatcggaagattacatgaatgtttatgtgacagtagaactgggccgtgtgggaa    46 500
tacgttgtaggatgtaagtgtgtagccttctaatgtacttacaaatacactgtcatcttgacccggcacaccctt
    20    25    30    35    40
D A T S Y I H T S E D Y M N V Y V T V E L G R V G
TP84_79 >

catgggaaacggaagcatggtgcaaattagctttacaacgatatgaaaacggcgcatggaaaacgatcgccacag    46 575
gtaccctttgccttcgtaccacgtttaatcgaaatgttgctatacttttgccgcgtaccttttgctagcggtgtc
    45    50    55    60    65
T W E T E A W C K L A L Q R Y E N G A W K T I A T
TP84_79 >

cgcaaggatatgccgcaacagggcaaaatctgaatcgaacattcagcaatatcagtaatgtgatggaaaagccaa    46 650
gcgttcctatacggcgttgtcccgttttagacttagcttgtaagtcgttatagtcattacactaccttttcggtt
    70    75    80    85    90
A Q G Y A A T G Q N L N R T F S N I S N V M E K P
TP84_79 >

tgcgtgtcaaagtggatctttatgcgaattcctcttattccgattatgtccaaactgtctacacaaaacagtgga    46 725
acgcacagtttcacctagaaatacgcttaaggagaataaggctaatacaggtttgacagatgtgttttgtcacct
    95    100    105    110    115
M R V K V D L Y A N S S Y S D Y V Q T V Y T K Q W
TP84_79 >

tcagataaggagtgaatgggcgacatggcgacaacttctaaagcgatcgaattgaatgataattttacttatggt    46 800
agtctattcctcacttacccgctgtaccgctgttgaagatttcgctagcttaacttactattaaaatgaatacca
            1    5    10    15    20
I R *       M G D M A T T S K A I E L N D N F T Y G
TP84_79     TP84_80 >

ggtactggataccttaacacatccgaagattatatgaatgtttatgccacctgggaaacgccatataatacttat    46 875
ccatgacctatggaattgtgtaggcttctaatatacttacaaatacggtggaccctttgcggtatattatgaata
    25    30    35    40    45
G T G Y L N T S E D Y M N V Y A T W E T P Y N T Y
TP84_80 >

gtttatgccagtatgacactgcaacgatatgaggatggcgtgtggaaaaacatcgaaacgaaaggtgcttatgca    46 950
caaatacggtcatactgtgacgttgctatactcctaccgcacacctttttgtagctttgctttccacgaatacgt
    50    55    60    65    70
V Y A S M T L Q R Y E D G V W K N I E T K G A Y A
TP84_80 >

tattacacacaccaagaacgcaaacacgctaatgtccaatttactaacatcgctaaaaaaggcacgccgatgagg    47 025
ataatgtgtgtggttcttgcgtttgtgcgattacaggttaaatgattgtagcgatttttttccgtgcggctactcc
    75    80    85    90    95
Y Y T H Q E R K H A N V Q F T N I A K K G T P M R
TP84_80 >
```

```
gtaaagcttaacttacatgacggaaacaatccgaatagcccatctatgcaaacagcttatagttatagctggaca   47 100
cattcgaattgaatgtactgcctttgttaggcttatcgggtagatacgtttgtcgaatatcaatatcgacctgt
          100       105       110       115       120
V  K  L  N  L  H  D  G  N  N  P  N  S  P  S  M  Q  T  A  Y  S  Y  S  W  T
TP84_80

agataaatcaacaaggtcgaaaaagatggatggacatcctggacattttccctggatgtccatttttattccat   47 175
tctatttagttgttccagctttttctacctacctgtaggacctgtaaaagggacctacaggtaaaaaataaggta
R  *
TP84_80
rho-independent terminator

gccgctgaatggttgtggtaaaatatagataagcaagtacagcccaaaatcccaccatgccgggtgtatagaata   47 250
cggcgacttaccaacaccattttatatctattcgttcatgtcgggttttagggtggtacggcccacatatcttat

ggcatggaccagtggcggatctggtgatcaaaaccgcatcaatgggtgtaagggagcgaaaacacctggcatcca   47 325
ccgtacctggtcaccgcctagaccactagttttggcgtagttacccacattccctcgcttttgtggaccgtaggt

gacaggccgcatagacggccaacaggtccgccgtgcctgggatcatagcagaaatggtccaaatcacggcgaatt   47 400
ctgtccggcgtatctgccggttgtccaggcggcacggaccctagtatcgtctttaccaggtttagtgccgcttaa
1       5
M  V  Q  I  T  A  N
TP84_81

tcactgcctgcatgttctgtctgtgggtggccgccacacaaaaaaaaacgagccaaaaacaaacaaaaggggatg   47 475
agtgacggacgtacaagacagacacccaccggcggtgtgtttttttttgctcggtttttgtttgttttcccctac
   10        15        20        25        30
F  T  A  C  M  F  C  L  W  V  A  A  T  Q  K  K  T  S  Q  K  Q  T  K  G  D
TP84_81

atctatggacagctaggcagtggacaaacaatcaaatcaaataaattcgtttcaccaggagtgagtggcgacaac   47 550
tagatacctgtcgatccgtcacctgtttgttagtttagtttatttaagcaaagtggtcctcactcaccgctgttg
   35        40        45
D  L  W  T  A  R  Q  W  T  N  N  Q  I  K  *
TP84_81

tgaaatgagaaggggaaagggatggatcgaacagggccgattcatccctttttatatggacatcctggacaaaat   47 625
actttactcttcccctttccctacctagcttgtcccggctaagtagggaaaaatatacctgtaggacctgtttta
rho-independent terminator

agtctagggtgtctttttttatgccaggaacaccacatatcgtgacaaaagacgacaaaaatctggtaaaatgaa   47 700
tcagatcccacagaaaaaaatacggtccttgtggtgtatagcactgttttctgctgtttttagaccattttactt
rho-independent terminator

cat  3'
        47 703
gta  5'
```
