# Supplementary material for: Sequence, genome organization, annotation and proteomics of the thermophilic, 47.7-kb Geobacillus stearothermophilus bacteriophage TP-84 and its classification in the new Tp84virus genus
Source: PLoS One. 2018 Apr 6;13(4):e0195449. doi: 10.1371/journal.pone.0195449 (PMC5889276; doi:10.1371/journal.pone.0195449)

This form should be used for all taxonomic proposals. Please complete all those modules that are applicable.
For guidance, see the notes written in blue and the separate document "Help with completing a taxonomic proposal"

Please try to keep related proposals within a single document.

Part 1: TITLE, AUTHORS, etc

**Code assigned:**	***2017.020B***	(to be completed by ICTV officers)

Short title: **To create a new genus, *Tp84virus*, within the family *Siphoviridae***

**Modules attached** (Modules 1, 4 and either 2 or 3 are required.	**1** ☒ **2** ☒ **3** ☐ **4** ☒

Author(s):

Piotr Skowron, University of Gdansk (Poland)
Andrew M. Kropinski, University of Guelph (Canada)
Jens H. Kuhn, National Institute of Allergy and Infectious Diseases (USA)
Evelien Adriaenssens, University of Liverpool (UK)
Marcin Los, University of Gdansk (Polland)

Corresponding author with e-mail address:

Andrew M. Kropinski, Phage.Canada@gmail.com

List the ICTV study group(s) that have seen this proposal:

A list of study groups and contacts is provided at http://www.ictvonline.org/subcommittees.asp . If in doubt, contact the appropriate subcommittee chair (there are six virus subcommittees: animal DNA and retroviruses, animal ssRNA-, animal ssRNA+, fungal and protist, plant, bacterial and archaeal)	**ICTV Bacterial and Archaeal Viruses Subcommittee**

ICTV Study Group comments (if any) and response of the proposer:

Date first submitted to ICTV: June 8, 2017
Date of this revision (if different to above):

ICTV-EC comments and response of the proposer:

Part 2: PROPOSED TAXONOMY

Present the proposed new taxonomy on accompanying spreadsheet

Name of accompanying spreadsheet: 2017.020B.N.v1.Tp84virus

Please display the taxonomic changes you are proposing on the accompanying spreadsheet 2017_TP_Template_Excel.xlsx. Submit both this and the accompanying spreadsheet to the appropriate ICTV Subcommittee Chair.

Part 4: **APPENDIX**: supporting material

-

References:

A. General

1. Darling AE, Mau B, Perna NT. progressiveMauve: multiple genome alignment with gene gain, loss and rearrangement. PLoS One. 2010; 5(6):e11147.
2. Turner D, Reynolds D, Seto D, Mahadevan P. CoreGenes3.5: a webserver for the determination of core genes from sets of viral and small bacterial genomes. BMC Res Notes. 2013; 6:140. doi: 10.1186/1756-0500-6-140.
3. Dereeper A, Guignon V, Blanc G, Audic S, Buffet S, Chevenet F, Dufayard JF, Guindon S, Lefort V, Lescot M, Claverie JM, Gascuel O. Phylogeny.fr: robust phylogenetic analysis for the non-specialist. Nucleic Acids Res. 2008; 36(Web Server issue):W465-9.
4. Agren J, Sundström A, Håfström T, Segerman B. Gegenees: fragmented alignment of multiple genomes for determining phylogenomic distances and genetic signatures unique for specified target groups. PLoS One. 2012;7(6):e39107.

B. This TaxoProp Specifically

5. Epstein I, Campbell LL. Production and purification of the thermophilic bacteriophage TP-84. Appl Microbiol. 1975;29(2):219-23.
6. Saunders GF, Campbell LL. Characterization of a thermophilic bacteriophage for Bacillus stearothermophilus. J Bacteriol. 1966;91(1):340-8.
7. Saunders GF, Campbell LL. Properties of the deoxyribonucleic acid of the thermophilic bacteriophage TP-84. Biochemistry. 1965;4(12):2836-44.
8. Bassel A, Shaw M, Campbell LL. Dissociation by chelating agents and substructure of the thermophilic bacteriophage TP84. J Virol. 1971;7: 663-672.

Introduction: Geobacillus phage TP-84 was discovered in 1952 in greenhouse soil using *Geobacillus stearothermophilus* strain 2184 as its host. The phage has a rather narrow host range, as it is lytic for only four out of 24 tested related thermophilic bacteria: *Geobacillus* (formerly *Bacillus*) *stearothermophilus*. Strains of *Bacillus (B.) subtilis*, *B. megaterium*, *B. pumilus*, *B. licheniformis*, and *B. coagulans*, as well as *Paenibacillus macerans* are resistant to Geobacillus phage TP-84 infection [5,6]. Electron microscopic evaluation of phage particles revealed an elongated head [8] with dimensions of 53 x 30 nm and a long noncontractile tail (3–5 nm wide by 131 nm in length). Geobacillus phage TP-84's double stranded (ds) genome contains 42% GC and is 13.9 μm long with a molecular weight of 22.4–27 MDa [7]. It was determined that the phage is sensitive to chelating agents, such as EDTA and phosphate, resulting in the dissociation of heads from tails and ghost structure formation [8].

The genome of Geobacillus phage TP-84 has recently been sequenced, revealing a 47.7-kbp double-stranded DNA genome (54.5 mol% G+C) encoding 85 polypeptides of 4 kDa or larger.

The genome has no sequence similarity to any other phage genome in GenBank. Interestingly, all the closest protein homologs (Fig. 2) are bacterial (possibly prophage) in origin.

It has been the subcommittee's approach not to propose genera for single phage isolates largely because of the time required to write them. In this case, we are making an exception since Drs. Skowron and Los are extensively studying this virus and have patented several of its proteins.

Species demarcation: We have chosen 95% DNA sequence identity as the criterion for demarcation of species in this new genus. The members of each of the proposed species differ from those of other species by more than 5% at the DNA level as confirmed with the BLASTN algorithm.

Genus demarcation: BLASTN and phylogenetic analyses (Fig. 2) [3] indicate that the proposed genus, *Tp84virus,* is cohesive and distinct from other genera. On average, the genomes of members of this genus are 47.7 kb in length (54.5 mol% G+C) and encode 82 proteins and 0 tRNAs.

The type species *Geobacillus virus Tp84* was chosen because it represents the first sequenced member of this genus.

The name of the new genus was based on that of its first sequenced member.

Figure 1. Negatively strained Geobacillus phage TP-84 particles (with permission from [8]). The bar represent 100 nm.

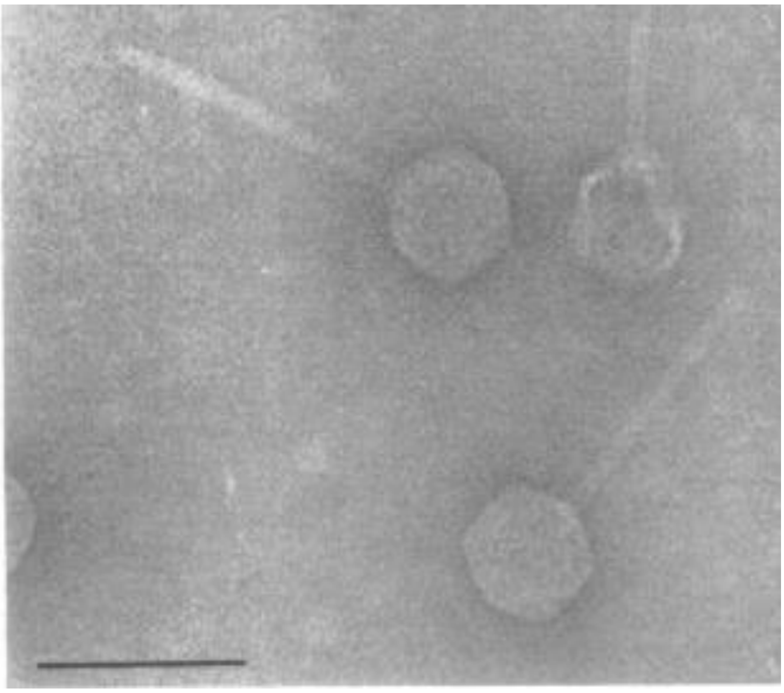

Table 1. Properties of the phages belonging to the genus *Tp84virus*.

Phage Name	RefSeq No.	GenBank accession No.	Genome length (kb)	%G+C	# proteins	# tRNA

Geobacillus phage TP-84	-	KY565347	47.7	54.5	82	0

Fig. 2. Phylogenetic analysis of (A) large subunit terminase proteins, and (B) thymidylate synthase proteins of Geobacillus phage TP-84 and related proteins constructed using "one click" at phylogeny.fr [3]. "The "One Click mode" targets users that do not wish to deal with program and parameter selection. By default, the pipeline is already set up to run and connect programs recognized for their accuracy and speed (MUSCLE for multiple alignment and PhyML for phylogeny) to reconstruct a robust phylogenetic tree from a set of sequences." It also includes the use of Gblocks to eliminate poorly aligned positions and divergent regions. "The usual bootstrapping procedure is replaced by a new confidence index that is much faster to compute. See: Anisimova M., Gascuel O. Approximate likelihood ratio test for branches: A fast, accurate and powerful alternative (Syst Biol. 2006;55(4):539-52.) for details".

A. TerL proteins, B. thymidylate synthase (Ts) proteins

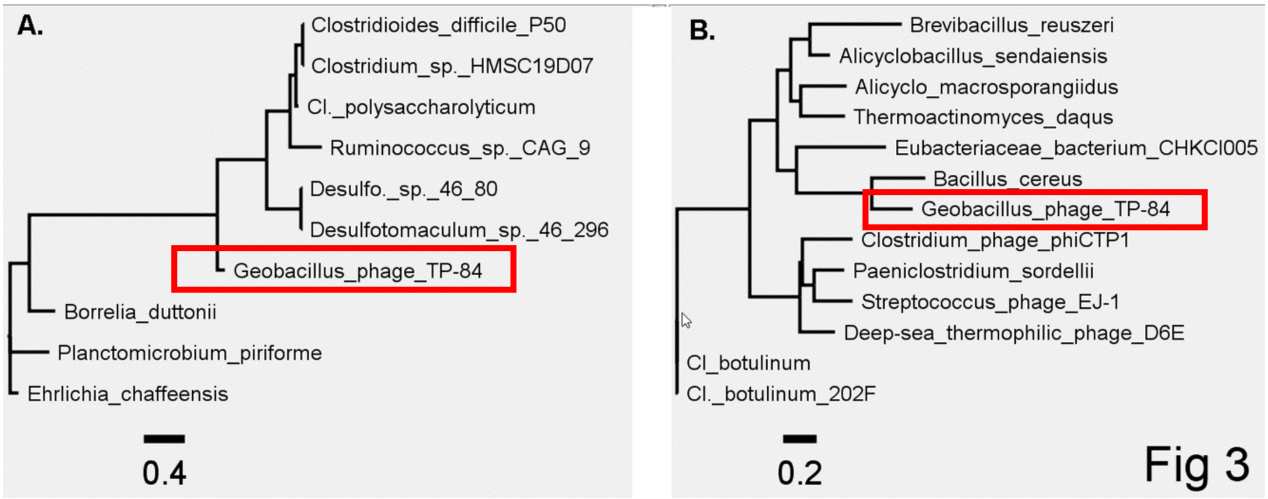

Supplement: S3 File — (PDF) [file pone.0195449.s003.pdf]
