# Supplementary material for: Sequence, genome organization, annotation and proteomics of the thermophilic, 47.7-kb Geobacillus stearothermophilus bacteriophage TP-84 and its classification in the new Tp84virus genus
Source: PLoS One. 2018 Apr 6;13(4):e0195449. doi: 10.1371/journal.pone.0195449 (PMC5889276; doi:10.1371/journal.pone.0195449)
Supplement: S4 File — (PDF) [file pone.0195449.s004.pdf]

S4

A.

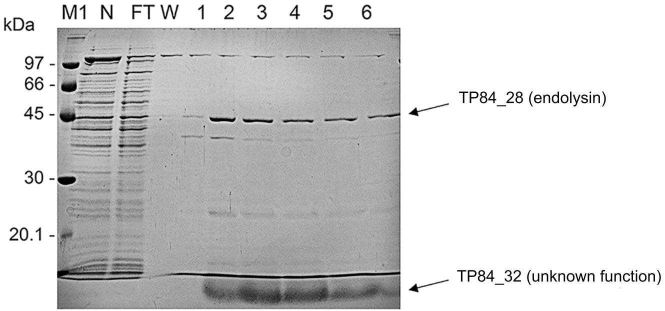

M1 N FT W 1 2 3 4 5 6
kDa
97 -
66 -
45 -
30 -
20.1 -
TP84_28 (endolysin)
TP84_32 (unknown function)

B.

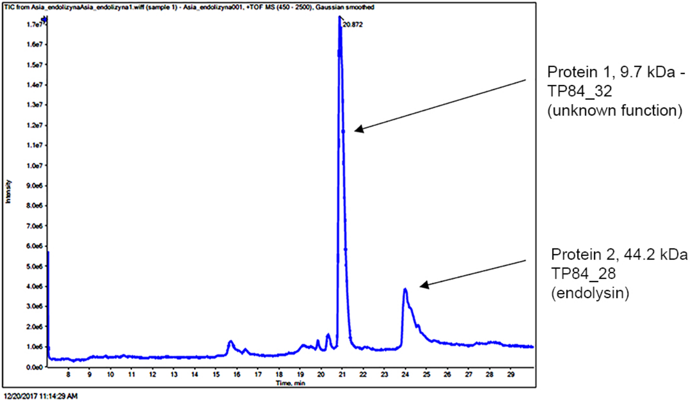

TIC from Asia_endolizynaAsia_endolizyna1.wiff (sample 1) - Asia_endolizyna001, +TOF MS (450 - 2500), Gaussian smoothed
20.872
Intensity
Time, min
Protein 1, 9.7 kDa - TP84_32 (unknown function)
Protein 2, 44.2 kDa TP84_28 (endolysin)
12/20/2017 11:14:29 AM

Result from Faculty of Chemistry:

PROTEIN VIEW: **TP84_28 (endolysin)**

SEQUENCE COVERAGE: 96.7%

Protein ID | Spectra | Summary Statistics

Proteins Detected

N	Unused	Total	% Cov	Accessio...	Name	Species	Peptides(95%)	Biological Processes	Molecular Functions	PANTHER ID
1	8.04	8.04	100.0	tr|A0A1U...	Uncharacterized protein OS=Geobacillus phage...	9VIRU	4			
2	6.53	6.53	92.5	tr|A0A1U...	Uncharacterized protein OS=Geobacillus phage...	9VIRU	2			
3	6.13	6.13	100.0	tr|A0A1U...	Uncharacterized protein OS=Geobacillus phage...	9VIRU	6			
4	5.00	5.00	53.1	tr|A0A1U...	Uncharacterized protein OS=Geobacillus phage...	9VIRU	3			
5	4.00	4.00	96.7	tr|A0A1U...	Endolysin OS=Geobacillus phage TP-84 PE=4...	9VIRU	2			
6	3.15	3.15	71.5	tr|A0A1U...	HNH homing endonuclease OS=Geobacillus phage TP-84 PE=4 SV=1		2			
7	2.00	2.00	94.4	tr|A0A1U...	Uncharacterized protein OS=Geobacillus phage...	9VIRU	1			
8	2.00	2.00	77.4	tr|A0A1U...	Uncharacterized protein OS=Geobacillus phage...	9VIRU	1			
[illegible]	[illegible]	[illegible]	[illegible]	[illegible]	[illegible]	[illegible]	[illegible]			

Protein Group 5 - Endolysin OS=Geobacillus phage TP-84 PE=4 SV=1

Proteins in Group

N	Unused	Total	Accessio...	Name	Species
5	4.00	4.00	tr|A0A1U...	Endolysin OS=Geob...	9VIRU

Peptides in Group

Con...	Conf	Sequence	Modifications	Cleavages	ΔMass	Prec MW	z	Sc	Spectrum	Type
2.00	99	QIGQTIKIPN			-0.0697	1110.57...	2	16	1.1.1.2945.4	Win...
2.00	99	TIKSGDTFW			-0.0671	1053.44...	2	12	1.1.1.3176.4	Win...
0.00	42.7	KVSGIAGNVDM	Dioxidation(M)@11		-0.0376	1121.50...	2	12	1.1.1.2744.3	Win...
0.00	0.2	NAREQARHFVNTVKGMPND...			-0.1872	2561.11...	4	5	1.1.1.3466.3	Win...
0.00	1.5	SVNPNALKVGQ	Deamidated(N)@5		-0.0616	1126.53...	2	9	1.1.1.2470.2	Win...
0.00	0.5	YGVDKPGDNPI			-0.0392	1173.52...	2	7	1.1.1.907.3	Win...
0.00	<1	AIAKYPVWIAH			-0.0888	1267.61...	3	6	1.1.1.1447.2	Win...
0.00	<1	AIAKYPVWIAH			-0.0850	1267.62...	3	6	1.1.1.1460.2	Win...
0.00	2.1	AKVEQPKQKV	Lys->Val@9		-0.0978	1124.55...	2	11	1.1.1.5042.4	Win...
0.00	0.4	AKVEQPKQKV	Lys->Val@9		-0.0978	1124.55...	2	10	1.1.1.5034.2	Win...
0.00	<1	AKVKAAGIQVAYLKATEGT...	Cation:K(E)@17		-0.1986	4975.43...	7	9	1.1.1.8050.3	Win...
0.00	0.2	ALDIETTEGLSNEALTKCAI	Thr->Arg@7		-0.0894	2146.00...	4	10	1.1.1.4349.2	Win...
0.00	0.5	ALKVGQVI	Gly->Gln@5		-0.0661	897.4987	2	9	1.1.1.3527.2	Win...
0.00	0.2	ALKVGQVI	Gly->Gln@5		-0.0671	897.4977	2	9	1.1.1.3534.2	Win...
0.00	<1	ANNIKGIDVSHWQGKIDWA...	Deamidated(N)@3		0.1180	4805.65...	7	6	1.1.1.7917.2	Win...
0.00	<1	ANNIKGIDVSHWQGKIDWA...	Deamidated(N)@3		-0.2822	5034.39...	7	7	1.1.1.7919.2	Win...
0.00	0.8	DIFVDAAKVEQPKQK	Cation:K(D)@5<br>Deamidated(Q)@14		-0.0272	1753.83...	4	11	1.1.1.2697.2	Win...

Protein Sequence Coverage - Endolysin OS=Geobacillus phage TP-84 PE=4 SV=1

MQARSANNIKGIDVSHWQGKIDWAKVKAAGIQVAYLKATEGTTHVDKMLKTNYQNAKKAGIKVGFYHFFRAKNEQNAREQARHFVNTVKGMPNDLKHALDIETTEGLSNEALTKCAIAFLEEVKKLTGQDPIVYTYTSFARSRLTAAIAKYPVWIAHYGVDKPGDNPIWDRWIGFQYTDKGKVSGIAGNVDMNEFTSDIFVDAAK
VEQPKQKVDAVQSTPSATGTYTIKSGDTFWELEEKYNWPHGTLQRLNPSVNPNALKVGQVIKVPKSEQPKQNASSVTGTYTIKSGDTFWDLEQKNGWPHGTLQKLNPGVNPNKLKVGQVIKVPKSEQKNVQRTVKNHQKPNYRTYKIKKGDTFWELEKKNGWPHGTLQKLNPGVNPAKLQIGQTIKIPN

Result from Faculty of Chemistry:

PROTEIN VIEW: **TP84_32**

SEQUENCE COVERAGE: 100%

Protein ID | Spectra | Summary Statistics

Proteins Detected

N	Unused	Total	% Cov	Accessio...	Name	Species	Peptides(95%)	Biological Processes	Molecular Functions	PANTHER ID
1	24.78	24.78	100.0	tr|A0A1U...	Uncharacterized protein OS=Geobacillus phage...	9VIRU	15			
2	16.78	16.78	100.0	tr|A0A1U...	HNH homing endonuclease OS=Geobacillus ph...	9VIRU	10			
3	16.64	16.64	97.8	tr|A0A1U...	Uncharacterized protein OS=Geobacillus phage...	9VIRU	10			
4	15.08	15.08	100.0	tr|A0A1U...	Uncharacterized protein OS=Geobacillus phage...	9VIRU	7			
5	14.58	14.58	100.0	tr|A0A1U...	Uncharacterized protein OS=Geobacillus phage...	9VIRU	13			
6	10.57	10.57	100.0	tr|A0A1U...	Uncharacterized protein OS=Geobacillus phage...	9VIRU	6			
7	9.37	9.37	100.0	tr|A0A1U...	Uncharacterized protein OS=Geobacillus phage...	9VIRU	5			
8	7.32	7.32	100.0	tr|A0A1U...	Endolysin OS=Geobacillus phage TP-84 PE=4...	9VIRU	5			
[illegible]	[illegible]	[illegible]	[illegible]	[illegible]	[illegible]	[illegible]	[illegible]			

Protein Group 5 - Uncharacterized protein OS=Geobacillus phage TP-84 PE=4 SV=1

Proteins in Group

N	Unused	Total	Accessio...	Name	S
5	14.58	14.58	tr|A0A1U...	Uncharacterized protein OS=Geobacillus phage TP-84 PE=4 SV=1	9

Peptides in Group

Con...	Conf	Sequence	Modifications	Cleavages	ΔMass	Prec MW	z	Sc	Spectrum	Type
2.00	99	AVIEAIQTIK			-0.0711	1084.57...	2	16	3.1.1.2831.3	Win...
2.00	99	AVIEAIQTIKEK			-0.0859	1341.70...	3	15	6.1.1.2778.3	Win...
2.00	99	ITGVVIETR			-0.0620	986.5141	2	14	6.1.1.2257.4	Win...
2.00	99	ITGVVIETRTM			-0.0782	1218.58...	2	18	1.1.1.3364.5	Win...
2.00	99	MEIKTLPIKW			-0.0813	1257.63...	2	17	4.1.1.5044.2	Win...
2.00	99	TMKGSAGLPKAVIEAIQTI...	Biotin(K)@22		-0.0738	2595.33...	5	25	1.1.1.7698.2	Win...
2.00	99	TMKGSAGLPKAVIEAIQTI...			-0.1644	2595.33...	5	18	1.1.1.7692.2	Win...
0.44	80.3	VIRKNGSDEFQVD			-0.0930	1505.65...	3	12	1.1.1.2103.2	Win...
0.12	43	AIQTIKEKGIL			-0.0766	1212.66...	3	11	1.1.1.2868.2	Win...
0.02	9.1	TLPIKW			-0.0460	756.4075	2	7	3.1.1.2981.2	Win...
0.00	0.6	FQVDRMDK			-0.0326	1037.46...	2	8	5.1.1.3621.2	Win...
0.00	0.6	GVVIETR			-0.0557	772.3887	2	5	5.1.1.946.2	Win...
0.00	< 1	AGLPKAVIEAIQT			-0.1624	1309.59...	3	3	2.1.1.1215.2	Win...
0.00	< 1	AGLPKAVIEAIQTI			-0.1651	1422.67...	3	5	3.1.1.3717.2	Win...
0.00	< 1	AGLPKAVIEAIQTIKE	Ala->Ser@10		-0.0101	1695.96...	3	6	6.1.1.905.3	Win...
0.00	1.4	AIQTIKE	Gln->Leu@3		-0.0363	786.4489	2	8	2.1.1.2473.2	Win...
0.00	0.1	AIQTIKEKGIL			-0.0763	1212.66...	3	7	1.1.1.2863.2	Win...
[illegible]	[illegible]	[illegible]			[illegible]	[illegible]	[illegible]	[illegible]	[illegible]	[illegible]

Protein Sequence Coverage - Uncharacterized protein OS=Geobacillus phage TP-84 PE=4 SV=1

MEIKTLPIKWSVPTNYITGVVIETRTMIYVIRKNGSDEFQVDRMDKNHKWNRYWTIDRFTMKGSAGLPKAVIEAIQTIKEKGIL
