# Supplementary material for: Sequence, genome organization, annotation and proteomics of the thermophilic, 47.7-kb Geobacillus stearothermophilus bacteriophage TP-84 and its classification in the new Tp84virus genus
Source: PLoS One. 2018 Apr 6;13(4):e0195449. doi: 10.1371/journal.pone.0195449 (PMC5889276; doi:10.1371/journal.pone.0195449)
Supplement: S5 File — (PDF) [file pone.0195449.s005.pdf]

MASCOT Search Results

Protein View: TP84_06

**Database:**	TP84
**Score:**	5396
**Nominal mass (M_r):**	62259
**Calculated pI:**	4.96

Sequence similarity is available as **an NCBI BLAST search of 61. against nr**.

Search parameters

**Enzyme:**	Trypsin: cuts C-term side of KR unless next residue is P.
**Fixed modifications:**	**Carbamidomethyl (C)**
**Variable modifications:**	**Oxidation (M)**

Protein sequence coverage: 75%

Matched peptides shown in ***bold red***.

```
  1 MGIKWTKWST NVIKKYHGNI QKYRKLYDGD HAKLFERAKR LIQEGEITDQ
 51 IIEGAEVARN VKTPYIVANV CKMIVDIPAM LVSRAIGQVT TSMSPDDFAG
101 MVTDQGDGTV ISLYEKQKEL IKGIAKRSNL QFEHKTNIIH HQMDGGIVGM
151 PFDDENGLRI EFKSRDVYYP HPDGRGCDLV YQLEIEDEET EEAIKYLHVY
201 RERVEEQKLV TQHMLYKIGE SGMLEEIEDE AEVKEILGIE KTYREFEGRD
251 KPFVVYWPNN KTFTHPLGRS ELYNLAGKQD EINWTLTRNA IVYERNGKPR
301 IAVSKEIFQA LQDKAFERYG DENKIDHRDL EIVTFDENGK AMEVIQIDVS
351 KIGDIKWVKD LMKLMLMETH TSEKAVDFYL EGNTSAQSGI AKFYDLFVSI
401 MKAEQIATEY VHFLQELFEN CLWIAHQDDP DIVIEEPRIQ IKDMIPISRR
451 ELIEQESTAY KNGTQSLETT VRNQNPTATE DWIEDELAAI EESQQSTDTT
501 SILMGRQTLS NLLDNRNPNG TPIGAAQQQP QQGTPQTGGG QA
```

Unformatted sequence string: **542 residues** (for pasting into other applications).

Sort peptides by ◉ Residue Number ○ Increasing Mass ○ Decreasing Mass

Show predicted peptides also

Query	Start – End	Observed	Mr(expt)	Mr(calc)	ppm	M	Score	Expect	Rank	U	Peptide
920	8 – 14	424.2339	846.4533	846.4599	-7.84	0	37	0.00021	1	U	K.WSTNVIK.K
1002	16 – 22	430.2211	858.4277	858.4348	-8.27	0	38	0.00017	1	U	K.YHGNIQK.Y
2567	25 – 33	349.5102	1045.5088	1045.5192	-9.98	1	39	0.00011	1	U	R.KLYDGDHAK.L
2568	25 – 33	349.5106	1045.5101	1045.5192	-8.78	1	44	4e-05	1	U	R.KLYDGDHAK.L
1456	26 – 33	459.7164	917.4183	917.4243	-6.52	0	47	1.8e-05	1	U	K.LYDGDHAK.L
8894	41 – 59	695.3564	2083.0473	2083.0797	-15.5	0	46	2.3e-05	1	U	R.LIQEGEITDQIIEGAEVAR.N
8896	41 – 59	695.3588	2083.0547	2083.0797	-12.0	0	45	2.9e-05	1	U	R.LIQEGEITDQIIEGAEVAR.N
8898	41 – 59	695.3610	2083.0613	2083.0797	-8.84	0	53	4.8e-06	1	U	R.LIQEGEITDQIIEGAEVAR.N
8899	41 – 59	695.3617	2083.0632	2083.0797	-7.89	0	45	3.1e-05	1	U	R.LIQEGEITDQIIEGAEVAR.N
8900	41 – 59	695.3620	2083.0643	2083.0797	-7.39	0	78	1.8e-08	1	U	R.LIQEGEITDQIIEGAEVAR.N
8901	41 – 59	695.3640	2083.0701	2083.0797	-4.59	0	31	0.00074	1	U	R.LIQEGEITDQIIEGAEVAR.N
8902	41 – 59	695.3642	2083.0709	2083.0797	-4.20	0	83	5.6e-09	1	U	R.LIQEGEITDQIIEGAEVAR.N
8903	41 – 59	695.3645	2083.0717	2083.0797	-3.84	0	109	1.3e-11	1	U	R.LIQEGEITDQIIEGAEVAR.N
8904	41 – 59	695.3650	2083.0731	2083.0797	-3.14	0	51	8.5e-06	1	U	R.LIQEGEITDQIIEGAEVAR.N
8905	41 – 59	695.3655	2083.0747	2083.0797	-2.40	0	68	1.5e-07	1	U	R.LIQEGEITDQIIEGAEVAR.N
8907	41 – 59	1042.5472	2083.0798	2083.0797	0.080	0	133	4.5e-14	1	U	R.LIQEGEITDQIIEGAEVAR.N
8908	41 – 59	1042.5492	2083.0838	2083.0797	2.00	0	142	6.8e-15	1	U	R.LIQEGEITDQIIEGAEVAR.N
8909	41 – 59	1042.5499	2083.0852	2083.0797	2.67	0	136	2.4e-14	1	U	R.LIQEGEITDQIIEGAEVAR.N
8910	41 – 59	1042.5502	2083.0858	2083.0797	2.96	0	146	2.3e-15	1	U	R.LIQEGEITDQIIEGAEVAR.N
3582	63 – 72	582.8038	1163.5929	1163.6009	-6.81	0	86	2.7e-09	1	U	K.TPYIVANVCK.M
5027	73 – 84	672.8694	1343.7242	1343.7305	-4.65	0	97	1.9e-10	1	U	K.MIVDIPAMLVSR.A
5123	73 – 84	680.8664	1359.7182	1359.7254	-5.29	0	81	7.5e-09	1	U	K.MIVDIPAMLVSR.A + Oxidation (M)
5124	73 – 84	680.8673	1359.7201	1359.7254	-3.93	0	78	1.7e-08	1	U	K.MIVDIPAMLVSR.A + Oxidation (M)
5125	73 – 84	680.8674	1359.7203	1359.7254	-3.73	0	82	7e-09	1	U	K.MIVDIPAMLVSR.A + Oxidation (M)
5230	73 – 84	688.8634	1375.7123	1375.7203	-5.81	0	48	1.7e-05	1	U	K.MIVDIPAMLVSR.A + 2 Oxidation (M)
11963	85 – 116	1111.8592	3332.5558	3332.5429	3.87	0	114	4.2e-12	1	U	R.AIGQVTTSMSPDDFAGMVTDQGDGTVISI
12015	85 – 116	1117.1900	3348.5482	3348.5378	3.10	0	100	9.5e-11	1	U	R.AIGQVTTSMSPDDFAGMVTDQGDGTVISI + Oxidation (M)
12016	85 – 116	1117.1901	3348.5485	3348.5378	3.19	0	98	1.7e-10	1	U	R.AIGQVTTSMSPDDFAGMVTDQGDGTVISI + Oxidation (M)
12037	85 – 116	1122.5237	3364.5493	3364.5327	4.92	0	130	1e-13	1	U	R.AIGQVTTSMSPDDFAGMVTDQGDGTVIS + 2 Oxidation (M)
2195	128 – 135	501.7498	1001.4851	1001.4930	-7.95	0	34	0.00043	1	U	R.SNLQFEHK.T
11011	136 – 159	667.3112	2665.2155	2665.2377	-8.32	0	37	0.00018	1	U	K.TNIIHHQMDGGIVGMPFDDENGLR.I
11012	136 – 159	889.4154	2665.2245	2665.2377	-4.95	0	62	5.9e-07	1	U	K.TNIIHHQMDGGIVGMPFDDENGLR.I
11068	136 – 159	675.3065	2697.1969	2697.2276	-11.4	0	34	0.00036	1	U	K.TNIIHHQMDGGIVGMPFDDENGLR.I + 2 Oxidation (M)

5689	164 - 175	487.8943	1460.6610	1460.6797	-12.8	1	50	1.1e-05	1	U	K.SRDVYYPHPDGR.G
5691	164 - 175	487.8949	1460.6628	1460.6797	-11.5	1	49	1.4e-05	1	U	K.SRDVYYPHPDGR.G
5692	164 - 175	487.8951	1460.6636	1460.6797	-11.0	1	43	5.3e-05	1	U	K.SRDVYYPHPDGR.G
5693	164 - 175	366.1741	1460.6672	1460.6797	-8.53	1	32	0.00067	1	U	K.SRDVYYPHPDGR.G
5694	164 - 175	487.8985	1460.6737	1460.6797	-4.11	1	32	0.0007	1	U	K.SRDVYYPHPDGR.G
3981	166 - 175	406.8518	1217.5335	1217.5465	-10.7	0	32	0.00059	1	U	R.DVYYPHPDGR.G
3982	166 - 175	406.8524	1217.5354	1217.5465	-9.15	0	36	0.00027	1	U	R.DVYYPHPDGR.G
3983	166 - 175	609.7759	1217.5372	1217.5465	-7.63	0	50	1.1e-05	1	U	R.DVYYPHPDGR.G
940	196 - 201	425.7283	849.4421	849.4497	-8.99	0	36	0.00026	1	U	K.YLHVYR.E
941	196 - 201	425.7283	849.4421	849.4497	-8.97	0	41	7.3e-05	1	U	K.YLHVYR.E
942	196 - 201	425.7286	849.4427	849.4497	-8.22	0	37	0.00022	1	U	K.YLHVYR.E
1446	202 - 208	459.2335	916.4525	916.4614	-9.64	1	46	2.3e-05	1	U	R.ERVEEQK.L
1447	202 - 208	459.2337	916.4529	916.4614	-9.24	1	47	1.8e-05	1	U	R.ERVEEQK.L
1448	202 - 208	459.2339	916.4531	916.4614	-8.98	1	39	0.00012	1	U	R.ERVEEQK.L
1449	202 - 208	459.2339	916.4532	916.4614	-8.90	1	46	2.3e-05	1	U	R.ERVEEQK.L
1450	202 - 208	459.2339	916.4532	916.4614	-8.87	1	56	2.4e-06	1	U	R.ERVEEQK.L
3264	209 - 217	566.8074	1131.6003	1131.6110	-9.50	0	66	2.4e-07	1	U	K.LVTQHMLYK.I
3265	209 - 217	378.2075	1131.6007	1131.6110	-9.13	0	45	3.5e-05	1	U	K.LVTQHMLYK.I
3405	209 - 217	383.5390	1147.5951	1147.6060	-9.42	0	41	7.4e-05	1	U	K.LVTQHMLYK.I + Oxidation (M)
3406	209 - 217	574.8057	1147.5968	1147.6060	-7.94	0	68	1.4e-07	1	U	K.LVTQHMLYK.I + Oxidation (M)
8004	218 - 234	626.6249	1876.8530	1876.8611	-4.36	0	38	0.00017	1	U	K.IGESGMLEEIEDEAEVK.E
8005	218 - 234	939.4372	1876.8598	1876.8611	-0.73	0	128	1.7e-13	1	U	K.IGESGMLEEIEDEAEVK.E
8060	218 - 234	947.4342	1892.8537	1892.8561	-1.22	0	122	7e-13	1	U	K.IGESGMLEEIEDEAEVK.E + Oxidation (M)
8061	218 - 234	947.4345	1892.8544	1892.8561	-0.90	0	124	4.4e-13	1	U	K.IGESGMLEEIEDEAEVK.E + Oxidation (M)
10994	218 - 241	887.4444	2659.3114	2659.3149	-1.33	1	151	7.7e-16	1	U	K.IGESGMLEEIEDEAEVKEILGIEK.T
10995	218 - 241	887.4448	2659.3127	2659.3149	-0.85	1	88	1.5e-09	1	U	K.IGESGMLEEIEDEAEVKEILGIEK.T
11029	218 - 241	892.7738	2675.2996	2675.3098	-3.84	1	138	1.8e-14	1	U	K.IGESGMLEEIEDEAEVKEILGIEK.T + Oxidation (M)
11030	218 - 241	892.7740	2675.3001	2675.3098	-3.65	1	44	3.6e-05	1	U	K.IGESGMLEEIEDEAEVKEILGIEK.T + Oxidation (M)
551	235 - 241	401.2359	800.4573	800.4643	-8.83	0	46	2.4e-05	1	U	K.EILGIEK.T
5938	250 - 261	502.9250	1505.7533	1505.7667	-8.88	0	48	1.4e-05	1	U	R.DKPFVVYWPNNK.T
5942	250 - 261	753.8876	1505.7606	1505.7667	-4.05	0	68	1.6e-07	1	U	R.DKPFVVYWPNNK.T
1508	262 - 269	464.7490	927.4835	927.4927	-9.87	0	50	9.9e-06	1	U	K.TFTHPLGR.S
1509	262 - 269	310.1687	927.4843	927.4927	-8.98	0	36	0.00023	1	U	K.TFTHPLGR.S
2119	270 - 278	497.7599	993.5052	993.5131	-7.90	0	41	8.6e-05	1	U	R.SELYNLAGK.Q
4452	279 - 288	638.3161	1274.6176	1274.6255	-6.19	0	69	1.3e-07	1	U	K.QDEINWTLTR.N
1046	289 - 295	432.7289	863.4432	863.4501	-7.92	0	51	7.7e-06	1	U	R.NAIVYER.N
2960	306 - 314	546.2863	1090.5581	1090.5659	-7.14	0	53	4.6e-06	1	U	K.EIFQALQDK.A
5243	329 - 340	690.3338	1378.6531	1378.6616	-6.17	0	75	3.4e-08	1	U	R.DLEIVTFDENGK.A
4087	341 - 351	616.8267	1231.6389	1231.6482	-7.57	0	57	2.2e-06	1	U	K.AMEVIQIDVSK.I
4088	341 - 351	616.8274	1231.6403	1231.6482	-6.40	0	53	5.1e-06	1	U	K.AMEVIQIDVSK.I
4089	341 - 351	616.8278	1231.6410	1231.6482	-5.85	0	66	2.8e-07	1	U	K.AMEVIQIDVSK.I
4855	364 - 374	660.3137	1318.6127	1318.6261	-10.1	0	40	0.0001	1	U	K.LMLMETHTSEK.A
4856	364 - 374	440.5449	1318.6129	1318.6261	-9.97	0	68	1.6e-07	1	U	K.LMLMETHTSEK.A
4857	364 - 374	660.3161	1318.6175	1318.6261	-6.47	0	81	7.9e-09	1	U	K.LMLMETHTSEK.A
4957	364 - 374	445.8764	1334.6074	1334.6210	-10.2	0	42	5.7e-05	1	U	K.LMLMETHTSEK.A + Oxidation (M)
4958	364 - 374	668.3134	1334.6122	1334.6210	-6.62	0	69	1.3e-07	1	U	K.LMLMETHTSEK.A + Oxidation (M)
5063	364 - 374	451.2121	1350.6146	1350.6159	-0.98	0	58	1.7e-06	1	U	K.LMLMETHTSEK.A + 2 Oxidation (M)
7957	375 - 392	935.9611	1869.9076	1869.9108	-1.75	0	98	1.5e-10	1	U	K.AVDFYLEGNTSAQSGIAK.F
7958	375 - 392	935.9675	1869.9204	1869.9108	5.10	0	68	1.5e-07	1	U	K.AVDFYLEGNTSAQSGIAK.F
4358	393 - 402	631.8249	1261.6353	1261.6417	-5.08	0	60	9.6e-07	1	U	K.FYDLFVSIMK.A
4469	393 - 402	639.8227	1277.6308	1277.6366	-4.52	0	53	4.5e-06	1	U	K.FYDLFVSIMK.A + Oxidation (M)
4927	439 - 449	443.9202	1328.7387	1328.7486	-7.45	1	59	1.4e-06	1	U	R.IQIKDMIPISR.R + Oxidation (M)
769	443 - 449	416.2214	830.4282	830.4320	-4.57	0	32	0.00062	1	U	K.DMIPISR.R
5733	450 - 461	489.5834	1465.7283	1465.7412	-8.81	1	55	3.3e-06	1	U	R.RELIEQESTAYK.N
4768	451 - 461	655.8233	1309.6320	1309.6401	-6.18	0	66	2.3e-07	1	U	R.ELIEQESTAYK.N
3903	462 - 472	603.3045	1204.5944	1204.6048	-8.63	0	89	1.2e-09	1	U	K.NGTQSLETTVR.N
12349	473 - 506	949.1882	3792.7235	3792.7272	-0.96	0	171	7.7e-18	1	U	R.NQNPTATEDWIEDELAAIEESQQSTDTTS
12350	473 - 506	1265.2561	3792.7465	3792.7272	5.08	0	134	3.9e-14	1	U	R.NQNPTATEDWIEDELAAIEESQQSTDTTS
12364	473 - 506	953.1865	3808.7168	3808.7221	-1.40	0	155	3e-16	1	U	R.NQNPTATEDWIEDELAAIEESQQSTDTTS + Oxidation (M)
12365	473 - 506	1270.5871	3808.7395	3808.7221	4.56	0	150	1.1e-15	1	U	R.NQNPTATEDWIEDELAAIEESQQSTDTTS + Oxidation (M)
3658	507 - 516	587.3105	1172.6064	1172.6149	-7.25	0	84	4.2e-09	1	U	R.QTLSNLLDNR.N
10566	517 - 542	835.0654	2502.1745	2502.1848	-4.12	0	96	2.7e-10	1	U	R.NPNGTPIGAAQQQPQQGTPQTGGGQA.-

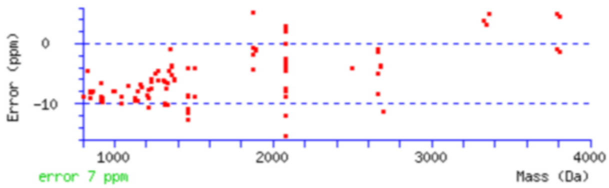

RMS error 7 ppm

PROTEIN VIEW: **TP84_8**

SEQUENCE COVERAGE: 97.9%

Protein ID | Spectra | Summary Statistics

Proteins Detected

N	Unused	Total	% Cov	Accessio...	Name	Species	Peptides(95%)	Biological Processes	Molecular Functions	PANTHER ID
1	6.00	6.00	85.9	tr|A0A1U...	Uncharacterized protein OS=Geobacillus phage...	9VIRU	4			
2	4.02	4.02	100.0	tr|A0A1U...	Uncharacterized protein OS=Geobacillus phage...	9VIRU	2			
3	2.34	2.34	99.1	tr|A0A1U...	Tail-length tape-measure protein OS=Geobacillu...	9VIRU	2			
4	1.16	1.16	92.7	tr|A0A1U...	Major capsid protein OS=Geobacillus phage TP-...	9VIRU	1			
5	0.41	0.41	97.9	tr|A0A1U...	Minor capsid protein OS=Geobacillus phage TP-...	9VIRU	0			
6	0.28	0.28	98.0	tr|A0A1U...	Distal tail protein OS=Geobacillus phage TP-84...	9VIRU	0			
7	0.14	0.14	72.2	tr|A0A1U...	Uncharacterized protein OS=Geobacillus phage...	9VIRU	0			

Protein Group 5 - Minor capsid protein OS=Geobacillus phage TP-84 PE=4 SV=1

Proteins in Group

N	Unused	Total	Accessio...	Name	Species
5	0.41	0.41	tr|A0A1U...	Minor capsid protein...	9VIRU

Peptides in Group

Con...	Conf	Sequence	Modifications	Cleavages	ΔMass	Prec MW	z	Sc	Spectrum	Type
0.25	69.8	FPVAGIDVPLP			0.0977	1123.72...	2	11	1.1.1.1187.5	Win...
0.11	46.9	IIDIIASVGGVD			0.1263	1170.77...	3	11	1.1.1.646.3	Win...
0.01	6.5	TLPMLKQ			0.0913	829.5645	2	7	1.1.1.520.6	Win...
0.01	5.8	NKQIHLAG			0.0482	879.5409	2	9	1.1.1.622.4	Win...
0.01	3.9	FLLDQI			0.0116	747.4283	2	5	1.1.1.631.2	Win...
0.01	3.7	SEYLIRTLPMLK			0.0782	1462.89...	3	9	1.1.1.627.8	Win...
0.00	1.5	VELCRQAIIDII			0.0806	1384.85...	2	9	1.1.1.1338.8	Win...
0.00	61.9	IGEYALLK	Tyr->Phe@4		0.0158	889.5431	2	9	1.1.1.604.6	Win...
0.00	0.9	LCRQAIIDI			0.0743	1043.65...	2	7	1.1.1.1067.3	Win...
0.00	0.6	KVSIRK			0.0283	729.5145	2	5	1.1.1.586.2	Win...
0.00	21.1	TLPKGPK	Oxidation(P)@3		0.0659	755.5201	2	7	1.1.1.346.2	Win...
0.00	32.1	HLAGIESIVSD	Asp->Glu@11		0.0799	1153.67...	2	12	1.1.1.969.8	Win...
0.00	0.2	IDIIASVGGV			0.0507	942.5893	2	4	1.1.1.959.6	Win...
0.00	0.2	LCRQAIIDIIASVGGVDNLL			0.0252	2082.17...	3	8	1.1.1.1131.6	Win...
0.00	0.3	LVNAIAARMQQLGAD			0.1226	1569.95...	3	8	1.1.1.970.6	Win...
0.00	0.3	NREIRL			-0.0086	799.4579	2	3	1.1.1.960.2	Win...
0.00	0.2	QIERQIVELCRQAIID			0.0243	1926.06...	3	8	1.1.1.1190.5	Win...
[illegible]	[illegible]	[illegible]			[illegible]	[illegible]	[illegible]	[illegible]	[illegible]	[illegible]

Protein Sequence Coverage - Minor capsid protein OS=Geobacillus phage TP-84 PE=4 SV=1

MANMDQIERQIVELCRQAIIDIIASVGGVDNLLDDERRMALVNAIAARMQQLGADISAILPAELAKAYYQGITDADAFLHQEGTEVTVQGVNKQIHLAGIESIVSDTMMDMQAAIRTAVAMAIADIDRILVDVRNDIAKGMILGDPSREITKRVMQTFANSGMTAFITKDNKRLPLDFYAMTVTRTKMRQAHTDGAVNRYKENGV
YHVKISEHGITCNKCARYQGLVVALDPEHAEGFPVAGIDVPLPPYHPNCRHTVRPFVMEYHSQNDIRNEKRKWKSFDPEGDPRTDAQKRLYKAEQDIRRKARQEMKEYMLMKATLPPDQVPKTLAAYRRMKRKNDAQWQKLQAQFKQALGEVDLQGPPPGTPRKRKGAKTGPGSSTPKTDKTDKKQNVSTLSTLPKGPKLTEADL
AAHKEFDKRIDGIEFADRRQTARFLLDQIPGGNQIKVSIRKIEANGHVGKFSVVGGKFIIGEYALLKDDPRPPQYRWKTVFHEYYHAQMHGMDYDDHFQRDNQEWTIWEETATECAAFFMAQRAGWNVSTILPSYSEYLIRTLPMLKQLPEYADCETMVDFGAKFMKYRFNEEHKTMEWRWLVRRVEQNADVFDINEYFREHYLE
HIKANKDRYADMIYDSLSQSNKFTHESVIRQMIKEGLESGIRFGTVNREIRLCLPVAMNELGVKKP

MASCOT Search Results

Protein View: TP84_11

**Database:**	TP84
**Score:**	743
**Nominal mass (M_r):**	14016
**Calculated pI:**	5.84

Sequence similarity is available as **an NCBI BLAST search of 66. against nr**.

Search parameters

**Enzyme:**	Trypsin: cuts C-term side of KR unless next residue is P.
**Fixed modifications:**	**Carbamidomethyl (C)**
**Variable modifications:**	**Oxidation (M)**

Protein sequence coverage: 57%

Matched peptides shown in ***bold red***.

```
  1 MNLQPKIVSI AGQKEFLATT QGLVHKVGGV TLDASKFTPD ENGFIKAGSA
 51 LALTASGKAE PFNVSTPGDP STANGTPYIL AHDVQIKDGT TNIDAVAGVL
101 EAAYLKSSVV TTAEPGRVVV TQDFIDASNG RFHLR
```

Unformatted sequence string: **135 residues** (for pasting into other applications).

Sort peptides by ● Residue Number ○ Increasing Mass ○ Decreasing Mass

Show predicted peptides also

Query	Start - End	Observed	Mr(expt)	Mr(calc)	ppm	M	Score	Expect	Rank	U	Peptide
464	7 - 14	408.2500	814.4855	814.4912	-7.09	0	56	2.5e-06	1	U	K.IVSIAGQK.E
3485	15 - 26	448.5794	1342.7163	1342.7245	-6.12	0	51	7.5e-06	1	U	K.EFLATTQGLVHK.V
3486	15 - 26	672.3656	1342.7167	1342.7245	-5.79	0	78	1.5e-08	1	U	K.EFLATTQGLVHK.V
3487	15 - 26	448.5795	1342.7168	1342.7245	-5.72	0	55	3.2e-06	1	U	K.EFLATTQGLVHK.V
1047	27 - 36	473.7585	945.5025	945.5131	-11.2	0	74	3.9e-08	1	U	K.VGGVTLDASK.F
1048	27 - 36	473.7586	945.5026	945.5131	-11.1	0	61	8.3e-07	1	U	K.VGGVTLDASK.F
1049	27 - 36	473.7602	945.5058	945.5131	-7.69	0	74	3.8e-08	1	U	K.VGGVTLDASK.F
2322	37 - 46	584.2825	1166.5504	1166.5608	-8.92	0	48	1.6e-05	1	U	K.FTPDENGFIK.A
2323	37 - 46	584.2848	1166.5551	1166.5608	-4.91	0	58	1.6e-06	1	U	K.FTPDENGFIK.A
1595	47 - 58	523.7925	1045.5704	1045.5768	-6.03	0	87	2e-09	1	U	K.AGSALALTASGK.A
1596	47 - 58	523.7927	1045.5709	1045.5768	-5.63	0	103	5.3e-11	1	U	K.AGSALALTASGK.A
1893	107 - 117	552.2841	1102.5537	1102.5619	-7.37	0	37	0.00021	1	U	K.SSVVTTAEPGR.V
1894	107 - 117	552.2845	1102.5545	1102.5619	-6.67	0	31	0.00085	1	U	K.SSVVTTAEPGR.V
4553	118 - 131	760.8833	1519.7521	1519.7631	-7.21	0	99	1.2e-10	1	U	R.VVVTQDFIDASNGR.F

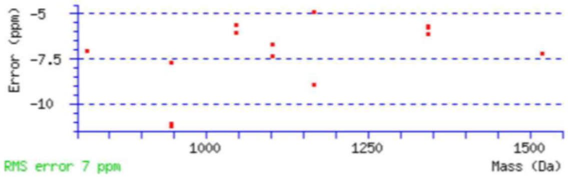

Mascot: **http://www.matrixscience.com/**

MASCOT Search Results

Protein View: TP84_12

**Database:**	TP84
**Score:**	2668
**Nominal mass (M_r):**	37757
**Calculated pI:**	4.98

Sequence similarity is available as an NCBI BLAST search of 67. against nr.

Search parameters

**Enzyme:**	Trypsin: cuts C-term side of KR unless next residue is P.
**Fixed modifications:**	Carbamidomethyl (C)
**Variable modifications:**	Oxidation (M)

Protein sequence coverage: 73%

Matched peptides shown in ***bold red***.

```
  1 MPLHLEQFQR EAFQGYVENV PPKREYALAK FMPNQPVYDI EFTYNIINGG
 51 YGQMASITAW DSGAPLRDKD VIQRLTAQIA KVQHAYRLTE KELLMFHRPR
101 MDEEQQQVIQ AIYNNTDKLV WGVQDREEWL RAKAVYVGQL QYSENDVQLN
151 IDFLIPAENK LTADVDWSDP TAPVIQHLQS AVQRFKEANN GEKPVEMHMS
201 SRVETWLLQN EQVKAHIYGN TTDPRIVTSE QLQQLFSALS LPPYRVIDEQ
251 VIGENGAEAL MPEDRVVLLG EELGHTMEGP TVENNYKPGI YVIPEIKETN
301 PPRQEVYVGK SVFPALERPQ AVVHLIVAQS
```

Unformatted sequence string: 330 residues (for pasting into other applications).

Sort peptides by ◉ Residue Number ○ Increasing Mass ○ Decreasing Mass

Show predicted peptides also

Query	Start – End	Observed	Mr(expt)	Mr(calc)	ppm	M	Score	Expect	Rank	U	Peptide
3650	2 – 10	389.8767	1166.6082	1166.6196	-9.76	0	53	5.6e-06	1	U	M.PLHLEQFQR.E
3651	2 – 10	584.3115	1166.6084	1166.6196	-9.60	0	55	3e-06	1	U	M.PLHLEQFQR.E
5774	11 – 23	739.3656	1476.7166	1476.7249	-5.58	0	51	7.9e-06	1	U	R.EAFQGYVENVPPK.R
5775	11 – 23	739.3662	1476.7179	1476.7249	-4.75	0	39	0.00012	1	U	R.EAFQGYVENVPPK.R
5776	11 – 23	739.3664	1476.7182	1476.7249	-4.49	0	37	0.00019	1	U	R.EAFQGYVENVPPK.R
6847	11 – 24	545.2763	1632.8072	1632.8260	-11.5	1	70	9.1e-08	1	U	R.EAFQGYVENVPPKR.E
6848	11 – 24	545.2767	1632.8084	1632.8260	-10.8	1	78	1.5e-08	1	U	R.EAFQGYVENVPPKR.E
6849	11 – 24	545.2768	1632.8085	1632.8260	-10.7	1	62	6.2e-07	1	U	R.EAFQGYVENVPPKR.E
6850	11 – 24	545.2769	1632.8090	1632.8260	-10.4	1	55	2.9e-06	1	U	R.EAFQGYVENVPPKR.E
6851	11 – 24	545.2771	1632.8095	1632.8260	-10.1	1	54	4e-06	1	U	R.EAFQGYVENVPPKR.E
6852	11 – 24	545.2774	1632.8103	1632.8260	-9.64	1	46	2.2e-05	1	U	R.EAFQGYVENVPPKR.E
6853	11 – 24	545.2774	1632.8105	1632.8260	-9.49	1	74	3.9e-08	1	U	R.EAFQGYVENVPPKR.E
6856	11 – 24	545.2782	1632.8127	1632.8260	-8.13	1	70	1e-07	1	U	R.EAFQGYVENVPPKR.E
6857	11 – 24	545.2784	1632.8134	1632.8260	-7.69	1	70	1e-07	1	U	R.EAFQGYVENVPPKR.E
6859	11 – 24	817.4168	1632.8190	1632.8260	-4.30	1	111	8e-12	1	U	R.EAFQGYVENVPPKR.E
6860	11 – 24	817.4198	1632.8250	1632.8260	-0.58	1	120	9.4e-13	1	U	R.EAFQGYVENVPPKR.E
1180	68 – 74	437.2388	872.4631	872.4716	-9.70	1	43	4.5e-05	1	U	R.DKDVIQR.L
223	75 – 81	372.7308	743.4470	743.4541	-9.56	0	48	1.5e-05	1	U	R.LTAQIAK.V
405	82 – 87	387.2025	772.3905	772.3980	-9.71	0	35	0.00033	1	U	K.VQHAYR.L
3893	92 – 100	400.2174	1197.6303	1197.6441	-11.5	0	37	0.00018	1	U	K.ELLMFHRPR.M
3894	92 – 100	400.2181	1197.6325	1197.6441	-9.67	0	36	0.00025	1	U	K.ELLMFHRPR.M
3990	92 – 100	405.5501	1213.6284	1213.6390	-8.75	0	35	0.00033	1	U	K.ELLMFHRPR.M + Oxidation (M)
9341	101 – 118	722.9996	2165.9769	2165.9899	-5.99	0	82	6.2e-09	1	U	R.MDEEQQQVIQAIYNNTDK.L
9342	101 – 118	1084.0045	2165.9944	2165.9899	2.11	0	92	5.9e-10	1	U	R.MDEEQQQVIQAIYNNTDK.L
9399	101 – 118	728.3298	2181.9675	2181.9848	-7.94	0	78	1.5e-08	1	U	R.MDEEQQQVIQAIYNNTDK.L + Oxidation (M)
9400	101 – 118	1091.9997	2181.9848	2181.9848	0.021	0	110	1e-11	1	U	R.MDEEQQQVIQAIYNNTDK.L + Oxidation (M)
2003	119 – 126	486.7614	971.5082	971.5189	-10.9	0	50	1.1e-05	1	U	K.LVWGVQDR.E
7202	119 – 131	562.6228	1684.8466	1684.8685	-13.0	1	37	0.0002	1	U	K.LVWGVQDREEWLR.A
7206	119 – 131	562.6261	1684.8566	1684.8685	-7.10	1	46	2.6e-05	1	U	K.LVWGVQDREEWLR.A
11905	134 – 160	1027.5221	3079.5445	3079.5502	-1.86	0	71	8.1e-08	1	U	K.AVYVGQLQYSENDVQLNIDFLIPAENK.I
11021	161 – 184	883.1184	2646.3333	2646.3402	-2.59	0	131	8.7e-14	1	U	K.LTADVDWSDPTAPVIQHLQSAVQR.F
11022	161 – 184	1324.1805	2646.3464	2646.3402	2.36	0	137	2.1e-14	1	U	K.LTADVDWSDPTAPVIQHLQSAVQR.F
5830	203 – 214	496.2633	1485.7681	1485.7827	-9.85	0	47	2.2e-05	1	U	R.VETWLLQNEQVK.A
5831	203 – 214	743.8944	1485.7741	1485.7827	-5.78	0	65	3.1e-07	1	U	R.VETWLLQNEQVK.A
4189	215 – 225	415.5348	1243.5827	1243.5945	-9.53	0	54	4.1e-06	1	U	K.AHIYGNTTDPR.I
4190	215 – 225	622.8006	1243.5866	1243.5945	-6.40	0	40	9.8e-05	1	U	K.AHIYGNTTDPR.I
9950	226 – 245	764.0837	2289.2292	2289.2369	-3.37	0	97	2.2e-10	1	U	R.IVTSEQLQQLFSALSLPPYR.V
9408	246 – 265	1093.0080	2184.0014	2184.0368	-16.2	0	67	2.2e-07	1	U	R.VIDEQVIGENGAEALMPEDR.V
9409	246 – 265	729.0116	2184.0130	2184.0368	-10.9	0	98	1.7e-10	1	U	R.VIDEQVIGENGAEALMPEDR.V
9455	246 – 265	734.3419	2200.0039	2200.0318	-12.7	0	110	9.7e-12	1	U	R.VIDEQVIGENGAEALMPEDR.V + Oxidation (M)
9456	246 – 265	1101.0229	2200.0312	2200.0318	-0.23	0	101	8.2e-11	1	U	R.VIDEQVIGENGAEALMPEDR.V + Oxidation (M)
9457	246 – 265	1101.0282	2200.0418	2200.0318	4.59	0	122	7.1e-13	1	U	R.VIDEQVIGENGAEALMPEDR.V + Oxidation (M)
12335	266 – 297	1180.2896	3537.8470	3537.8429	1.16	0	84	3.7e-09	1	U	R.VVLLGEELGHTMEGPTVENNYKPGIYVIE
12336	266 – 297	1180.2921	3537.8545	3537.8429	3.28	0	89	1.3e-09	1	U	R.VVLLGEELGHTMEGPTVENNYKPGIYVIE
12387	266 – 297	1185.6232	3553.8478	3553.8378	2.81	0	56	2.3e-06	1	U	R.VVLLGEELGHTMEGPTVENNYKPGIYVII + Oxidation (M)
5984	298 – 310	506.2582	1515.7528	1515.7681	-10.1	1	31	0.00086	1	U	K.ETNPPRQEVYVGK.S
5985	298 – 310	758.8886	1515.7626	1515.7681	-3.66	1	65	3.3e-07	1	U	K.ETNPPRQEVYVGK.S
758	304 – 310	411.7176	821.4207	821.4283	-9.28	0	39	0.00011	1	U	R.QEVYVGK.S

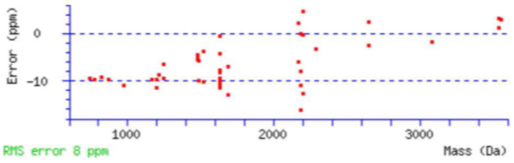

MASCOT Search Results

Protein View: TP84_13

**Database:**	TP84
**Score:**	972
**Nominal mass (M_r):**	7214
**Calculated pI:**	9.19

Sequence similarity is available as an NCBI BLAST search of 68. against nr.

Search parameters

**Enzyme:**	No enzyme cleavage specificity.
**Fixed modifications:**	Carbamidomethyl (C)
**Variable modifications:**	Oxidation (M)

Protein sequence coverage: 29%

Matched peptides shown in ***bold red***.

```
 1 MPKYIAKRHL VTRTGIKKPG DVIEYTKEQA QKLLAAGFIE EAEEEKKTTS
51 KGKQKADEAD KVDK
```

Unformatted sequence string: 64 residues (for pasting into other applications).

Sort peptides by ● Residue Number ○ Increasing Mass ○ Decreasing Mass

Query	Start – End	Observed	Mr(expt)	Mr(calc)	ppm	M	Score	Expect	Rank	U	Peptide
4788	33 – 46	774.8815	1547.7484	1547.7719	-15.2	0	81	2.1e-08	1	U	K.LLAAGFIEEAEEEK.K
4789	33 – 46	774.8832	1547.7518	1547.7719	-12.9	0	94	1.1e-09	1	U	K.LLAAGFIEEAEEEK.K
4790	33 – 46	774.8856	1547.7567	1547.7719	-9.81	0	88	4.7e-09	1	U	K.LLAAGFIEEAEEEK.K
5287	33 – 47	559.6265	1675.8578	1675.8668	-5.36	0	90	3.3e-09	1	U	K.LLAAGFIEEAEEEKK.T
5288	33 – 47	559.6268	1675.8585	1675.8668	-4.95	0	99	4.7e-10	1	U	K.LLAAGFIEEAEEEKK.T
5289	33 – 47	559.6269	1675.8589	1675.8668	-4.70	0	114	1.5e-11	1	U	K.LLAAGFIEEAEEEKK.T
5290	33 – 47	838.9371	1675.8597	1675.8668	-4.26	0	99	4.3e-10	1	U	K.LLAAGFIEEAEEEKK.T
5291	33 – 47	838.9377	1675.8609	1675.8668	-3.54	0	112	2.4e-11	1	U	K.LLAAGFIEEAEEEKK.T
5292	33 – 47	838.9377	1675.8609	1675.8668	-3.52	0	97	7.9e-10	1	U	K.LLAAGFIEEAEEEKK.T
5293	33 – 47	559.6276	1675.8611	1675.8668	-3.41	0	32	0.0024	1	U	K.LLAAGFIEEAEEEKK.T
5294	33 – 47	838.9381	1675.8617	1675.8668	-3.03	0	123	2.1e-12	1	U	K.LLAAGFIEEAEEEKK.T
6519	33 – 51	524.2758	2093.0741	2093.0892	-7.20	0	37	0.00068	1	U	K.LLAAGFIEEAEEEKKTTSK.G
6520	33 – 51	524.2761	2093.0753	2093.0892	-6.62	0	32	0.0019	1	U	K.LLAAGFIEEAEEEKKTTSK.G
6522	33 – 51	524.2764	2093.0766	2093.0892	-6.01	0	36	0.00076	1	U	K.LLAAGFIEEAEEEKKTTSK.G
6523	33 – 51	698.7007	2093.0804	2093.0892	-4.19	0	71	2.1e-07	1	U	K.LLAAGFIEEAEEEKKTTSK.G
6524	33 – 51	698.7010	2093.0812	2093.0892	-3.80	0	30	0.0029	1	U	K.LLAAGFIEEAEEEKKTTSK.G

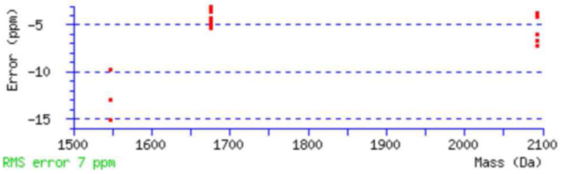

MASCOT Search Results

Protein View: TP84_16

**Database:**	TP84
**Score:**	499
**Nominal mass (M_r):**	18714
**Calculated pI:**	6.26

Sequence similarity is available as **an NCBI BLAST search of 71. against nr**.

Search parameters

**Enzyme:**	Trypsin: cuts C-term side of KR unless next residue is P.
**Fixed modifications:**	**Carbamidomethyl (C)**
**Variable modifications:**	**Oxidation (M)**

Protein sequence coverage: 26%

Matched peptides shown in ***bold red***.

1 VARDHIFSFE WEGIDELVEL LENMDRKTRR IMVQEYTK**FG** **LLVEEGARAL**
51 **APKDEGNLED** **SINAEK**AKII GEGVEVEVGV GSVYGLRRHE EPPRIGKYPK
101 YERGAKFPNY YVNGLGARTR SKPGWRGEKP GRKYLQR**AVE** **LVVDDFDQMN**
151 **ER**ILERIMEG RR

Unformatted sequence string: **162 residues** (for pasting into other applications).

Sort peptides by ◉ Residue Number ○ Increasing Mass ○ Decreasing Mass

Show predicted peptides also

Query	Start – End	Observed	Mr(expt)	Mr(calc)	ppm	M	Score	Expect	Rank	U	Peptide
2703	39 – 48	545.7963	1089.5781	1089.5818	-3.40	0	85	3.2e-09	1	U	K.FGLLVEEGAR.A
7273	49 – 66	638.6504	1912.9294	1912.9377	-4.35	1	78	1.5e-08	1	U	R.ALAPKDEGNLEDSINAEK.A
4894	54 – 66	717.3201	1432.6256	1432.6317	-4.31	0	99	1.1e-10	1	U	K.DEGNLEDSINAEK.A
4895	54 – 66	717.3209	1432.6272	1432.6317	-3.18	0	90	9.4e-10	1	U	K.DEGNLEDSINAEK.A
6688	138 – 152	890.4152	1778.8159	1778.8145	0.76	0	115	3.3e-12	1	U	R.AVELVVDDFDQMNER.I
6759	138 – 152	898.4135	1794.8125	1794.8094	1.71	0	96	2.6e-10	1	U	R.AVELVVDDFDQMNER.I + Oxidation (M)

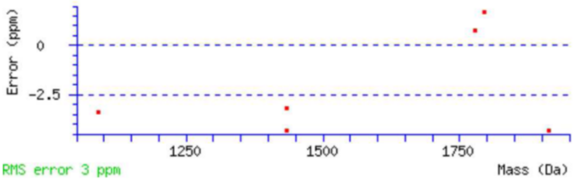

Mascot: **http://www.matrixscience.com/**

MASCOT Search Results

Protein View: TP84_18

**Database:**	TP84
**Score:**	1209
**Nominal mass (M_r):**	20371
**Calculated pI:**	4.38

Sequence similarity is available as **an NCBI BLAST search of 73. against nr**.

Search parameters

**Enzyme:**	Trypsin: cuts C-term side of KR unless next residue is P.
**Fixed modifications:**	**Carbamidomethyl (C)**
**Variable modifications:**	**Oxidation (M)**

Protein sequence coverage: 82%

Matched peptides shown in ***bold red***.

```
  1 MPGVNIPFGL ATITVGDPQD PNKIVFDGVN YFQAEGGELN IEPELEPIEL
 51 QDFGATPYDE RINGYTCELT IVVGQNDLKL MRKLFAYHSE IVDEGTGEVI
101 GLTDEKIGAS MRDKAVPVTI HPREMGSDTS LDIHIYKMSG VGAFNRTYEN
151 AQGSYEVTLR AYPRDGADPT KPGNFYYIGD TDPNA
```

Unformatted sequence string: **185 residues** (for pasting into other applications).

Sort peptides by ● Residue Number ○ Increasing Mass ○ Decreasing Mass

Show predicted peptides also

Query	Start - End	Observed	Mr(expt)	Mr(calc)	ppm	M	Score	Expect	Rank	U	Peptide
8987	1 - 23	799.7445	2396.2116	2396.2046	2.91	0	54	3.8e-06	1	U	-.MPGVNIPFGLATITVGDPQDPNK.I + Oxidation (M)
8465	2 - 23	750.7300	2249.1683	2249.1692	-0.41	0	83	4.7e-09	1	U	M.PGVNIPFGLATITVGDPQDPNK.I
8466	2 - 23	1125.6010	2249.1874	2249.1692	8.10	0	39	0.00013	1	U	M.PGVNIPFGLATITVGDPQDPNK.I
11269	24 - 61	1079.2712	4313.0557	4313.0328	5.31	0	88	1.4e-09	1	U	K.IVFDGVNYFQAEGGELNIEPELEPIELQD
11271	24 - 61	1438.6947	4313.0623	4313.0328	6.83	0	49	1.3e-05	1	U	K.IVFDGVNYFQAEGGELNIEPELEPIELQD
9525	83 - 106	663.3347	2649.3099	2649.3174	-2.82	1	56	2.6e-06	1	U	R.KLFAYHSEIVDEGTGEVIGLTDEK.I
9526	83 - 106	663.3350	2649.3107	2649.3174	-2.52	1	68	1.5e-07	1	U	R.KLFAYHSEIVDEGTGEVIGLTDEK.I
9527	83 - 106	884.1132	2649.3179	2649.3174	0.21	1	95	3.3e-10	1	U	R.KLFAYHSEIVDEGTGEVIGLTDEK.I
9529	83 - 106	884.1155	2649.3248	2649.3174	2.79	1	143	4.6e-15	1	U	R.KLFAYHSEIVDEGTGEVIGLTDEK.I
9240	84 - 106	841.4061	2521.1965	2521.2224	-10.3	0	31	0.00086	1	U	K.LFAYHSEIVDEGTGEVIGLTDEK.I
9242	84 - 106	841.4157	2521.2253	2521.2224	1.13	0	150	1e-15	1	U	K.LFAYHSEIVDEGTGEVIGLTDEK.I
2061	115 - 123	495.2960	988.5775	988.5818	-4.34	0	63	5.2e-07	1	U	K.AVPVTIHPR.E
2062	115 - 123	330.5334	988.5783	988.5818	-3.56	0	47	1.9e-05	1	U	K.AVPVTIHPR.E
6132	124 - 137	542.2536	1623.7391	1623.7450	-3.67	0	73	5.5e-08	1	U	R.EMGSDTSLDIHIYK.M + Oxidation (M)
1578	138 - 146	469.7265	937.4384	937.4440	-5.93	0	59	1.1e-06	1	U	K.MSGVGAFNR.T
1723	138 - 146	477.7241	953.4336	953.4389	-5.55	0	68	1.6e-07	1	U	K.MSGVGAFNR.T + Oxidation (M)
1724	138 - 146	477.7256	953.4366	953.4389	-2.41	0	69	1.4e-07	1	U	K.MSGVGAFNR.T + Oxidation (M)
6167	147 - 160	815.8885	1629.7624	1629.7634	-0.65	0	53	5.5e-06	1	U	R.TYENAQGSYEVTLR.A
8389	165 - 185	743.3296	2226.9671	2226.9706	-1.56	0	62	5.8e-07	1	U	R.DGADPTKPGNFYYIGDTDPNA.-
8390	165 - 185	1114.4942	2226.9738	2226.9706	1.47	0	53	5.2e-06	1	U	R.DGADPTKPGNFYYIGDTDPNA.-
8391	165 - 185	1114.4973	2226.9800	2226.9706	4.26	0	67	2.1e-07	1	U	R.DGADPTKPGNFYYIGDTDPNA.-

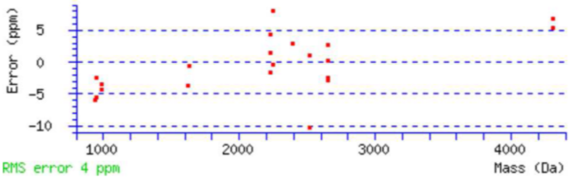

Mascot: **http://www.matrixscience.com/**

MASCOT Search Results

Protein View: TP84_19

Database: TP84
Score: 540
Nominal mass (M_r): 19335
Calculated pI: 4.90

Sequence similarity is available as **an NCBI BLAST search of 74. against nr**.

Search parameters

Enzyme: Trypsin: cuts C-term side of KR unless next residue is P.
Fixed modifications: **Carbamidomethyl (C)**
Variable modifications: **Oxidation (M)**

Protein sequence coverage: 57%

Matched peptides shown in ***bold red***.

1 MAKVTLTIKN GNVK**ESQQFE** **IDKITTFQAL** **K**LKNEIHAIL KDLKNNGELK
51 EVMEGLFSGE FDVDNMDIK**N** **ITADQLEQMK** **DEKFITSLAG** **AFDRLLETVP**
101 **ERAMNLLSIM** **SGIDREVLEK** **AYLEELFDVY** **DAVMEENDII** **K**LIDRMKRSF
151 FTTK**GQWSQA** **LR**TFLANK

Unformatted sequence string: **168 residues** (for pasting into other applications).

Sort peptides by ◉ Residue Number ○ Increasing Mass ○ Decreasing Mass

Show predicted peptides also

Query	Start – End	Observed	Mr(expt)	Mr(calc)	ppm	M	Score	Expect	Rank	U	Peptide
7868	15 – 31	676.0195	2025.0366	2025.0419	-2.57	1	90	1.1e-09	1	U	K.ESQQFEIDKITTFQALK.L
6481	70 – 83	560.2681	1677.7825	1677.7879	-3.22	1	58	1.8e-06	1	U	K.NITADQLEQMKDEK.F + Oxidation (M)
6482	70 – 83	839.9018	1677.7891	1677.7879	0.70	1	72	7e-08	1	U	K.NITADQLEQMKDEK.F + Oxidation (M)
3481	84 – 94	599.3144	1196.6142	1196.6190	-3.97	0	83	5e-09	1	U	K.FITSLAGAFDR.L
1673	95 – 102	478.7723	955.5300	955.5338	-3.96	0	38	0.00018	1	U	R.LLETVPER.A
5024	103 – 115	710.8695	1419.7245	1419.7214	2.22	0	92	6.7e-10	1	U	R.AMNLLSIMSGIDR.E
7848	103 – 120	673.6908	2018.0505	2018.0540	-1.72	1	60	1e-06	1	U	R.AMNLLSIMSGIDREVLEK.A
9422	121 – 141	840.4042	2518.1908	2518.1825	3.32	0	115	3.5e-12	1	U	K.AYLEELFDVYDAVMEENDIIK.L
1587	155 – 162	473.2453	944.4761	944.4828	-7.13	0	39	0.00014	1	U	K.GQWSQALR.T

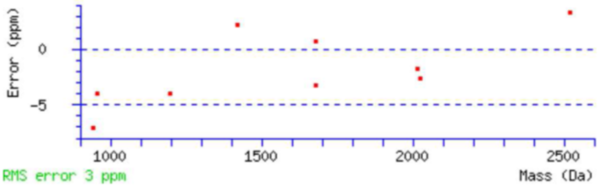

Mascot: **http://www.matrixscience.com/**

MASCOT Search Results

Protein View: TP84_21

Database: TP84
Score: 344
Nominal mass (M_r): 93056
Calculated pI: 9.70

Sequence similarity is available as **an NCBI BLAST search of 76. against nr**.

Search parameters

Enzyme: Trypsin: cuts C-term side of KR unless next residue is P.
Fixed modifications: **Carbamidomethyl (C)**
Variable modifications: **Oxidation (M)**

Protein sequence coverage: 9%

Matched peptides shown in ***bold red***.

```
  1 VATIRELRAK FTATANGFKS AIQGIKKDIQ SLNDASNKAA DNMNSRFSNL
 51 KSTLVGFAGA YLGFEALKGG ITSVATAMIQ GNANMEQYHA TLTTVLKSSE
101 KATEMLAWAE KFAASTPFEI PDIVEATTKL EVYGISAKET LKDIGDMAAI
151 TGKPLMQAVE AIADAQTGEL ERLKEFGITK QMLIDKAQEL YGKEIVNAKG
201 QITDMETMNK ALFAIMRERY SGGMEYISKT FNGMLANIKD SMGTIAAELG
251 KPIFEKLKGQ MEDLVPIMSA FTSFIRGDMS GAMKTLTEAF GANKAQQIMS
301 FFQTIKNAGM GIKDFFVSLA PTVQNIGTIL GNIAPIIIGP LVIAFKAIAA
351 VLPPVLNTIT GIVAKFTEWE GFIPLVTGLA AAMAVFKAQT VATQIVTTVA
401 TRATQMWAAA QRLLNLAMSM NPMTLIIGLI VGLVAALVMA YQRSETFRNI
451 VNNAWQSIKS VVSAVINWFV TTIPQWVENV KNWFVGLGQR ISETWSNIKN
501 GIVSIWQNII GTVVPIVQSF VQAIVERAKQ IATNVMNFIR PLIDFFKNTW
551 NNIKLLVLSI VGVFLNLLVG NFEGLKISLL GIWTAIKNQV INIATTIKSM
601 AVNIFTALKN GVLAVINGLK SLAISAWNGL KSAAISVWTG LKNGVVNTVQ
651 ALKNTAINIV NSVKSGVVNA FNSAKNLAIS AWNALKSGVS NAINSVKSLV
701 SNMKNNIIST IKGINLFEMG KNVIQGFIKG IKSMVGAVGK AIKEVASNVT
751 NKIKSALGIH SPSRVLMEIG AYTGQGFAIG IENMKKAVVN ATQSLADATI
801 GTISSAELNP TEPQVAVAAA TGAQTNYNAP LMYVENQYVN DNTDVRDISH
851 GLYNLQRRSD RKKGW
```

Unformatted sequence string: **865 residues** (for pasting into other applications).

Sort peptides by ◉ Residue Number ○ Increasing Mass ○ Decreasing Mass

Show predicted peptides also

Query	Start - End	Observed	Mr(expt)	Mr(calc)	ppm	M	Score	Expect	Rank	U	Peptide
79	20 - 26	358.7157	715.4169	715.4228	-8.27	0	51	8.3e-06	1	U	K.SAIQGIK.K
9245	112 - 129	646.3284	1935.9633	1935.9830	-10.1	0	57	2.1e-06	1	U	K.FAASTPFEIPDIVEATTK.L
9246	112 - 129	968.9989	1935.9833	1935.9830	0.18	0	81	8.8e-09	1	U	K.FAASTPFEIPDIVEATTK.L
2186	130 - 138	490.2718	978.5291	978.5386	-9.69	0	72	5.7e-08	1	U	K.LEVYGISAK.E
13178	143 - 172	1038.8554	3113.5444	3113.5373	2.28	0	83	5.5e-09	1	U	K.DIGDMAAITGKPLMQAVEAIADAQTGEL[illegible]
1771	173 - 180	468.2765	934.5385	934.5488	-10.9	1	46	2.7e-05	1	U	R.LKEFGITK.Q
3677	643 - 653	571.8264	1141.6382	1141.6455	-6.44	0	33	0.00051	1	U	K.NGVVNTVQALK.N

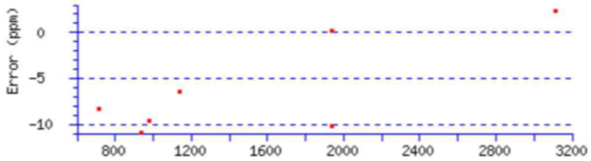

RMS error 7 ppm

Mascot: **http://www.matrixscience.com/**

MASCOT Search Results

Protein View: TP84_22

Database: TP84
Score: 936
Nominal mass (M_r): 27864
Calculated pI: 5.15

Sequence similarity is available as an NCBI BLAST search of 77. against nr.

Search parameters

Enzyme: Trypsin: cuts C-term side of KR unless next residue is P.
Fixed modifications: Carbamidomethyl (C)
Variable modifications: Oxidation (M)

Protein sequence coverage: 42%

Matched peptides shown in ***bold red***.

1 M**PMGYTFGGR HIHEFLVEMT GKNVPMTPPI K**NLSEEQGGI DGGWDFGIQY
51 EPK**IITIDHY ILAK**TREERQ NMIR**ELAGWL NPR**LGAR**ELI FDDEPDKMYY**
101 **ARLSEQFALE K**VIGTYSDFS LNFICYDPFT YSVQEYTQNI TGSGQIEHLG
151 THVSKPILIV DHRGGSATIT NQTQDGQTQT VTFASTTPPG IFTIDMKEGT
201 VKLGTQSGEK **YIDSIEWFEL EQGMNTITHS GNINQITVK**Y RHTWL

Unformatted sequence string: 245 residues (for pasting into other applications).

Sort peptides by ◉ Residue Number ○ Increasing Mass ○ Decreasing Mass

Show predicted peptides also

Query	Start - End	Observed	Mr(expt)	Mr(calc)	ppm	M	Score	Expect	Rank	U	Peptide
2185	2 - 10	501.2222	1000.4298	1000.4437	-13.9	0	53	4.8e-06	1	U	M.PMGYTFGGR.H + Oxidation (M)
5554	11 - 22	486.2417	1455.7032	1455.7180	-10.2	0	41	7.6e-05	1	U	R.HIHEFLVEMTGK.N + Oxidation (M)
5555	11 - 22	486.2419	1455.7038	1455.7180	-9.75	0	49	1.4e-05	1	U	R.HIHEFLVEMTGK.N + Oxidation (M)
5557	11 - 22	486.2452	1455.7137	1455.7180	-2.99	0	45	2.9e-05	1	U	R.HIHEFLVEMTGK.N + Oxidation (M)
2142	23 - 31	498.7740	995.5334	995.5474	-14.0	0	47	1.9e-05	1	U	K.NVPMTPPIK.N
2300	23 - 31	506.7723	1011.5300	1011.5423	-12.1	0	44	3.8e-05	1	U	K.NVPMTPPIK.N + Oxidation (M)
4523	54 - 64	433.9220	1298.7441	1298.7598	-12.1	0	68	1.7e-07	1	U	K.IITIDHYILAK.T
4524	54 - 64	650.3807	1298.7468	1298.7598	-10.0	0	75	3.2e-08	1	U	K.IITIDHYILAK.T
2632	75 - 83	528.2795	1054.5445	1054.5560	-10.8	0	55	3.2e-06	1	U	R.ELAGWLNPR.L
3941	88 - 97	610.7814	1219.5483	1219.5608	-10.2	0	63	5.2e-07	1	U	R.ELIFDDEPDK.M
8127	88 - 102	635.6204	1903.8392	1903.8662	-14.2	1	77	2e-08	1	U	R.ELIFDDEPDKMYYAR.L
8128	88 - 102	635.6230	1903.8472	1903.8662	-9.97	1	87	2.2e-09	1	U	R.ELIFDDEPDKMYYAR.L
8196	88 - 102	640.9545	1919.8418	1919.8611	-10.1	1	88	1.7e-09	1	U	R.ELIFDDEPDKMYYAR.L + Oxidation (M)
8197	88 - 102	960.9360	1919.8574	1919.8611	-1.93	1	90	1.1e-09	1	U	R.ELIFDDEPDKMYYAR.L + Oxidation (M)
2729	103 - 111	532.7783	1063.5420	1063.5549	-12.1	0	83	4.5e-09	1	U	R.LSEQFALEK.V
11323	211 - 239	1127.5593	3379.6561	3379.6394	4.92	0	89	1.4e-09	1	U	K.YIDSIEWFELEQGMNTITHSGNINQITV[illegible]
11324	211 - 239	1127.5608	3379.6606	3379.6394	6.25	0	91	8.8e-10	1	U	K.YIDSIEWFELEQGMNTITHSGNINQITV[illegible]

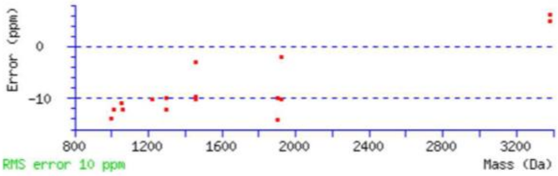

Mascot: http://www.matrixscience.com/

MASCOT Search Results

Protein View: TP84_23

**Database:**	TP84
**Score:**	2720
**Nominal mass (M_r):**	99414
**Calculated pI:**	5.06

Sequence similarity is available as **an NCBI BLAST search of 78. against nr**.

Search parameters

**Enzyme:**	Trypsin: cuts C-term side of KR unless next residue is P.
**Fixed modifications:**	**Carbamidomethyl (C)**
**Variable modifications:**	**Oxidation (M)**

Protein sequence coverage: 49%

Matched peptides shown in ***bold red***.

1 MAIQK**ALFTN QSDFIQIGSN QVGVDPFLYY NGDQYQQNKV AIQMQR**FGSP
51 AAIGPGIRGN AAAMDGGCTI WR**NVPNPAAR** KR**MISLWIKP TAADLSRFFS**
101 **VICTDR**GDGW TDHGMHLALN FDSAKQKTLL NARIIRSGSG TSQWYTHDPD
151 QGTIEFQADQ WYHICFFYDA DGGLPGGR**YV AVYVNKTLQL QSFVAPFDIT**
201 **AAYTR**SFTLG DIPNNGSYSG TYR**FSGMIDE VIRLHGDDVW TIQEMQQYYD**
251 **DIMAGNYIDA ETEPGTMK**VG K**NFVTGQYNT NLMTWTSPTI DLGQDGFDDF**
301 **GRVQLNFEQP PGTFINIYTR TSDDGQNWDA WVKTSVDGTI NSSDKR**YLQI
351 K**IEFQTTNGA ITPKIMEVQV LDYQK**IKR**LT LTSEPLIIYK DLESGLER**IG
401 ELKNAYDVII TEEINGEEVI EFKMASNDPK **RIELGAEPVE LIAR**IGDKQF
451 IIRNAIDKRD ENGKK**YTQFF GEALWYELR**D AKVINYEQVE KTAYEHIQAI
501 LNSAVVPTGW TIYKVESDGR KRTIR**GEWKS VLELLR**EVVD QFGGELQFDT
551 INR**TISLVNR** IGEDNGVRFY YNK**NLKTIER SVDTYNLITR LYLYGK**NGMT
601 VQSVHPQGLE YIEDLTWVNA LNLRNKIR**IG VWKDERYTIP QNLYDDGMK**M
651 LQEMAKPNVS YAMTIADLSM LSGHEHESIG LGDTVWVVDT ELMNLLVEAR
701 IVRRKYNVRQ PWK**TEVELNQ PKKELADANR RAIDDAIETL VESDPLDTSD**
751 **VQQMTVFNHL LNSR**AEDGTK YWEVTGSDIT IEPSGFSGNA SWKIQSGYGK
801 TNKLK**QSIYG VSHR**SAYTIS AYVATEGTIT R**GTSQDAFVG IKVTIHYTEP**
851 **DSDGK**TYEEH FLAIPDITQQ GGEDNGG

Unformatted sequence string: **877 residues** (for pasting into other applications).

Sort peptides by ● Residue Number ○ Increasing Mass ○ Decreasing Mass

Show predicted peptides also

Query	Start – End	Observed	Mr(expt)	Mr(calc)	ppm	M	Score	Expect	Rank	U	Peptide
12686	6 – 39	1304.6230	3910.8472	3910.8439	0.85	0	39	0.00012	1	U	K.ALFTNQSDFIQIGSNQVGVDPFLYYNGDQ
12687	6 – 39	1304.6311	3910.8715	3910.8439	7.06	0	83	4.9e-09	1	U	K.ALFTNQSDFIQIGSNQVGVDPFLYYNGDQ
949	40 – 46	423.2339	844.4532	844.4589	-6.68	0	52	7.1e-06	1	U	K.VAIQMQR.F
1074	40 – 46	431.2313	860.4481	860.4538	-6.68	0	44	4e-05	1	U	K.VAIQMQR.F + Oxidation (M)
1075	40 – 46	431.2317	860.4489	860.4538	-5.72	0	49	1.3e-05	1	U	K.VAIQMQR.F + Oxidation (M)
883	73 – 80	419.7280	837.4414	837.4457	-5.08	0	34	0.0004	1	U	R.NVPNPAAR.K
7486	83 – 97	573.3119	1716.9140	1716.9232	-5.39	0	50	1.1e-05	1	U	R.MISLWIKPTAADLSR.F + Oxidation (M)
3499	98 – 106	572.7752	1143.5358	1143.5383	-2.14	0	54	4.1e-06	1	U	R.FFSVICTDR.G
1828	179 – 186	478.2622	954.5097	954.5175	-8.08	0	58	1.4e-06	1	U	R.YVAVYVNK.T
9450	187 – 205	714.7111	2141.1116	2141.1157	-1.92	0	115	3.3e-12	1	U	K.TLQLQSFVAPFDITAAYTR.S
9451	187 – 205	1071.5739	2141.1332	2141.1157	8.19	0	124	4.1e-13	1	U	K.TLQLQSFVAPFDITAAYTR.S
3724	224 – 233	583.7893	1165.5640	1165.5801	-13.8	0	4	0.38	1	U	R.FSGMIDEVIR.L
3725	224 – 233	583.7935	1165.5724	1165.5801	-6.67	0	87	2.2e-09	1	U	R.FSGMIDEVIR.L
12718	234 – 268	1359.9406	4076.8000	4076.7754	6.02	0	102	7e-11	1	U	R.LHGDDVWTIQEMQQYYDDIMAGNYIDAET
12725	234 – 268	1365.2721	4092.7945	4092.7703	5.90	0	102	6.9e-11	1	U	R.LHGDDVWTIQEMQQYYDDIMAGNYIDAET + Oxidation (M)
12507	272 – 302	1170.8754	3509.6044	3509.5835	5.96	0	58	1.5e-06	1	U	K.NFVTGQYNTNLMTWTSPTIDLGQDGFDDF
9432	303 – 320	713.0394	2136.0965	2136.1004	-1.81	0	11	0.076	1	U	R.VQLNFEQPPGTFINIYTR.T
9433	303 – 320	1069.0623	2136.1100	2136.1004	4.52	0	136	2.4e-14	1	U	R.VQLNFEQPPGTFINIYTR.T
6186	321 – 333	761.3319	1520.6493	1520.6532	-2.58	0	90	9.5e-10	1	U	R.TSDDGQNWDAWVK.T
4182	334 – 345	612.2883	1222.5621	1222.5677	-4.62	0	73	5.3e-08	1	U	K.TSVDGTINSSDK.R
5324	334 – 346	460.5612	1378.6617	1378.6688	-5.15	1	79	1.1e-08	1	U	K.TSVDGTINSSDKR.Y
5325	334 – 346	690.3408	1378.6671	1378.6688	-1.24	1	89	1.1e-09	1	U	K.TSVDGTINSSDKR.Y
5326	334 – 346	690.3410	1378.6675	1378.6688	-0.94	1	113	5e-12	1	U	K.TSVDGTINSSDKR.Y
5613	352 – 364	710.3773	1418.7401	1418.7405	-0.32	0	84	3.9e-09	1	U	K.IEFQTTNGAITPK.I
5240	365 – 375	683.3549	1364.6952	1364.7010	-4.23	0	77	2e-08	1	U	K.IMEVQVLDYQK.I
5342	365 – 375	691.3532	1380.6918	1380.6959	-2.94	0	81	8.6e-09	1	U	K.IMEVQVLDYQK.I + Oxidation (M)
5410	379 – 390	695.9118	1389.8091	1389.8119	-1.97	0	100	1.1e-10	1	U	R.LTLTSEPLIIYK.D
1515	391 – 398	459.7265	917.4385	917.4454	-7.54	0	50	1e-05	1	U	K.DLESGLER.I
1516	391 – 398	459.7267	917.4389	917.4454	-7.08	0	40	8.9e-05	1	U	K.DLESGLER.I
6471	431 – 444	522.6351	1564.8835	1564.8936	-6.50	1	62	6.5e-07	1	U	K.RIELGAEPVELIAR.I
6472	431 – 444	783.4531	1564.8916	1564.8936	-1.29	1	72	6.8e-08	1	U	K.RIELGAEPVELIAR.I
5551	432 – 444	705.4019	1408.7893	1408.7925	-2.31	0	115	3.4e-12	1	U	R.IELGAEPVELIAR.I
8015	466 – 479	911.9298	1821.8451	1821.8726	-15.1	0	1	0.82	1	U	K.YTQFFGEALWYELR.D
5023	526 – 536	665.3878	1328.7611	1328.7452	12.0	1	0	0.95	1	U	R.GEWKSVLELLR.E
808	530 – 536	415.2582	828.5018	828.5069	-6.15	0	50	1.1e-05	1	U	K.SVLELLR.E
608	554 – 560	401.7408	801.4670	801.4708	-4.83	0	5	0.29	1	U	R.TISLVNR.I
1184	574 – 580	437.2589	872.5033	872.5079	-5.33	1	14	0.036	1	U	K.NLKTIER.S
3857	581 – 590	591.3076	1180.6007	1180.6088	-6.82	0	50	9.1e-06	1	U	R.SVDTYNLITR.L

314	591 – 596	378.7155	755.4164	755.4218	-7.13	0	34	0.0004	1	U	R.LYLYGK.N
2300	629 – 636	334.8488	1001.5247	1001.5294	-4.75	1	25	0.0032	1	U	R.IGVWKDER.Y
2302	629 – 636	334.8527	1001.5363	1001.5294	6.90	1	18	0.016	1	U	R.IGVWKDER.Y
6406	637 – 649	779.3653	1556.7161	1556.7181	-1.28	0	73	4.8e-08	1	U	R.YTIPQNLYDDGMK.M
6510	637 – 649	787.3626	1572.7106	1572.7130	-1.54	0	57	1.9e-06	1	U	R.YTIPQNLYDDGMK.M + Oxidation (M)
2791	714 – 722	529.2771	1056.5395	1056.5451	-5.27	0	49	1.1e-05	1	U	K.TEVELNQPK.K
1499	723 – 730	458.7437	915.4728	915.4774	-4.99	1	44	4e-05	1	U	K.KELADANR.R
1722	724 – 731	472.7526	943.4907	943.4835	7.59	1	16	0.025	1	U	K.ELADANRR.A
12609	732 – 764	922.4529	3685.7825	3685.7781	1.18	0	86	2.3e-09	1	U	R.AIDDAIETLVESDPLDTSDVQQMTVFNHI
12610	732 – 764	1229.6051	3685.7935	3685.7781	4.16	0	165	3.2e-17	1	U	R.AIDDAIETLVESDPLDTSDVQQMTVFNHI
12615	732 – 764	1234.9360	3701.7862	3701.7731	3.54	0	108	1.5e-11	1	U	R.AIDDAIETLVESDPLDTSDVQQMTVFNHI + Oxidation (M)
2709	806 – 814	523.7690	1045.5234	1045.5305	-6.75	0	64	3.8e-07	1	U	K.QSIYGVSHR.S
3329	832 – 842	561.7918	1121.5691	1121.5717	-2.29	0	65	3.3e-07	1	U	R.GTSQDAFVGIK.V
5834	843 – 855	487.8973	1460.6701	1460.6783	-5.62	0	51	7.6e-06	1	U	K.VTIHYTEPDSDGK.T
5835	843 – 855	731.3523	1460.6900	1460.6783	8.00	0	50	9.5e-06	1	U	K.VTIHYTEPDSDGK.T

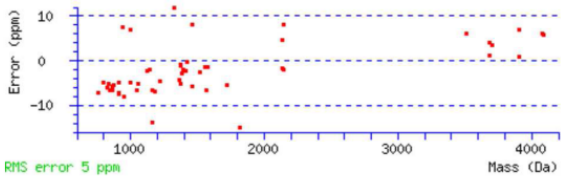

MASCOT Search Results

Protein View: TP84_25

Database: TP84
Score: 678
Nominal mass (M_r): 34431
Calculated pI: 5.35

Sequence similarity is available as **an NCBI BLAST search of 80. against nr**.

Search parameters

Enzyme: Trypsin: cuts C-term side of KR unless next residue is P.
Fixed modifications: **Carbamidomethyl (C)**
Variable modifications: **Oxidation (M)**

Protein sequence coverage: 42%

Matched peptides shown in ***bold red***.

1 MPKITDWK**VF** **GGTVELER**KH MKVDHVDLEI VVHDTVSWK**D** **GPQPIYFTDL**
51 **QFQPGHQKTG** **WIPNTQEFLD** **RVEFTVDELR** **R**YRLADGSVD PYFQFPPGVT
101 PR**TYTPEELG** **YQRLFNIMGR** **GHEVIVLPND** **LPEPEFWDLD** **LIAQK**GLERP
151 VEILSTGIDF TIIPKDDFEL MRLSNNIGAL LPEEEQKYPD DPEHPLNYRY
201 TREFWIGSGH AGDVIEINAT TMTAKVNGIT INTQGIK**QIT** **TGSDTIK**IYK
251 NKFHLMPRGS VRFR**VEFYGR** DANGR**LADTG** **IGYR**GTATFK **QWTYGVER**L

Unformatted sequence string: **299 residues** (for pasting into other applications).

Sort peptides by ● Residue Number ○ Increasing Mass ○ Decreasing Mass

Show predicted peptides also

Query	Start – End	Observed	Mr(expt)	Mr(calc)	ppm	M	Score	Expect	Rank	U	Peptide
2919	9 – 18	553.7899	1105.5651	1105.5768	-10.5	0	93	5.1e-10	1	U	K.VFGGTVELER.K
8951	40 – 58	739.3597	2215.0574	2215.0698	-5.63	0	74	3.6e-08	1	U	K.DGPQPIYFTDLQFQPGHQK.T
5958	59 – 71	788.8888	1575.7631	1575.7682	-3.20	0	66	2.4e-07	1	U	K.TGWIPNTQEFLDR.V
10419	59 – 80	889.1102	2664.3087	2664.3184	-3.64	1	43	5.5e-05	1	U	K.TGWIPNTQEFLDRVEFTVDELR.R
4077	72 – 81	421.8902	1262.6489	1262.6619	-10.3	1	48	1.5e-05	1	U	R.VEFTVDELRR.Y
4729	103 – 113	678.8198	1355.6250	1355.6357	-7.89	0	71	8.9e-08	1	U	R.TYTPEELGYQR.L
908	114 – 120	425.7286	849.4426	849.4531	-12.3	0	47	2e-05	1	U	R.LFNIMGR.G
1009	114 – 120	433.7263	865.4380	865.4480	-11.5	0	32	0.00068	1	U	R.LFNIMGR.G + Oxidation (M)
10863	121 – 145	963.1648	2886.4725	2886.4803	-2.70	0	118	1.8e-12	1	U	R.GHEVIVLPNDLPEPEFWDLDLIAQK.G
2570	238 – 247	532.2790	1062.5435	1062.5557	-11.4	0	84	4.5e-09	1	U	K.QITTGSDTIK.I
350	265 – 270	385.6899	769.3653	769.3759	-13.8	0	38	0.00016	1	U	R.VEFYGR.D
1761	276 – 284	483.2496	964.4846	964.4978	-13.7	0	68	1.7e-07	1	U	R.LADTGIGYR.G
2393	291 – 298	519.7464	1037.4783	1037.4930	-14.2	0	53	5e-06	1	U	K.QWTYGVER.L

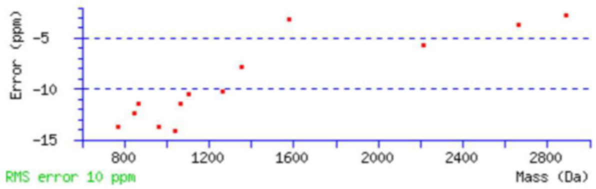

PROTEIN VIEW: **TP84_47**

SEQUENCE COVERAGE: 93.7%

Protein ID | Spectra

Proteins Detected

N	Unused	Total	% Cov	Accessio...	Name	Species	Peptides(95%)	Biological Processes	Molecular Functions	PANTHER ID
1	1.20	1.20	93.7	tr|A0A1U...	Putative membrane protein OS=Geobacillus pha...	9VIRU	0			
2	0.11	0.11	29.1	tr|A0A1U...	Uncharacterized protein OS=Geobacillus phage...	9VIRU	0			

Protein Group 1 - Putative membrane protein OS=Geobacillus phage TP-84 PE=4 SV=1

Proteins in Group

N	Unused	Total	Accessio...	Name	Species
1	1.20	1.20	tr|A0A1U...	Putative membrane...	9VIRU

Peptides in Group

Con...	Conf	Sequence	Modifications	Cleavages	ΔMass	Prec MW	z	Sc	Spectrum	Type
1.20	93.7	LDEVITRLA			0.0955	1028.68...	2	10	1.1.1.2195.2	Win...
0.00	0.1	APALMQIVIQI			-0.0970	1195.60...	2	4	1.1.1.2293.3	Win...
0.00	< 1	ADMGDIIMKACQPIIDLLQ			0.1004	2087.14...	3	5	1.1.1.2402.2	Win...
0.00	0.1	ALMQIVIQIGQ	Gln->Ser@4		-0.0339	1171.62...	2	6	1.1.1.2470.2	Win...
0.00	< 1	ALMQIVIQIGQNIQA			0.0744	1638.98...	3	5	1.1.1.2838.2	Win...
0.00	< 1	ALMQIVIQIGQNIQA			0.0744	1638.98...	3	5	1.1.1.2839.2	Win...
0.00	< 1	ALMQIVIQIGQNIQA			0.0758	1638.98...	3	4	1.1.1.2955.2	Win...
0.00	2.9	ALMQIVIQIGQNIQAQVGVV	Val->Tyr@6 Deamidated(Q)@16		0.0333	2186.21...	3	12	1.1.1.3516.3	Win...
0.00	< 1	APALMQIVIQI			-0.0970	1195.60...	2	4	1.1.1.2290.2	Win...
0.00	< 1	DAHAADMGDIIMKAC	Oxidation(M)@7		-0.1411	1576.52...	4	3	1.1.1.3229.2	Win...
0.00	< 1	DLLQGISYPVAFIMITGGF	Dethiomethyl(M)@14		0.1035	1993.15...	3	8	1.1.1.3290.3	Win...
0.00	0.1	FAPALMQIVIQI	Gln->Ser@11		0.0843	1301.82...	2	9	1.1.1.3190.3	Win...
0.00	0.1	FAPALMQIVIQI	Gln->Ser@11		0.0843	1301.82...	2	9	1.1.1.3194.2	Win...
0.00	0.1	FAPALMQIVIQI	Gln->Ser@11		0.0843	1301.82...	2	9	1.1.1.3196.2	Win...
0.00	0.1	FAPALMQIVIQI	Gln->Ser@11		0.0843	1301.82...	2	9	1.1.1.3197.2	Win...
0.00	0.1	FAPALMQIVIQI	Gln->Ser@11		0.0843	1301.82...	2	9	1.1.1.3198.2	Win...
0.00	0.1	FAPALMQIVIQI	Gln->Ser@11		0.0843	1301.82...	2	9	1.1.1.3200.2	Win...

Protein Sequence Coverage - Putative membrane protein OS=Geobacillus phage TP-84 PE=4 SV=1

MDGNWREKKAERRKESLDEVITRLAAVGTGAHMVIHPIDAHAADMGDIIMKACQPIIDLLQGISYPVAFIMITGGFLLIMTGQTSRGMHFIKWACLGYLGLQFAPALMQIVIQIGQNIQAQVGVVR

MASCOT Search Results

Protein View: TP84_74

**Database:**	TP84
**Score:**	456
**Nominal mass (M_r):**	21341
**Calculated pI:**	9.19

Sequence similarity is available as **an NCBI BLAST search of 46. against nr**.

Search parameters

**Enzyme:**	Trypsin: cuts C-term side of KR unless next residue is P.
**Fixed modifications:**	**Carbamidomethyl (C)**
**Variable modifications:**	**Oxidation (M)**

Protein sequence coverage: 34%

Matched peptides shown in ***bold red***.

```
  1 MTKNIDSNKQ AATTKPPQGK IYDQRWQDYQ RRIGFYKKTV AKYNAIVKRI
 51 EELYKITEQL DDNNPDDVLK KAEIYGMILE DVGDLHYISI TAWKYADALK
101 KEAYALAIIR ERPNGRTVEA HREMAVLESQ EWRWKMAEWE GLTKRWENAK
151 TTIEEQIKIM KWKIKWTLAN MQQAGMANPN A
```

Unformatted sequence string: **181 residues** (for pasting into other applications).

Sort peptides by ◉ Residue Number ○ Increasing Mass ○ Decreasing Mass

Show predicted peptides also

Query	Start – End	Observed	Mr(expt)	Mr(calc)	ppm	M	Score	Expect	Rank	U	Peptide
3211	10 – 20	563.8108	1125.6070	1125.6142	-6.36	0	76	2.5e-08	1	U	K.QAATTKPPQGK.I
1699	49 – 55	475.7665	949.5185	949.5232	-5.00	1	35	0.00029	1	U	K.RIEELYK.I
539	50 – 55	397.7161	793.4176	793.4221	-5.68	0	48	1.7e-05	1	U	R.IEELYK.I
6670	56 – 70	864.9194	1727.8242	1727.8214	1.62	0	101	8.8e-11	1	U	K.ITEQLDDNNPDDVLK.K
7225	56 – 71	619.6447	1855.9123	1855.9163	-2.15	1	99	1.3e-10	1	U	K.ITEQLDDNNPDDVLKK.A
2297	102 – 110	510.2961	1018.5776	1018.5811	-3.45	0	66	2.4e-07	1	U	K.EAYALAIIR.E
5051	123 – 133	697.3269	1392.6393	1392.6343	3.57	0	61	7.9e-07	1	U	R.EMAVLESQEWR.W + Oxidation (M)
2664	136 – 144	532.7556	1063.4967	1063.5008	-3.86	0	62	7e-07	1	U	K.MAEWEGLTK.R

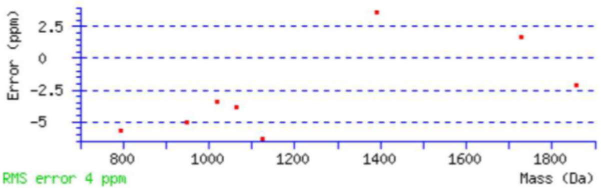

Mascot: **http://www.matrixscience.com/**
