# Supplementary material for: Sequence, genome organization, annotation and proteomics of the thermophilic, 47.7-kb Geobacillus stearothermophilus bacteriophage TP-84 and its classification in the new Tp84virus genus
Source: PLoS One. 2018 Apr 6;13(4):e0195449. doi: 10.1371/journal.pone.0195449 (PMC5889276; doi:10.1371/journal.pone.0195449)
Supplement: S6 File — (PDF) [file pone.0195449.s006.pdf]

MASCOT Search Results

Protein View: TP84_03

Database: TP84
Score: 261
Nominal mass (M_r): 26292
Calculated pI: 9.17

Sequence similarity is available as **an NCBI BLAST search of 58. against nr**.

Search parameters

MS data file: O:\FA\02-luty2018\80202245zeb_czas25.raw
Enzyme: Trypsin: cuts C-term side of KR unless next residue is P.
Fixed modifications: **Carbamidomethyl (C)**
Variable modifications: **Oxidation (M)**

Protein sequence coverage: 22%

Matched peptides shown in ***bold red***.

```
  1 LVKTEQKQMS SVQNIRCFTI KMSADRIGQH PKIPNNHHFI HSKGESFMGL
 51 KMWIKKKLGL VYPSDVLRDK PGGFIDTTAF GTMPVNQIKP KQGGSNMADT
101 YAEAMINKTN YPDADPEMTF EVQRSTLDKL AEEYKKTMDK VDKNENVHDV
151 HQDVINKPRH YHQGGFDALY VIERKFGRVV LRGFYIGNII KYILRFEQKN
201 GVEDLKKARF YLDKLIELEE GSAPDQRG
```

Unformatted sequence string: **228 residues** (for pasting into other applications).

Sort peptides by ◉ Residue Number ○ Increasing Mass ○ Decreasing Mass

Show predicted peptides also

Query	Start - End	Observed	Mr(expt)	Mr(calc)	ppm	M	Score	Expect	Rank	U	Peptide
33512	69 - 91	821.4311	2461.2714	2461.2675	1.56	0	92	6.3e-10	1	U	R.DKPGGFIDTTAFGTMPVNQIKPK.Q
26656	109 - 124	956.9213	1911.8281	1911.8309	-1.43	0	141	8.6e-15	1	U	K.TNYPDADPEMTFEVQR.S
16446	215 - 227	728.8669	1455.7192	1455.7205	-0.90	0	54	4.1e-06	1	U	K.LIELEEGSAPDQR.G

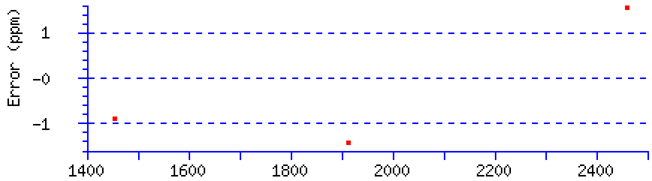

RMS error 1 ppm

Mascot: http://www.matrixscience.com/

MASCOT Search Results

Protein View: TP84_03

Database: TP84
Score: 743
Nominal mass (M_r): 26292
Calculated pI: 9.17

Sequence similarity is available as **an NCBI BLAST search of 58. against nr**.

Search parameters

MS data file: O:\FA\02-luty2018\80202246zeb_czas30.raw
Enzyme: Trypsin: cuts C-term side of KR unless next residue is P.
Fixed modifications: **Carbamidomethyl (C)**
Variable modifications: **Oxidation (M)**

Protein sequence coverage: 28%

Matched peptides shown in ***bold red***.

```
  1 LVKTEQKQMS SVQNIRCFTI KMSADRIGQH PKIPNNHHFI HSKGESFMGL
 51 KMWIKKKLGL VYPSDVLRDK PGGFIDTTAF GTMPVNQIKP KQGGSNMADT
101 YAEAMINKTN YPDADPEMTF EVQRSTLDKL AEEYKKTMDK VDKNENVHDV
151 HQDVINKPRH YHQGGFDALY VIERKFGRVV LRGFYIGNII KYILRFEQKN
201 GVEDLKKARF YLDKLIELEE GSAPDQRG
```

Unformatted sequence string: **228 residues** (for pasting into other applications).

Sort peptides by ◉ Residue Number ○ Increasing Mass ○ Decreasing Mass

Show predicted peptides also

Query	Start – End	Observed	Mr(expt)	Mr(calc)	ppm	M	Score	Expect	Rank	U	Peptide
14652	58 – 68	616.3560	1230.6975	1230.6972	0.23	0	57	2e-06	1	U	K.LGLVYPSDVLR.D
14653	58 – 68	616.3561	1230.6977	1230.6972	0.36	0	56	2.3e-06	1	U	K.LGLVYPSDVLR.D
14654	58 – 68	616.3565	1230.6984	1230.6972	0.98	0	54	4e-06	1	U	K.LGLVYPSDVLR.D
14655	58 – 68	616.3569	1230.6992	1230.6972	1.60	0	62	6.3e-07	1	U	K.LGLVYPSDVLR.D
43216	69 – 91	616.3251	2461.2712	2461.2675	1.49	0	71	8.2e-08	1	U	R.DKPGGFIDTTAFGTMPVNQIKPK.Q
43217	69 – 91	616.3254	2461.2725	2461.2675	1.99	0	51	8.3e-06	1	U	R.DKPGGFIDTTAFGTMPVNQIKPK.Q
41117	141 – 159	752.7197	2255.1374	2255.1407	-1.45	1	102	6.2e-11	1	U	K.VDKNENVHDVHQDVINKPR.H
41119	141 – 159	564.7925	2255.1409	2255.1407	0.075	1	71	7.2e-08	1	U	K.VDKNENVHDVHQDVINKPR.H
35694	144 – 159	638.6574	1912.9503	1912.9504	-0.011	0	77	2e-08	1	U	K.NENVHDVHQDVINKPR.H
23071	215 – 227	728.8672	1455.7198	1455.7205	-0.47	0	90	9.6e-10	1	U	K.LIELEEGSAPDQR.G
23072	215 – 227	728.8673	1455.7201	1455.7205	-0.25	0	90	9.5e-10	1	U	K.LIELEEGSAPDQR.G
23073	215 – 227	728.8675	1455.7204	1455.7205	-0.080	0	104	3.9e-11	1	U	K.LIELEEGSAPDQR.G

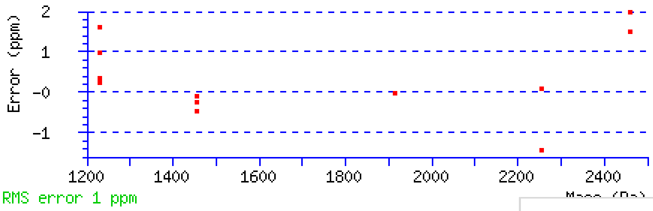

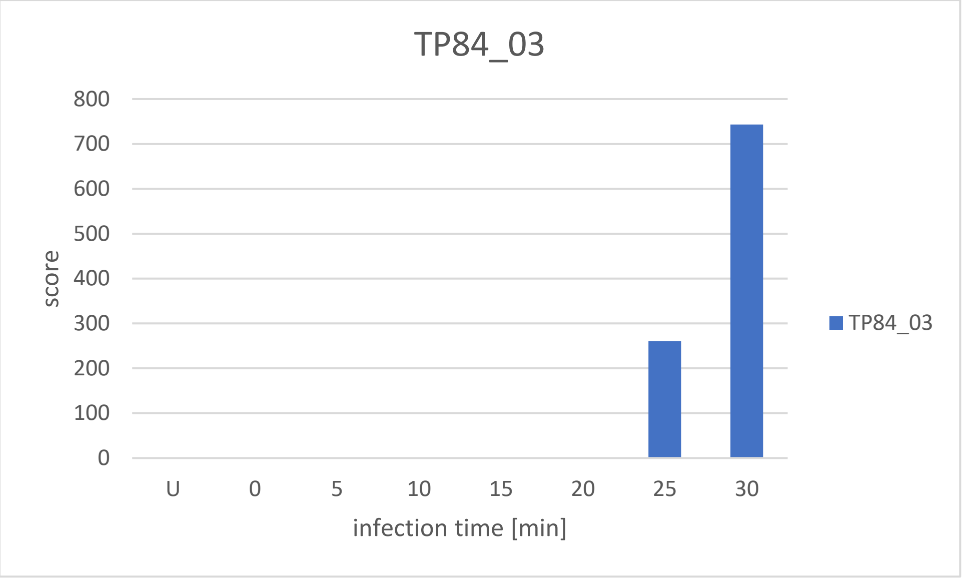

MASCOT Search Results

Protein View: TP84_06

**Database:**	TP84
**Score:**	194
**Nominal mass (M_r):**	62259
**Calculated pI:**	4.96

Sequence similarity is available as **an NCBI BLAST search of 61. against nr**.

Search parameters

**MS data file:**	O:\FA\02-luty2018\80202240zeb_czas0.raw
**Enzyme:**	Trypsin: cuts C-term side of KR unless next residue is P.
**Fixed modifications:**	**Carbamidomethyl (C)**
**Variable modifications:**	**Oxidation (M)**

Protein sequence coverage: 5%

Matched peptides shown in ***bold red***.

```
  1 MGIKWTKWST NVIKKYHGNI QKYRKLYDGD HAKLFERAKR LIQEGEITDQ
 51 IIEGAEVARN VKTPYIVANV CKMIVDIPAM LVSRAIGQVT TSMSPDDFAG
101 MVTDQGDGTV ISLYEKQKEL IKGIAKRSNL QFEHKTNIIH HQMDGGIVGM
151 PFDDENGLRI EFKSRDVYYP HPDGRGCDLV YQLEIEDEET EEAIKYLHVY
201 RERVEEQKLV TQHMLYKIGE SGMLEEIEDE AEVKEILGIE KTYREFEGRD
251 KPFVVYWPNN KTFTHPLGRS ELYNLAGKQD EINWTLTRNA IVYERNGKPR
301 IAVSKEIFQA LQDKAFERYG DENKIDHRDL EIVTFDENGK AMEVIQIDVS
351 KIGDIKWVKD LMKLMLMETH TSEKAVDFYL EGNTSAQSGI AKFYDLFVSI
401 MKAEQIATEY VHFLQELFEN CLWIAHQDDP DIVIEEPRIQ IKDMIPISRR
451 ELIEQESTAY KNGTQSLETT VRNQNPTATE DWIEDELAAI EESQQSTDTT
501 SILMGRQTLS NLLDNRNPNG TPIGAAQQQP QQGTPQTGGG QA
```

Unformatted sequence string: **542 residues** (for pasting into other applications).

Sort peptides by (•) Residue Number ( ) Increasing Mass ( ) Decreasing Mass

Show predicted peptides also

Query	Start – End	Observed	Mr(expt)	Mr(calc)	ppm	M	Score	Expect	Rank	U	Peptide
26286	41 – 59	1042.5502	2083.0858	2083.0797	2.96	0	139	1.2e-14	1	U	R.LIQEGEITDQIIEGAEVAR.N
10714	451 – 461	655.8302	1309.6459	1309.6401	4.42	0	67	1.8e-07	1	U	R.ELIEQESTAYK.N

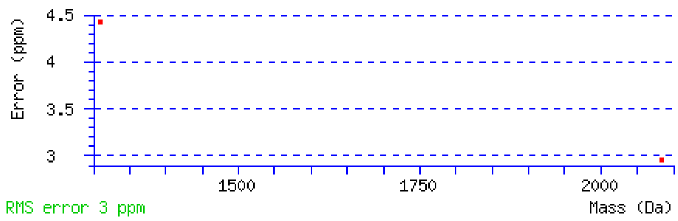

MASCOT Search Results

Protein View: TP84_06

Database: TP84
Score: 203
Nominal mass (M_r): 62259
Calculated pI: 4.96

Sequence similarity is available as an NCBI BLAST search of 61. against nr.

Search parameters

MS data file: O:\FA\02-luty2018\80202241zeb_czas5.raw
Enzyme: Trypsin: cuts C-term side of KR unless next residue is P.
Fixed modifications: Carbamidomethyl (C)
Variable modifications: Oxidation (M)

Protein sequence coverage: 9%

Matched peptides shown in ***bold red***.

1 MGIKWTKWST NVIKKYHGNI QKYRKLYDGD HAKLFERAKR **LIQEGEITDQ**
51 **IIEGAEVAR**N VKTPYIVANV CK**MIVDIPAM LVSR**AIGQVT TSMSPDDFAG
101 MVTDQGDGTV ISLYEKQKEL IKGIAKRSNL QFEHKTNIIH HQMDGGIVGM
151 PFDDENGLRI EFKSRDVYYP HPDGRGCDLV YQLEIEDEET EEAIKYLHVY
201 RERVEEQKLV TQHMLYKIGE SGMLEEIEDE AEVKEILGIE KTYREFEGRD
251 KPFVVYWPNN KTFTHPLGRS ELYNLAGKQD EINWTLTRNA IVYERNGKPR
301 IAVSKEIFQA LQDKAFERYG DENKIDHRDL EIVTFDENGK AMEVIQIDVS
351 KIGDIKWVKD LMKLMLMETH TSEKAVDFYL EGNTSAQSGI AKFYDLFVSI
401 MKAEQIATEY VHFLQELFEN CLWIAHQDDP DIVIEEPRIQ IKDMIPISRR
451 ELIEQESTAY K**NGTQSLETT VR**NQNPTATE DWIEDELAAI EESQQSTDTT
501 SILMGR**QTLS NLLDNR**NPNG TPIGAAQQQP QQGTPQTGGG QA

Unformatted sequence string: 542 residues (for pasting into other applications).

Sort peptides by ◉ Residue Number ○ Increasing Mass ○ Decreasing Mass

Show predicted peptides also

Query	Start – End	Observed	Mr(expt)	Mr(calc)	ppm	M	Score	Expect	Rank	U	Peptide
26156	41 – 59	1042.5348	2083.0550	2083.0797	-11.8	0	112	6.3e-12	1	U	R.LIQEGEITDQIIEGAEVAR.N
11659	73 – 84	672.8733	1343.7321	1343.7305	1.23	0	84	4.2e-09	1	U	K.MIVDIPAMLVSR.A
8266	462 – 472	603.3020	1204.5894	1204.6048	-12.8	0	14	0.037	1	U	K.NGTQSLETTVR.N
7528	507 – 516	587.3221	1172.6297	1172.6149	12.6	0	32	0.0007	1	U	R.QTLSNLLDNR.N

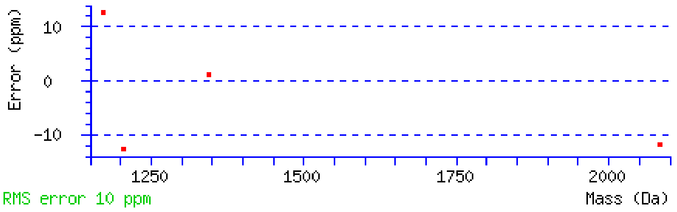

Mascot: http://www.matrixscience.com/

MASCOT Search Results

Protein View: TP84_06

Database: TP84
Score: 148
Nominal mass (M_r): 62259
Calculated pI: 4.96

Sequence similarity is available as **an NCBI BLAST search of 61. against nr**.

Search parameters

MS data file: O:\FA\02-luty2018\80202243zeb_czas15.raw
Enzyme: Trypsin: cuts C-term side of KR unless next residue is P.
Fixed modifications: **Carbamidomethyl (C)**
Variable modifications: **Oxidation (M)**

Protein sequence coverage: 9%

Matched peptides shown in ***bold red***.

```
  1 MGIKWTKWST NVIKKYHGNI QKYRKLYDGD HAKLFERAKR LIQEGEITDQ
 51 IIEGAEVARN VKTPYIVANV CKMIVDIPAM LVSRAIGQVT TSMSPDDFAG
101 MVTDQGDGTV ISLYEKQKEL IKGIAKRSNL QFEHKTNIIH HQMDGGIVGM
151 PFDDENGLRI EFKSRDVYYP HPDGRGCDLV YQLEIEDEET EEAIKYLHVY
201 RERVEEQKLV TQHMLYKIGE SGMLEEIEDE AEVKEILGIE KTYREFEGRD
251 KPFVVYWPNN KTFTHPLGRS ELYNLAGKQD EINWTLTRNA IVYERNGKPR
301 IAVSKEIFQA LQDKAFERYG DENKIDHRDL EIVTFDENGK AMEVIQIDVS
351 KIGDIKWVKD LMKLMLMETH TSEKAVDFYL EGNTSAQSGI AKFYDLFVSI
401 MKAEQIATEY VHFLQELFEN CLWIAHQDDP DIVIEEPRIQ IKDMIPISRR
451 ELIEQESTAY KNGTQSLETT VRNQNPTATE DWIEDELAAI EESQQSTDTT
501 SILMGRQTLS NLLDNRNPNG TPIGAAQQQP QQGTPQTGGG QA
```

Unformatted sequence string: **542 residues** (for pasting into other applications).

Sort peptides by ◉ Residue Number ○ Increasing Mass ○ Decreasing Mass

Show predicted peptides also

Query	Start – End	Observed	Mr(expt)	Mr(calc)	ppm	M	Score	Expect	Rank	U	Peptide
24737	41 – 59	695.3661	2083.0766	2083.0797	-1.50	0	82	6.8e-09	1	U	R.LIQEGEITDQIIEGAEVAR.N
10426	451 – 461	655.8313	1309.6481	1309.6401	6.11	0	64	3.8e-07	1	U	R.ELIEQESTAYK.N
7951	462 – 472	603.3011	1204.5876	1204.6048	-14.3	0	15	0.035	1	U	K.NGTQSLETTVR.N
7952	462 – 472	603.3012	1204.5878	1204.6048	-14.1	0	13	0.049	1	U	K.NGTQSLETTVR.N
7954	462 – 472	603.3028	1204.5910	1204.6048	-11.4	0	15	0.034	1	U	K.NGTQSLETTVR.N
7286	507 – 516	587.3177	1172.6208	1172.6149	5.03	0	25	0.0032	1	U	R.QTLSNLLDNR.N

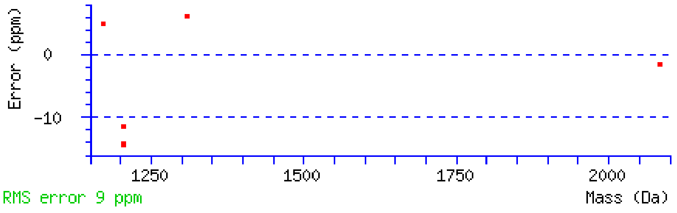

Mascot: http://www.matrixscience.com/

MASCOT Search Results

Protein View: TP84_06

**Database:**	TP84
**Score:**	393
**Nominal mass (M_r):**	62259
**Calculated pI:**	4.96

Sequence similarity is available as an NCBI BLAST search of 61. against nr.

Search parameters

**MS data file:**	O:\FA\02-luty2018\80202245zeb_czas25.raw
**Enzyme:**	Trypsin: cuts C-term side of KR unless next residue is P.
**Fixed modifications:**	Carbamidomethyl (C)
**Variable modifications:**	Oxidation (M)

Protein sequence coverage: 9%

Matched peptides shown in ***bold red***.

```
  1 MGIKWTKWST NVIKKYHGNI QKYRKLYDGD HAKLFERAKR LIQEGEITDQ
 51 IIEGAEVARN VKTPYIVANV CKMIVDIPAM LVSRAIGQVT TSMSPDDFAG
101 MVTDQGDGTV ISLYEKQKEL IKGIAKRSNL QFEHKTNIIH HQMDGGIVGM
151 PFDDENGLRI EFKSRDVYYP HPDGRGCDLV YQLEIEDEET EEAIKYLHVY
201 RERVEEQKLV TQHMLYKIGE SGMLEEIEDE AEVKEILGIE KTYREFEGRD
251 KPFVVYWPNN KTFTHPLGRS ELYNLAGKQD EINWTLTRNA IVYERNGKPR
301 IAVSKEIFQA LQDKAFERYG DENKIDHRDL EIVTFDENGK AMEVIQIDVS
351 KIGDIKWVKD LMKLMLMETH TSEKAVDFYL EGNTSAQSGI AKFYDLFVSI
401 MKAEQIATEY VHFLQELFEN CLWIAHQDDP DIVIEEPRIQ IKDMIPISRR
451 ELIEQESTAY KNGTQSLETT VRNQNPTATE DWIEDELAAI EESQQSTDTT
501 SILMGRQTLS NLLDNRNPNG TPIGAAQQQP QQGTPQTGGG QA
```

Unformatted sequence string: 542 residues (for pasting into other applications).

Sort peptides by ◉ Residue Number ○ Increasing Mass ○ Decreasing Mass

Show predicted peptides also

Query	Start – End	Observed	Mr(expt)	Mr(calc)	ppm	M	Score	Expect	Rank	U	Peptide
29292	41 – 59	1042.5436	2083.0726	2083.0797	-3.38	0	164	4.3e-17	1	U	R.LIQEGEITDQIIEGAEVAR.N
29295	41 – 59	695.3705	2083.0896	2083.0797	4.75	0	51	8.1e-06	1	U	R.LIQEGEITDQIIEGAEVAR.N
13606	73 – 84	672.8727	1343.7309	1343.7305	0.33	0	93	4.6e-10	1	U	K.MIVDIPAMLVSR.A
12923	364 – 374	440.5489	1318.6248	1318.6261	-1.01	0	59	1.2e-06	1	U	K.LMLMETHTSEK.A
12675	451 – 461	655.8292	1309.6439	1309.6401	2.88	0	78	1.4e-08	1	U	R.ELIEQESTAYK.N

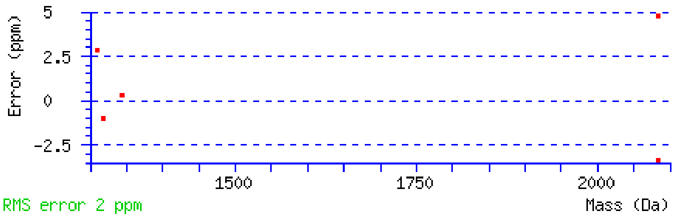

Mascot: http://www.matrixscience.com/

MASCOT Search Results

Protein View: TP84_06

Database: TP84
Score: 829
Nominal mass (M_r): 62259
Calculated pI: 4.96

Sequence similarity is available as **an NCBI BLAST search of 61. against nr**.

Search parameters

MS data file: O:\FA\02-luty2018\80202246zeb_czas30.raw
Enzyme: Trypsin: cuts C-term side of KR unless next residue is P.
Fixed modifications: **Carbamidomethyl (C)**
Variable modifications: **Oxidation (M)**

Protein sequence coverage: 11%

Matched peptides shown in ***bold red***.

1 MGIKWTKWST NVIKKYHGNI QKYRKLYDGD HAKLFERAKR **LIQEGEITDQ**
51 **IIEGAEVAR**N VKTPYIVANV CK**MIVDIPAM** **LVSR**AIGQVT TSMSPDDFAG
101 MVTDQGDGTV ISLYEKQKEL IKGIAKRSNL QFEHKTNIIH HQMDGGIVGM
151 PFDDENGLRI EFKSRDVYYP HPDGRGCDLV YQLEIEDEET EEAIKYLHVY
201 RERVEEQKLV TQHMLYKIGE SGMLEEIEDE AEVKEILGIE KTYREFEGR**D**
251 **KPFVVYWPNN** **K**TFTHPLGRS ELYNLAGK**QD** **EINWTLTR**NA IVYERNGKPR
301 IAVSKEIFQA LQDKAFERYG DENKIDHRDL EIVTFDENGK AMEVIQIDVS
351 KIGDIKWVKD LMKLMLMETH TSEKAVDFYL EGNTSAQSGI AKFYDLFVSI
401 MKAEQIATEY VHFLQELFEN CLWIAHQDDP DIVIEEPRIQ IKDMIPISRR
451 **ELIEQESTAY** **K**NGTQSLETT VRNQNPTATE DWIEDELAAI EESQQSTDTT
501 SILMGRQTLS NLLDNRNPNG TPIGAAQQQP QQGTPQTGGG QA

Unformatted sequence string: **542 residues** (for pasting into other applications).

Sort peptides by ◉ Residue Number ○ Increasing Mass ○ Decreasing Mass

Show predicted peptides also

Query	Start – End	Observed	Mr(expt)	Mr(calc)	ppm	M	Score	Expect	Rank	U	Peptide
38519	41 – 59	1042.5436	2083.0726	2083.0797	-3.38	0	136	2.4e-14	1	U	R.LIQEGEITDQIIEGAEVAR.N
38520	41 – 59	1042.5476	2083.0806	2083.0797	0.46	0	148	1.6e-15	1	U	R.LIQEGEITDQIIEGAEVAR.N
38521	41 – 59	695.3682	2083.0827	2083.0797	1.47	0	68	1.7e-07	1	U	R.LIQEGEITDQIIEGAEVAR.N
38522	41 – 59	695.3686	2083.0839	2083.0797	2.02	0	67	2.1e-07	1	U	R.LIQEGEITDQIIEGAEVAR.N
19164	73 – 84	672.8729	1343.7312	1343.7305	0.56	0	76	2.7e-08	1	U	K.MIVDIPAMLVSR.A
19165	73 – 84	672.8729	1343.7313	1343.7305	0.59	0	89	1.3e-09	1	U	K.MIVDIPAMLVSR.A
19166	73 – 84	672.8732	1343.7319	1343.7305	1.08	0	87	2e-09	1	U	K.MIVDIPAMLVSR.A
24704	250 – 261	753.8862	1505.7579	1505.7667	-5.83	0	70	8.9e-08	1	U	R.DKPFVVYWPNNK.T
24705	250 – 261	753.8913	1505.7681	1505.7667	0.94	0	78	1.4e-08	1	U	R.DKPFVVYWPNNK.T
16512	279 – 288	638.3181	1274.6216	1274.6255	-3.07	0	80	9.2e-09	1	U	K.QDEINWTLTR.N
17805	451 – 461	655.8290	1309.6435	1309.6401	2.60	0	59	1.2e-06	1	U	R.ELIEQESTAYK.N

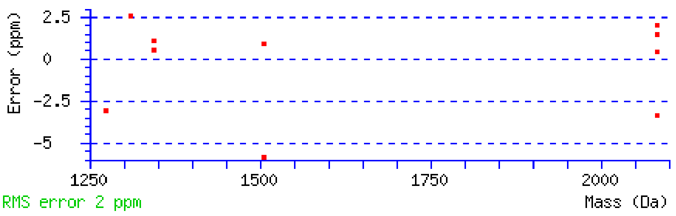

Mascot: http://www.matrixscience.com/

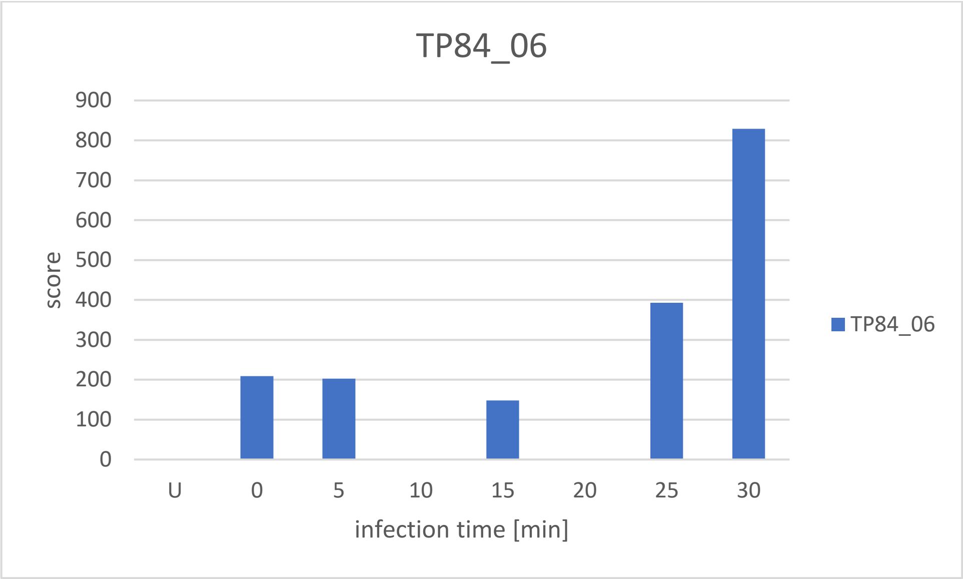
TP84_06
score
900
800
700
600
500
400
300
200
100
0
U
0
5
10
15
20
25
30
infection time [min]
TP84_06

MASCOT Search Results

Protein View: TP84_10

**Database:**	TP84
**Score:**	387
**Nominal mass (M_r):**	31601
**Calculated pI:**	4.70

Sequence similarity is available as **an NCBI BLAST search of 65. against nr**.

Search parameters

**MS data file:**	O:\FA\02-luty2018\80202245zeb_czas25.raw
**Enzyme:**	Trypsin: cuts C-term side of KR unless next residue is P.
**Fixed modifications:**	**Carbamidomethyl (C)**
**Variable modifications:**	**Oxidation (M)**

Protein sequence coverage: 15%

Matched peptides shown in ***bold red***.

```
  1 MQDKQITDEF IQKANKASEF GFLQFFAGEG GQQGGEPTDP TDPTDTDPLDDP
 51 EGGEPNDPTD PTDPVDDQDD NDDDTGITYE KGLIDRILKA NNIDFGQLLK
101 DNPELKRQYQ ARFNKNMSKR LEKYQGVDVD EYFELKKRAE SGNLEGDAKT
151 WKEKYEQLKA EMETTTKKTA IQQYAIENGF DSEQIAFITS MIDMNKLERD
201 DEGEWMGIDE EVERIKEKFP RMFEPRDGQV GGTPKKESKY NPGTKKHNQD
251 TKPTDPRELG RQIALQRHKN RLKQQ
```

Unformatted sequence string: **275 residues** (for pasting into other applications).

Sort peptides by ◉ Residue Number ○ Increasing Mass ○ Decreasing Mass

Show predicted peptides also

Query	Start – End	Observed	Mr(expt)	Mr(calc)	ppm	M	Score	Expect	Rank	U	Peptide
10489	90 – 100	616.8359	1231.6572	1231.6561	0.96	0	53	4.9e-06	1	U	K.ANNIDFGQLLK.D
10490	90 – 100	616.8361	1231.6576	1231.6561	1.27	0	57	2e-06	1	U	K.ANNIDFGQLLK.D
10491	90 – 100	616.8366	1231.6586	1231.6561	2.08	0	59	1.4e-06	1	U	K.ANNIDFGQLLK.D
6847	139 – 149	545.7547	1089.4948	1089.4938	0.90	0	99	1.2e-10	1	U	R.AESGNLEGDAK.T
6848	139 – 149	545.7554	1089.4963	1089.4938	2.30	0	93	4.9e-10	1	U	R.AESGNLEGDAK.T
2841	160 – 167	455.7133	909.4120	909.4113	0.74	0	52	6.1e-06	1	U	K.AEMETTTK.K
15923	246 – 257	479.5794	1435.7164	1435.7168	-0.24	1	51	7.1e-06	1	U	K.KHNQDTKPTDPR.E

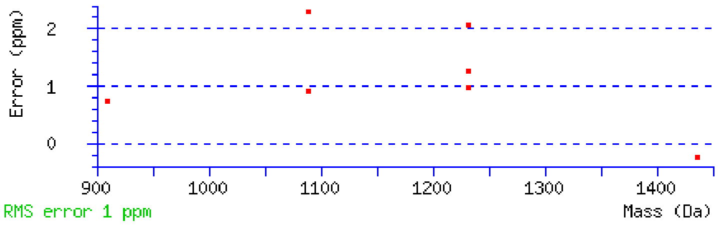

Mascot: http://www.matrixscience.com/

MASCOT Search Results

Protein View: TP84_10

**Database:**	TP84
**Score:**	1083
**Nominal mass (M_r):**	31601
**Calculated pI:**	4.70

Sequence similarity is available as **an NCBI BLAST search of 65. against nr**.

Search parameters

**MS data file:**	O:\FA\02-luty2018\80202246zeb_czas30.raw
**Enzyme:**	Trypsin: cuts C-term side of KR unless next residue is P.
**Fixed modifications:**	**Carbamidomethyl (C)**
**Variable modifications:**	**Oxidation (M)**

Protein sequence coverage: 17%

Matched peptides shown in ***bold red***.

```
  1 MQDKQITDEF IQKANKASEF GFLQFFAGEG GQQGGEPTDP TDPTDPLDDP
 51 EGGEPNDPTD PTDPVDDQDD NDDDTGITYE KGLIDRILKA NNIDFGQLLK
101 DNPELKRQYQ ARFNKNMSKR LEKYQGVDVD EYFELKKRAE SGNLEGDAKT
151 WKEKYEQLKA EMETTTKKTA IQQYAIENGF DSEQIAFITS MIDMNKLERD
201 DEGEWMGIDE EVERIKEKFP RMFEPRDGQV GGTPKKESKY NPGTKKHNQD
251 TKPTDPRELG RQIALQRHKN RLKQQ
```

Unformatted sequence string: **275 residues** (for pasting into other applications).

Sort peptides by ◉ Residue Number ○ Increasing Mass ○ Decreasing Mass

Show predicted peptides also

Query	Start – End	Observed	Mr(expt)	Mr(calc)	ppm	M	Score	Expect	Rank	U	Peptide
14691	90 – 100	616.8344	1231.6543	1231.6561	-1.46	0	52	6e-06	1	U	K.ANNIDFGQLLK.D
14692	90 – 100	616.8350	1231.6555	1231.6561	-0.45	0	61	7.3e-07	1	U	K.ANNIDFGQLLK.D
14693	90 – 100	616.8354	1231.6563	1231.6561	0.16	0	64	3.6e-07	1	U	K.ANNIDFGQLLK.D
14694	90 – 100	616.8354	1231.6563	1231.6561	0.20	0	61	8.2e-07	1	U	K.ANNIDFGQLLK.D
14695	90 – 100	616.8355	1231.6565	1231.6561	0.33	0	59	1.3e-06	1	U	K.ANNIDFGQLLK.D
14696	90 – 100	616.8358	1231.6571	1231.6561	0.85	0	54	4.2e-06	1	U	K.ANNIDFGQLLK.D
14697	90 – 100	616.8358	1231.6571	1231.6561	0.85	0	64	3.6e-07	1	U	K.ANNIDFGQLLK.D
14698	90 – 100	616.8358	1231.6571	1231.6561	0.86	0	58	1.5e-06	1	U	K.ANNIDFGQLLK.D
14699	90 – 100	616.8359	1231.6573	1231.6561	1.01	0	58	1.7e-06	1	U	K.ANNIDFGQLLK.D
14700	90 – 100	616.8360	1231.6574	1231.6561	1.11	0	62	6.3e-07	1	U	K.ANNIDFGQLLK.D
27607	124 – 136	802.8767	1603.7388	1603.7406	-1.11	0	83	4.5e-09	1	U	K.YQGVDVDEYFELK.K
27608	124 – 136	802.8770	1603.7394	1603.7406	-0.73	0	90	9.7e-10	1	U	K.YQGVDVDEYFELK.K
27609	124 – 136	802.8777	1603.7408	1603.7406	0.10	0	82	6.7e-09	1	U	K.YQGVDVDEYFELK.K
27611	124 – 136	802.8787	1603.7429	1603.7406	1.45	0	76	2.3e-08	1	U	K.YQGVDVDEYFELK.K
15263	138 – 149	416.2055	1245.5946	1245.5949	-0.26	1	53	4.5e-06	1	U	K.RAESGNLEGDAK.T
15264	138 – 149	416.2056	1245.5950	1245.5949	0.098	1	59	1.1e-06	1	U	K.RAESGNLEGDAK.T
15265	138 – 149	623.8051	1245.5956	1245.5949	0.56	1	59	1.4e-06	1	U	K.RAESGNLEGDAK.T
9432	139 – 149	545.7553	1089.4961	1089.4938	2.10	0	87	1.9e-09	1	U	R.AESGNLEGDAK.T
9433	139 – 149	545.7561	1089.4976	1089.4938	3.47	0	94	4.1e-10	1	U	R.AESGNLEGDAK.T
22369	246 – 257	479.5802	1435.7187	1435.7168	1.37	1	52	6.5e-06	1	U	K.KHNQDTKPTDPR.E

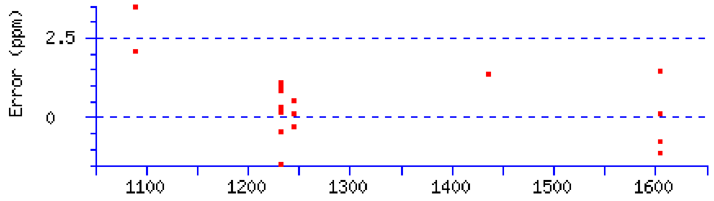

Mascot: **http://www.matrixscience.com/**

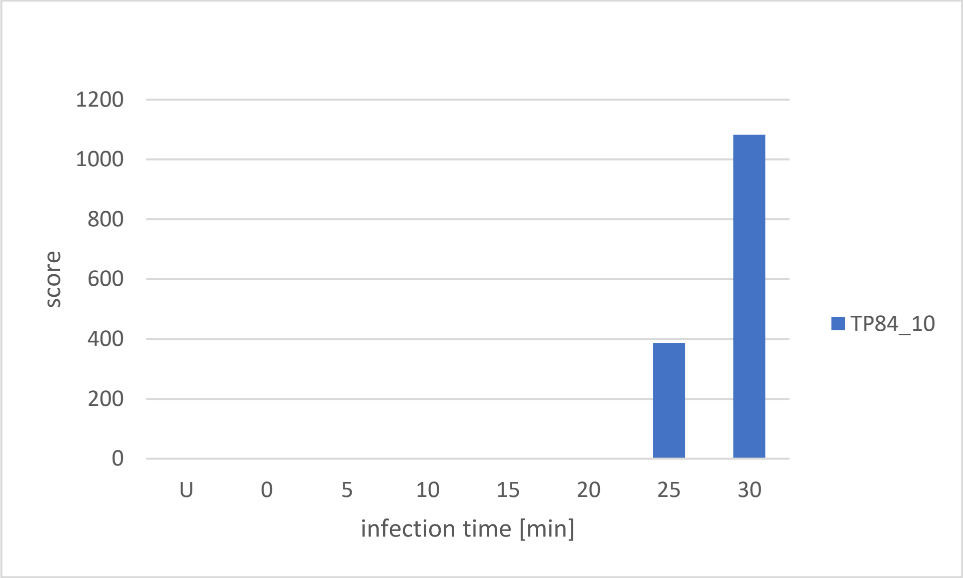

9.02.2018

MASCOT Search Results

Protein View: TP84_11

**Database:**	TP84
**Score:**	341
**Nominal mass (M_r):**	14016
**Calculated pI:**	5.84

Sequence similarity is available as **an NCBI BLAST search of 66. against nr**.

Search parameters

**MS data file:**	O:\FA\02-luty2018\80202241zeb_czas5.raw
**Enzyme:**	Trypsin: cuts C-term side of KR unless next residue is P.
**Fixed modifications:**	**Carbamidomethyl (C)**
**Variable modifications:**	**Oxidation (M)**

Protein sequence coverage: 24%

Matched peptides shown in ***bold red***.

```
  1 MNLQPKIVSI AGQKEFLATT QGLVHKVGGV TLDASKFTPD ENGFIKAGSA
 51 LALTASGKAE PFNVSTPGDP STANGTPYIL AHDVQIKDGT TNIDAVAGVL
101 EAAYLKSSVV TTAEPGRVVV TQDFIDASNG RFHLR
```

Unformatted sequence string: **135 residues** (for pasting into other applications).

Sort peptides by ◉ Residue Number ○ Increasing Mass ○ Decreasing Mass

Show predicted peptides also

Query	Start – End	Observed	Mr(expt)	Mr(calc)	ppm	M	Score	Expect	Rank	U	Peptide
2916	27 – 36	473.7660	945.5175	945.5131	4.60	0	52	6.4e-06	1	U	K.VGGVTLDASK.F
4837	47 – 58	523.7960	1045.5775	1045.5768	0.70	0	79	1.3e-08	1	U	K.AGSALALTASGK.A
4839	47 – 58	523.7974	1045.5803	1045.5768	3.42	0	86	2.6e-09	1	U	K.AGSALALTASGK.A
5948	107 – 117	552.2900	1102.5654	1102.5619	3.18	0	84	3.6e-09	1	U	K.SSVVTTAEPGR.V
5956	107 – 117	552.2909	1102.5673	1102.5619	4.98	0	91	7.2e-10	1	U	K.SSVVTTAEPGR.V

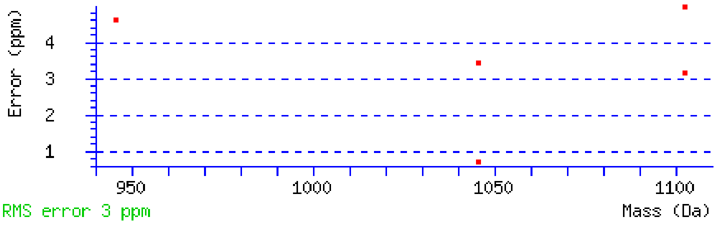

MASCOT Search Results

Protein View: TP84_11

**Database:**	TP84
**Score:**	449
**Nominal mass (M_r):**	14016
**Calculated pI:**	5.84

Sequence similarity is available as **an NCBI BLAST search of 66. against nr**.

Search parameters

**MS data file:**	O:\FA\02-luty2018\80202242zeb_czas10.raw
**Enzyme:**	Trypsin: cuts C-term side of KR unless next residue is P.
**Fixed modifications:**	Carbamidomethyl (C)
**Variable modifications:**	Oxidation (M)

Protein sequence coverage: 17%

Matched peptides shown in ***bold red***.

```
  1 MNLQPKIVSI AGQKEFLATT QGLVHKVGGV TLDASKFTPD ENGFIKAGSA
 51 LALTASGKAE PFNVSTPGDP STANGTPYIL AHDVQIKDGT TNIDAVAGVL
101 EAAYLKSSVV TTAEPGRVVV TQDFIDASNG RFHLR
```

Unformatted sequence string: **135 residues** (for pasting into other applications).

Sort peptides by ◉ Residue Number ○ Increasing Mass ○ Decreasing Mass

Show predicted peptides also

Query	Start – End	Observed	Mr(expt)	Mr(calc)	ppm	M	Score	Expect	Rank	U	Peptide
4756	47 – 58	523.7957	1045.5767	1045.5768	-0.0038	0	62	6.1e-07	1	U	K.AGSALALTASGK.A
4757	47 – 58	523.7967	1045.5788	1045.5768	1.97	0	93	4.8e-10	1	U	K.AGSALALTASGK.A
4759	47 – 58	523.7970	1045.5795	1045.5768	2.64	0	85	2.8e-09	1	U	K.AGSALALTASGK.A
4760	47 – 58	523.7977	1045.5809	1045.5768	3.99	0	102	5.7e-11	1	U	K.AGSALALTASGK.A
5831	107 – 117	552.2892	1102.5639	1102.5619	1.86	0	85	3e-09	1	U	K.SSVVTTAEPGR.V
5834	107 – 117	552.2899	1102.5651	1102.5619	2.98	0	85	3e-09	1	U	K.SSVVTTAEPGR.V

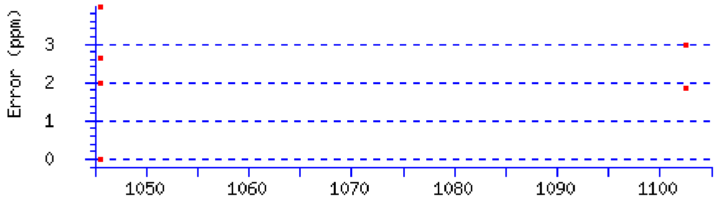

RMS error 2 ppm

Mascot: http://www.matrixscience.com/

MASCOT Search Results

Protein View: TP84_11

**Database:**	TP84
**Score:**	606
**Nominal mass (M_r):**	14016
**Calculated pI:**	5.84

Sequence similarity is available as an NCBI BLAST search of 66. against nr.

Search parameters

**MS data file:**	O:\FA\02-luty2018\80202243zeb_czas15.raw
**Enzyme:**	Trypsin: cuts C-term side of KR unless next residue is P.
**Fixed modifications:**	Carbamidomethyl (C)
**Variable modifications:**	Oxidation (M)

Protein sequence coverage: 33%

Matched peptides shown in ***bold red***.

```
  1 MNLQPKIVSI AGQKEFLATT QGLVHKVGGV TLDASKFTPD ENGFIKAGSA
 51 LALTASGKAE PFNVSTPGDP STANGTPYIL AHDVQIKDGT TNIDAVAGVL
101 EAAYLKSSVV TTAEPGRVVV TQDFIDASNG RFHLR
```

Unformatted sequence string: 135 residues (for pasting into other applications).

Sort peptides by ◉ Residue Number ○ Increasing Mass ○ Decreasing Mass

Show predicted peptides also

Query	Start - End	Observed	Mr(expt)	Mr(calc)	ppm	M	Score	Expect	Rank	U	Peptide
11185	15 - 26	448.5821	1342.7245	1342.7245	-0.00074	0	49	1.2e-05	1	U	K.EFLATTQGLVHK.V
2852	27 - 36	473.7643	945.5140	945.5131	0.96	0	79	1.2e-08	1	U	K.VGGVTLDASK.F
4756	47 - 58	523.7964	1045.5783	1045.5768	1.51	0	104	3.7e-11	1	U	K.AGSALALTASGK.A
4757	47 - 58	523.7966	1045.5787	1045.5768	1.85	0	94	3.6e-10	1	U	K.AGSALALTASGK.A
4758	47 - 58	523.7973	1045.5801	1045.5768	3.23	0	95	3e-10	1	U	K.AGSALALTASGK.A
5781	107 - 117	552.2887	1102.5628	1102.5619	0.90	0	92	6.7e-10	1	U	K.SSVVTTAEPGR.V
5788	107 - 117	552.2896	1102.5646	1102.5619	2.53	0	92	6.7e-10	1	U	K.SSVVTTAEPGR.V
5790	107 - 117	552.2902	1102.5659	1102.5619	3.64	0	92	7.1e-10	1	U	K.SSVVTTAEPGR.V

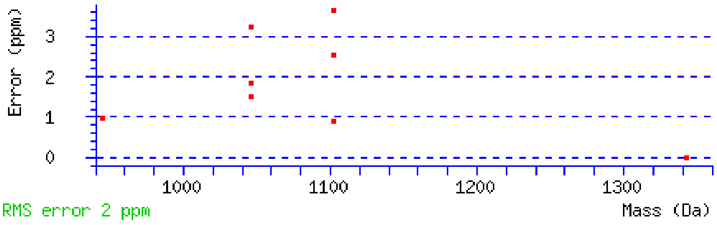

Mascot: http://www.matrixscience.com/

MASCOT Search Results

Protein View: TP84_11

Database: TP84
Score: 1304
Nominal mass (M_r): 14016
Calculated pI: 5.84

Sequence similarity is available as **an NCBI BLAST search of 66. against nr**.

Search parameters

MS data file: O:\FA\02-luty2018\80202244zeb_czas20.raw
Enzyme: Trypsin: cuts C-term side of KR unless next residue is P.
Fixed modifications: Carbamidomethyl (C)
Variable modifications: Oxidation (M)

Protein sequence coverage: 30%

Matched peptides shown in ***bold red***.

```
  1 MNLQPKIVSI AGQKEFLATT QGLVHKVGGV TLDASKFTPD ENGFIKAGSA
 51 LALTASGKAE PFNVSTPGDP STANGTPYIL AHDVQIKDGT TNIDAVAGVL
101 EAAYLKSSVV TTAEPGRVVV TQDFIDASNG RFHLR
```

Unformatted sequence string: **135 residues** (for pasting into other applications).

Sort peptides by ◉ Residue Number ○ Increasing Mass ○ Decreasing Mass

Show predicted peptides also

Query	Start – End	Observed	Mr(expt)	Mr(calc)	ppm	M	Score	Expect	Rank	U	Peptide
1952	7 – 14	408.2545	814.4944	814.4912	3.86	0	51	8.1e-06	1	U	K.IVSIAGQK.E
20854	15 – 26	448.5838	1342.7295	1342.7245	3.71	0	84	4.5e-09	1	U	K.EFLATTQGLVHK.V
20855	15 – 26	448.5839	1342.7298	1342.7245	3.95	0	81	7.4e-09	1	U	K.EFLATTQGLVHK.V
20856	15 – 26	672.3726	1342.7307	1342.7245	4.62	0	83	5.5e-09	1	U	K.EFLATTQGLVHK.V
20857	15 – 26	672.3729	1342.7313	1342.7245	5.08	0	80	1e-08	1	U	K.EFLATTQGLVHK.V
5673	27 – 36	473.7648	945.5151	945.5131	2.10	0	76	2.7e-08	1	U	K.VGGVTLDASK.F
5674	27 – 36	473.7655	945.5165	945.5131	3.60	0	75	3e-08	1	U	K.VGGVTLDASK.F
5675	27 – 36	473.7656	945.5166	945.5131	3.64	0	74	4.2e-08	1	U	K.VGGVTLDASK.F
5676	27 – 36	473.7659	945.5173	945.5131	4.47	0	74	4.4e-08	1	U	K.VGGVTLDASK.F
5677	27 – 36	473.7660	945.5174	945.5131	4.55	0	71	8.5e-08	1	U	K.VGGVTLDASK.F
5678	27 – 36	473.7661	945.5175	945.5131	4.68	0	59	1.3e-06	1	U	K.VGGVTLDASK.F
5680	27 – 36	473.7662	945.5179	945.5131	5.06	0	58	1.6e-06	1	U	K.VGGVTLDASK.F
5681	27 – 36	473.7665	945.5184	945.5131	5.61	0	56	2.3e-06	1	U	K.VGGVTLDASK.F
5682	27 – 36	473.7666	945.5186	945.5131	5.84	0	54	3.6e-06	1	U	K.VGGVTLDASK.F
5683	27 – 36	473.7667	945.5188	945.5131	6.01	0	71	8.2e-08	1	U	K.VGGVTLDASK.F
5684	27 – 36	473.7671	945.5196	945.5131	6.86	0	75	3e-08	1	U	K.VGGVTLDASK.F
11487	107 – 117	552.2913	1102.5680	1102.5619	5.60	0	92	6.8e-10	1	U	K.SSVVTTAEPGR.V
11488	107 – 117	552.2915	1102.5685	1102.5619	6.05	0	92	6.7e-10	1	U	K.SSVVTTAEPGR.V
11489	107 – 117	552.2916	1102.5686	1102.5619	6.14	0	92	6.6e-10	1	U	K.SSVVTTAEPGR.V
11490	107 – 117	552.2917	1102.5688	1102.5619	6.34	0	92	6.7e-10	1	U	K.SSVVTTAEPGR.V
11497	107 – 117	552.2922	1102.5699	1102.5619	7.34	0	76	2.5e-08	1	U	K.SSVVTTAEPGR.V

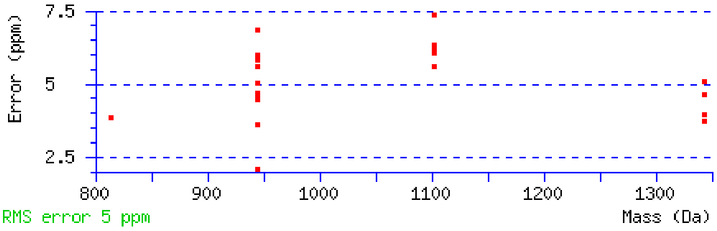

Mascot: http://www.matrixscience.com/

MASCOT Search Results

Protein View: TP84_11

**Database:**	TP84
**Score:**	1935
**Nominal mass (M_r):**	14016
**Calculated pI:**	5.84

Sequence similarity is available as an NCBI BLAST search of 66. against nr.

Search parameters

**MS data file:**	O:\FA\02-luty2018\80202245zeb_czas25.raw
**Enzyme:**	Trypsin: cuts C-term side of KR unless next residue is P.
**Fixed modifications:**	Carbamidomethyl (C)
**Variable modifications:**	Oxidation (M)

Protein sequence coverage: 74%

Matched peptides shown in ***bold red***.

```
  1 MNLQPKIVSI AGQKEFLATT QGLVHKVGGV TLDASKFTPD ENGFIKAGSA
 51 LALTASGKAE PFNVSTPGDP STANGTPYIL AHDVQIKDGT TNIDAVAGVL
101 EAAYLKSSVV TTAEPGRVVV TQDFIDASNG RFHLR
```

Unformatted sequence string: 135 residues (for pasting into other applications).

Sort peptides by ◉ Residue Number ○ Increasing Mass ○ Decreasing Mass

Show predicted peptides also

Query	Start – End	Observed	Mr(expt)	Mr(calc)	ppm	M	Score	Expect	Rank	U	Peptide
1292	7 – 14	408.2525	814.4904	814.4912	-1.02	0	54	3.9e-06	1	U	K.IVSIAGQK.E
13580	15 – 26	448.5822	1342.7246	1342.7245	0.089	0	72	6.7e-08	1	U	K.EFLATTQGLVHK.V
13581	15 – 26	448.5822	1342.7246	1342.7245	0.089	0	57	1.8e-06	1	U	K.EFLATTQGLVHK.V
13582	15 – 26	448.5823	1342.7251	1342.7245	0.42	0	70	1e-07	1	U	K.EFLATTQGLVHK.V
3595	27 – 36	473.7606	945.5067	945.5131	-6.74	0	59	1.3e-06	1	U	K.VGGVTLDASK.F
3596	27 – 36	473.7635	945.5125	945.5131	-0.65	0	90	1.1e-09	1	U	K.VGGVTLDASK.F
3597	27 – 36	473.7636	945.5127	945.5131	-0.42	0	77	1.8e-08	1	U	K.VGGVTLDASK.F
3598	27 – 36	473.7636	945.5127	945.5131	-0.40	0	92	5.9e-10	1	U	K.VGGVTLDASK.F
3599	27 – 36	473.7638	945.5130	945.5131	-0.14	0	80	9.7e-09	1	U	K.VGGVTLDASK.F
3600	27 – 36	473.7639	945.5132	945.5131	0.13	0	80	9.4e-09	1	U	K.VGGVTLDASK.F
3601	27 – 36	473.7640	945.5134	945.5131	0.26	0	88	1.7e-09	1	U	K.VGGVTLDASK.F
3602	27 – 36	473.7643	945.5141	945.5131	1.08	0	67	1.9e-07	1	U	K.VGGVTLDASK.F
5964	47 – 58	523.7958	1045.5771	1045.5768	0.36	0	96	2.5e-10	1	U	K.AGSALALTASGK.A
5965	47 – 58	523.7959	1045.5773	1045.5768	0.55	0	95	3.1e-10	1	U	K.AGSALALTASGK.A
5966	47 – 58	523.7960	1045.5774	1045.5768	0.59	0	96	2.5e-10	1	U	K.AGSALALTASGK.A
5968	47 – 58	523.7961	1045.5777	1045.5768	0.93	0	104	4.3e-11	1	U	K.AGSALALTASGK.A
5969	47 – 58	523.7962	1045.5779	1045.5768	1.07	0	102	6.3e-11	1	U	K.AGSALALTASGK.A
5970	47 – 58	523.7962	1045.5779	1045.5768	1.11	0	70	9.5e-08	1	U	K.AGSALALTASGK.A
36469	59 – 87	1013.8376	3038.4910	3038.4985	-2.49	0	116	2.3e-12	1	U	K.AEPFNVSTPGDPSTANGTPYILAHDVQIK.D
36470	59 – 87	1013.8377	3038.4913	3038.4985	-2.39	0	106	2.6e-11	1	U	K.AEPFNVSTPGDPSTANGTPYILAHDVQIK.D
36471	59 – 87	1013.8383	3038.4931	3038.4985	-1.80	0	120	1.1e-12	1	U	K.AEPFNVSTPGDPSTANGTPYILAHDVQIK.D
36472	59 – 87	1013.8385	3038.4937	3038.4985	-1.60	0	99	1.2e-10	1	U	K.AEPFNVSTPGDPSTANGTPYILAHDVQIK.D
26795	88 – 106	641.0018	1919.9835	1919.9840	-0.27	0	93	4.8e-10	1	U	K.DGTTNIDAVAGVLEAAYLK.S
7174	107 – 117	552.2880	1102.5615	1102.5619	-0.35	0	92	6.6e-10	1	U	K.SSVVTTAEPGR.V
7175	107 – 117	552.2882	1102.5618	1102.5619	-0.083	0	92	6.5e-10	1	U	K.SSVVTTAEPGR.V
7176	107 – 117	552.2886	1102.5626	1102.5619	0.72	0	92	6.6e-10	1	U	K.SSVVTTAEPGR.V

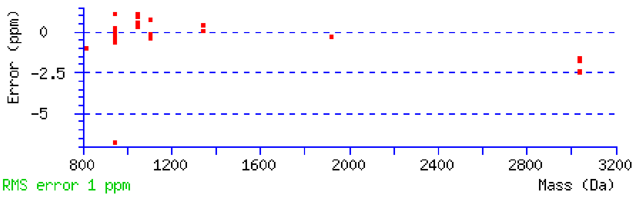

Mascot: http://www.matrixscience.com/

MASCOT Search Results

Protein View: TP84_11

**Database:**	TP84
**Score:**	4174
**Nominal mass (M_r):**	14016
**Calculated pI:**	5.84

Sequence similarity is available as **an NCBI BLAST search of 66. against nr**.

Search parameters

**MS data file:**	O:\FA\02-luty2018\80202246zeb_czas30.raw
**Enzyme:**	Trypsin: cuts C-term side of KR unless next residue is P.
**Fixed modifications:**	**Carbamidomethyl (C)**
**Variable modifications:**	**Oxidation (M)**

Protein sequence coverage: 85%

Matched peptides shown in ***bold red***.

```
  1 MNLQPKIVSI AGQKEFLATT QGLVHKVGGV TLDASKFTPD ENGFIKAGSA
 51 LALTASGKAE PFNVSTPGDP STANGTPYIL AHDVQIKDGT TNIDAVAGVL
101 EAAYLKSSVV TTAEPGRVVV TQDFIDASNG RFHLR
```

Unformatted sequence string: **135 residues** (for pasting into other applications).

Sort peptides by ◉ Residue Number ○ Increasing Mass ○ Decreasing Mass

Show predicted peptides also

Query	Start – End	Observed	Mr(expt)	Mr(calc)	ppm	M	Score	Expect	Rank	U	Peptide
1539	7 – 14	408.2524	814.4902	814.4912	-1.22	0	61	8.3e-07	1	U	K.IVSIAGQK.E
1543	7 – 14	408.2529	814.4913	814.4912	0.13	0	60	1.1e-06	1	U	K.IVSIAGQK.E
19130	15 – 26	448.5815	1342.7226	1342.7245	-1.41	0	76	2.7e-08	1	U	K.EFLATTQGLVHK.V
19131	15 – 26	448.5815	1342.7228	1342.7245	-1.25	0	75	3.5e-08	1	U	K.EFLATTQGLVHK.V
19132	15 – 26	672.3687	1342.7228	1342.7245	-1.23	0	80	9.4e-09	1	U	K.EFLATTQGLVHK.V
19133	15 – 26	448.5818	1342.7235	1342.7245	-0.78	0	78	1.4e-08	1	U	K.EFLATTQGLVHK.V
19134	15 – 26	672.3692	1342.7239	1342.7245	-0.41	0	77	1.8e-08	1	U	K.EFLATTQGLVHK.V
19135	15 – 26	448.5819	1342.7240	1342.7245	-0.40	0	83	5.6e-09	1	U	K.EFLATTQGLVHK.V
19136	15 – 26	448.5820	1342.7242	1342.7245	-0.25	0	75	3.1e-08	1	U	K.EFLATTQGLVHK.V
19137	15 – 26	448.5820	1342.7243	1342.7245	-0.16	0	77	2.1e-08	1	U	K.EFLATTQGLVHK.V
19138	15 – 26	672.3696	1342.7246	1342.7245	0.063	0	83	5.5e-09	1	U	K.EFLATTQGLVHK.V
19139	15 – 26	448.5822	1342.7248	1342.7245	0.25	0	74	4.4e-08	1	U	K.EFLATTQGLVHK.V
19140	15 – 26	672.3698	1342.7250	1342.7245	0.41	0	83	5.3e-09	1	U	K.EFLATTQGLVHK.V
19141	15 – 26	448.5824	1342.7254	1342.7245	0.65	0	75	3.4e-08	1	U	K.EFLATTQGLVHK.V
19142	15 – 26	672.3700	1342.7255	1342.7245	0.72	0	78	1.8e-08	1	U	K.EFLATTQGLVHK.V
19143	15 – 26	672.3703	1342.7261	1342.7245	1.17	0	80	1.1e-08	1	U	K.EFLATTQGLVHK.V
19144	15 – 26	672.3703	1342.7261	1342.7245	1.21	0	63	5.6e-07	1	U	K.EFLATTQGLVHK.V
4565	27 – 36	473.7636	945.5127	945.5131	-0.46	0	78	1.5e-08	1	U	K.VGGVTLDASK.F
4566	27 – 36	473.7637	945.5129	945.5131	-0.27	0	76	2.6e-08	1	U	K.VGGVTLDASK.F
4567	27 – 36	473.7638	945.5130	945.5131	-0.079	0	63	5.2e-07	1	U	K.VGGVTLDASK.F
4568	27 – 36	473.7639	945.5132	945.5131	0.090	0	75	2.9e-08	1	U	K.VGGVTLDASK.F
4569	27 – 36	473.7640	945.5135	945.5131	0.41	0	71	8.3e-08	1	U	K.VGGVTLDASK.F
4570	27 – 36	473.7641	945.5136	945.5131	0.53	0	77	1.8e-08	1	U	K.VGGVTLDASK.F
4571	27 – 36	473.7641	945.5137	945.5131	0.64	0	82	6.9e-09	1	U	K.VGGVTLDASK.F
4572	27 – 36	473.7644	945.5142	945.5131	1.15	0	64	3.7e-07	1	U	K.VGGVTLDASK.F
4573	27 – 36	473.7644	945.5143	945.5131	1.23	0	82	5.8e-09	1	U	K.VGGVTLDASK.F
7964	47 – 58	523.7956	1045.5766	1045.5768	-0.16	0	84	3.7e-09	1	U	K.AGSALALTASGK.A
7965	47 – 58	523.7957	1045.5768	1045.5768	0.015	0	84	4.3e-09	1	U	K.AGSALALTASGK.A
7966	47 – 58	523.7957	1045.5769	1045.5768	0.19	0	105	3e-11	1	U	K.AGSALALTASGK.A
7967	47 – 58	523.7958	1045.5771	1045.5768	0.34	0	90	1.1e-09	1	U	K.AGSALALTASGK.A
7968	47 – 58	523.7958	1045.5771	1045.5768	0.38	0	85	2.9e-09	1	U	K.AGSALALTASGK.A
7969	47 – 58	523.7959	1045.5773	1045.5768	0.49	0	103	4.7e-11	1	U	K.AGSALALTASGK.A
7970	47 – 58	523.7959	1045.5773	1045.5768	0.57	0	83	4.7e-09	1	U	K.AGSALALTASGK.A
7971	47 – 58	523.7960	1045.5774	1045.5768	0.61	0	85	2.9e-09	1	U	K.AGSALALTASGK.A
7972	47 – 58	523.7961	1045.5777	1045.5768	0.90	0	85	3e-09	1	U	K.AGSALALTASGK.A
7973	47 – 58	523.7962	1045.5778	1045.5768	0.99	0	87	1.9e-09	1	U	K.AGSALALTASGK.A
7974	47 – 58	523.7962	1045.5779	1045.5768	1.11	0	96	2.6e-10	1	U	K.AGSALALTASGK.A
7975	47 – 58	523.7962	1045.5779	1045.5768	1.14	0	87	2.1e-09	1	U	K.AGSALALTASGK.A
7976	47 – 58	523.7963	1045.5780	1045.5768	1.20	0	84	4.3e-09	1	U	K.AGSALALTASGK.A
7977	47 – 58	523.7965	1045.5785	1045.5768	1.66	0	86	2.4e-09	1	U	K.AGSALALTASGK.A
7978	47 – 58	523.7966	1045.5785	1045.5768	1.72	0	99	1.4e-10	1	U	K.AGSALALTASGK.A
7979	47 – 58	523.7966	1045.5786	1045.5768	1.74	0	86	2.3e-09	1	U	K.AGSALALTASGK.A
7980	47 – 58	523.7966	1045.5787	1045.5768	1.85	0	84	4.2e-09	1	U	K.AGSALALTASGK.A
7981	47 – 58	523.7968	1045.5790	1045.5768	2.18	0	75	3e-08	1	U	K.AGSALALTASGK.A
46599	59 – 87	1013.8386	3038.4940	3038.4985	-1.50	0	126	2.4e-13	1	U	K.AEPFNVSTPGDPSTANGTPYILAHDVQIK.D
46600	59 – 87	1013.8396	3038.4970	3038.4985	-0.51	0	91	7.6e-10	1	U	K.AEPFNVSTPGDPSTANGTPYILAHDVQIK.D
46601	59 – 87	1013.8402	3038.4988	3038.4985	0.078	0	109	1.4e-11	1	U	K.AEPFNVSTPGDPSTANGTPYILAHDVQIK.D
35818	88 – 106	641.0026	1919.9859	1919.9840	1.01	0	113	5.6e-12	1	U	K.DGTTNIDAVAGVLEAAYLK.S

Query	Start – End	Observed	Mr(expt)	Mr(calc)	ppm	M	Score	Expect	Rank	U	Peptide
35819	88 – 106	641.0033	1919.9880	1919.9840	2.07	0	99	1.2e-10	1	U	K.DGTTNIDAVAGVLEAAYLK.S
35820	88 – 106	641.0046	1919.9920	1919.9840	4.18	0	93	5.6e-10	1	U	K.DGTTNIDAVAGVLEAAYLK.S
9849	107 – 117	552.2883	1102.5620	1102.5619	0.17	0	92	6.6e-10	1	U	K.SSVVTTAEPGR.V
9850	107 – 117	552.2885	1102.5624	1102.5619	0.52	0	92	6.5e-10	1	U	K.SSVVTTAEPGR.V
9851	107 – 117	552.2885	1102.5625	1102.5619	0.55	0	92	6.5e-10	1	U	K.SSVVTTAEPGR.V
9853	107 – 117	552.2886	1102.5627	1102.5619	0.81	0	92	6.5e-10	1	U	K.SSVVTTAEPGR.V
9855	107 – 117	552.2891	1102.5636	1102.5619	1.59	0	92	6.5e-10	1	U	K.SSVVTTAEPGR.V
25079	118 – 131	760.8823	1519.7500	1519.7631	-8.62	0	62	6.4e-07	1	U	R.VVVTQDFIDASNGR.F
25080	118 – 131	760.8823	1519.7501	1519.7631	-8.57	0	62	6.3e-07	1	U	R.VVVTQDFIDASNGR.F
25081	118 – 131	760.8831	1519.7517	1519.7631	-7.48	0	70	1.1e-07	1	U	R.VVVTQDFIDASNGR.F
25082	118 – 131	760.8832	1519.7517	1519.7631	-7.46	0	64	4e-07	1	U	R.VVVTQDFIDASNGR.F
25083	118 – 131	760.8839	1519.7532	1519.7631	-6.53	0	62	6.8e-07	1	U	R.VVVTQDFIDASNGR.F

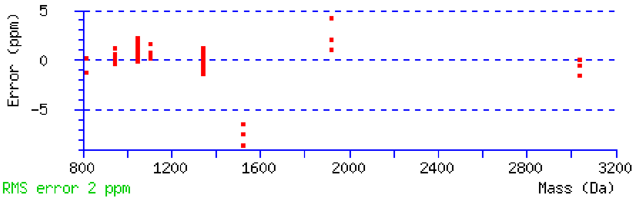

Mascot: http://www.matrixscience.com/

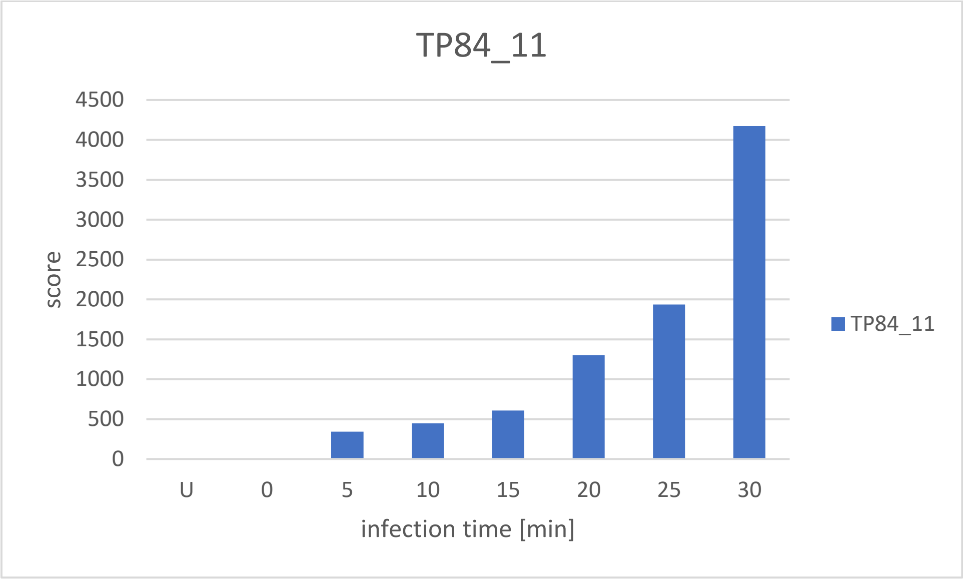

MASCOT Search Results

Protein View: TP84_12

Database: TP84
Score: 164
Nominal mass (M_r): 37757
Calculated pI: 4.98

Sequence similarity is available as **an NCBI BLAST search of 67. against nr**.

Search parameters

MS data file: O:\FA\02-luty2018\80202240zeb_czas0.raw
Enzyme: Trypsin: cuts C-term side of KR unless next residue is P.
Fixed modifications: **Carbamidomethyl (C)**
Variable modifications: **Oxidation (M)**

Protein sequence coverage: 16%

Matched peptides shown in ***bold red***.

```
  1 MPLHLEQFQR EAFQGYVENV PPKREYALAK FMPNQPVYDI EFTYNIINGG
 51 YGQMASITAW DSGAPLRDKD VIQRLTAQIA KVQHAYRLTE KELLMFHRPR
101 MDEEQQQVIQ AIYNNTDKLV WGVQDREEWL RAKAVYVGQL QYSENDVQLN
151 IDFLIPAENK LTADVDWSDP TAPVIQHLQS AVQRFKEANN GEKPVEMHMS
201 SRVETWLLQN EQVKAHIYGN TTDPRIVTSE QLQQLFSALS LPPYRVIDEQ
251 VIGENGAEAL MPEDRVVLLG EELGHTMEGP TVENNYKPGI YVIPEIKETN
301 PPRQEVYVGK SVFPALERPQ AVVHLIVAQS
```

Unformatted sequence string: **330 residues** (for pasting into other applications).

Sort peptides by ◉ Residue Number ○ Increasing Mass ○ Decreasing Mass

Show predicted peptides also

Query	Start – End	Observed	Mr(expt)	Mr(calc)	ppm	M	Score	Expect	Rank	U	Peptide
31317	161 – 184	883.1239	2646.3498	2646.3402	3.62	0	119	1.2e-12	1	U	K.LTADVDWSDPTAPVIQHLQSAVQR.F
34727	266 – 297	885.4711	3537.8553	3537.8429	3.51	0	58	1.7e-06	1	U	R.VVLLGEELGHTMEGPTVENNYKPGIYVIPEIK.E

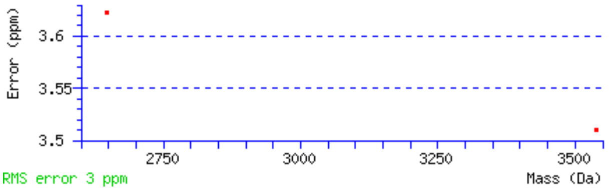

Mascot: **http://www.matrixscience.com/**

MASCOT Search Results

Protein View: TP84_12

Database: TP84
Score: 181
Nominal mass (M_r): 37757
Calculated pI: 4.98

Sequence similarity is available as an NCBI BLAST search of 67. against nr.

Search parameters

MS data file: O:\FA\02-luty2018\80202242zeb_czas10.raw
Enzyme: Trypsin: cuts C-term side of KR unless next residue is P.
Fixed modifications: Carbamidomethyl (C)
Variable modifications: Oxidation (M)

Protein sequence coverage: 20%

Matched peptides shown in ***bold red***.

```
  1 MPLHLEQFQR EAFQGYVENV PPKREYALAK FMPNQPVYDI EFTYNIINGG
 51 YGQMASITAW DSGAPLRDKD VIQRLTAQIA KVQHAYRLTE KELLMFHRPR
101 MDEEQQQVIQ AIYNNTDKLV WGVQDREEWL RAKAVYVGQL QYSENDVQLN
151 IDFLIPAENK LTADVDWSDP TAPVIQHLQS AVQRFKEANN GEKPVEMHMS
201 SRVETWLLQN EQVKAHIYGN TTDPRIVTSE QLQQLFSALS LPPYRVIDEQ
251 VIGENGAEAL MPEDRVVLLG EELGHTMEGP TVENNYKPGI YVIPEIKETN
301 PPRQEVYVGK SVFPALERPQ AVVHLIVAQS
```

Unformatted sequence string: 330 residues (for pasting into other applications).

Sort peptides by ◉ Residue Number ○ Increasing Mass ○ Decreasing Mass

Show predicted peptides also

Query	Start – End	Observed	Mr(expt)	Mr(calc)	ppm	M	Score	Expect	Rank	U	Peptide
29771	161 – 184	883.1192	2646.3357	2646.3402	-1.69	0	109	1.4e-11	1	U	K.LTADVDWSDPTAPVIQHLQSAVQR.F
8994	215 – 225	622.8062	1243.5978	1243.5945	2.64	0	28	0.0015	1	U	K.AHIYGNTTDPR.I
32972	266 – 297	885.4699	3537.8505	3537.8429	2.16	0	70	9.7e-08	1	U	R.VVLLGEELGHTMEGPTVENNYKPGIYVIPEIK.E

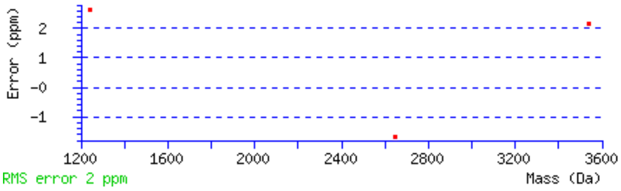

Mascot: http://www.matrixscience.com/

MASCOT Search Results

Protein View: TP84_12

Database: TP84
Score: 476
Nominal mass (M_r): 37757
Calculated pI: 4.98

Sequence similarity is available as **an NCBI BLAST search of 67. against nr**.

Search parameters

MS data file: O:\FA\02-luty2018\80202243zeb_czas15.raw
Enzyme: Trypsin: cuts C-term side of KR unless next residue is P.
Fixed modifications: **Carbamidomethyl (C)**
Variable modifications: **Oxidation (M)**

Protein sequence coverage: 22%

Matched peptides shown in ***bold red***.

```
  1 MPLHLEQFQR EAFQGYVENV PPKREYALAK FMPNQPVYDI EFTYNIINGG
 51 YGQMASITAW DSGAPLRDKD VIQRLTAQIA KVQHAYRLTE KELLMFHRPR
101 MDEEQQQVIQ AIYNNTDKLV WGVQDREEWL RAKAVYVGQL QYSENDVQLN
151 IDFLIPAENK LTADVDWSDP TAPVIQHLQS AVQRFKEANN GEKPVEMHMS
201 SRVETWLLQN EQVKAHIYGN TTDPRIVTSE QLQQLFSALS LPPYRVIDEQ
251 VIGENGAEAL MPEDRVVLLG EELGHTMEGP TVENNYKPGI YVIPEIKETN
301 PPRQEVYVGK SVFPALERPQ AVVHLIVAQS
```

Unformatted sequence string: **330 residues** (for pasting into other applications).

Sort peptides by ◉ Residue Number ○ Increasing Mass ○ Decreasing Mass

Show predicted peptides also

Query	Start – End	Observed	Mr(expt)	Mr(calc)	ppm	M	Score	Expect	Rank	U	Peptide
3419	119 – 126	486.7736	971.5326	971.5189	14.1	0	14	0.043	1	U	K.LVWGVQDR.E
29561	161 – 184	883.1193	2646.3360	2646.3402	-1.58	0	108	1.6e-11	1	U	K.LTADVDWSDPTAPVIQHLQSAVQR.F
29562	161 – 184	883.1199	2646.3379	2646.3402	-0.86	0	122	6.1e-13	1	U	K.LTADVDWSDPTAPVIQHLQSAVQR.F
29563	161 – 184	883.1217	2646.3431	2646.3402	1.11	0	127	2e-13	1	U	K.LTADVDWSDPTAPVIQHLQSAVQR.F
8898	215 – 225	622.8043	1243.5941	1243.5945	-0.38	0	28	0.0014	1	U	K.AHIYGNTTDPR.I
32877	266 – 297	885.4684	3537.8444	3537.8429	0.42	0	42	5.7e-05	1	U	R.VVLLGEELGHTMEGPTVENNYKPGIYVIPEIK.E
32878	266 – 297	885.4690	3537.8469	3537.8429	1.12	0	40	0.0001	1	U	R.VVLLGEELGHTMEGPTVENNYKPGIYVIPEIK.E
32879	266 – 297	885.4692	3537.8476	3537.8429	1.33	0	55	3.1e-06	1	U	R.VVLLGEELGHTMEGPTVENNYKPGIYVIPEIK.E
32880	266 – 297	885.4708	3537.8539	3537.8429	3.13	0	44	4.1e-05	1	U	R.VVLLGEELGHTMEGPTVENNYKPGIYVIPEIK.E

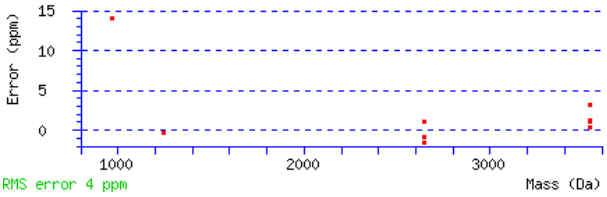

Mascot: **http://www.matrixscience.com/**

MASCOT Search Results

Protein View: TP84_12

Database: TP84
Score: 1547
Nominal mass (M_r): 37757
Calculated pI: 4.98

Sequence similarity is available as **an NCBI BLAST search of 67. against nr**.

Search parameters

MS data file: O:\FA\02-luty2018\80202244zeb_czas20.raw
Enzyme: Trypsin: cuts C-term side of KR unless next residue is P.
Fixed modifications: Carbamidomethyl (C)
Variable modifications: Oxidation (M)

Protein sequence coverage: 28%

Matched peptides shown in ***bold red***.

```
  1 MPLHLEQFQR EAFQGYVENV PPKREYALAK FMPNQPVYDI EFTYNIINGG
 51 YGQMASITAW DSGAPLRDKD VIQRLTAQIA KVQHAYRLTE KELLMFHRPR
101 MDEEQQQVIQ AIYNNTDKLV WGVQDREEWL RAKAVYVGQL QYSENDVQLN
151 IDFLIPAENK LTADVDWSDP TAPVIQHLQS AVQRFKEANN GEKPVEMHMS
201 SRVETWLLQN EQVKAHIYGN TTDPRIVTSE QLQQLFSALS LPPYRVIDEQ
251 VIGENGAEAL MPEDRVVLLG EELGHTMEGP TVENNYKPGI YVIPEIKETN
301 PPRQEVYVGK SVFPALERPQ AVVHLIVAQS
```

Unformatted sequence string: **330 residues** (for pasting into other applications).

Sort peptides by ◉ Residue Number ○ Increasing Mass ○ Decreasing Mass

Show predicted peptides also

Query	Start – End	Observed	Mr(expt)	Mr(calc)	ppm	M	Score	Expect	Rank	U	Peptide
13816	2 – 10	389.8817	1166.6232	1166.6196	3.07	0	58	1.7e-06	1	U	M.PLHLEQFQR.E
13817	2 – 10	389.8820	1166.6241	1166.6196	3.79	0	58	1.7e-06	1	U	M.PLHLEQFQR.E
13818	2 – 10	389.8822	1166.6248	1166.6196	4.43	0	58	1.7e-06	1	U	M.PLHLEQFQR.E
39909	101 – 118	1084.0054	2165.9962	2165.9899	2.94	0	108	1.5e-11	1	U	R.MDEEQQQVIQAIYNNTDK.L
39910	101 – 118	1084.0112	2166.0078	2165.9899	8.29	0	116	2.4e-12	1	U	R.MDEEQQQVIQAIYNNTDK.L
43995	161 – 184	883.1211	2646.3415	2646.3402	0.49	0	111	7.2e-12	1	U	K.LTADVDWSDPTAPVIQHLQSAVQR.F
43996	161 – 184	883.1216	2646.3429	2646.3402	1.02	0	83	5.3e-09	1	U	K.LTADVDWSDPTAPVIQHLQSAVQR.F
43997	161 – 184	883.1216	2646.3429	2646.3402	1.03	0	124	4.4e-13	1	U	K.LTADVDWSDPTAPVIQHLQSAVQR.F
43998	161 – 184	883.1217	2646.3434	2646.3402	1.21	0	132	6.9e-14	1	U	K.LTADVDWSDPTAPVIQHLQSAVQR.F
43999	161 – 184	883.1223	2646.3452	2646.3402	1.89	0	93	4.9e-10	1	U	K.LTADVDWSDPTAPVIQHLQSAVQR.F
44000	161 – 184	883.1225	2646.3456	2646.3402	2.02	0	135	3.3e-14	1	U	K.LTADVDWSDPTAPVIQHLQSAVQR.F
44001	161 – 184	883.1225	2646.3457	2646.3402	2.08	0	137	2.2e-14	1	U	K.LTADVDWSDPTAPVIQHLQSAVQR.F
44002	161 – 184	883.1230	2646.3471	2646.3402	2.59	0	142	6.2e-15	1	U	K.LTADVDWSDPTAPVIQHLQSAVQR.F
44003	161 – 184	883.1241	2646.3504	2646.3402	3.85	0	58	1.4e-06	1	U	K.LTADVDWSDPTAPVIQHLQSAVQR.F
16856	215 – 225	622.8080	1243.6013	1243.5945	5.47	0	65	3.4e-07	1	U	K.AHIYGNTTDPR.I
47347	266 – 297	885.4690	3537.8470	3537.8429	1.17	0	60	1e-06	1	U	R.VVLLGEELGHTMEGPTVENNYKPGIYVIPEIK.E
47348	266 – 297	885.4695	3537.8490	3537.8429	1.72	0	61	7.4e-07	1	U	R.VVLLGEELGHTMEGPTVENNYKPGIYVIPEIK.E
47349	266 – 297	885.4699	3537.8506	3537.8429	2.18	0	57	1.8e-06	1	U	R.VVLLGEELGHTMEGPTVENNYKPGIYVIPEIK.E
47350	266 – 297	1180.2910	3537.8512	3537.8429	2.34	0	81	8e-09	1	U	R.VVLLGEELGHTMEGPTVENNYKPGIYVIPEIK.E
47351	266 – 297	885.4710	3537.8549	3537.8429	3.39	0	58	1.7e-06	1	U	R.VVLLGEELGHTMEGPTVENNYKPGIYVIPEIK.E

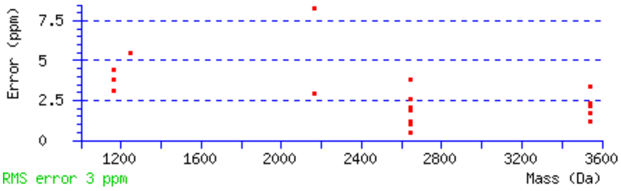

Mascot: http://www.matrixscience.com/

MASCOT Search Results

Protein View: TP84_12

Database: TP84
Score: 2724
Nominal mass (M_r): 37757
Calculated pI: 4.98

Sequence similarity is available as **an NCBI BLAST search of 67. against nr**.

Search parameters

MS data file: O:\FA\02-luty2018\80202245zeb_czas25.raw
Enzyme: Trypsin: cuts C-term side of KR unless next residue is P.
Fixed modifications: **Carbamidomethyl (C)**
Variable modifications: **Oxidation (M)**

Protein sequence coverage: 49%

Matched peptides shown in ***bold red***.

```
  1 MPLHLEQFQR EAFQGYVENV PPKREYALAK FMPNQPVYDI EFTYNIINGG
 51 YGQMASITAW DSGAPLRDKD VIQRLTAQIA KVQHAYRLTE KELLMFHRPR
101 MDEEQQQVIQ AIYNNTDKLV WGVQDREEWL RAKAVYVGQL QYSENDVQLN
151 IDFLIPAENK LTADVDWSDP TAPVIQHLQS AVQRFKEANN GEKPVEMHMS
201 SRVETWLLQN EQVKAHIYGN TTDPRIVTSE QLQQLFSALS LPPYRVIDEQ
251 VIGENGAEAL MPEDRVVLLG EELGHTMEGP TVENNYKPGI YVIPEIKETN
301 PPRQEVYVGK SVFPALERPQ AVVHLIVAQS
```

Unformatted sequence string: **330 residues** (for pasting into other applications).

Sort peptides by ◉ Residue Number ○ Increasing Mass ○ Decreasing Mass

Show predicted peptides also

Query	Start – End	Observed	Mr(expt)	Mr(calc)	ppm	M	Score	Expect	Rank	U	Peptide
8703	2 – 10	389.8800	1166.6182	1166.6196	-1.20	0	58	1.7e-06	1	U	M.PLHLEQFQR.E
8704	2 – 10	389.8802	1166.6187	1166.6196	-0.81	0	57	1.9e-06	1	U	M.PLHLEQFQR.E
8705	2 – 10	389.8802	1166.6189	1166.6196	-0.63	0	58	1.7e-06	1	U	M.PLHLEQFQR.E
8706	2 – 10	389.8805	1166.6196	1166.6196	-0.015	0	58	1.8e-06	1	U	M.PLHLEQFQR.E
8707	2 – 10	584.3181	1166.6217	1166.6196	1.75	0	61	7.2e-07	1	U	M.PLHLEQFQR.E
8708	2 – 10	584.3182	1166.6219	1166.6196	1.95	0	57	1.9e-06	1	U	M.PLHLEQFQR.E
16970	11 – 23	739.3652	1476.7159	1476.7249	-6.05	0	53	5.5e-06	1	U	R.EAFQGYVENVPPK.R
16973	11 – 23	739.3667	1476.7187	1476.7249	-4.16	0	72	5.9e-08	1	U	R.EAFQGYVENVPPK.R
16977	11 – 23	739.3706	1476.7266	1476.7249	1.17	0	67	1.8e-07	1	U	R.EAFQGYVENVPPK.R
16978	11 – 23	739.3706	1476.7267	1476.7249	1.21	0	68	1.7e-07	1	U	R.EAFQGYVENVPPK.R
30492	101 – 118	1083.9981	2165.9816	2165.9899	-3.80	0	117	2e-12	1	U	R.MDEEQQQVIQAIYNNTDK.L
30493	101 – 118	1083.9984	2165.9822	2165.9899	-3.52	0	115	3.1e-12	1	U	R.MDEEQQQVIQAIYNNTDK.L
30494	101 – 118	723.0019	2165.9840	2165.9899	-2.71	0	56	2.6e-06	1	U	R.MDEEQQQVIQAIYNNTDK.L
30495	101 – 118	1083.9997	2165.9848	2165.9899	-2.32	0	129	1.4e-13	1	U	R.MDEEQQQVIQAIYNNTDK.L
34495	161 – 184	883.1196	2646.3369	2646.3402	-1.26	0	136	2.5e-14	1	U	K.LTADVDWSDPTAPVIQHLQSAVQR.F
34496	161 – 184	883.1196	2646.3371	2646.3402	-1.16	0	143	5.6e-15	1	U	K.LTADVDWSDPTAPVIQHLQSAVQR.F
34497	161 – 184	883.1200	2646.3382	2646.3402	-0.74	0	139	1.2e-14	1	U	K.LTADVDWSDPTAPVIQHLQSAVQR.F
34498	161 – 184	883.1209	2646.3408	2646.3402	0.24	0	151	7.8e-16	1	U	K.LTADVDWSDPTAPVIQHLQSAVQR.F
34499	161 – 184	883.1213	2646.3421	2646.3402	0.73	0	140	1e-14	1	U	K.LTADVDWSDPTAPVIQHLQSAVQR.F
25060	187 – 202	605.9417	1814.8032	1814.8039	-0.41	0	85	3.4e-09	1	U	K.EANNGEKPVEMHMSSR.V
17287	203 – 214	743.8986	1485.7827	1485.7827	-0.0013	0	69	1.3e-07	1	U	R.VETWLLQNEQVK.A
17288	203 – 214	743.8990	1485.7834	1485.7827	0.46	0	65	3.1e-07	1	U	R.VETWLLQNEQVK.A
17289	203 – 214	743.8991	1485.7836	1485.7827	0.60	0	65	3e-07	1	U	R.VETWLLQNEQVK.A
17290	203 – 214	743.8991	1485.7837	1485.7827	0.63	0	75	2.9e-08	1	U	R.VETWLLQNEQVK.A
17291	203 – 214	743.8995	1485.7844	1485.7827	1.16	0	62	6.3e-07	1	U	R.VETWLLQNEQVK.A
17292	203 – 214	743.8996	1485.7847	1485.7827	1.32	0	64	3.7e-07	1	U	R.VETWLLQNEQVK.A
17293	203 – 214	743.8997	1485.7849	1485.7827	1.45	0	61	7.8e-07	1	U	R.VETWLLQNEQVK.A
10832	215 – 225	622.8047	1243.5947	1243.5945	0.16	0	64	3.6e-07	1	U	K.AHIYGNTTDPR.I
10833	215 – 225	622.8050	1243.5954	1243.5945	0.70	0	60	1e-06	1	U	K.AHIYGNTTDPR.I
37983	266 – 297	1180.2857	3537.8353	3537.8429	-2.15	0	105	3.5e-11	1	U	R.VVLLGEELGHTMEGPTVENNYKPGIYVIPEIK.E
37984	266 – 297	1180.2857	3537.8353	3537.8429	-2.15	0	135	2.9e-14	1	U	R.VVLLGEELGHTMEGPTVENNYKPGIYVIPEIK.E
37985	266 – 297	885.4665	3537.8367	3537.8429	-1.74	0	66	2.7e-07	1	U	R.VVLLGEELGHTMEGPTVENNYKPGIYVIPEIK.E
37986	266 – 297	1180.2865	3537.8377	3537.8429	-1.47	0	112	6.5e-12	1	U	R.VVLLGEELGHTMEGPTVENNYKPGIYVIPEIK.E
37987	266 – 297	885.4669	3537.8386	3537.8429	-1.21	0	58	1.6e-06	1	U	R.VVLLGEELGHTMEGPTVENNYKPGIYVIPEIK.E
37988	266 – 297	885.4672	3537.8396	3537.8429	-0.93	0	65	3.2e-07	1	U	R.VVLLGEELGHTMEGPTVENNYKPGIYVIPEIK.E
37989	266 – 297	708.5755	3537.8410	3537.8429	-0.54	0	61	8.1e-07	1	U	R.VVLLGEELGHTMEGPTVENNYKPGIYVIPEIK.E
37990	266 – 297	885.4680	3537.8428	3537.8429	-0.029	0	71	7.2e-08	1	U	R.VVLLGEELGHTMEGPTVENNYKPGIYVIPEIK.E
1460	304 – 310	411.7213	821.4281	821.4283	-0.25	0	61	8.1e-07	1	U	R.QEVYVGK.S
30437	311 – 330	1081.1114	2160.2082	2160.2055	1.26	0	79	1.2e-08	1	U	K.SVFPALERPQAVVHLIVAQS.-

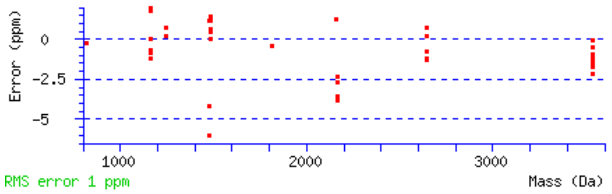

Mascot: http://www.matrixscience.com/

MASCOT Search Results

Protein View: TP84_12

Database: TP84
Score: 5774
Nominal mass (M_r): 37757
Calculated pI: 4.98

Sequence similarity is available as **an NCBI BLAST search of 67. against nr**.

Search parameters

MS data file: O:\FA\02-luty2018\80202246zeb_czas30.raw
Enzyme: Trypsin: cuts C-term side of KR unless next residue is P.
Fixed modifications: Carbamidomethyl (C)
Variable modifications: Oxidation (M)

Protein sequence coverage: 57%

Matched peptides shown in ***bold red***.

1 M**PLHLEQFQR** **EAFQGYVENV** **PPK**REYALAK FMPNQPVYDI EFTYNIINGG
51 YGQMASITAW DSGAPLRDKD VIQRLTAQIA KVQHAYRLTE KELLMFHRPR
101 **MDEEQQQVIQ** **AIYNNTDKLV** **WGVQDR**EEWL RAKAVYVGQL QYSENDVQLN
151 IDFLIPAENK **LTADVDWSDP** **TAPVIQHLQS** **AVQR**FK**EANN** **GEKPVEMHMS**
201 **SRVETWLLQN** **EQVKAHIYGN** **TTDPR**IVTSE QLQQLFSALS LPPYR**VIDEQ**
251 **VIGENGAEAL** **MPEDRVVLLG** **EELGHTMEGP** **TVENNYKPGI** **YVIPEIK**ETN
301 PPR**QEVYVGK** **SVFPALERPQ** **AVVHLIVAQS**

Unformatted sequence string: **330 residues** (for pasting into other applications).

Sort peptides by ◉ Residue Number ○ Increasing Mass ○ Decreasing Mass

Show predicted peptides also

Query	Start – End	Observed	Mr(expt)	Mr(calc)	ppm	M	Score	Expect	Rank	U	Peptide
12089	2 – 10	389.8793	1166.6162	1166.6196	-2.97	0	58	1.7e-06	1	U	M.PLHLEQFQR.E
12090	2 – 10	389.8795	1166.6168	1166.6196	-2.43	0	58	1.6e-06	1	U	M.PLHLEQFQR.E
12091	2 – 10	389.8797	1166.6173	1166.6196	-1.99	0	58	1.7e-06	1	U	M.PLHLEQFQR.E
12092	2 – 10	389.8798	1166.6176	1166.6196	-1.71	0	58	1.6e-06	1	U	M.PLHLEQFQR.E
12093	2 – 10	389.8799	1166.6178	1166.6196	-1.61	0	55	3e-06	1	U	M.PLHLEQFQR.E
12094	2 – 10	584.3163	1166.6181	1166.6196	-1.34	0	54	4.3e-06	1	U	M.PLHLEQFQR.E
12095	2 – 10	389.8800	1166.6181	1166.6196	-1.30	0	58	1.7e-06	1	U	M.PLHLEQFQR.E
12096	2 – 10	389.8801	1166.6184	1166.6196	-1.07	0	58	1.6e-06	1	U	M.PLHLEQFQR.E
12097	2 – 10	389.8801	1166.6184	1166.6196	-1.07	0	58	1.8e-06	1	U	M.PLHLEQFQR.E
12098	2 – 10	584.3165	1166.6185	1166.6196	-1.00	0	53	5.2e-06	1	U	M.PLHLEQFQR.E
12099	2 – 10	584.3171	1166.6197	1166.6196	0.051	0	61	7.5e-07	1	U	M.PLHLEQFQR.E
12100	2 – 10	584.3176	1166.6207	1166.6196	0.89	0	53	4.6e-06	1	U	M.PLHLEQFQR.E
23758	11 – 23	739.3701	1476.7256	1476.7249	0.50	0	87	2.2e-09	1	U	R.EAFQGYVENVPPK.R
23759	11 – 23	739.3732	1476.7318	1476.7249	4.70	0	54	3.6e-06	1	U	R.EAFQGYVENVPPK.R
23760	11 – 23	739.3734	1476.7323	1476.7249	5.00	0	57	2e-06	1	U	R.EAFQGYVENVPPK.R
39795	101 – 118	1083.9988	2165.9830	2165.9899	-3.16	0	132	6.9e-14	1	U	R.MDEEQQQVIQAIYNNTDK.L
39796	101 – 118	723.0017	2165.9831	2165.9899	-3.12	0	77	2e-08	1	U	R.MDEEQQQVIQAIYNNTDK.L
39797	101 – 118	1083.9996	2165.9846	2165.9899	-2.42	0	139	1.3e-14	1	U	R.MDEEQQQVIQAIYNNTDK.L
39798	101 – 118	1083.9997	2165.9848	2165.9899	-2.32	0	128	1.4e-13	1	U	R.MDEEQQQVIQAIYNNTDK.L
39799	101 – 118	723.0025	2165.9858	2165.9899	-1.89	0	73	4.6e-08	1	U	R.MDEEQQQVIQAIYNNTDK.L
39800	101 – 118	723.0030	2165.9871	2165.9899	-1.31	0	55	3.2e-06	1	U	R.MDEEQQQVIQAIYNNTDK.L
39802	101 – 118	723.0042	2165.9907	2165.9899	0.40	0	62	6.1e-07	1	U	R.MDEEQQQVIQAIYNNTDK.L
5436	119 – 126	486.7665	971.5185	971.5189	-0.38	0	50	9.5e-06	1	U	K.LVWGVQDR.E
5437	119 – 126	486.7667	971.5188	971.5189	-0.069	0	50	9.6e-06	1	U	K.LVWGVQDR.E
5438	119 – 126	486.7668	971.5190	971.5189	0.12	0	59	1.4e-06	1	U	K.LVWGVQDR.E
5439	119 – 126	486.7668	971.5190	971.5189	0.12	0	65	3e-07	1	U	K.LVWGVQDR.E
5442	119 – 126	486.7668	971.5191	971.5189	0.26	0	64	4.1e-07	1	U	K.LVWGVQDR.E
5443	119 – 126	486.7669	971.5193	971.5189	0.40	0	57	1.8e-06	1	U	K.LVWGVQDR.E
5444	119 – 126	486.7669	971.5193	971.5189	0.43	0	58	1.6e-06	1	U	K.LVWGVQDR.E
44348	161 – 184	883.1194	2646.3362	2646.3402	-1.50	0	119	1.3e-12	1	U	K.LTADVDWSDPTAPVIQHLQSAVQR.F
44349	161 – 184	883.1195	2646.3366	2646.3402	-1.38	0	132	5.8e-14	1	U	K.LTADVDWSDPTAPVIQHLQSAVQR.F
44350	161 – 184	883.1196	2646.3369	2646.3402	-1.23	0	119	1.2e-12	1	U	K.LTADVDWSDPTAPVIQHLQSAVQR.F
44351	161 – 184	883.1198	2646.3375	2646.3402	-1.03	0	155	3.3e-16	1	U	K.LTADVDWSDPTAPVIQHLQSAVQR.F
44352	161 – 184	883.1200	2646.3381	2646.3402	-0.79	0	157	2.1e-16	1	U	K.LTADVDWSDPTAPVIQHLQSAVQR.F
44353	161 – 184	883.1200	2646.3383	2646.3402	-0.73	0	145	3.1e-15	1	U	K.LTADVDWSDPTAPVIQHLQSAVQR.F
44354	161 – 184	883.1202	2646.3387	2646.3402	-0.57	0	140	1e-14	1	U	K.LTADVDWSDPTAPVIQHLQSAVQR.F
44355	161 – 184	883.1204	2646.3392	2646.3402	-0.37	0	137	2.1e-14	1	U	K.LTADVDWSDPTAPVIQHLQSAVQR.F
44356	161 – 184	883.1205	2646.3395	2646.3402	-0.25	0	136	2.3e-14	1	U	K.LTADVDWSDPTAPVIQHLQSAVQR.F
44357	161 – 184	883.1205	2646.3396	2646.3402	-0.23	0	136	2.3e-14	1	U	K.LTADVDWSDPTAPVIQHLQSAVQR.F
44358	161 – 184	883.1207	2646.3402	2646.3402	0.018	0	127	2.1e-13	1	U	K.LTADVDWSDPTAPVIQHLQSAVQR.F
33728	187 – 202	605.9417	1814.8033	1814.8039	-0.34	0	74	4.4e-08	1	U	K.EANNGEKPVEMHMSSR.V
33730	187 – 202	605.9424	1814.8053	1814.8039	0.76	0	70	1.1e-07	1	U	K.EANNGEKPVEMHMSSR.V
24064	203 – 214	743.8977	1485.7808	1485.7827	-1.27	0	82	5.8e-09	1	U	R.VETWLLQNEQVK.A
24065	203 – 214	743.8980	1485.7814	1485.7827	-0.89	0	76	2.5e-08	1	U	R.VETWLLQNEQVK.A
24066	203 – 214	743.8981	1485.7816	1485.7827	-0.74	0	85	3.4e-09	1	U	R.VETWLLQNEQVK.A

Query	Start – End	Observed	Mr(expt)	Mr(calc)	ppm	M	Score	Expect	Rank	U	Peptide
24067	203 – 214	743.8984	1485.7822	1485.7827	-0.34	0	82	6.2e-09	1	U	R.VETWLLQNEQVK.A
24068	203 – 214	743.8984	1485.7822	1485.7827	-0.32	0	83	5.2e-09	1	U	R.VETWLLQNEQVK.A
24069	203 – 214	743.8985	1485.7824	1485.7827	-0.22	0	73	5.6e-08	1	U	R.VETWLLQNEQVK.A
24070	203 – 214	743.8985	1485.7824	1485.7827	-0.19	0	53	4.8e-06	1	U	R.VETWLLQNEQVK.A
24071	203 – 214	743.8986	1485.7826	1485.7827	-0.096	0	77	1.9e-08	1	U	R.VETWLLQNEQVK.A
24072	203 – 214	743.8986	1485.7826	1485.7827	-0.055	0	84	3.8e-09	1	U	R.VETWLLQNEQVK.A
24073	203 – 214	743.8986	1485.7827	1485.7827	-0.042	0	50	9.2e-06	1	U	R.VETWLLQNEQVK.A
24074	203 – 214	743.8987	1485.7829	1485.7827	0.093	0	80	1.1e-08	1	U	R.VETWLLQNEQVK.A
24075	203 – 214	743.8987	1485.7829	1485.7827	0.13	0	71	8.2e-08	1	U	R.VETWLLQNEQVK.A
24076	203 – 214	743.8987	1485.7829	1485.7827	0.15	0	65	3.1e-07	1	U	R.VETWLLQNEQVK.A
24077	203 – 214	743.8989	1485.7833	1485.7827	0.36	0	77	2e-08	1	U	R.VETWLLQNEQVK.A
24078	203 – 214	743.8991	1485.7836	1485.7827	0.58	0	82	6.7e-09	1	U	R.VETWLLQNEQVK.A
24079	203 – 214	743.8991	1485.7836	1485.7827	0.60	0	64	4.1e-07	1	U	R.VETWLLQNEQVK.A
24080	203 – 214	743.8992	1485.7839	1485.7827	0.81	0	52	6.1e-06	1	U	R.VETWLLQNEQVK.A
24081	203 – 214	743.8994	1485.7842	1485.7827	0.97	0	59	1.4e-06	1	U	R.VETWLLQNEQVK.A
24082	203 – 214	743.8997	1485.7848	1485.7827	1.39	0	59	1.2e-06	1	U	R.VETWLLQNEQVK.A
24083	203 – 214	743.8999	1485.7852	1485.7827	1.65	0	76	2.5e-08	1	U	R.VETWLLQNEQVK.A
24084	203 – 214	743.9006	1485.7866	1485.7827	2.60	0	76	2.6e-08	1	U	R.VETWLLQNEQVK.A
15194	215 – 225	415.5381	1243.5925	1243.5945	-1.67	0	53	5.4e-06	1	U	K.AHIYGNTTDPR.I
15195	215 – 225	415.5383	1243.5930	1243.5945	-1.28	0	67	2.1e-07	1	U	K.AHIYGNTTDPR.I
15196	215 – 225	415.5384	1243.5933	1243.5945	-1.01	0	60	1.1e-06	1	U	K.AHIYGNTTDPR.I
15197	215 – 225	415.5385	1243.5937	1243.5945	-0.70	0	63	4.8e-07	1	U	K.AHIYGNTTDPR.I
15198	215 – 225	622.8041	1243.5937	1243.5945	-0.67	0	61	8.2e-07	1	U	K.AHIYGNTTDPR.I
15199	215 – 225	622.8043	1243.5940	1243.5945	-0.43	0	75	3.2e-08	1	U	K.AHIYGNTTDPR.I
15200	215 – 225	622.8044	1243.5943	1243.5945	-0.22	0	58	1.7e-06	1	U	K.AHIYGNTTDPR.I
15201	215 – 225	622.8047	1243.5949	1243.5945	0.31	0	75	3.3e-08	1	U	K.AHIYGNTTDPR.I
39967	246 – 265	729.0181	2184.0324	2184.0368	-2.01	0	93	4.6e-10	1	U	R.VIDEQVIGENGAEALMPEDR.V
48291	266 – 297	1180.2864	3537.8374	3537.8429	-1.56	0	121	7.1e-13	1	U	R.VVLLGEELGHTMEGPTVENNYKPGIYVIPEIK.E
48292	266 – 297	1180.2865	3537.8377	3537.8429	-1.47	0	106	2.3e-11	1	U	R.VVLLGEELGHTMEGPTVENNYKPGIYVIPEIK.E
48293	266 – 297	885.4669	3537.8383	3537.8429	-1.30	0	72	6.3e-08	1	U	R.VVLLGEELGHTMEGPTVENNYKPGIYVIPEIK.E
48294	266 – 297	1180.2876	3537.8410	3537.8429	-0.54	0	106	2.7e-11	1	U	R.VVLLGEELGHTMEGPTVENNYKPGIYVIPEIK.E
48295	266 – 297	885.4678	3537.8420	3537.8429	-0.24	0	55	2.9e-06	1	U	R.VVLLGEELGHTMEGPTVENNYKPGIYVIPEIK.E
48296	266 – 297	1180.2880	3537.8422	3537.8429	-0.20	0	106	2.7e-11	1	U	R.VVLLGEELGHTMEGPTVENNYKPGIYVIPEIK.E
48297	266 – 297	885.4679	3537.8425	3537.8429	-0.12	0	70	9.8e-08	1	U	R.VVLLGEELGHTMEGPTVENNYKPGIYVIPEIK.E
48298	266 – 297	1180.2881	3537.8425	3537.8429	-0.12	0	100	1.1e-10	1	U	R.VVLLGEELGHTMEGPTVENNYKPGIYVIPEIK.E
48299	266 – 297	1180.2882	3537.8428	3537.8429	-0.031	0	128	1.6e-13	1	U	R.VVLLGEELGHTMEGPTVENNYKPGIYVIPEIK.E
48300	266 – 297	708.5760	3537.8435	3537.8429	0.18	0	72	7e-08	1	U	R.VVLLGEELGHTMEGPTVENNYKPGIYVIPEIK.E
48304	266 – 297	885.4693	3537.8483	3537.8429	1.52	0	54	4e-06	1	U	R.VVLLGEELGHTMEGPTVENNYKPGIYVIPEIK.E
48305	266 – 297	708.5779	3537.8529	3537.8429	2.84	0	56	2.4e-06	1	U	R.VVLLGEELGHTMEGPTVENNYKPGIYVIPEIK.E
1704	304 – 310	411.7212	821.4279	821.4283	-0.49	0	61	7.5e-07	1	U	R.QEVYVGK.S
39731	311 – 330	1081.1081	2160.2016	2160.2055	-1.79	0	67	2e-07	1	U	K.SVFPALERPQAVVHLIVAQS.-

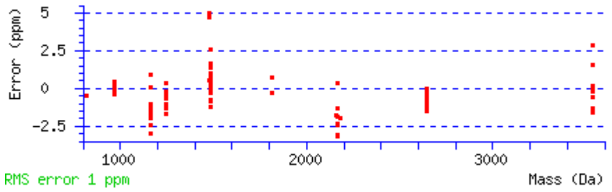

Mascot: http://www.matrixscience.com/

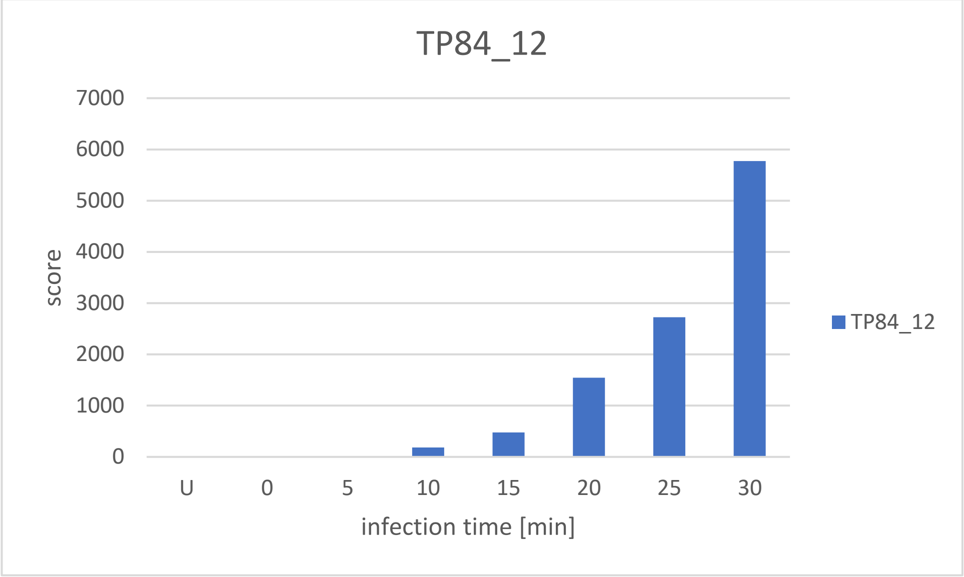

MASCOT Search Results

Protein View: TP84_13

Database: TP84
Score: 428
Nominal mass (M_r): 7214
Calculated pI: 9.19

Sequence similarity is available as **an NCBI BLAST search of 68. against nr**.

Search parameters

MS data file: O:\FA\02-luty2018\80202240zeb_czas0.raw
Enzyme: Trypsin: cuts C-term side of KR unless next residue is P.
Fixed modifications: Carbamidomethyl (C)
Variable modifications: Oxidation (M)

Protein sequence coverage: 39%

Matched peptides shown in ***bold red***.

```
 1 MPKYIAKRHL VTRTGIKKPG DVIEYTKEQA QKLLAAGFIE EAEEEKKTTS
51 KGKQKADEAD KVDK
```

Unformatted sequence string: **64 residues** (for pasting into other applications).

Sort peptides by ◉ Residue Number ○ Increasing Mass ○ Decreasing Mass

Show predicted peptides also

Query	Start – End	Observed	Mr(expt)	Mr(calc)	ppm	M	Score	Expect	Rank	U	Peptide
6701	18 – 27	575.3153	1148.6161	1148.6077	7.31	0	56	2.7e-06	1	U	K.KPGDVIEYTK.E
19286	33 – 47	559.6305	1675.8698	1675.8668	1.76	1	104	4.2e-11	1	U	K.LLAAGFIEEAEEEKK.T
19287	33 – 47	559.6308	1675.8707	1675.8668	2.32	1	105	3.1e-11	1	U	K.LLAAGFIEEAEEEKK.T
19288	33 – 47	559.6318	1675.8735	1675.8668	4.00	1	101	7.2e-11	1	U	K.LLAAGFIEEAEEEKK.T
19289	33 – 47	559.6323	1675.8751	1675.8668	4.97	1	114	4.2e-12	1	U	K.LLAAGFIEEAEEEKK.T

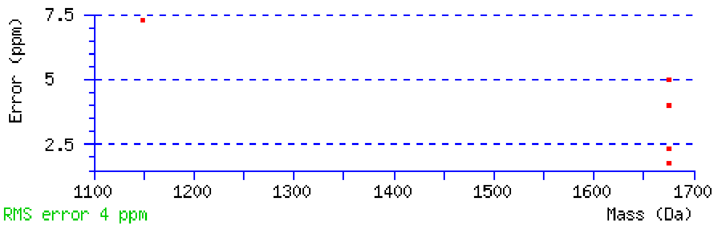

Mascot: http://www.matrixscience.com/

MASCOT Search Results

Protein View: TP84_13

**Database:**	TP84
**Score:**	467
**Nominal mass (M_r):**	7214
**Calculated pI:**	9.19

Sequence similarity is available as **an NCBI BLAST search of 68. against nr**.

Search parameters

**MS data file:**	O:\FA\02-luty2018\80202241zeb_czas5.raw
**Enzyme:**	Trypsin: cuts C-term side of KR unless next residue is P.
**Fixed modifications:**	Carbamidomethyl (C)
**Variable modifications:**	Oxidation (M)

Protein sequence coverage: 23%

Matched peptides shown in ***bold red***.

```
 1 MPKYIAKRHL VTRTGIKKPG DVIEYTKEQA QKLLAAGFIE EAEEEKKTTS
51 KGKQKADEAD KVDK
```

Unformatted sequence string: **64 residues** (for pasting into other applications).

Sort peptides by ◉ Residue Number ○ Increasing Mass ○ Decreasing Mass

Show predicted peptides also

Query	Start - End	Observed	Mr(expt)	Mr(calc)	ppm	M	Score	Expect	Rank	U	Peptide
19287	33 - 47	559.6299	1675.8679	1675.8668	0.65	1	106	2.4e-11	1	U	K.LLAAGFIEEAEEEKK.T
19288	33 - 47	559.6301	1675.8684	1675.8668	0.98	1	102	5.7e-11	1	U	K.LLAAGFIEEAEEEKK.T
19289	33 - 47	559.6304	1675.8694	1675.8668	1.53	1	110	1e-11	1	U	K.LLAAGFIEEAEEEKK.T
19290	33 - 47	559.6306	1675.8700	1675.8668	1.89	1	110	1.1e-11	1	U	K.LLAAGFIEEAEEEKK.T
19291	33 - 47	559.6311	1675.8714	1675.8668	2.73	1	91	8.4e-10	1	U	K.LLAAGFIEEAEEEKK.T

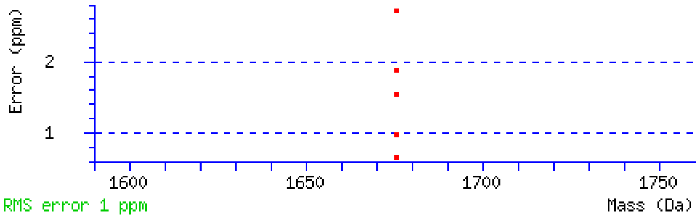

Mascot: http://www.matrixscience.com/

MASCOT Search Results

Protein View: TP84_13

**Database:**	TP84
**Score:**	182
**Nominal mass (M_r):**	7214
**Calculated pI:**	9.19

Sequence similarity is available as **an NCBI BLAST search of 68. against nr**.

Search parameters

**MS data file:**	O:\FA\02-luty2018\80202242zeb_czas10.raw
**Enzyme:**	Trypsin: cuts C-term side of KR unless next residue is P.
**Fixed modifications:**	Carbamidomethyl (C)
**Variable modifications:**	Oxidation (M)

Protein sequence coverage: 23%

Matched peptides shown in ***bold red***.

```
 1 MPKYIAKRHL VTRTGIKKPG DVIEYTKEQA QKLLAAGFIE EAEEEKKTTS
51 KGKQKADEAD KVDK
```

Unformatted sequence string: **64 residues** (for pasting into other applications).

Sort peptides by ◉ Residue Number ○ Increasing Mass ○ Decreasing Mass

Show predicted peptides also

Query	Start - End	Observed	Mr(expt)	Mr(calc)	ppm	M	Score	Expect	Rank	U	Peptide
18390	33 - 47	559.6297	1675.8673	1675.8668	0.28	1	86	2.8e-09	1	U	K.LLAAGFIEEAEEEKK.T
18391	33 - 47	559.6315	1675.8726	1675.8668	3.43	1	110	1e-11	1	U	K.LLAAGFIEEAEEEKK.T

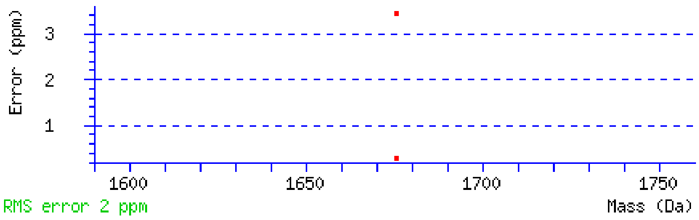

Mascot: http://www.matrixscience.com/

MASCOT Search Results

Protein View: TP84_13

Database: TP84
Score: 837
Nominal mass (M_r): 7214
Calculated pI: 9.19

Sequence similarity is available as **an NCBI BLAST search of 68. against nr**.

Search parameters

MS data file: O:\FA\02-luty2018\80202244zeb_czas20.raw
Enzyme: Trypsin: cuts C-term side of KR unless next residue is P.
Fixed modifications: Carbamidomethyl (C)
Variable modifications: Oxidation (M)

Protein sequence coverage: 39%

Matched peptides shown in ***bold red***.

```
 1 MPKYIAKRHL VTRTGIKKPG DVIEYTKEQA QKLLAAGFIE EAEEEKKTTS
51 KGKQKADEAD KVDK
```

Unformatted sequence string: **64 residues** (for pasting into other applications).

Sort peptides by ◉ Residue Number ○ Increasing Mass ○ Decreasing Mass

Show predicted peptides also

Query	Start – End	Observed	Mr(expt)	Mr(calc)	ppm	M	Score	Expect	Rank	U	Peptide
13175	18 – 27	575.3149	1148.6153	1148.6077	6.60	0	81	8.1e-09	1	U	K.KPGDVIEYTK.E
13177	18 – 27	575.3152	1148.6157	1148.6077	6.98	0	74	4.4e-08	1	U	K.KPGDVIEYTK.E
13178	18 – 27	575.3154	1148.6162	1148.6077	7.37	0	62	5.9e-07	1	U	K.KPGDVIEYTK.E
13179	18 – 27	575.3157	1148.6168	1148.6077	7.89	0	70	1e-07	1	U	K.KPGDVIEYTK.E
30807	33 – 47	559.6310	1675.8712	1675.8668	2.62	1	104	3.9e-11	1	U	K.LLAAGFIEEAEEEKK.T
30808	33 – 47	559.6311	1675.8715	1675.8668	2.82	1	82	6.4e-09	1	U	K.LLAAGFIEEAEEEKK.T
30809	33 – 47	559.6314	1675.8723	1675.8668	3.30	1	90	9.1e-10	1	U	K.LLAAGFIEEAEEEKK.T
30810	33 – 47	559.6314	1675.8724	1675.8668	3.36	1	100	9.1e-11	1	U	K.LLAAGFIEEAEEEKK.T
30811	33 – 47	559.6315	1675.8728	1675.8668	3.55	1	107	2.1e-11	1	U	K.LLAAGFIEEAEEEKK.T
30812	33 – 47	559.6317	1675.8731	1675.8668	3.77	1	87	1.9e-09	1	U	K.LLAAGFIEEAEEEKK.T
30813	33 – 47	559.6328	1675.8767	1675.8668	5.90	1	109	1.1e-11	1	U	K.LLAAGFIEEAEEEKK.T

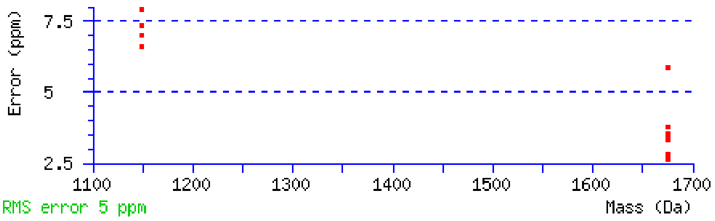

Mascot: http://www.matrixscience.com/

MASCOT Search Results

Protein View: TP84_13

Database: TP84
Score: 1553
Nominal mass (M_r): 7214
Calculated pI: 9.19

Sequence similarity is available as **an NCBI BLAST search of 68. against nr**.

Search parameters

MS data file: O:\FA\02-luty2018\80202245zeb_czas25.raw
Enzyme: Trypsin: cuts C-term side of KR unless next residue is P.
Fixed modifications: Carbamidomethyl (C)
Variable modifications: Oxidation (M)

Protein sequence coverage: 53%

Matched peptides shown in ***bold red***.

```
 1 MPKYIAKRHL VTRTGIKKPG DVIEYTKEQA QKLLAAGFIE EAEEEKKTTS
51 KGKQKADEAD KVDK
```

Unformatted sequence string: **64 residues** (for pasting into other applications).

Sort peptides by ◉ Residue Number ○ Increasing Mass ○ Decreasing Mass

Show predicted peptides also

Query	Start – End	Observed	Mr(expt)	Mr(calc)	ppm	M	Score	Expect	Rank	U	Peptide
8273	18 – 27	575.3114	1148.6082	1148.6077	0.45	0	87	2e-09	1	U	K.KPGDVIEYTK.E
8276	18 – 27	575.3122	1148.6099	1148.6077	1.93	0	97	2.1e-10	1	U	K.KPGDVIEYTK.E
18895	33 – 46	774.8919	1547.7693	1547.7719	-1.67	0	98	1.6e-10	1	U	K.LLAAGFIEEAEEEK.K
18896	33 – 46	774.8931	1547.7716	1547.7719	-0.13	0	116	2.6e-12	1	U	K.LLAAGFIEEAEEEK.K
18897	33 – 46	774.8935	1547.7724	1547.7719	0.36	0	102	6.5e-11	1	U	K.LLAAGFIEEAEEEK.K
21836	33 – 47	559.6291	1675.8656	1675.8668	-0.74	1	114	3.8e-12	1	U	K.LLAAGFIEEAEEEKK.T
21837	33 – 47	559.6292	1675.8659	1675.8668	-0.55	1	104	4.1e-11	1	U	K.LLAAGFIEEAEEEKK.T
21838	33 – 47	559.6293	1675.8660	1675.8668	-0.51	1	112	6.3e-12	1	U	K.LLAAGFIEEAEEEKK.T
21839	33 – 47	559.6296	1675.8669	1675.8668	0.063	1	99	1.4e-10	1	U	K.LLAAGFIEEAEEEKK.T
21840	33 – 47	559.6296	1675.8670	1675.8668	0.099	1	106	2.3e-11	1	U	K.LLAAGFIEEAEEEKK.T
21841	33 – 47	559.6296	1675.8671	1675.8668	0.19	1	103	4.8e-11	1	U	K.LLAAGFIEEAEEEKK.T
21842	33 – 47	559.6297	1675.8673	1675.8668	0.31	1	106	2.5e-11	1	U	K.LLAAGFIEEAEEEKK.T
21843	33 – 47	559.6297	1675.8674	1675.8668	0.37	1	115	3.5e-12	1	U	K.LLAAGFIEEAEEEKK.T
21844	33 – 47	559.6298	1675.8677	1675.8668	0.53	1	118	1.7e-12	1	U	K.LLAAGFIEEAEEEKK.T
21845	33 – 47	559.6300	1675.8683	1675.8668	0.87	1	110	1e-11	1	U	K.LLAAGFIEEAEEEKK.T
21846	33 – 47	559.6301	1675.8684	1675.8668	0.94	1	101	7.5e-11	1	U	K.LLAAGFIEEAEEEKK.T
4688	56 – 64	495.7409	989.4673	989.4665	0.82	1	74	3.6e-08	1	U	K.ADEADKVDK.-

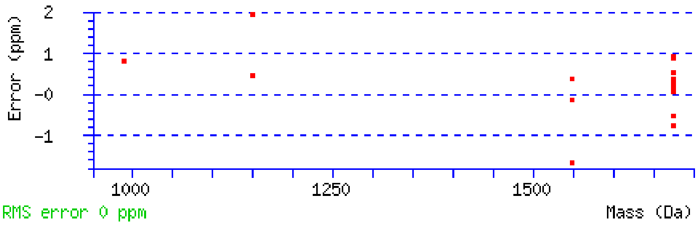

Mascot: http://www.matrixscience.com/

MASCOT Search Results

Protein View: TP84_13

**Database:**	TP84
**Score:**	2311
**Nominal mass (M_r):**	7214
**Calculated pI:**	9.19

Sequence similarity is available as **an NCBI BLAST search of 68. against nr**.

Search parameters

**MS data file:**	O:\FA\02-luty2018\80202246zeb_czas30.raw
**Enzyme:**	Trypsin: cuts C-term side of KR unless next residue is P.
**Fixed modifications:**	Carbamidomethyl (C)
**Variable modifications:**	Oxidation (M)

Protein sequence coverage: 39%

Matched peptides shown in ***bold red***.

```
 1 MPKYIAKRHL VTRTGIKKPG DVIEYTKEQA QKLLAAGFIE EAEEEKKTTS
51 KGKQKADEAD KVDK
```

Unformatted sequence string: **64 residues** (for pasting into other applications).

Sort peptides by ◉ Residue Number ○ Increasing Mass ○ Decreasing Mass

Show predicted peptides also

Query	Start - End	Observed	Mr(expt)	Mr(calc)	ppm	M	Score	Expect	Rank	U	Peptide
11411	18 - 27	383.8762	1148.6069	1148.6077	-0.73	0	53	4.5e-06	1	U	K.KPGDVIEYTK.E
11415	18 - 27	575.3114	1148.6083	1148.6077	0.47	0	97	2.2e-10	1	U	K.KPGDVIEYTK.E
11416	18 - 27	575.3115	1148.6084	1148.6077	0.63	0	82	6.4e-09	1	U	K.KPGDVIEYTK.E
11417	18 - 27	575.3116	1148.6087	1148.6077	0.84	0	82	6.6e-09	1	U	K.KPGDVIEYTK.E
11418	18 - 27	575.3118	1148.6090	1148.6077	1.08	0	92	7.1e-10	1	U	K.KPGDVIEYTK.E
11419	18 - 27	575.3118	1148.6091	1148.6077	1.22	0	86	2.7e-09	1	U	K.KPGDVIEYTK.E
11420	18 - 27	575.3119	1148.6091	1148.6077	1.24	0	92	7e-10	1	U	K.KPGDVIEYTK.E
11421	18 - 27	575.3119	1148.6093	1148.6077	1.34	0	86	2.6e-09	1	U	K.KPGDVIEYTK.E
11422	18 - 27	575.3121	1148.6097	1148.6077	1.71	0	97	2.1e-10	1	U	K.KPGDVIEYTK.E
26024	33 - 46	774.8878	1547.7610	1547.7719	-6.98	0	55	3.5e-06	1	U	K.LLAAGFIEEAEEEK.K
26025	33 - 46	774.8909	1547.7673	1547.7719	-2.94	0	87	2e-09	1	U	K.LLAAGFIEEAEEEK.K
26026	33 - 46	774.8924	1547.7702	1547.7719	-1.07	0	100	9.5e-11	1	U	K.LLAAGFIEEAEEEK.K
26027	33 - 46	774.8926	1547.7706	1547.7719	-0.82	0	99	1.4e-10	1	U	K.LLAAGFIEEAEEEK.K
26028	33 - 46	774.8931	1547.7716	1547.7719	-0.18	0	119	1.3e-12	1	U	K.LLAAGFIEEAEEEK.K
26029	33 - 46	774.8933	1547.7720	1547.7719	0.11	0	108	1.5e-11	1	U	K.LLAAGFIEEAEEEK.K
26030	33 - 46	774.8939	1547.7732	1547.7719	0.85	0	116	2.3e-12	1	U	K.LLAAGFIEEAEEEK.K
26031	33 - 46	774.8939	1547.7733	1547.7719	0.94	0	118	1.5e-12	1	U	K.LLAAGFIEEAEEEK.K
29702	33 - 47	559.6292	1675.8657	1675.8668	-0.65	1	94	4.4e-10	1	U	K.LLAAGFIEEAEEEKK.T
29703	33 - 47	559.6294	1675.8663	1675.8668	-0.29	1	106	2.5e-11	1	U	K.LLAAGFIEEAEEEKK.T
29704	33 - 47	559.6296	1675.8669	1675.8668	0.027	1	101	7.6e-11	1	U	K.LLAAGFIEEAEEEKK.T
29705	33 - 47	559.6296	1675.8669	1675.8668	0.063	1	91	7.3e-10	1	U	K.LLAAGFIEEAEEEKK.T
29706	33 - 47	559.6297	1675.8674	1675.8668	0.37	1	99	1.4e-10	1	U	K.LLAAGFIEEAEEEKK.T
29708	33 - 47	559.6298	1675.8677	1675.8668	0.51	1	103	4.9e-11	1	U	K.LLAAGFIEEAEEEKK.T
29709	33 - 47	559.6300	1675.8681	1675.8668	0.80	1	106	2.4e-11	1	U	K.LLAAGFIEEAEEEKK.T
29710	33 - 47	559.6301	1675.8684	1675.8668	0.96	1	94	3.8e-10	1	U	K.LLAAGFIEEAEEEKK.T
29711	33 - 47	559.6307	1675.8702	1675.8668	2.05	1	103	4.9e-11	1	U	K.LLAAGFIEEAEEEKK.T
29712	33 - 47	559.6307	1675.8703	1675.8668	2.10	1	101	7.3e-11	1	U	K.LLAAGFIEEAEEEKK.T
29713	33 - 47	559.6309	1675.8708	1675.8668	2.37	1	96	2.3e-10	1	U	K.LLAAGFIEEAEEEKK.T

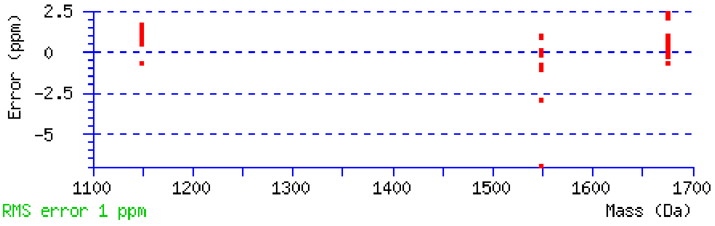

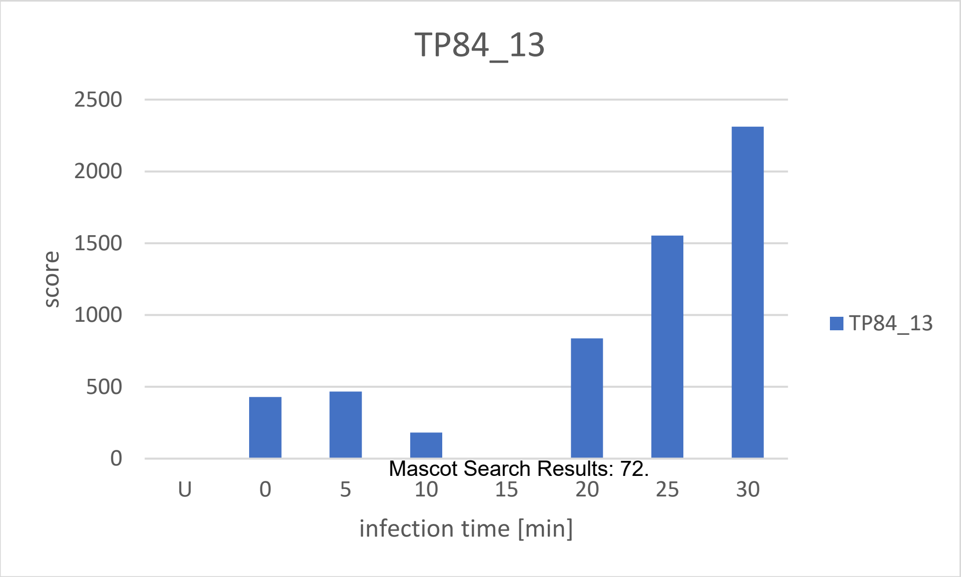

12.02.2018

MASCOT Search Results

Protein View: TP84_17

**Database:**	TP84
**Score:**	246
**Nominal mass (M_r):**	14639
**Calculated pI:**	5.35

Sequence similarity is available as **an NCBI BLAST search of 72. against nr**.

Search parameters

**MS data file:**	O:\FA\02-luty2018\80202245zeb_czas25.raw
**Enzyme:**	Trypsin: cuts C-term side of KR unless next residue is P.
**Fixed modifications:**	**Carbamidomethyl (C)**
**Variable modifications:**	**Oxidation (M)**

Protein sequence coverage: 20%

Matched peptides shown in ***bold red***.

```
  1 MIQKWIKDQL EAAIPNLEWT YDYKTGKDHT GVVYHETPGQ ISRDDFEIIT
 51 PSYSVYIETS DMKNAEKWAW IVYDTMNKRR QEVATIDDRS FQVIFIEVTT
101 PPILVGIVDK KMTYSINLQA TIRKI
```

Unformatted sequence string: **125 residues** (for pasting into other applications).

Sort peptides by ◉ Residue Number ○ Increasing Mass ○ Decreasing Mass

Show predicted peptides also

Query	Start - End	Observed	Mr(expt)	Mr(calc)	ppm	M	Score	Expect	Rank	U	Peptide
29123	8 - 24	1034.9977	2067.9808	2067.9789	0.94	0	108	1.5e-11	1	U	K.DQLEAAIPNLEWTYDYK.T
29124	8 - 24	1034.9982	2067.9818	2067.9789	1.43	0	88	1.7e-09	1	U	K.DQLEAAIPNLEWTYDYK.T
5934	81 - 89	523.7593	1045.5041	1045.5040	0.12	0	76	2.7e-08	1	U	R.QEVATIDDR.S

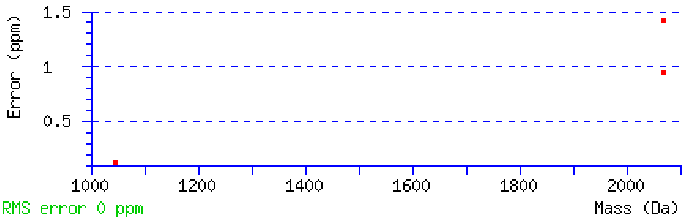

Mascot: **http://www.matrixscience.com/**

MASCOT Search Results

Protein View: TP84_17

**Database:**	TP84
**Score:**	451
**Nominal mass (M_r):**	14639
**Calculated pI:**	5.35

Sequence similarity is available as **an NCBI BLAST search of 72. against nr**.

Search parameters

**MS data file:**	O:\FA\02-luty2018\80202246zeb_czas30.raw
**Enzyme:**	Trypsin: cuts C-term side of KR unless next residue is P.
**Fixed modifications:**	**Carbamidomethyl (C)**
**Variable modifications:**	**Oxidation (M)**

Protein sequence coverage: 20%

Matched peptides shown in ***bold red***.

```
  1 MIQKWIKDQL EAAIPNLEWT YDYKTGKDHT GVVYHETPGQ ISRDDFEIIT
 51 PSYSVYIETS DMKNAEKWAW IVYDTMNKRR QEVATIDDRS FQVIFIEVTT
101 PPILVGIVDK KMTYSINLQA TIRKI
```

Unformatted sequence string: **125 residues** (for pasting into other applications).

Sort peptides by ◉ Residue Number ○ Increasing Mass ○ Decreasing Mass

Show predicted peptides also

Query	Start - End	Observed	Mr(expt)	Mr(calc)	ppm	M	Score	Expect	Rank	U	Peptide
33001	28 - 43	898.4375	1794.8605	1794.8649	-2.45	0	74	3.6e-08	1	U	K.DHTGVVYHETPGQISR.D
33002	28 - 43	898.4394	1794.8642	1794.8649	-0.40	0	120	1.1e-12	1	U	K.DHTGVVYHETPGQISR.D
33003	28 - 43	599.2956	1794.8650	1794.8649	0.024	0	80	9.1e-09	1	U	K.DHTGVVYHETPGQISR.D
33004	28 - 43	599.2960	1794.8663	1794.8649	0.76	0	53	4.5e-06	1	U	K.DHTGVVYHETPGQISR.D
33005	28 - 43	599.2961	1794.8664	1794.8649	0.81	0	71	7.7e-08	1	U	K.DHTGVVYHETPGQISR.D
7925	81 - 89	523.7592	1045.5038	1045.5040	-0.14	0	66	2.5e-07	1	U	R.QEVATIDDR.S
7926	81 - 89	523.7595	1045.5045	1045.5040	0.47	0	64	3.8e-07	1	U	R.QEVATIDDR.S

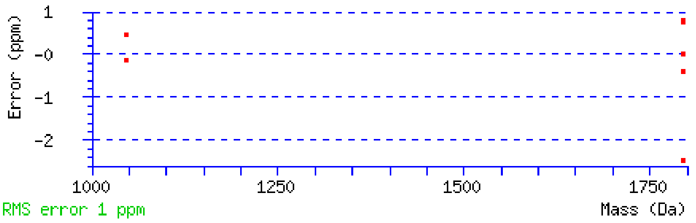

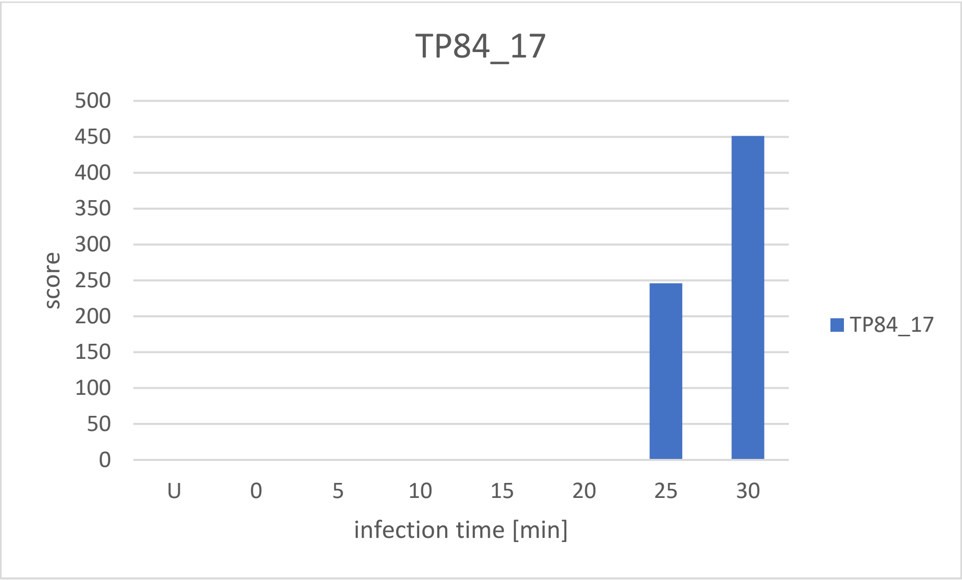

9.02.2018

MASCOT Search Results

Protein View: TP84_18

**Database:**	TP84
**Score:**	743
**Nominal mass (M_r):**	20371
**Calculated pI:**	4.38

Sequence similarity is available as **an NCBI BLAST search of 73. against nr**.

Search parameters

**MS data file:**	O:\FA\02-luty2018\80202245zeb_czas25.raw
**Enzyme:**	Trypsin: cuts C-term side of KR unless next residue is P.
**Fixed modifications:**	**Carbamidomethyl (C)**
**Variable modifications:**	**Oxidation (M)**

Protein sequence coverage: 49%

Matched peptides shown in ***bold red***.

```
  1 MPGVNIPFGL ATITVGDPQD PNKIVFDGVN YFQAEGGELN IEPELEPIEL
 51 QDFGATPYDE RINGYTCELT IVVGQNDLKL MRKLFAYHSE IVDEGTGEVI
101 GLTDEKIGAS MRDKAVPVTI HPREMGSDTS LDIHIYKMSG VGAFNRTYEN
151 AQGSYEVTLR AYPRDGADPT KPGNFYYIGD TDPNA
```

Unformatted sequence string: **185 residues** (for pasting into other applications).

Sort peptides by ◉ Residue Number ○ Increasing Mass ○ Decreasing Mass

Show predicted peptides also

Query	Start - End	Observed	Mr(expt)	Mr(calc)	ppm	M	Score	Expect	Rank	U	Peptide
32901	1 - 23	794.4100	2380.2083	2380.2097	-0.59	0	71	7.9e-08	1	U	-.MPGVNIPFGLATITVGDPQDPNK.I
32902	1 - 23	794.4109	2380.2108	2380.2097	0.45	0	77	2.1e-08	1	U	-.MPGVNIPFGLATITVGDPQDPNK.I
32903	1 - 23	794.4118	2380.2137	2380.2097	1.66	0	58	1.5e-06	1	U	-.MPGVNIPFGLATITVGDPQDPNK.I
34506	83 - 106	884.1130	2649.3171	2649.3174	-0.11	1	137	2.2e-14	1	U	R.KLFAYHSEIVDEGTGEVIGLTDEK.I
33808	84 - 106	841.4135	2521.2186	2521.2224	-1.50	0	117	2.1e-12	1	U	K.LFAYHSEIVDEGTGEVIGLTDEK.I
4687	115 - 123	495.3005	988.5865	988.5818	4.76	0	55	3.3e-06	1	U	K.AVPVTIHPR.E
20692	147 - 160	815.8891	1629.7636	1629.7634	0.10	0	104	4.2e-11	1	U	R.TYENAQGSYEVTLR.A
31193	165 - 185	1114.4889	2226.9632	2226.9706	-3.29	0	117	1.9e-12	1	U	R.DGADPTKPGNFYYIGDTDPNA.-
31195	165 - 185	1114.4898	2226.9650	2226.9706	-2.48	0	111	7.3e-12	1	U	R.DGADPTKPGNFYYIGDTDPNA.-

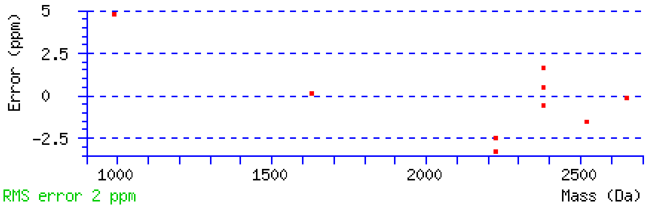

MASCOT Search Results

Protein View: TP84_18

**Database:**	TP84
**Score:**	648
**Nominal mass (M_r):**	20371
**Calculated pI:**	4.38

Sequence similarity is available as an NCBI BLAST search of 73. against nr.

Search parameters

**MS data file:**	O:\FA\02-luty2018\80202246zeb_czas30.raw
**Enzyme:**	Trypsin: cuts C-term side of KR unless next residue is P.
**Fixed modifications:**	Carbamidomethyl (C)
**Variable modifications:**	Oxidation (M)

Protein sequence coverage: 34%

Matched peptides shown in ***bold red***.

```
  1 MPGVNIPFGL ATITVGDPQD PNKIVFDGVN YFQAEGGELN IEPELEPIEL
 51 QDFGATPYDE RINGYTCELT IVVGQNDLKL MRKLFAYHSE IVDEGTGEVI
101 GLTDEKIGAS MRDKAVPVTI HPREMGSDTS LDIHIYKMSG VGAFNRTYEN
151 AQGSYEVTLR AYPRDGADPT KPGNFYYIGD TDPNA
```

Unformatted sequence string: 185 residues (for pasting into other applications).

Sort peptides by ◉ Residue Number ○ Increasing Mass ○ Decreasing Mass

Show predicted peptides also

Query	Start – End	Observed	Mr(expt)	Mr(calc)	ppm	M	Score	Expect	Rank	U	Peptide
44378	83 – 106	884.1135	2649.3186	2649.3174	0.46	1	121	8.1e-13	1	U	R.KLFAYHSEIVDEGTGEVIGLTDEK.I
6013	115 – 123	330.5344	988.5815	988.5818	-0.34	0	52	6.2e-06	1	U	K.AVPVTIHPR.E
6014	115 – 123	495.2982	988.5819	988.5818	0.067	0	67	1.9e-07	1	U	K.AVPVTIHPR.E
6015	115 – 123	495.2988	988.5831	988.5818	1.30	0	59	1.2e-06	1	U	K.AVPVTIHPR.E
4306	138 – 146	469.7285	937.4425	937.4440	-1.62	0	60	9.6e-07	1	U	K.MSGVGAFNR.T
4307	138 – 146	469.7286	937.4427	937.4440	-1.36	0	58	1.7e-06	1	U	K.MSGVGAFNR.T
4309	138 – 146	469.7288	937.4430	937.4440	-1.00	0	57	1.9e-06	1	U	K.MSGVGAFNR.T
4310	138 – 146	469.7292	937.4438	937.4440	-0.21	0	53	5.5e-06	1	U	K.MSGVGAFNR.T
4311	138 – 146	469.7292	937.4438	937.4440	-0.19	0	53	5.5e-06	1	U	K.MSGVGAFNR.T
40590	165 – 185	1114.4877	2226.9608	2226.9706	-4.36	0	101	7.1e-11	1	U	R.DGADPTKPGNFYYIGDTDPNA.-
40591	165 – 185	1114.4933	2226.9720	2226.9706	0.67	0	96	2.3e-10	1	U	R.DGADPTKPGNFYYIGDTDPNA.-

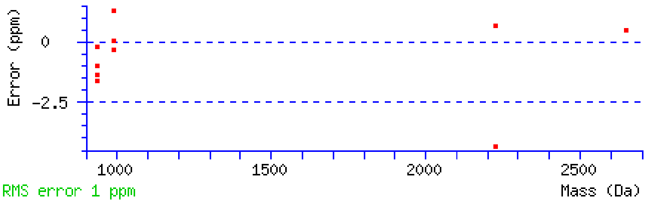

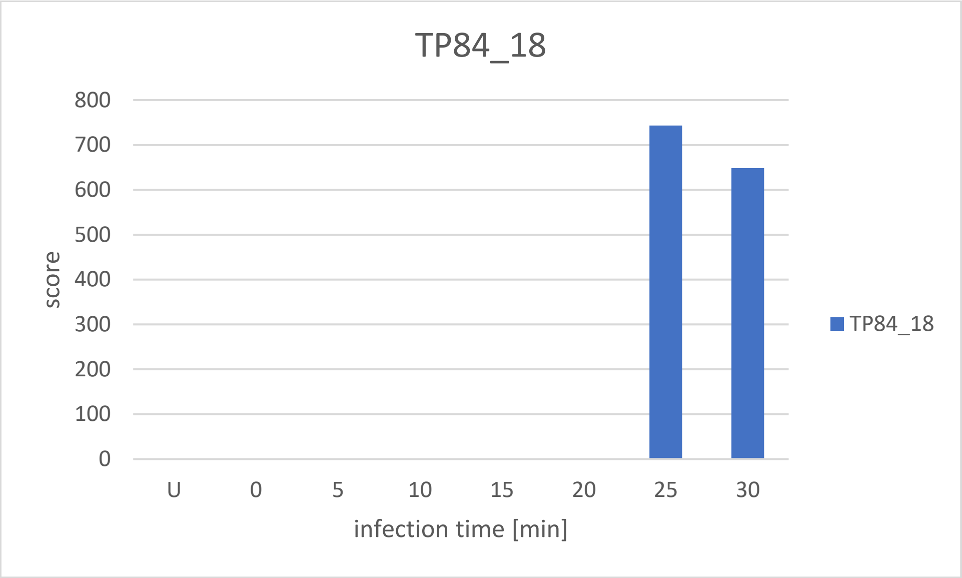

MASCOT Search Results

Protein View: TP84_19

Database: TP84
Score: 1463
Nominal mass (M_r): 19335
Calculated pI: 4.90

Sequence similarity is available as an NCBI BLAST search of 74. against nr.

Search parameters

MS data file: O:\FA\02-luty2018\80202244zeb_czas20.raw
Enzyme: Trypsin: cuts C-term side of KR unless next residue is P.
Fixed modifications: Carbamidomethyl (C)
Variable modifications: Oxidation (M)

Protein sequence coverage: 30%

Matched peptides shown in ***bold red***.

```
  1 MAKVTLTIKN GNVKESQQFE IDKITTFQAL KLKNEIHAIL KDLKNNGELK
 51 EVMEGLFSGE FDVDNMDIKN ITADQLEQMK DEKFITSLAG AFDRLLETVP
101 ERAMNLLSIM SGIDREVLEK AYLEELFDVY DAVMEENDII KLIDRMKRSF
151 FTTKGQWSQA LRTFLANK
```

Unformatted sequence string: 168 residues (for pasting into other applications).

Sort peptides by ◉ Residue Number ○ Increasing Mass ○ Decreasing Mass

Show predicted peptides also

Query	Start – End	Observed	Mr(expt)	Mr(calc)	ppm	M	Score	Expect	Rank	U	Peptide
4826	24 – 31	461.2764	920.5382	920.5331	5.49	0	57	2.1e-06	1	U	K.ITTFQALK.L
4827	24 – 31	461.2767	920.5388	920.5331	6.21	0	62	6.6e-07	1	U	K.ITTFQALK.L
14323	32 – 41	393.5820	1177.7241	1177.7182	4.96	1	63	5.6e-07	1	U	K.LKNEIHAILK.D
14324	32 – 41	393.5821	1177.7244	1177.7182	5.26	1	60	9.7e-07	1	U	K.LKNEIHAILK.D
14325	32 – 41	589.8707	1177.7269	1177.7182	7.32	1	82	6.7e-09	1	U	K.LKNEIHAILK.D
14326	32 – 41	589.8708	1177.7270	1177.7182	7.47	1	80	9.9e-09	1	U	K.LKNEIHAILK.D
30287	70 – 83	831.9073	1661.8000	1661.7930	4.20	1	126	2.5e-13	1	U	K.NITADQLEQMKDEK.F
30288	70 – 83	554.9407	1661.8002	1661.7930	4.33	1	71	8.7e-08	1	U	K.NITADQLEQMKDEK.F
30289	70 – 83	554.9409	1661.8009	1661.7930	4.76	1	63	4.8e-07	1	U	K.NITADQLEQMKDEK.F
30290	70 – 83	831.9080	1661.8014	1661.7930	5.04	1	120	9.4e-13	1	U	K.NITADQLEQMKDEK.F
30291	70 – 83	831.9080	1661.8015	1661.7930	5.10	1	120	9.1e-13	1	U	K.NITADQLEQMKDEK.F
30292	70 – 83	554.9413	1661.8020	1661.7930	5.41	1	75	3e-08	1	U	K.NITADQLEQMKDEK.F
30293	70 – 83	554.9414	1661.8023	1661.7930	5.58	1	68	1.6e-07	1	U	K.NITADQLEQMKDEK.F
30294	70 – 83	554.9415	1661.8026	1661.7930	5.79	1	65	2.9e-07	1	U	K.NITADQLEQMKDEK.F
30295	70 – 83	554.9419	1661.8037	1661.7930	6.44	1	70	9.7e-08	1	U	K.NITADQLEQMKDEK.F
15079	84 – 94	599.3177	1196.6208	1196.6190	1.55	0	72	5.9e-08	1	U	K.FITSLAGAFDR.L
6049	95 – 102	478.7765	955.5384	955.5338	4.81	0	56	2.4e-06	1	U	R.LLETVPER.A
6050	95 – 102	478.7765	955.5385	955.5338	4.88	0	56	2.2e-06	1	U	R.LLETVPER.A
6051	95 – 102	478.7766	955.5386	955.5338	4.98	0	59	1.1e-06	1	U	R.LLETVPER.A
6052	95 – 102	478.7766	955.5386	955.5338	5.02	0	63	4.7e-07	1	U	R.LLETVPER.A
6053	95 – 102	478.7767	955.5388	955.5338	5.23	0	56	2.5e-06	1	U	R.LLETVPER.A
6055	95 – 102	478.7768	955.5391	955.5338	5.52	0	51	8.1e-06	1	U	R.LLETVPER.A
6056	95 – 102	478.7770	955.5395	955.5338	5.90	0	60	1.1e-06	1	U	R.LLETVPER.A
6058	95 – 102	478.7774	955.5403	955.5338	6.74	0	60	1.1e-06	1	U	R.LLETVPER.A
6059	95 – 102	478.7776	955.5406	955.5338	7.05	0	60	1.1e-06	1	U	R.LLETVPER.A

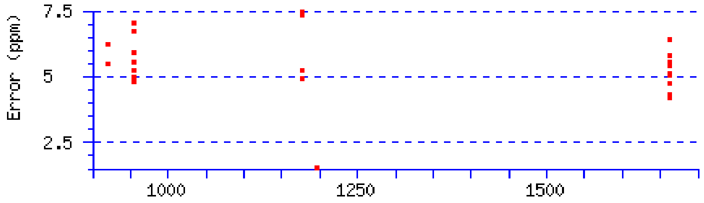

RMS error 5 ppm

MASCOT Search Results

Protein View: TP84_19

Database: TP84
Score: 2591
Nominal mass (M_r): 19335
Calculated pI: 4.90

Sequence similarity is available as an NCBI BLAST search of 74. against nr.

Search parameters

MS data file: O:\FA\02-luty2018\80202245zeb_czas25.raw
Enzyme: Trypsin: cuts C-term side of KR unless next residue is P.
Fixed modifications: Carbamidomethyl (C)
Variable modifications: Oxidation (M)

Protein sequence coverage: 60%

Matched peptides shown in ***bold red***.

```
  1 MAKVTLTIKN GNVKESQQFE IDKITTFQAL KLKNEIHAIL KDLKNNGELK
 51 EVMEGLFSGE FDVDNMDIKN ITADQLEQMK DEKFITSLAG AFDRLLETVP
101 ERAMNLLSIM SGIDREVLEK AYLEELFDVY DAVMEENDII KLIDRMKRSF
151 FTTKGQWSQA LRTFLANK
```

Unformatted sequence string: 168 residues (for pasting into other applications).

Sort peptides by ◉ Residue Number ○ Increasing Mass ○ Decreasing Mass

Show predicted peptides also

Query	Start – End	Observed	Mr(expt)	Mr(calc)	ppm	M	Score	Expect	Rank	U	Peptide
7571	15 – 23	562.2666	1122.5186	1122.5193	-0.64	0	57	1.8e-06	1	U	K.ESQQFEIDK.I
7572	15 – 23	562.2666	1122.5186	1122.5193	-0.58	0	53	4.6e-06	1	U	K.ESQQFEIDK.I
7573	15 – 23	562.2667	1122.5189	1122.5193	-0.39	0	57	1.9e-06	1	U	K.ESQQFEIDK.I
7574	15 – 23	562.2668	1122.5190	1122.5193	-0.26	0	62	6.8e-07	1	U	K.ESQQFEIDK.I
7575	15 – 23	562.2672	1122.5199	1122.5193	0.54	0	73	5.2e-08	1	U	K.ESQQFEIDK.I
7576	15 – 23	562.2674	1122.5203	1122.5193	0.88	0	59	1.2e-06	1	U	K.ESQQFEIDK.I
7577	15 – 23	562.2678	1122.5211	1122.5193	1.63	0	67	2.2e-07	1	U	K.ESQQFEIDK.I
3031	24 – 31	461.2733	920.5321	920.5331	-1.13	0	62	6.4e-07	1	U	K.ITTFQALK.L
9019	32 – 41	393.5794	1177.7163	1177.7182	-1.69	1	79	1.3e-08	1	U	K.LKNEIHAILK.D
9020	32 – 41	393.5795	1177.7166	1177.7182	-1.39	1	71	8.6e-08	1	U	K.LKNEIHAILK.D
9021	32 – 41	393.5795	1177.7168	1177.7182	-1.21	1	53	5.4e-06	1	U	K.LKNEIHAILK.D
9022	32 – 41	393.5796	1177.7170	1177.7182	-1.06	1	63	5.5e-07	1	U	K.LKNEIHAILK.D
9023	32 – 41	393.5797	1177.7171	1177.7182	-0.95	1	71	8.7e-08	1	U	K.LKNEIHAILK.D
9024	32 – 41	393.5797	1177.7171	1177.7182	-0.95	1	62	5.6e-07	1	U	K.LKNEIHAILK.D
9025	32 – 41	393.5797	1177.7173	1177.7182	-0.83	1	65	3.5e-07	1	U	K.LKNEIHAILK.D
9027	32 – 41	393.5800	1177.7182	1177.7182	-0.063	1	68	1.7e-07	1	U	K.LKNEIHAILK.D
9028	32 – 41	393.5800	1177.7182	1177.7182	-0.012	1	65	2.9e-07	1	U	K.LKNEIHAILK.D
9029	32 – 41	589.8667	1177.7188	1177.7182	0.48	1	91	8.2e-10	1	U	K.LKNEIHAILK.D
9031	32 – 41	589.8670	1177.7194	1177.7182	0.97	1	101	7.6e-11	1	U	K.LKNEIHAILK.D
9032	32 – 41	589.8670	1177.7195	1177.7182	1.04	1	80	9.4e-09	1	U	K.LKNEIHAILK.D
9033	32 – 41	589.8670	1177.7195	1177.7182	1.11	1	98	1.6e-10	1	U	K.LKNEIHAILK.D
3352	34 – 41	469.2769	936.5393	936.5392	0.083	0	59	1.3e-06	1	U	K.NEIHAILK.D
21344	70 – 83	831.9036	1661.7926	1661.7930	-0.23	1	91	8.8e-10	1	U	K.NITADQLEQMKDEK.F
21345	70 – 83	554.9386	1661.7941	1661.7930	0.63	1	73	5.1e-08	1	U	K.NITADQLEQMKDEK.F
21346	70 – 83	554.9388	1661.7945	1661.7930	0.90	1	61	7.8e-07	1	U	K.NITADQLEQMKDEK.F
21347	70 – 83	554.9389	1661.7948	1661.7930	1.05	1	72	6e-08	1	U	K.NITADQLEQMKDEK.F
21348	70 – 83	554.9394	1661.7963	1661.7930	1.98	1	69	1.2e-07	1	U	K.NITADQLEQMKDEK.F
9549	84 – 94	599.3163	1196.6180	1196.6190	-0.79	0	70	9.4e-08	1	U	K.FITSLAGAFDR.L
9550	84 – 94	599.3166	1196.6186	1196.6190	-0.34	0	73	4.9e-08	1	U	K.FITSLAGAFDR.L
9551	84 – 94	599.3166	1196.6187	1196.6190	-0.25	0	72	5.7e-08	1	U	K.FITSLAGAFDR.L
9552	84 – 94	599.3167	1196.6189	1196.6190	-0.054	0	67	2.1e-07	1	U	K.FITSLAGAFDR.L
9553	84 – 94	599.3168	1196.6190	1196.6190	0.013	0	71	7.2e-08	1	U	K.FITSLAGAFDR.L
9555	84 – 94	599.3168	1196.6190	1196.6190	0.029	0	76	2.4e-08	1	U	K.FITSLAGAFDR.L
9556	84 – 94	599.3168	1196.6191	1196.6190	0.11	0	72	6.7e-08	1	U	K.FITSLAGAFDR.L
9557	84 – 94	599.3170	1196.6193	1196.6190	0.31	0	83	5.6e-09	1	U	K.FITSLAGAFDR.L
3857	95 – 102	478.7741	955.5336	955.5338	-0.19	0	64	4.3e-07	1	U	R.LLETVPER.A
3858	95 – 102	478.7745	955.5344	955.5338	0.59	0	63	4.7e-07	1	U	R.LLETVPER.A
3859	95 – 102	478.7745	955.5345	955.5338	0.75	0	61	8.4e-07	1	U	R.LLETVPER.A
3860	95 – 102	478.7746	955.5346	955.5338	0.82	0	60	1.1e-06	1	U	R.LLETVPER.A
15477	103 – 115	710.8679	1419.7213	1419.7214	-0.019	0	103	4.8e-11	1	U	R.AMNLLSIMSGIDR.E
15478	103 – 115	710.8702	1419.7259	1419.7214	3.21	0	86	2.8e-09	1	U	R.AMNLLSIMSGIDR.E

Query	Start - End	Observed	Mr(expt)	Mr(calc)	ppm	M	Score	Expect	Rank	U	Peptide
33793	121 - 141	840.4017	2518.1831	2518.1825	0.25	0	75	3.3e-08	1	U	K.AYLEELFDVYDAVMEENDIIK.L
33794	121 - 141	1260.0995	2518.1844	2518.1825	0.78	0	120	9.6e-13	1	U	K.AYLEELFDVYDAVMEENDIIK.L
3573	155 - 162	473.2491	944.4836	944.4828	0.83	0	54	4.4e-06	1	U	K.GQWSQALR.T

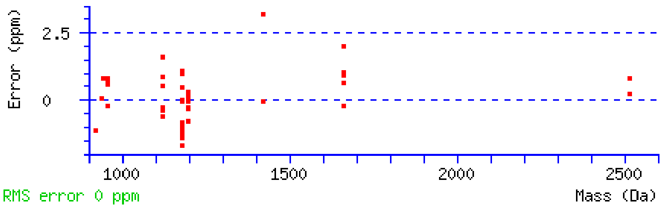

Mascot: http://www.matrixscience.com/

MASCOT Search Results

Protein View: TP84_19

**Database:**	TP84
**Score:**	5197
**Nominal mass (M_r):**	19335
**Calculated pI:**	4.90

Sequence similarity is available as **an NCBI BLAST search of 74. against nr**.

Search parameters

**MS data file:**	O:\FA\02-luty2018\80202246zeb_czas30.raw
**Enzyme:**	Trypsin: cuts C-term side of KR unless next residue is P.
**Fixed modifications:**	**Carbamidomethyl (C)**
**Variable modifications:**	**Oxidation (M)**

Protein sequence coverage: 55%

Matched peptides shown in ***bold red***.

```
  1 MAKVTLTIKN GNVKESQQFE IDKITTFQAL KLKNEIHAIL KDLKNNGELK
 51 EVMEGLFSGE FDVDNMDIKN ITADQLEQMK DEKFITSLAG AFDRLLETVP
101 ERAMNLLSIM SGIDREVLEK AYLEELFDVY DAVMEENDII KLIDRMKRSF
151 FTTKGQWSQA LRTFLANK
```

Unformatted sequence string: **168 residues** (for pasting into other applications).

Sort peptides by ◉ Residue Number ○ Increasing Mass ○ Decreasing Mass

Show predicted peptides also

Query	Start – End	Observed	Mr(expt)	Mr(calc)	ppm	M	Score	Expect	Rank	U	Peptide
10395	15 – 23	562.2662	1122.5178	1122.5193	-1.33	0	56	2.8e-06	1	U	K.ESQQFEIDK.I
10396	15 – 23	562.2665	1122.5185	1122.5193	-0.74	0	53	4.5e-06	1	U	K.ESQQFEIDK.I
10397	15 – 23	562.2681	1122.5217	1122.5193	2.18	0	67	2.2e-07	1	U	K.ESQQFEIDK.I
3847	24 – 31	461.2736	920.5326	920.5331	-0.53	0	59	1.3e-06	1	U	K.ITTFQALK.L
3848	24 – 31	461.2736	920.5327	920.5331	-0.44	0	62	6.2e-07	1	U	K.ITTFQALK.L
3849	24 – 31	461.2736	920.5327	920.5331	-0.44	0	58	1.7e-06	1	U	K.ITTFQALK.L
3853	24 – 31	461.2737	920.5328	920.5331	-0.29	0	58	1.7e-06	1	U	K.ITTFQALK.L
3856	24 – 31	461.2738	920.5331	920.5331	0.039	0	58	1.8e-06	1	U	K.ITTFQALK.L
3859	24 – 31	461.2739	920.5333	920.5331	0.23	0	56	2.6e-06	1	U	K.ITTFQALK.L
12662	32 – 41	393.5793	1177.7162	1177.7182	-1.77	1	73	5.3e-08	1	U	K.LKNEIHAILK.D
12663	32 – 41	393.5793	1177.7162	1177.7182	-1.72	1	63	5.4e-07	1	U	K.LKNEIHAILK.D
12664	32 – 41	393.5795	1177.7167	1177.7182	-1.29	1	67	1.9e-07	1	U	K.LKNEIHAILK.D
12665	32 – 41	393.5797	1177.7174	1177.7182	-0.70	1	69	1.3e-07	1	U	K.LKNEIHAILK.D
12666	32 – 41	393.5798	1177.7176	1177.7182	-0.55	1	66	2.8e-07	1	U	K.LKNEIHAILK.D
12667	32 – 41	589.8665	1177.7185	1177.7182	0.22	1	83	5.5e-09	1	U	K.LKNEIHAILK.D
12668	32 – 41	589.8667	1177.7188	1177.7182	0.46	1	83	5.6e-09	1	U	K.LKNEIHAILK.D
12669	32 – 41	589.8670	1177.7194	1177.7182	0.99	1	89	1.4e-09	1	U	K.LKNEIHAILK.D
12670	32 – 41	589.8670	1177.7194	1177.7182	1.00	1	83	5.4e-09	1	U	K.LKNEIHAILK.D
12671	32 – 41	589.8674	1177.7202	1177.7182	1.63	1	83	5.5e-09	1	U	K.LKNEIHAILK.D
12672	32 – 41	589.8674	1177.7202	1177.7182	1.70	1	69	1.2e-07	1	U	K.LKNEIHAILK.D
29164	70 – 83	831.9020	1661.7894	1661.7930	-2.16	1	114	3.9e-12	1	U	K.NITADQLEQMKDEK.F
29165	70 – 83	831.9032	1661.7918	1661.7930	-0.71	1	112	6.8e-12	1	U	K.NITADQLEQMKDEK.F
29166	70 – 83	831.9034	1661.7923	1661.7930	-0.43	1	114	3.8e-12	1	U	K.NITADQLEQMKDEK.F
29167	70 – 83	831.9034	1661.7923	1661.7930	-0.43	1	112	6.8e-12	1	U	K.NITADQLEQMKDEK.F
29168	70 – 83	831.9035	1661.7923	1661.7930	-0.40	1	124	4.5e-13	1	U	K.NITADQLEQMKDEK.F
29169	70 – 83	831.9035	1661.7924	1661.7930	-0.34	1	115	3.5e-12	1	U	K.NITADQLEQMKDEK.F
29170	70 – 83	831.9036	1661.7926	1661.7930	-0.23	1	121	8.7e-13	1	U	K.NITADQLEQMKDEK.F
29171	70 – 83	554.9382	1661.7927	1661.7930	-0.18	1	69	1.1e-07	1	U	K.NITADQLEQMKDEK.F
29172	70 – 83	831.9037	1661.7929	1661.7930	-0.088	1	120	9.1e-13	1	U	K.NITADQLEQMKDEK.F
29173	70 – 83	554.9383	1661.7931	1661.7930	0.070	1	65	3.2e-07	1	U	K.NITADQLEQMKDEK.F
29174	70 – 83	554.9385	1661.7936	1661.7930	0.32	1	72	6.1e-08	1	U	K.NITADQLEQMKDEK.F
29175	70 – 83	554.9385	1661.7936	1661.7930	0.38	1	84	3.8e-09	1	U	K.NITADQLEQMKDEK.F
29176	70 – 83	554.9386	1661.7940	1661.7930	0.59	1	70	1e-07	1	U	K.NITADQLEQMKDEK.F
29177	70 – 83	831.9044	1661.7943	1661.7930	0.77	1	114	3.7e-12	1	U	K.NITADQLEQMKDEK.F
29178	70 – 83	831.9045	1661.7944	1661.7930	0.81	1	117	1.9e-12	1	U	K.NITADQLEQMKDEK.F
29179	70 – 83	831.9046	1661.7947	1661.7930	0.99	1	95	3.3e-10	1	U	K.NITADQLEQMKDEK.F
29180	70 – 83	554.9389	1661.7947	1661.7930	1.03	1	65	2.9e-07	1	U	K.NITADQLEQMKDEK.F
29181	70 – 83	554.9391	1661.7954	1661.7930	1.44	1	70	9.7e-08	1	U	K.NITADQLEQMKDEK.F
29182	70 – 83	554.9391	1661.7956	1661.7930	1.53	1	73	4.8e-08	1	U	K.NITADQLEQMKDEK.F
29183	70 – 83	554.9392	1661.7957	1661.7930	1.62	1	69	1.2e-07	1	U	K.NITADQLEQMKDEK.F
29184	70 – 83	554.9394	1661.7963	1661.7930	2.00	1	64	4.1e-07	1	U	K.NITADQLEQMKDEK.F

Query	Start – End	Observed	Mr(expt)	Mr(calc)	ppm	M	Score	Expect	Rank	U	Peptide
13401	84 – 94	599.3166	1196.6187	1196.6190	-0.19	0	76	2.8e-08	1	U	K.FITSLAGAFDR.L
13402	84 – 94	599.3170	1196.6195	1196.6190	0.45	0	82	5.9e-09	1	U	K.FITSLAGAFDR.L
13403	84 – 94	599.3171	1196.6196	1196.6190	0.51	0	83	5.6e-09	1	U	K.FITSLAGAFDR.L
13404	84 – 94	599.3171	1196.6196	1196.6190	0.55	0	79	1.3e-08	1	U	K.FITSLAGAFDR.L
13405	84 – 94	599.3172	1196.6198	1196.6190	0.71	0	73	5.6e-08	1	U	K.FITSLAGAFDR.L
13406	84 – 94	599.3172	1196.6198	1196.6190	0.73	0	69	1.3e-07	1	U	K.FITSLAGAFDR.L
13407	84 – 94	599.3173	1196.6200	1196.6190	0.85	0	83	4.7e-09	1	U	K.FITSLAGAFDR.L
13408	84 – 94	599.3173	1196.6200	1196.6190	0.85	0	71	7.6e-08	1	U	K.FITSLAGAFDR.L
13409	84 – 94	599.3173	1196.6200	1196.6190	0.90	0	75	2.9e-08	1	U	K.FITSLAGAFDR.L
13410	84 – 94	599.3174	1196.6202	1196.6190	1.00	0	70	9.2e-08	1	U	K.FITSLAGAFDR.L
13411	84 – 94	599.3174	1196.6202	1196.6190	1.07	0	88	1.6e-09	1	U	K.FITSLAGAFDR.L
13412	84 – 94	599.3175	1196.6204	1196.6190	1.22	0	82	5.7e-09	1	U	K.FITSLAGAFDR.L
13413	84 – 94	599.3175	1196.6204	1196.6190	1.23	0	79	1.3e-08	1	U	K.FITSLAGAFDR.L
13414	84 – 94	599.3175	1196.6205	1196.6190	1.27	0	82	6.1e-09	1	U	K.FITSLAGAFDR.L
13415	84 – 94	599.3177	1196.6208	1196.6190	1.52	0	79	1.4e-08	1	U	K.FITSLAGAFDR.L
13416	84 – 94	599.3177	1196.6208	1196.6190	1.57	0	75	2.9e-08	1	U	K.FITSLAGAFDR.L
13417	84 – 94	599.3179	1196.6212	1196.6190	1.90	0	84	3.5e-09	1	U	K.FITSLAGAFDR.L
4890	95 – 102	478.7738	955.5330	955.5338	-0.88	0	64	4.2e-07	1	U	R.LLETVPER.A
4891	95 – 102	478.7740	955.5333	955.5338	-0.50	0	63	4.6e-07	1	U	R.LLETVPER.A
4892	95 – 102	478.7742	955.5338	955.5338	-0.022	0	56	2.3e-06	1	U	R.LLETVPER.A
4893	95 – 102	478.7743	955.5340	955.5338	0.15	0	69	1.4e-07	1	U	R.LLETVPER.A
4894	95 – 102	478.7743	955.5340	955.5338	0.17	0	69	1.4e-07	1	U	R.LLETVPER.A
4895	95 – 102	478.7743	955.5341	955.5338	0.27	0	60	9.5e-07	1	U	R.LLETVPER.A
4897	95 – 102	478.7745	955.5344	955.5338	0.56	0	56	2.4e-06	1	U	R.LLETVPER.A
4898	95 – 102	478.7745	955.5344	955.5338	0.61	0	59	1.2e-06	1	U	R.LLETVPER.A
4899	95 – 102	478.7745	955.5344	955.5338	0.63	0	60	1.1e-06	1	U	R.LLETVPER.A
4900	95 – 102	478.7746	955.5347	955.5338	0.92	0	53	5.2e-06	1	U	R.LLETVPER.A
4901	95 – 102	478.7747	955.5349	955.5338	1.13	0	54	4.3e-06	1	U	R.LLETVPER.A
4902	95 – 102	478.7749	955.5352	955.5338	1.40	0	60	9.8e-07	1	U	R.LLETVPER.A
21829	103 – 115	710.8675	1419.7205	1419.7214	-0.62	0	94	3.9e-10	1	U	R.AMNLLSIMSGIDR.E
21830	103 – 115	710.8677	1419.7207	1419.7214	-0.44	0	90	1e-09	1	U	R.AMNLLSIMSGIDR.E
21832	103 – 115	710.8678	1419.7210	1419.7214	-0.24	0	92	6.7e-10	1	U	R.AMNLLSIMSGIDR.E
21833	103 – 115	710.8678	1419.7211	1419.7214	-0.22	0	85	3e-09	1	U	R.AMNLLSIMSGIDR.E
21834	103 – 115	710.8683	1419.7220	1419.7214	0.42	0	81	7.8e-09	1	U	R.AMNLLSIMSGIDR.E
21835	103 – 115	710.8689	1419.7233	1419.7214	1.33	0	97	2e-10	1	U	R.AMNLLSIMSGIDR.E
21836	103 – 115	710.8691	1419.7236	1419.7214	1.59	0	100	1.1e-10	1	U	R.AMNLLSIMSGIDR.E
43565	121 – 141	1260.0964	2518.1782	2518.1825	-1.68	0	98	1.7e-10	1	U	K.AYLEELFDVYDAVMEENDIIK.L
43566	121 – 141	840.4011	2518.1816	2518.1825	-0.35	0	112	6.4e-12	1	U	K.AYLEELFDVYDAVMEENDIIK.L

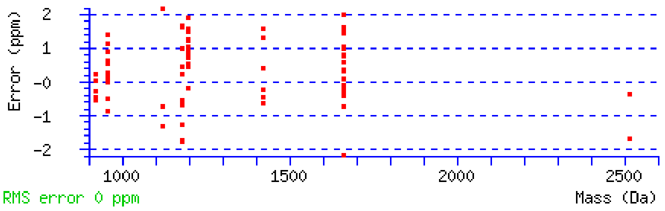

Mascot: http://www.matrixscience.com/

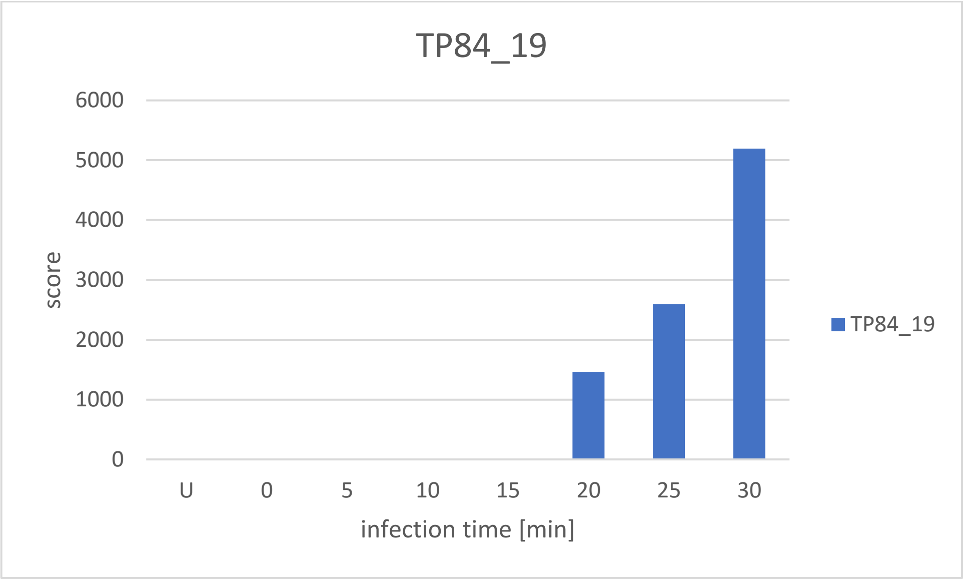

MASCOT Search Results

Protein View: TP84_28

Database: TP84
Score: 1188
Nominal mass (M_r): 44214
Calculated pI: 9.67

Sequence similarity is available as an NCBI BLAST search of 83. against nr.

Search parameters

MS data file: O:\FA\02-luty2018\80202245zeb_czas25.raw
Enzyme: Trypsin: cuts C-term side of KR unless next residue is P.
Fixed modifications: Carbamidomethyl (C)
Variable modifications: Oxidation (M)

Protein sequence coverage: 41%

Matched peptides shown in ***bold red***.

```
  1 MQARSANNIK GIDVSHWQGK IDWAKVKAAG IQVAYLKATE GTTHVDKMLK
 51 TNYQNAKKAG IKVGFYHFFR AKNEQNAREQ ARHFVNTVKG MPNDLKHALD
101 IETTEGLSNE ALTKCAIAFL EEVKKLTGQD PIVYTYTSFA RSRLTAAIAK
151 YPVWIAHYGV DKPGDNPIWD RWIGFQYTDK GKVSGIAGNV DMNEFTSDIF
201 VDAAKVEQPK QKVDAVQSTP SATGTYTIKS GDTFWELEEK YNWPHGTLQR
251 LNPSVNPNAL KVGQVIKVPK SEQPKQNASS VTGTYTIKSG DTFWDLEQKN
301 GWPHGTLQKL NPGVNPNKLK VGQVIKVPKS EQKNVQRTVK NHQKPNYRTY
351 KIKKGDTFWE LEKKNGWPHG TLQKLNPGVN PAKLQIGQTI KIPN
```

Unformatted sequence string: 394 residues (for pasting into other applications).

Sort peptides by ◉ Residue Number ○ Increasing Mass ○ Decreasing Mass

Show predicted peptides also

Query	Start – End	Observed	Mr(expt)	Mr(calc)	ppm	M	Score	Expect	Rank	U	Peptide
7661	11 – 20	563.7873	1125.5601	1125.5567	3.03	0	55	3.2e-06	1	U	K.GIDVSHWQGK.I
27340	97 – 114	647.9971	1940.9695	1940.9691	0.24	0	115	3.5e-12	1	U	K.HALDIETTEGLSNEALTK.C
12557	115 – 125	654.3524	1306.6903	1306.6955	-3.94	1	68	1.6e-07	1	U	K.CAIAFLEEVKK.L
12558	115 – 125	436.5714	1306.6925	1306.6955	-2.31	1	55	3.5e-06	1	U	K.CAIAFLEEVKK.L
12559	115 – 125	436.5715	1306.6925	1306.6955	-2.26	1	54	3.7e-06	1	U	K.CAIAFLEEVKK.L
12560	115 – 125	436.5718	1306.6936	1306.6955	-1.41	1	54	3.6e-06	1	U	K.CAIAFLEEVKK.L
25281	126 – 141	916.4665	1830.9185	1830.9152	1.82	0	86	2.5e-09	1	U	K.LTGQDPIVYTYTSFAR.S
33697	151 – 171	625.3111	2497.2154	2497.2179	-0.99	0	65	3.4e-07	1	U	K.YPVWIAHYGVDKPGDNPIWDR.W
33700	151 – 171	625.3121	2497.2195	2497.2179	0.63	0	68	1.7e-07	1	U	K.YPVWIAHYGVDKPGDNPIWDR.W
32981	183 – 205	1200.5655	2399.1164	2399.1315	-6.27	0	143	5.5e-15	1	U	K.VSGIAGNVDMNEFTSDIFVDAAK.V
23218	213 – 229	869.9462	1737.8778	1737.8785	-0.39	0	106	2.4e-11	1	U	K.VDAVQSTPSATGTYTIK.S
14159	276 – 288	685.3525	1368.6904	1368.6885	1.37	0	90	1e-09	1	U	K.QNASSVTGTYTIK.S
14160	276 – 288	685.3534	1368.6922	1368.6885	2.69	0	90	9.3e-10	1	U	K.QNASSVTGTYTIK.S
14161	276 – 288	685.3551	1368.6957	1368.6885	5.28	0	83	5.2e-09	1	U	K.QNASSVTGTYTIK.S
3779	310 – 318	476.7648	951.5151	951.5138	1.43	0	52	5.7e-06	1	U	K.LNPGVNPNK.L
3780	310 – 318	476.7652	951.5159	951.5138	2.30	0	56	2.8e-06	1	U	K.LNPGVNPNK.L
11095	354 – 363	626.8131	1251.6117	1251.6135	-1.50	1	64	3.9e-07	1	U	K.KGDTFWELEK.K
2831	375 – 383	455.2612	908.5079	908.5080	-0.090	0	66	2.8e-07	1	U	K.LNPGVNPAK.L
2565	384 – 391	450.7790	899.5434	899.5440	-0.71	0	53	4.6e-06	1	U	K.LQIGQTIK.I

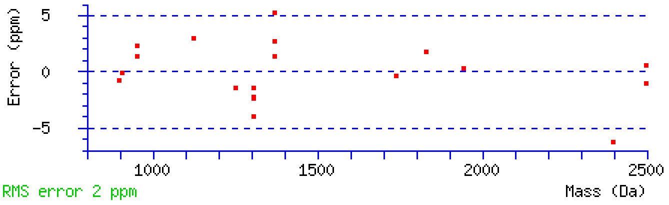

Mascot: http://www.matrixscience.com/

MASCOT Search Results

Protein View: TP84_28

**Database:**	TP84
**Score:**	2309
**Nominal mass (M_r):**	44214
**Calculated pI:**	9.67

Sequence similarity is available as **an NCBI BLAST search of 83. against nr**.

Search parameters

**MS data file:**	O:\FA\02-luty2018\80202246zeb_czas30.raw
**Enzyme:**	Trypsin: cuts C-term side of KR unless next residue is P.
**Fixed modifications:**	**Carbamidomethyl (C)**
**Variable modifications:**	**Oxidation (M)**

Protein sequence coverage: 37%

Matched peptides shown in ***bold red***.

1 MQARSANNIK GIDVSHWQGK IDWAKVKAAG IQVAYLKATE GTTHVDKMLK
51 TNYQNAKKAG IKVGFYHFFR AKNEQNAREQ AR**HFVNTVK**G MPNDLK**HALD**
101 **IETTEGLSNE ALTKCAIAFL EEVKKLTGQD PIVYTYTSFA R**SRLTAAIAK
151 **YPVWIAHYGV DKPGDNPIWD R**WIGFQYTDK GK**VSGIAGNV DMNEFTSDIF**
201 **VDAAK**VEQPK QKVDAVQSTP SATGTYTIK**S GDTFWELEEK** YNWPHGTLQR
251 **LNPSVNPNAL K**VGQVIKVPK SEQPKQNASS VTGTYTIK**SG DTFWDLEQK**N
301 GWPHGTLQK**L NPGVNPNK**LK VGQVIKVPKS EQKNVQRTVK NHQKPNYRTY
351 KIKKGDTFWE LEKKNGWPHG TLQKLNPGVN PAK**LQIGQTI K**IPN

Unformatted sequence string: **394 residues** (for pasting into other applications).

Sort peptides by ◉ Residue Number ○ Increasing Mass ○ Decreasing Mass

Show predicted peptides also

Query	Start – End	Observed	Mr(expt)	Mr(calc)	ppm	M	Score	Expect	Rank	U	Peptide
2192	83 – 89	422.7376	843.4607	843.4603	0.51	0	59	1.4e-06	1	U	R.HFVNTVK.G
2193	83 – 89	422.7380	843.4614	843.4603	1.29	0	54	3.6e-06	1	U	R.HFVNTVK.G
36326	97 – 114	971.4907	1940.9669	1940.9691	-1.13	0	136	2.3e-14	1	U	K.HALDIETTEGLSNEALTK.C
36327	97 – 114	971.4915	1940.9684	1940.9691	-0.35	0	154	4.4e-16	1	U	K.HALDIETTEGLSNEALTK.C
36328	97 – 114	971.4916	1940.9687	1940.9691	-0.18	0	147	2e-15	1	U	K.HALDIETTEGLSNEALTK.C
36329	97 – 114	647.9980	1940.9721	1940.9691	1.54	0	81	7.2e-09	1	U	K.HALDIETTEGLSNEALTK.C
36330	97 – 114	971.4939	1940.9733	1940.9691	2.17	0	63	4.7e-07	1	U	K.HALDIETTEGLSNEALTK.C
17750	115 – 125	436.5717	1306.6934	1306.6955	-1.62	1	56	2.7e-06	1	U	K.CAIAFLEEVKK.L
33987	126 – 141	916.4617	1830.9088	1830.9152	-3.48	0	66	2.3e-07	1	U	K.LTGQDPIVYTYTSFAR.S
33988	126 – 141	916.4621	1830.9096	1830.9152	-3.07	0	81	8.7e-09	1	U	K.LTGQDPIVYTYTSFAR.S
33989	126 – 141	916.4636	1830.9127	1830.9152	-1.38	0	75	3.2e-08	1	U	K.LTGQDPIVYTYTSFAR.S
43457	151 – 171	833.4121	2497.2146	2497.2179	-1.33	0	111	7.3e-12	1	U	K.YPVWIAHYGVDKPGDNPIWDR.W
43459	151 – 171	625.3114	2497.2166	2497.2179	-0.51	0	56	2.3e-06	1	U	K.YPVWIAHYGVDKPGDNPIWDR.W
43460	151 – 171	625.3116	2497.2171	2497.2179	-0.31	0	61	7.4e-07	1	U	K.YPVWIAHYGVDKPGDNPIWDR.W
43461	151 – 171	625.3117	2497.2178	2497.2179	-0.042	0	70	9e-08	1	U	K.YPVWIAHYGVDKPGDNPIWDR.W
43463	151 – 171	625.3118	2497.2180	2497.2179	0.038	0	62	6e-07	1	U	K.YPVWIAHYGVDKPGDNPIWDR.W
43465	151 – 171	625.3119	2497.2186	2497.2179	0.28	0	67	2e-07	1	U	K.YPVWIAHYGVDKPGDNPIWDR.W
43466	151 – 171	625.3124	2497.2205	2497.2179	1.05	0	66	2.3e-07	1	U	K.YPVWIAHYGVDKPGDNPIWDR.W
42718	183 – 205	1200.5717	2399.1288	2399.1315	-1.11	0	134	4.1e-14	1	U	K.VSGIAGNVDMNEFTSDIFVDAAK.V
42719	183 – 205	800.7201	2399.1383	2399.1315	2.84	0	87	1.9e-09	1	U	K.VSGIAGNVDMNEFTSDIFVDAAK.V
42720	183 – 205	1200.5771	2399.1396	2399.1315	3.40	0	130	1.1e-13	1	U	K.VSGIAGNVDMNEFTSDIFVDAAK.V
42721	183 – 205	800.7236	2399.1491	2399.1315	7.32	0	93	5.3e-10	1	U	K.VSGIAGNVDMNEFTSDIFVDAAK.V
18984	230 – 240	670.8032	1339.5919	1339.5932	-0.97	0	66	2.7e-07	1	U	K.SGDTFWELEEK.Y
18985	230 – 240	670.8035	1339.5924	1339.5932	-0.61	0	79	1.3e-08	1	U	K.SGDTFWELEEK.Y
12029	251 – 261	583.8295	1165.6445	1165.6455	-0.83	0	55	3.2e-06	1	U	R.LNPSVNPNALK.V
12030	251 – 261	583.8298	1165.6451	1165.6455	-0.31	0	51	7.9e-06	1	U	R.LNPSVNPNALK.V
12031	251 – 261	583.8300	1165.6455	1165.6455	-0.021	0	54	4.3e-06	1	U	R.LNPSVNPNALK.V
18422	289 – 299	663.3050	1324.5955	1324.5936	1.48	0	72	5.6e-08	1	U	K.SGDTFWDLEQK.N
18424	289 – 299	663.3057	1324.5969	1324.5936	2.52	0	76	2.5e-08	1	U	K.SGDTFWDLEQK.N
4770	310 – 318	476.7645	951.5144	951.5138	0.64	0	64	4.3e-07	1	U	K.LNPGVNPNK.L
4773	310 – 318	476.7648	951.5150	951.5138	1.31	0	67	1.9e-07	1	U	K.LNPGVNPNK.L
4774	310 – 318	476.7648	951.5150	951.5138	1.33	0	63	5e-07	1	U	K.LNPGVNPNK.L
3341	384 – 391	450.7789	899.5432	899.5440	-0.89	0	51	7.4e-06	1	U	K.LQIGQTIK.I
3342	384 – 391	450.7790	899.5434	899.5440	-0.64	0	51	8.6e-06	1	U	K.LQIGQTIK.I
3346	384 – 391	450.7791	899.5437	899.5440	-0.38	0	54	4.4e-06	1	U	K.LQIGQTIK.I
3350	384 – 391	450.7795	899.5444	899.5440	0.42	0	51	8.9e-06	1	U	K.LQIGQTIK.I

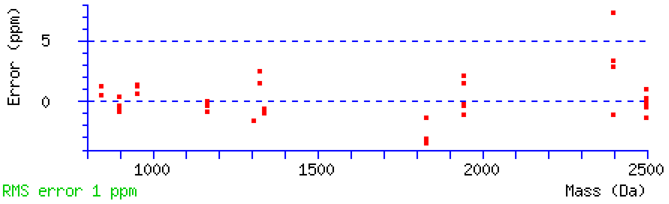

Mascot: http://www.matrixscience.com/

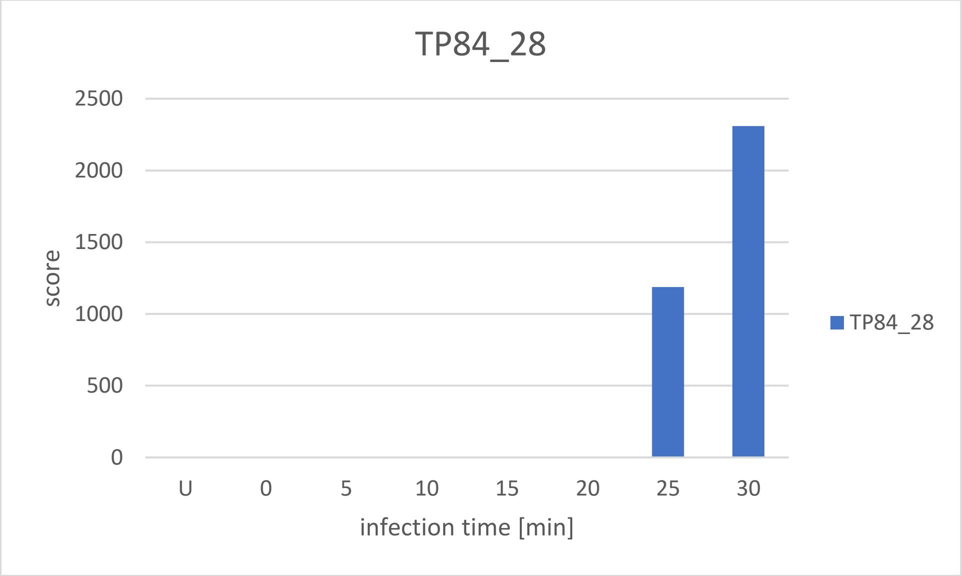

MASCOT Search Results

Protein View: TP84_36

**Database:**	TP84
**Score:**	440
**Nominal mass (M_r):**	9310
**Calculated pI:**	9.91

Sequence similarity is available as **an NCBI BLAST search of 6. against nr**.

Search parameters

**MS data file:**	O:\FA\02-luty2018\80202243zeb_czas15.raw
**Enzyme:**	Trypsin: cuts C-term side of KR unless next residue is P.
**Fixed modifications:**	**Carbamidomethyl (C)**
**Variable modifications:**	**Oxidation (M)**

Protein sequence coverage: 32%

Matched peptides shown in ***bold red***.

```
 1 MVKQMTDAQR KAIEWAVKMA ESQIKSSQRR IKRAERSLEL FKGMDELDDK
51 LKRRQIKHQQ NRIESLQNYI DALKSLLD
```

Unformatted sequence string: **78 residues** (for pasting into other applications).

Sort peptides by ◉ Residue Number ○ Increasing Mass ○ Decreasing Mass

Show predicted peptides also

Query	Start – End	Observed	Mr(expt)	Mr(calc)	ppm	M	Score	Expect	Rank	U	Peptide
1153	12 – 18	408.7341	815.4537	815.4541	-0.55	0	46	2.7e-05	1	U	K.AIEWAVK.M
301	37 – 42	368.7160	735.4174	735.4167	0.97	0	53	4.7e-06	1	U	R.SLELFK.G
12621	63 – 74	703.8797	1405.7448	1405.7453	-0.30	0	119	1.2e-12	1	U	R.IESLQNYIDALK.S
12622	63 – 74	703.8815	1405.7484	1405.7453	2.27	0	88	1.5e-09	1	U	R.IESLQNYIDALK.S
12623	63 – 74	703.8817	1405.7488	1405.7453	2.53	0	97	2.2e-10	1	U	R.IESLQNYIDALK.S
12624	63 – 74	703.8819	1405.7493	1405.7453	2.91	0	102	6.1e-11	1	U	R.IESLQNYIDALK.S

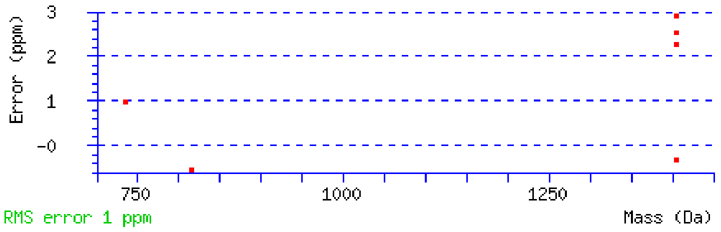

Mascot: **http://www.matrixscience.com/**

MASCOT Search Results

Protein View: TP84_36

Database: TP84
Score: 807
Nominal mass (M_r): 9310
Calculated pI: 9.91

Sequence similarity is available as an NCBI BLAST search of 6. against nr.

Search parameters

MS data file: O:\FA\02-luty2018\80202244zeb_czas20.raw
Enzyme: Trypsin: cuts C-term side of KR unless next residue is P.
Fixed modifications: Carbamidomethyl (C)
Variable modifications: Oxidation (M)

Protein sequence coverage: 44%

Matched peptides shown in ***bold red***.

```
 1 MVKQMTDAQR KAIEWAVKMA ESQIKSSQRR IKRAERSLEL FKGMDELDDK
51 LKRRQIKHQQ NRIESLQNYI DALKSLLD
```

Unformatted sequence string: 78 residues (for pasting into other applications).

Sort peptides by ◉ Residue Number ○ Increasing Mass ○ Decreasing Mass

Show predicted peptides also

Query	Start – End	Observed	Mr(expt)	Mr(calc)	ppm	M	Score	Expect	Rank	U	Peptide
1990	12 – 18	408.7365	815.4584	815.4541	5.31	0	53	4.7e-06	1	U	K.AIEWAVK.M
556	37 – 42	368.7168	735.4190	735.4167	3.15	0	58	1.5e-06	1	U	R.SLELFK.G
557	37 – 42	368.7169	735.4193	735.4167	3.58	0	54	4.1e-06	1	U	R.SLELFK.G
559	37 – 42	368.7170	735.4194	735.4167	3.72	0	58	1.5e-06	1	U	R.SLELFK.G
560	37 – 42	368.7170	735.4195	735.4167	3.91	0	54	4.2e-06	1	U	R.SLELFK.G
570	37 – 42	368.7179	735.4212	735.4167	6.11	0	21	0.0073	1	U	R.SLELFK.G
572	37 – 42	368.7181	735.4216	735.4167	6.77	0	58	1.4e-06	1	U	R.SLELFK.G
13669	43 – 52	388.5262	1162.5567	1162.5540	2.36	1	40	0.0001	1	U	K.GMDELDDKLK.R
13670	43 – 52	388.5264	1162.5573	1162.5540	2.85	1	59	1.2e-06	1	U	K.GMDELDDKLK.R
13671	43 – 52	388.5272	1162.5599	1162.5540	5.12	1	57	1.9e-06	1	U	K.GMDELDDKLK.R
13672	43 – 52	582.2890	1162.5634	1162.5540	8.14	1	64	3.6e-07	1	U	K.GMDELDDKLK.R
23049	63 – 74	703.8814	1405.7483	1405.7453	2.16	0	105	3.2e-11	1	U	R.IESLQNYIDALK.S
23050	63 – 74	703.8820	1405.7494	1405.7453	2.94	0	102	5.8e-11	1	U	R.IESLQNYIDALK.S
23051	63 – 74	703.8822	1405.7498	1405.7453	3.21	0	102	6.2e-11	1	U	R.IESLQNYIDALK.S
23052	63 – 74	703.8827	1405.7508	1405.7453	3.98	0	103	5.5e-11	1	U	R.IESLQNYIDALK.S

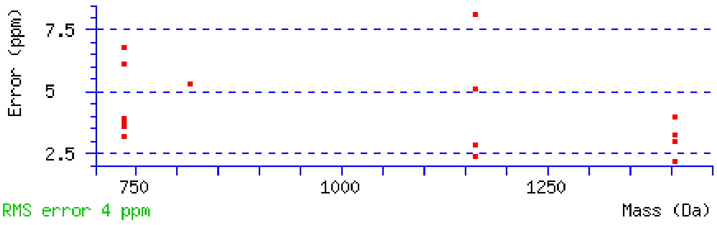

Mascot: http://www.matrixscience.com/

MASCOT Search Results

Protein View: TP84_36

Database: TP84
Score: 742
Nominal mass (M_r): 9310
Calculated pI: 9.91

Sequence similarity is available as **an NCBI BLAST search of 6. against nr**.

Search parameters

MS data file: O:\FA\02-luty2018\80202245zeb_czas25.raw
Enzyme: Trypsin: cuts C-term side of KR unless next residue is P.
Fixed modifications: Carbamidomethyl (C)
Variable modifications: Oxidation (M)

Protein sequence coverage: 32%

Matched peptides shown in ***bold red***.

```
 1 MVKQMTDAQR KAIEWAVKMA ESQIKSSQRR IKRAERSLEL FKGMDELDDK
51 LKRRQIKHQQ NRIESLQNYI DALKSLLD
```

Unformatted sequence string: **78 residues** (for pasting into other applications).

Sort peptides by ◉ Residue Number ○ Increasing Mass ○ Decreasing Mass

Show predicted peptides also

Query	Start - End	Observed	Mr(expt)	Mr(calc)	ppm	M	Score	Expect	Rank	U	Peptide
1925	4 - 10	425.1977	848.3809	848.3810	-0.21	0	53	5e-06	1	U	K.QMTDAQR.K
329	37 - 42	368.7152	735.4158	735.4167	-1.23	0	50	9.5e-06	1	U	R.SLELFK.G
334	37 - 42	368.7157	735.4169	735.4167	0.29	0	58	1.5e-06	1	U	R.SLELFK.G
15198	63 - 74	703.8793	1405.7440	1405.7453	-0.89	0	113	5.4e-12	1	U	R.IESLQNYIDALK.S
15199	63 - 74	703.8795	1405.7444	1405.7453	-0.57	0	113	5.5e-12	1	U	R.IESLQNYIDALK.S
15200	63 - 74	703.8800	1405.7455	1405.7453	0.21	0	102	5.7e-11	1	U	R.IESLQNYIDALK.S
15201	63 - 74	703.8802	1405.7458	1405.7453	0.42	0	110	1e-11	1	U	R.IESLQNYIDALK.S
15202	63 - 74	703.8804	1405.7463	1405.7453	0.72	0	107	1.9e-11	1	U	R.IESLQNYIDALK.S
15203	63 - 74	703.8813	1405.7481	1405.7453	2.03	0	103	5.5e-11	1	U	R.IESLQNYIDALK.S
15204	63 - 74	703.8834	1405.7522	1405.7453	4.97	0	50	9.9e-06	1	U	R.IESLQNYIDALK.S

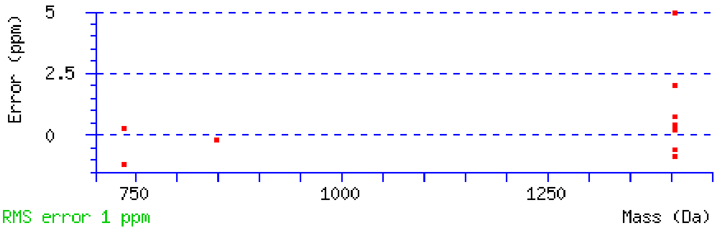

Mascot: http://www.matrixscience.com/

MASCOT Search Results

Protein View: TP84_36

Database: TP84
Score: 950
Nominal mass (M_r): 9310
Calculated pI: 9.91

Sequence similarity is available as **an NCBI BLAST search of 6. against nr**.

Search parameters

MS data file: O:\FA\02-luty2018\80202246zeb_czas30.raw
Enzyme: Trypsin: cuts C-term side of KR unless next residue is P.
Fixed modifications: Carbamidomethyl (C)
Variable modifications: Oxidation (M)

Protein sequence coverage: 23%

Matched peptides shown in ***bold red***.

```
 1 MVKQMTDAQR KAIEWAVKMA ESQIKSSQRR IKRAERSLEL FKGMDELDDK
51 LKRRQIKHQQ NRIESLQNYI DALKSLLD
```

Unformatted sequence string: **78 residues** (for pasting into other applications).

Sort peptides by ◉ Residue Number ○ Increasing Mass ○ Decreasing Mass

Show predicted peptides also

Query	Start - End	Observed	Mr(expt)	Mr(calc)	ppm	M	Score	Expect	Rank	U	Peptide
419	37 - 42	368.7149	735.4152	735.4167	-1.99	0	58	1.5e-06	1	U	R.SLELFK.G
21415	63 - 74	703.8789	1405.7433	1405.7453	-1.41	0	112	5.7e-12	1	U	R.IESLQNYIDALK.S
21416	63 - 74	703.8793	1405.7441	1405.7453	-0.80	0	105	3.4e-11	1	U	R.IESLQNYIDALK.S
21417	63 - 74	703.8800	1405.7454	1405.7453	0.11	0	102	5.6e-11	1	U	R.IESLQNYIDALK.S
21418	63 - 74	703.8807	1405.7469	1405.7453	1.16	0	105	3.3e-11	1	U	R.IESLQNYIDALK.S
21419	63 - 74	703.8813	1405.7479	1405.7453	1.92	0	105	3.3e-11	1	U	R.IESLQNYIDALK.S
21420	63 - 74	703.8813	1405.7480	1405.7453	1.93	0	105	3.2e-11	1	U	R.IESLQNYIDALK.S
21421	63 - 74	703.8817	1405.7488	1405.7453	2.54	0	107	1.9e-11	1	U	R.IESLQNYIDALK.S
21422	63 - 74	703.8817	1405.7489	1405.7453	2.58	0	89	1.3e-09	1	U	R.IESLQNYIDALK.S
21423	63 - 74	703.8821	1405.7496	1405.7453	3.10	0	98	1.5e-10	1	U	R.IESLQNYIDALK.S
21424	63 - 74	703.8828	1405.7511	1405.7453	4.19	0	93	4.6e-10	1	U	R.IESLQNYIDALK.S

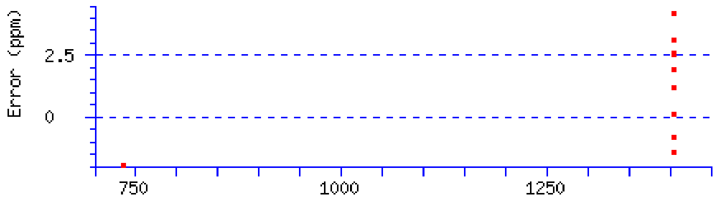

RMS error 2 ppm

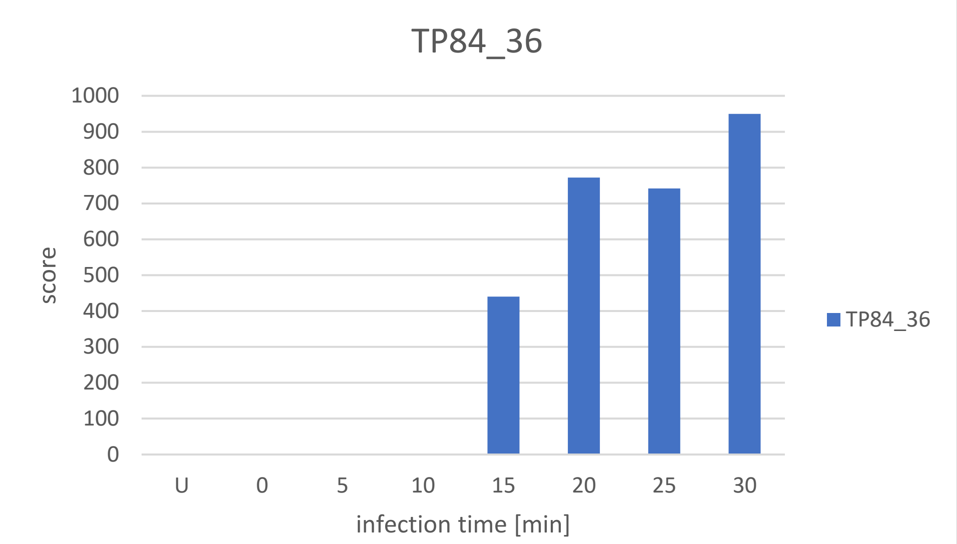

12.02.2018

MASCOT Search Results

Protein View: TP84_37

**Database:**	TP84
**Score:**	315
**Nominal mass (M_r):**	14846
**Calculated pI:**	6.41

Sequence similarity is available as an NCBI BLAST search of 8. against nr.

Search parameters

**MS data file:**	O:\FA\02-luty2018\80202246zeb_czas30.raw
**Enzyme:**	Trypsin: cuts C-term side of KR unless next residue is P.
**Fixed modifications:**	Carbamidomethyl (C)
**Variable modifications:**	Oxidation (M)

Protein sequence coverage: 15%

Matched peptides shown in ***bold red***.

```
  1 MQVRKIHKVT YESETMPLEQ AVEEIAQSTG SPVDRVRGLI IQFGKVMTVH
 51 HVYHIVDASG IGLVKDAEKF LGQSMVFQLK GGETVILKVM EVTRWMNGDY
101 FIKGYDDEGM WRTIDVDSIK AYFTPGKGMI R
```

Unformatted sequence string: 131 residues (for pasting into other applications).

Sort peptides by ◉ Residue Number ○ Increasing Mass ○ Decreasing Mass

Show predicted peptides also

Query	Start - End	Observed	Mr(expt)	Mr(calc)	ppm	M	Score	Expect	Rank	U	Peptide
17191	70 - 80	649.3530	1296.6915	1296.6900	1.14	0	77	2.1e-08	1	U	K.FLGQSMVFQLK.G
17192	70 - 80	649.3533	1296.6921	1296.6900	1.62	0	76	2.3e-08	1	U	K.FLGQSMVFQLK.G
17193	70 - 80	649.3534	1296.6922	1296.6900	1.68	0	78	1.7e-08	1	U	K.FLGQSMVFQLK.G
17194	70 - 80	649.3537	1296.6928	1296.6900	2.11	0	80	1.1e-08	1	U	K.FLGQSMVFQLK.G
10565	104 - 112	564.7244	1127.4343	1127.4342	0.057	0	57	1.9e-06	1	U	K.GYDDEGMWR.T

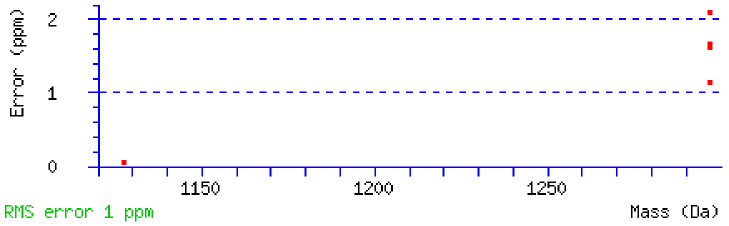

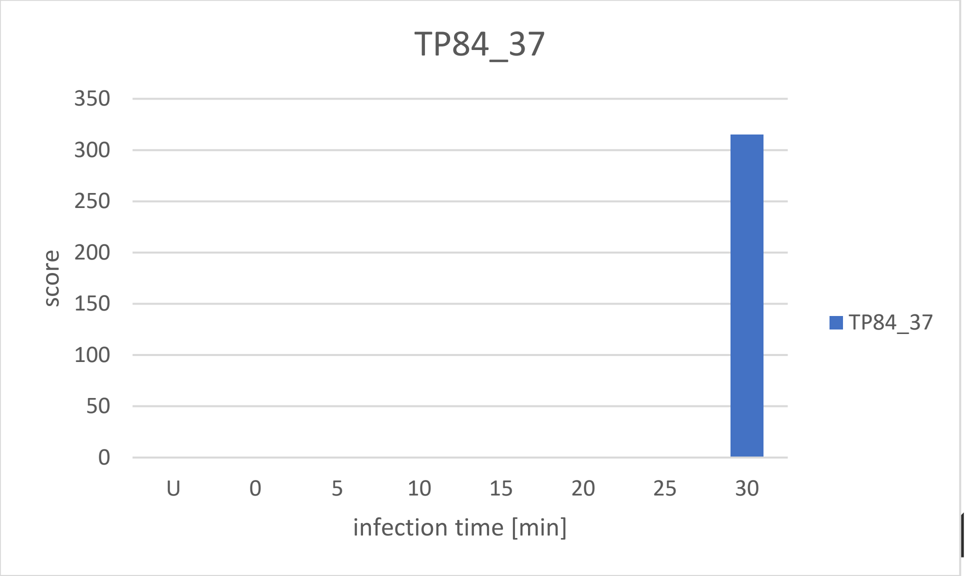

MASCOT Search Results

Protein View: TP84_38

Database: TP84
Score: 186
Nominal mass (M_r): 7380
Calculated pI: 6.73

Sequence similarity is available as **an NCBI BLAST search of 9. against nr**.

Search parameters

MS data file: O:\FA\02-luty2018\80202243zeb_czas15.raw
Enzyme: Trypsin: cuts C-term side of KR unless next residue is P.
Fixed modifications: **Carbamidomethyl (C)**
Variable modifications: **Oxidation (M)**

Protein sequence coverage: 34%

Matched peptides shown in ***bold red***.

```
 1 MKPYDPIPMA ITLKPHQTIR KGEVIQETIG GKVVKFKVID IRMVEIRETH
51 ALVGVMAREI GEGDAE
```

Unformatted sequence string: **66 residues** (for pasting into other applications).

Sort peptides by ◉ Residue Number ○ Increasing Mass ○ Decreasing Mass

Show predicted peptides also

Query	Start – End	Observed	Mr(expt)	Mr(calc)	ppm	M	Score	Expect	Rank	U	Peptide
9268	21 – 32	629.8534	1257.6923	1257.6929	-0.40	1	122	6.9e-13	1	U	R.KGEVIQETIGGK.V
7435	48 – 58	395.2131	1182.6174	1182.6179	-0.48	0	33	0.00047	1	U	R.ETHALVGVMAR.E
7436	48 – 58	395.2133	1182.6181	1182.6179	0.15	0	57	1.8e-06	1	U	R.ETHALVGVMAR.E

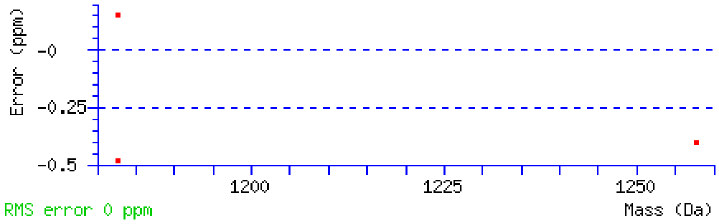

MASCOT Search Results

Protein View: TP84_38

Database: TP84
Score: 494
Nominal mass (M_r): 7380
Calculated pI: 6.73

Sequence similarity is available as **an NCBI BLAST search of 9. against nr**.

Search parameters

MS data file: O:\FA\02-luty2018\80202244zeb_czas20.raw
Enzyme: Trypsin: cuts C-term side of KR unless next residue is P.
Fixed modifications: **Carbamidomethyl (C)**
Variable modifications: **Oxidation (M)**

Protein sequence coverage: 18%

Matched peptides shown in ***bold red***.

```
 1 MKPYDPIPMA ITLKPHQTIR KGEVIQETIG GKVVKFKVID IRMVEIRETH
51 ALVGVMAREI GEGDAE
```

Unformatted sequence string: **66 residues** (for pasting into other applications).

Sort peptides by ◉ Residue Number ○ Increasing Mass ○ Decreasing Mass

Show predicted peptides also

Query	Start – End	Observed	Mr(expt)	Mr(calc)	ppm	M	Score	Expect	Rank	U	Peptide
17637	21 – 32	629.8556	1257.6967	1257.6929	3.03	1	87	1.9e-09	1	U	R.KGEVIQETIGGK.V
17639	21 – 32	629.8570	1257.6995	1257.6929	5.30	1	126	2.6e-13	1	U	R.KGEVIQETIGGK.V
17640	21 – 32	629.8570	1257.6995	1257.6929	5.30	1	110	1e-11	1	U	R.KGEVIQETIGGK.V
17641	21 – 32	629.8572	1257.6998	1257.6929	5.54	1	131	8e-14	1	U	R.KGEVIQETIGGK.V
17642	21 – 32	629.8581	1257.7017	1257.6929	7.02	1	92	6.6e-10	1	U	R.KGEVIQETIGGK.V

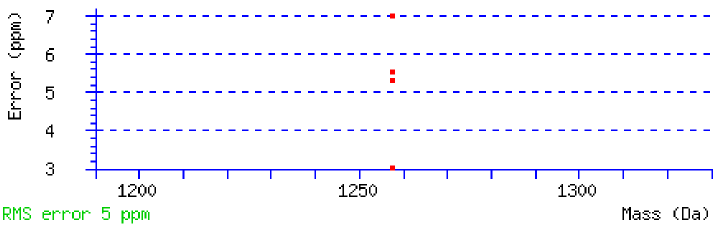

Mascot: http://www.matrixscience.com/

MASCOT Search Results

Protein View: TP84_38

**Database:**	TP84
**Score:**	304
**Nominal mass (M_r):**	7380
**Calculated pI:**	6.73

Sequence similarity is available as an NCBI BLAST search of 9. against nr.

Search parameters

**MS data file:**	O:\FA\02-luty2018\80202245zeb_czas25.raw
**Enzyme:**	Trypsin: cuts C-term side of KR unless next residue is P.
**Fixed modifications:**	Carbamidomethyl (C)
**Variable modifications:**	Oxidation (M)

Protein sequence coverage: 34%

Matched peptides shown in ***bold red***.

```
 1 MKPYDPIPMA ITLKPHQTIR KGEVIQETIG GKVVKFKVID IRMVEIRETH
51 ALVGVMAREI GEGDAE
```

Unformatted sequence string: 66 residues (for pasting into other applications).

Sort peptides by ◉ Residue Number ○ Increasing Mass ○ Decreasing Mass

Show predicted peptides also

Query	Start - End	Observed	Mr(expt)	Mr(calc)	ppm	M	Score	Expect	Rank	U	Peptide
11288	21 - 32	629.8525	1257.6904	1257.6929	-1.93	1	126	2.4e-13	1	U	R.KGEVIQETIGGK.V
9100	48 - 58	592.3179	1182.6213	1182.6179	2.85	0	75	2.8e-08	1	U	R.ETHALVGVMAR.E
9101	48 - 58	592.3180	1182.6214	1182.6179	2.93	0	73	5.2e-08	1	U	R.ETHALVGVMAR.E
9102	48 - 58	592.3182	1182.6219	1182.6179	3.37	0	68	1.6e-07	1	U	R.ETHALVGVMAR.E

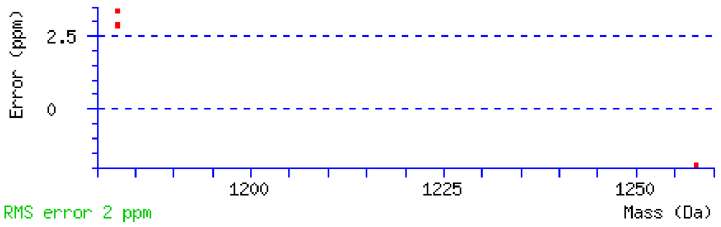

Mascot: http://www.matrixscience.com/

MASCOT Search Results

Protein View: TP84_38

**Database:**	TP84
**Score:**	420
**Nominal mass (M_r):**	7380
**Calculated pI:**	6.73

Sequence similarity is available as **an NCBI BLAST search of 9. against nr**.

Search parameters

**MS data file:**	O:\FA\02-luty2018\80202246zeb_czas30.raw
**Enzyme:**	Trypsin: cuts C-term side of KR unless next residue is P.
**Fixed modifications:**	**Carbamidomethyl (C)**
**Variable modifications:**	**Oxidation (M)**

Protein sequence coverage: 18%

Matched peptides shown in ***bold red***.

```
 1 MKPYDPIPMA ITLKPHQTIR KGEVIQETIG GKVVKFKVID IRMVEIRETH
51 ALVGVMAREI GEGDAE
```

Unformatted sequence string: **66 residues** (for pasting into other applications).

Sort peptides by ◉ Residue Number ○ Increasing Mass ○ Decreasing Mass

Show predicted peptides also

Query	Start – End	Observed	Mr(expt)	Mr(calc)	ppm	M	Score	Expect	Rank	U	Peptide
15931	21 – 32	629.8529	1257.6913	1257.6929	-1.20	1	108	1.5e-11	1	U	R.KGEVIQETIGGK.V
15932	21 – 32	629.8531	1257.6915	1257.6929	-1.04	1	121	7.6e-13	1	U	R.KGEVIQETIGGK.V
15936	21 – 32	629.8536	1257.6926	1257.6929	-0.21	1	108	1.6e-11	1	U	R.KGEVIQETIGGK.V
15938	21 – 32	629.8548	1257.6950	1257.6929	1.71	1	122	7.1e-13	1	U	R.KGEVIQETIGGK.V

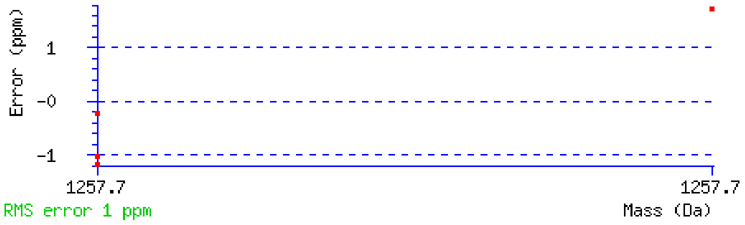

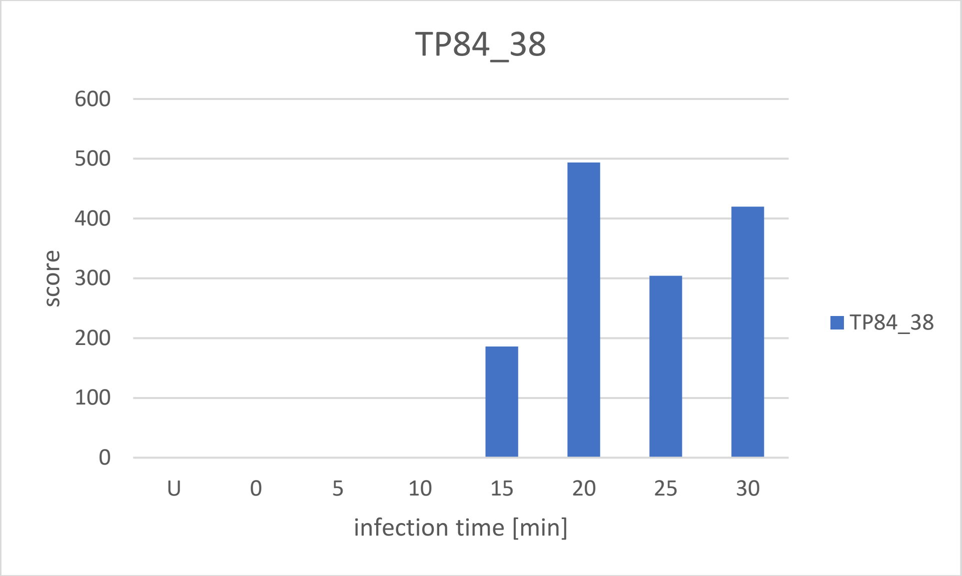

MASCOT Search Results

Protein View: TP84_53

**Database:**	TP84
**Score:**	333
**Nominal mass (M_r):**	38747
**Calculated pI:**	5.33

Sequence similarity is available as **an NCBI BLAST search of 25. against nr**.

Search parameters

**MS data file:**	O:\FA\02-luty2018\80202246zeb_czas30.raw
**Enzyme:**	Trypsin: cuts C-term side of KR unless next residue is P.
**Fixed modifications:**	Carbamidomethyl (C)
**Variable modifications:**	Oxidation (M)

Protein sequence coverage: 10%

Matched peptides shown in ***bold red***.

```
  1 MRKWAVLTDD LKFCEITFDG EWYRVRITDE FGQELMTNES ISLQGAKKQV
 51 KRFIGTGQTL KWQEVETDKI ETPSKLHLPF GYLSVSQVRK YLTCPRAYEF
101 KYVNKLNEPI GSTLVMGRAF HKGMQMASIK KVVDGEILST DDVLDVYSDA
151 FDQERENNDV DWAEDDPAKV KDDGAKLMQK YYEEMGINAI PMVDDRGLPL
201 VEREHAFEIV PGLKAKAVID IIEQDGSIRD YKTSKRSPSQ TIIDETIQMP
251 VYALAYRDIT GQVEKTVGLD YAVNLKKEKK IMRLETDGPV DDGRIERVKQ
301 TFVGVAKAIS AGIFYPNEES NACGYCSFKD ICKKSKTF
```

Unformatted sequence string: **338 residues** (for pasting into other applications).

Sort peptides by ◉ Residue Number ○ Increasing Mass ○ Decreasing Mass

Show predicted peptides also

Query	Start – End	Observed	Mr(expt)	Mr(calc)	ppm	M	Score	Expect	Rank	U	Peptide
20795	106 – 118	693.8754	1385.7362	1385.7337	1.80	0	106	2.4e-11	1	U	K.LNEPIGSTLVMGR.A
20796	106 – 118	693.8775	1385.7404	1385.7337	4.87	0	88	1.8e-09	1	U	K.LNEPIGSTLVMGR.A
14975	204 – 214	620.3402	1238.6659	1238.6659	-0.033	0	61	8.1e-07	1	U	R.EHAFEIVPGLK.A
12441	284 – 294	587.2735	1172.5325	1172.5310	1.32	0	64	4.1e-07	1	U	R.LETDGPVDDGR.I
12442	284 – 294	587.2737	1172.5329	1172.5310	1.64	0	67	2.2e-07	1	U	R.LETDGPVDDGR.I

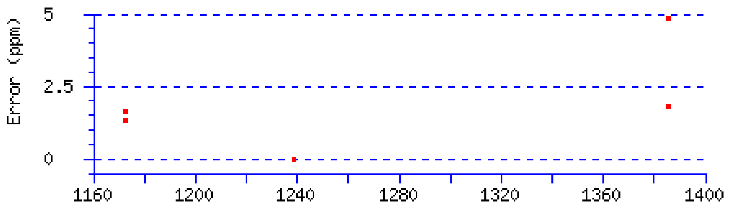

RMS error 2 ppm

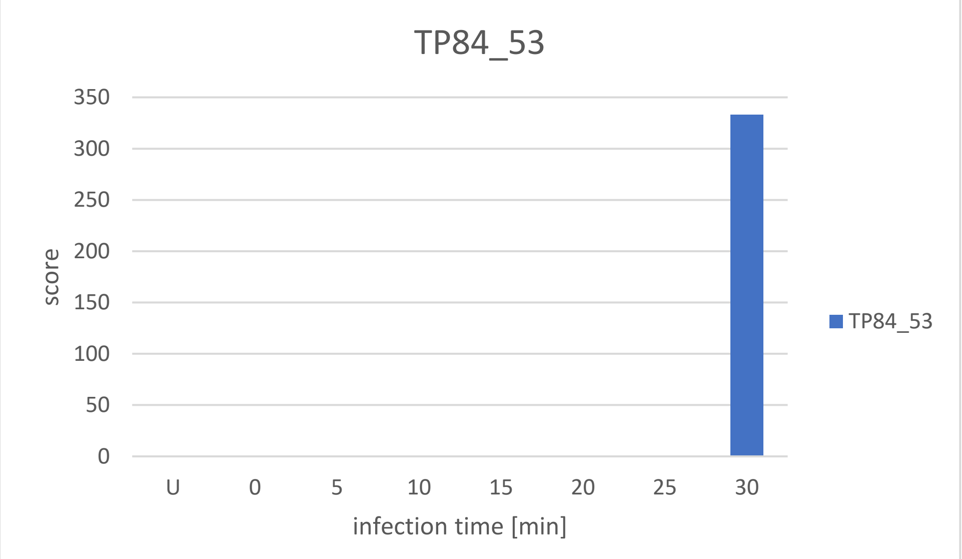

MASCOT Search Results

Protein View: TP84_54

**Database:**	TP84
**Score:**	542
**Nominal mass (M_r):**	38765
**Calculated pI:**	5.78

Sequence similarity is available as an NCBI BLAST search of 26. against nr.

Search parameters

**MS data file:**	O:\FA\02-luty2018\80202246zeb_czas30.raw
**Enzyme:**	Trypsin: cuts C-term side of KR unless next residue is P.
**Fixed modifications:**	Carbamidomethyl (C)
**Variable modifications:**	Oxidation (M)

Protein sequence coverage: 14%

Matched peptides shown in ***bold red***.

```
  1 MGFDMQYFSE KEAAQQYQDAV EKLTEQNTIQ VVSLNDIKQK LLEEVKEFEF
 51 SPLEYVPGPD ETQKTIFLKI ANIMGELDRI PKSGYNKQFD YYYVTESDVV
101 GAVRPLMAKY KLVMMPSLKK YKTETIQGKY NKMIMGTVEI EWIVRDAESY
151 EQIKFTMLGK GLDNLEKDIY KAITGNKKYA LITLFMIDSG DDPERNDTPT
201 GEEGPQNAQN GPENTKKTQG NSKTPGQPKN GQEVPKNASQ GQPQQPPQQP
251 PVKQPTKGDL LTRWVVLAGP DQKQEVRKA FDEWYEKKKA EGWDHMAMMQ
301 ALTKKLHEKN QKEKQAQQQQ ADGQSGQAQQ QQTDDKSKE
```

Unformatted sequence string: 339 residues (for pasting into other applications).

Sort peptides by ◉ Residue Number ○ Increasing Mass ○ Decreasing Mass

Show predicted peptides also

Query	Start – End	Observed	Mr(expt)	Mr(calc)	ppm	M	Score	Expect	Rank	U	Peptide
17755	12 – 22	654.8074	1307.6002	1307.5993	0.64	0	72	6.3e-08	1	U	K.EAAQQYQDAVEK.L
38893	47 – 64	1056.4870	2110.9594	2110.9735	-6.66	0	69	1.3e-07	1	U	K.EFEFSPLEYVPGPDETQK.T
38894	47 – 64	1056.4876	2110.9606	2110.9735	-6.09	0	75	2.9e-08	1	U	K.EFEFSPLEYVPGPDETQK.T
38895	47 – 64	1056.4885	2110.9624	2110.9735	-5.24	0	71	8e-08	1	U	K.EFEFSPLEYVPGPDETQK.T
38896	47 – 64	1056.4921	2110.9696	2110.9735	-1.83	0	81	8.1e-09	1	U	K.EFEFSPLEYVPGPDETQK.T
9112	146 – 154	541.7542	1081.4938	1081.4927	0.95	0	58	1.7e-06	1	U	R.DAESYEQIK.F
9113	146 – 154	541.7544	1081.4942	1081.4927	1.40	0	60	9.8e-07	1	U	R.DAESYEQIK.F
9114	146 – 154	541.7549	1081.4952	1081.4927	2.30	0	60	1e-06	1	U	R.DAESYEQIK.F
9115	146 – 154	541.7550	1081.4954	1081.4927	2.45	0	53	4.8e-06	1	U	R.DAESYEQIK.F
10116	264 – 273	556.8080	1111.6015	1111.6026	-0.98	0	60	1e-06	1	U	R.WVVLAGPDQK.K

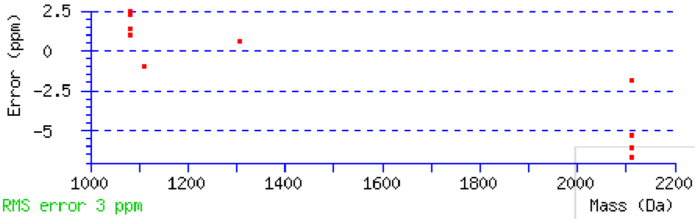

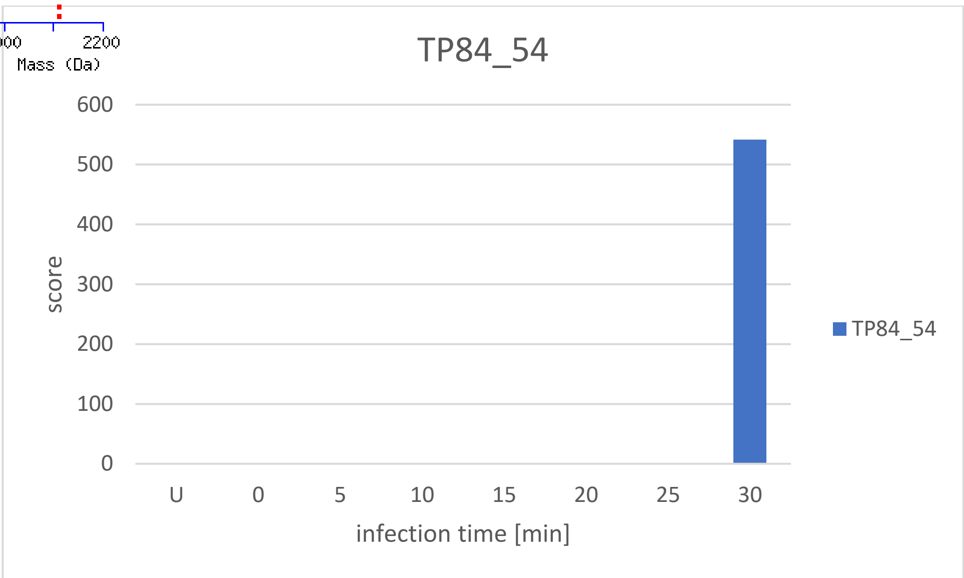

MASCOT Search Results

Protein View: TP84_56

Database: TP84
Score: 271
Nominal mass (M_r): 7416
Calculated pI: 9.25

Sequence similarity is available as **an NCBI BLAST search of 28. against nr**.

Search parameters

MS data file: O:\FA\02-luty2018\80202244zeb_czas20.raw
Enzyme: Trypsin: cuts C-term side of KR unless next residue is P.
Fixed modifications: **Carbamidomethyl (C)**
Variable modifications: **Oxidation (M)**

Protein sequence coverage: 50%

Matched peptides shown in ***bold red***.

```
 1 MEKKQKFNPI LSFQTDESVC EFIQKVMIKR MEFNRSNIVR EIFMIGLEEF
51 KKRHPEIDKK
```

Unformatted sequence string: **60 residues** (for pasting into other applications).

Sort peptides by ◉ Residue Number ○ Increasing Mass ○ Decreasing Mass

Show predicted peptides also

Query	Start – End	Observed	Mr(expt)	Mr(calc)	ppm	M	Score	Expect	Rank	U	Peptide
41727	7 – 25	1151.5592	2301.1038	2301.0987	2.22	0	73	5.5e-08	1	U	K.FNPILSFQTDESVCEFIQK.V
21210	41 – 51	678.3491	1354.6837	1354.6843	-0.43	0	53	4.7e-06	1	U	R.EIFMIGLEEFK.K
21211	41 – 51	678.3494	1354.6843	1354.6843	0.013	0	69	1.3e-07	1	U	R.EIFMIGLEEFK.K
21212	41 – 51	678.3499	1354.6852	1354.6843	0.69	0	62	7.1e-07	1	U	R.EIFMIGLEEFK.K
21213	41 – 51	678.3502	1354.6858	1354.6843	1.18	0	67	1.9e-07	1	U	R.EIFMIGLEEFK.K

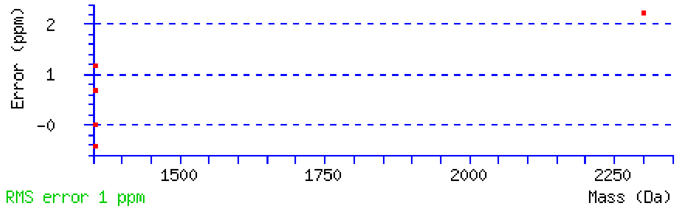

Mascot: **http://www.matrixscience.com/**

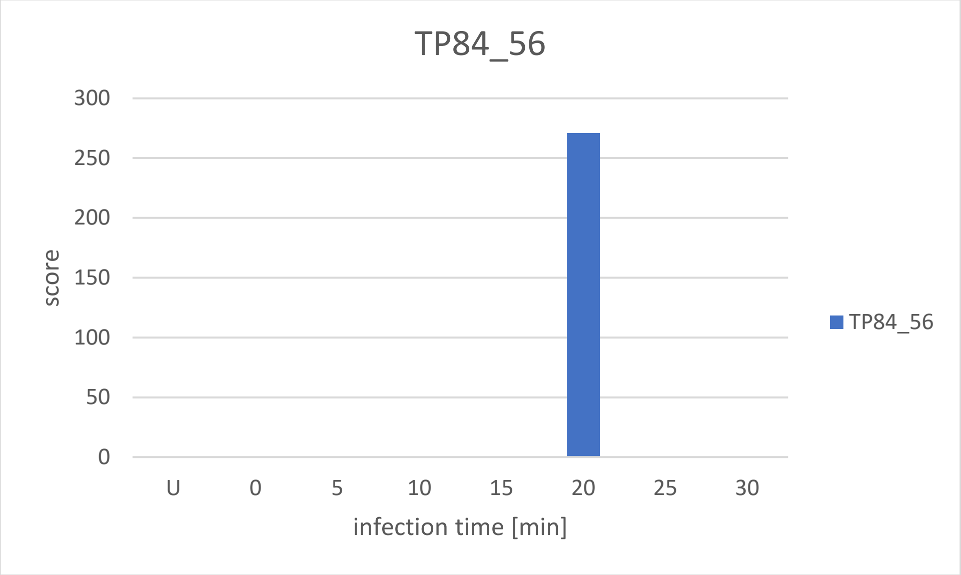

MASCOT Search Results

Protein View: TP84_57

**Database:**	TP84
**Score:**	338
**Nominal mass (M_r):**	30368
**Calculated pI:**	8.57

Sequence similarity is available as an NCBI BLAST search of 29. against nr.

Search parameters

**MS data file:**	O:\FA\02-luty2018\80202246zeb_czas30.raw
**Enzyme:**	Trypsin: cuts C-term side of KR unless next residue is P.
**Fixed modifications:**	Carbamidomethyl (C)
**Variable modifications:**	Oxidation (M)

Protein sequence coverage: 10%

Matched peptides shown in ***bold red***.

```
  1 MSDQEKQEYL MSDDMWVYGY SVIDNGVIYS KEITDSEFRT YCVIRSLVNE
 51 RKAVAWPSYE TIAELSGHSK RTAMRNVARL IELDLIEKRP RSGTSNEFVV
101 KKLQNSKVLK NKQDILDYIE KCRDDEPKKA TDPGEKVDKP EKADPIPYKE
151 IIDYLNEKAG TKYSHTGSAN QKLIKARWNE MAKINKDRDW IVAQFKHVID
201 VKTAQWKGTE WEKYLRPSTL FGNKFDQYRN ESPNHKPVGG QNRPKEDIRT
251 RLAGFLADED
```

Unformatted sequence string: 260 residues (for pasting into other applications).

Sort peptides by ◉ Residue Number ○ Increasing Mass ○ Decreasing Mass

Show predicted peptides also

Query	Start – End	Observed	Mr(expt)	Mr(calc)	ppm	M	Score	Expect	Rank	U	Peptide
36376	53 – 70	649.3261	1944.9563	1944.9581	-0.92	0	104	3.9e-11	1	U	K.AVAWPSYETIAELSGHSK.R
36377	53 – 70	649.3286	1944.9641	1944.9581	3.06	0	84	4.2e-09	1	U	K.AVAWPSYETIAELSGHSK.R
36378	53 – 70	649.3287	1944.9644	1944.9581	3.21	0	99	1.1e-10	1	U	K.AVAWPSYETIAELSGHSK.R
9242	80 – 88	543.3270	1084.6394	1084.6379	1.37	0	89	1.2e-09	1	U	R.LIELDLIEK.R

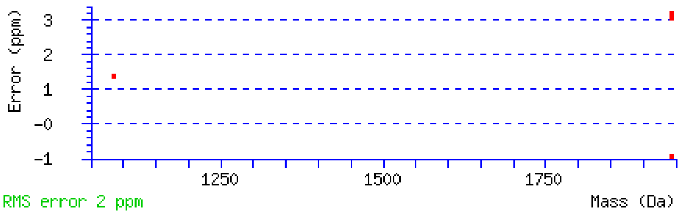

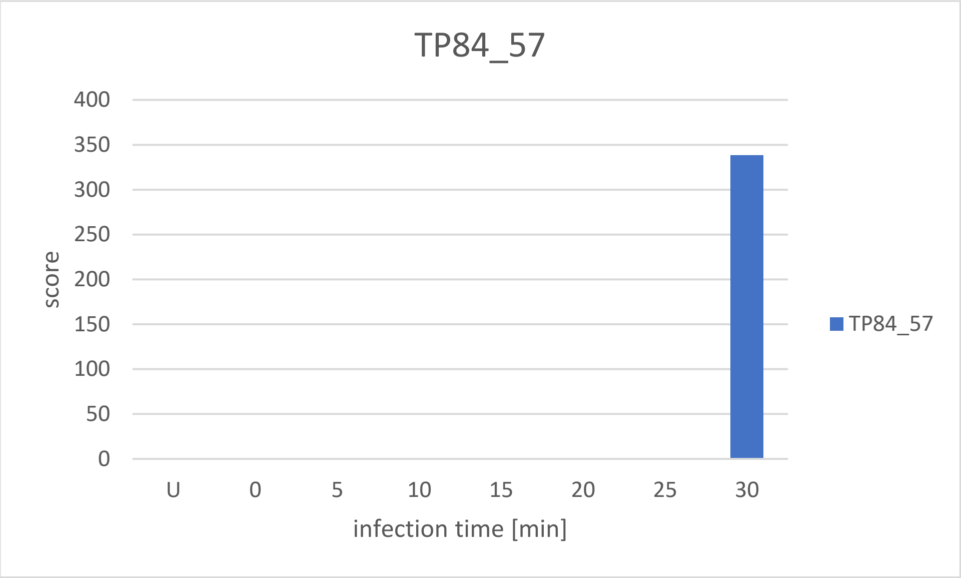

MASCOT Search Results

Protein View: TP84_63

**Database:**	TP84
**Score:**	357
**Nominal mass (M_r):**	17391
**Calculated pI:**	5.16

Sequence similarity is available as an NCBI BLAST search of 35. against nr.

Search parameters

**MS data file:**	O:\FA\02-luty2018\80202245zeb_czas25.raw
**Enzyme:**	Trypsin: cuts C-term side of KR unless next residue is P.
**Fixed modifications:**	Carbamidomethyl (C)
**Variable modifications:**	Oxidation (M)

Protein sequence coverage: 18%

Matched peptides shown in ***bold red***.

```
  1 MNNVTLVGRL TNDVELRYTQ QGKAVATFNL AVQREFKNQD NVYEVDFPQI
 51 VVWGKPAETL ANYTKKGSLI GITGRLQTRS YERQDGSRVY VTEVVANNVR
101 IYQWKDSGQG GQSRQGGSQP QGRGSRYPDP YGPYDGDPFA NDGEPIYVND
151 DDLPF
```

Unformatted sequence string: 155 residues (for pasting into other applications).

Sort peptides by ◉ Residue Number ○ Increasing Mass ○ Decreasing Mass

Show predicted peptides also

Query	Start – End	Observed	Mr(expt)	Mr(calc)	ppm	M	Score	Expect	Rank	U	Peptide
5004	1 – 9	502.2718	1002.5290	1002.5280	0.92	0	59	1.1e-06	1	U	-.MNNVTLVGR.L
5005	1 – 9	502.2718	1002.5291	1002.5280	1.08	0	60	1e-06	1	U	-.MNNVTLVGR.L
3934	10 – 17	480.2616	958.5087	958.5083	0.33	0	53	5.4e-06	1	U	R.LTNDVELR.Y
3936	10 – 17	480.2618	958.5091	958.5083	0.81	0	51	8.4e-06	1	U	R.LTNDVELR.Y
3938	10 – 17	480.2619	958.5092	958.5083	0.94	0	52	6.3e-06	1	U	R.LTNDVELR.Y
3939	10 – 17	480.2621	958.5097	958.5083	1.38	0	52	6.1e-06	1	U	R.LTNDVELR.Y
9300	24 – 34	595.3336	1188.6527	1188.6615	-7.38	0	50	9.6e-06	1	U	K.AVATFNLAVQR.E
9301	24 – 34	595.3390	1188.6634	1188.6615	1.62	0	71	8.8e-08	1	U	K.AVATFNLAVQR.E

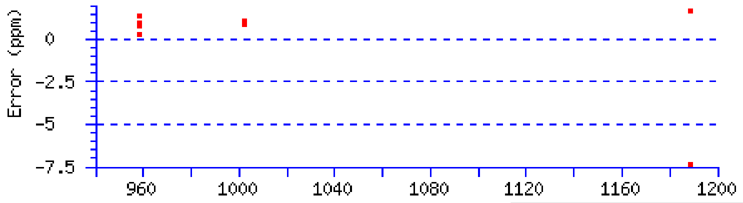

RMS error 2 ppm

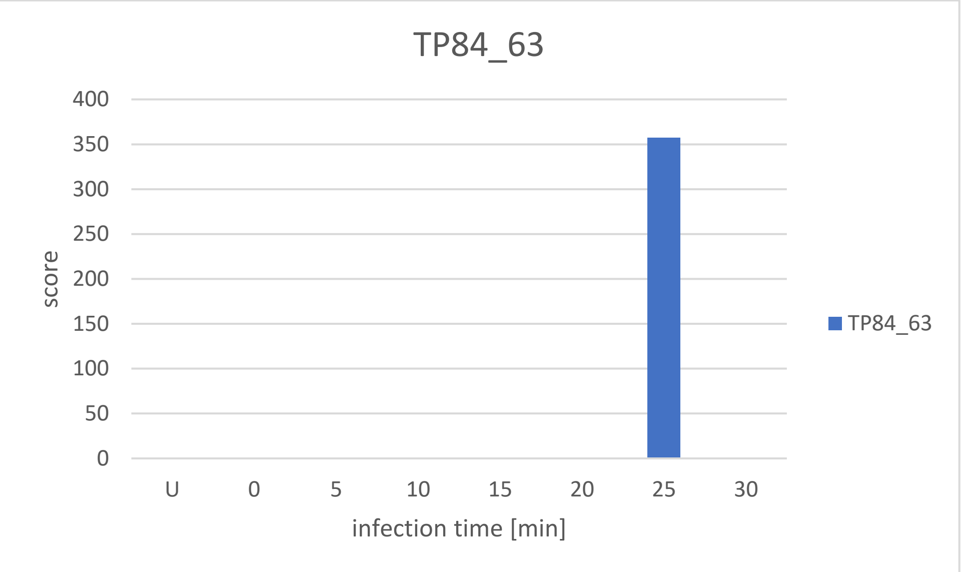

MASCOT Search Results

Protein View: TP84_64

Database: TP84
Score: 232
Nominal mass (M_r): 9330
Calculated pI: 6.89

Sequence similarity is available as **an NCBI BLAST search of 36. against nr**.

Search parameters

MS data file: O:\FA\02-luty2018\80202242zeb_czas10.raw
Enzyme: Trypsin: cuts C-term side of KR unless next residue is P.
Fixed modifications: Carbamidomethyl (C)
Variable modifications: Oxidation (M)

Protein sequence coverage: 27%

Matched peptides shown in ***bold red***.

```
 1 MVKYFVGSDV HFIAFDQNLF KHDKCFHIKR SDQIYSGITI YKVAGSQIAD
51 YLHDRIMNMY IKGDINLEVM PESLLYKTI
```

Unformatted sequence string: 79 residues (for pasting into other applications).

Sort peptides by ◉ Residue Number ○ Increasing Mass ○ Decreasing Mass

Show predicted peptides also

Query	Start - End	Observed	Mr(expt)	Mr(calc)	ppm	M	Score	Expect	Rank	U	Peptide
2335	56 - 62	456.7388	911.4631	911.4608	2.46	0	31	0.00089	1	U	R.IMNMYIK.G
19412	63 - 77	860.9433	1719.8720	1719.8753	-1.90	0	68	1.4e-07	1	U	K.GDINLEVMPESLLYK.T
19413	63 - 77	860.9444	1719.8743	1719.8753	-0.59	0	83	5.6e-09	1	U	K.GDINLEVMPESLLYK.T
19414	63 - 77	860.9450	1719.8754	1719.8753	0.069	0	89	1.2e-09	1	U	K.GDINLEVMPESLLYK.T

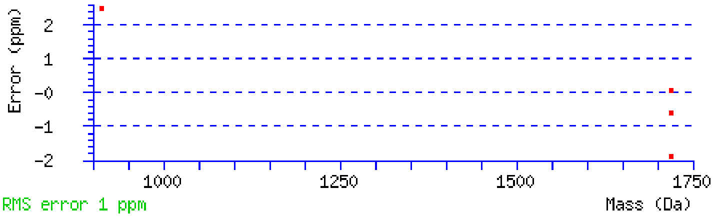

Mascot: http://www.matrixscience.com/

MASCOT Search Results

Protein View: TP84_64

**Database:**	TP84
**Score:**	200
**Nominal mass (M_r):**	9330
**Calculated pI:**	6.89

Sequence similarity is available as **an NCBI BLAST search of 36. against nr**.

Search parameters

**MS data file:**	O:\FA\02-luty2018\80202243zeb_czas15.raw
**Enzyme:**	Trypsin: cuts C-term side of KR unless next residue is P.
**Fixed modifications:**	Carbamidomethyl (C)
**Variable modifications:**	Oxidation (M)

Protein sequence coverage: 50%

Matched peptides shown in ***bold red***.

```
 1 MVKYFVGSDV HFIAFDQNLF KHDKCFHIKR SDQIYSGITI YKVAGSQIAD
51 YLHDRIMNMY IKGDINLEVM PESLLYKTI
```

Unformatted sequence string: **79 residues** (for pasting into other applications).

Sort peptides by ◉ Residue Number ○ Increasing Mass ○ Decreasing Mass

Show predicted peptides also

Query	Start - End	Observed	Mr(expt)	Mr(calc)	ppm	M	Score	Expect	Rank	U	Peptide
12268	31 - 42	694.3564	1386.6983	1386.7031	-3.43	0	22	0.0063	1	U	R.SDQIYSGITIYK.V
12269	31 - 42	694.3581	1386.7017	1386.7031	-1.00	0	14	0.043	1	U	R.SDQIYSGITIYK.V
13458	43 - 55	482.2434	1443.7085	1443.7106	-1.48	0	70	1e-07	1	U	K.VAGSQIADYLHDR.I
13459	43 - 55	482.2441	1443.7105	1443.7106	-0.069	0	51	8.1e-06	1	U	K.VAGSQIADYLHDR.I
19316	63 - 77	860.9434	1719.8722	1719.8753	-1.78	0	96	2.7e-10	1	U	K.GDINLEVMPESLLYK.T

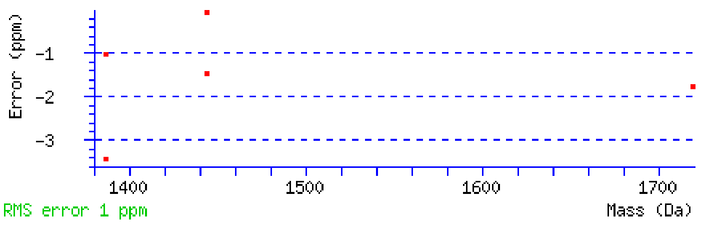

Mascot: http://www.matrixscience.com/

MASCOT Search Results

Protein View: TP84_64

Database: TP84
Score: 522
Nominal mass (M_r): 9330
Calculated pI: 6.89

Sequence similarity is available as an NCBI BLAST search of 36. against nr.

Search parameters

MS data file: O:\FA\02-luty2018\80202244zeb_czas20.raw
Enzyme: Trypsin: cuts C-term side of KR unless next residue is P.
Fixed modifications: Carbamidomethyl (C)
Variable modifications: Oxidation (M)

Protein sequence coverage: 58%

Matched peptides shown in ***bold red***.

```
 1 MVKYFVGSDV HFIAFDQNLF KHDKCFHIKR SDQIYSGITI YKVAGSQIAD
51 YLHDRIMNMY IKGDINLEVM PESLLYKTI
```

Unformatted sequence string: 79 residues (for pasting into other applications).

Sort peptides by ◉ Residue Number ○ Increasing Mass ○ Decreasing Mass

Show predicted peptides also

Query	Start – End	Observed	Mr(expt)	Mr(calc)	ppm	M	Score	Expect	Rank	U	Peptide
39718	4 – 21	716.3572	2146.0497	2146.0524	-1.25	0	59	1.4e-06	1	U	K.YFVGSDVHFIAFDQNLFK.H
24184	43 – 55	482.2466	1443.7179	1443.7106	5.04	0	69	1.4e-07	1	U	K.VAGSQIADYLHDR.I
24185	43 – 55	482.2466	1443.7181	1443.7106	5.15	0	73	4.7e-08	1	U	K.VAGSQIADYLHDR.I
24186	43 – 55	722.8665	1443.7184	1443.7106	5.39	0	122	5.6e-13	1	U	K.VAGSQIADYLHDR.I
24187	43 – 55	482.2468	1443.7186	1443.7106	5.54	0	79	1.3e-08	1	U	K.VAGSQIADYLHDR.I
24188	43 – 55	722.8670	1443.7194	1443.7106	6.09	0	116	2.3e-12	1	U	K.VAGSQIADYLHDR.I
32080	63 – 77	860.9463	1719.8780	1719.8753	1.57	0	82	6.7e-09	1	U	K.GDINLEVMPESLLYK.T

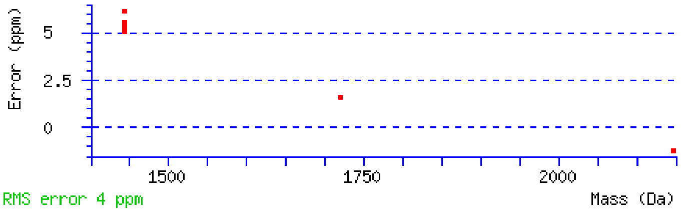

Mascot: http://www.matrixscience.com/

MASCOT Search Results

Protein View: TP84_64

Database: TP84
Score: 818
Nominal mass (M_r): 9330
Calculated pI: 6.89

Sequence similarity is available as an NCBI BLAST search of 36. against nr.

Search parameters

MS data file: O:\FA\02-luty2018\80202245zeb_czas25.raw
Enzyme: Trypsin: cuts C-term side of KR unless next residue is P.
Fixed modifications: Carbamidomethyl (C)
Variable modifications: Oxidation (M)

Protein sequence coverage: 58%

Matched peptides shown in ***bold red***.

```
 1 MVKYFVGSDV HFIAFDQNLF KHDKCFHIKR SDQIYSGITI YKVAGSQIAD
51 YLHDRIMNMY IKGDINLEVM PESLLYKTI
```

Unformatted sequence string: 79 residues (for pasting into other applications).

Sort peptides by ◉ Residue Number ○ Increasing Mass ○ Decreasing Mass

Show predicted peptides also

Query	Start – End	Observed	Mr(expt)	Mr(calc)	ppm	M	Score	Expect	Rank	U	Peptide
30268	4 – 21	1074.0378	2146.0610	2146.0524	4.03	0	107	2.1e-11	1	U	K.YFVGSDVHFIAFDQNLFK.H
16136	43 – 55	722.8606	1443.7067	1443.7106	-2.69	0	114	3.9e-12	1	U	K.VAGSQIADYLHDR.I
16137	43 – 55	722.8615	1443.7084	1443.7106	-1.51	0	114	4e-12	1	U	K.VAGSQIADYLHDR.I
16138	43 – 55	482.2435	1443.7087	1443.7106	-1.36	0	73	4.8e-08	1	U	K.VAGSQIADYLHDR.I
16139	43 – 55	482.2436	1443.7090	1443.7106	-1.11	0	74	3.8e-08	1	U	K.VAGSQIADYLHDR.I
22949	63 – 77	860.9448	1719.8751	1719.8753	-0.094	0	88	1.6e-09	1	U	K.GDINLEVMPESLLYK.T
22950	63 – 77	860.9450	1719.8755	1719.8753	0.14	0	86	2.3e-09	1	U	K.GDINLEVMPESLLYK.T
22951	63 – 77	860.9452	1719.8758	1719.8753	0.31	0	97	1.9e-10	1	U	K.GDINLEVMPESLLYK.T
22952	63 – 77	860.9458	1719.8771	1719.8753	1.08	0	93	5.4e-10	1	U	K.GDINLEVMPESLLYK.T
22953	63 – 77	860.9466	1719.8787	1719.8753	1.98	0	88	1.6e-09	1	U	K.GDINLEVMPESLLYK.T

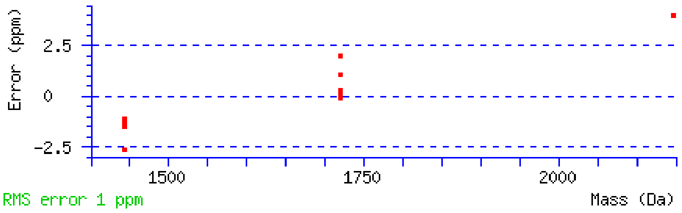

Mascot: http://www.matrixscience.com/

MASCOT Search Results

Protein View: TP84_64

**Database:**	TP84
**Score:**	474
**Nominal mass (M_r):**	9330
**Calculated pI:**	6.89

Sequence similarity is available as **an NCBI BLAST search of 36. against nr**.

Search parameters

**MS data file:**	O:\FA\02-luty2018\80202246zeb_czas30.raw
**Enzyme:**	Trypsin: cuts C-term side of KR unless next residue is P.
**Fixed modifications:**	Carbamidomethyl (C)
**Variable modifications:**	Oxidation (M)

Protein sequence coverage: 35%

Matched peptides shown in ***bold red***.

```
 1 MVKYFVGSDV HFIAFDQNLF KHDKCFHIKR SDQIYSGITI YKVAGSQIAD
51 YLHDRIMNMY IKGDINLEVM PESLLYKTI
```

Unformatted sequence string: **79 residues** (for pasting into other applications).

Sort peptides by ◉ Residue Number ○ Increasing Mass ○ Decreasing Mass

Show predicted peptides also

Query	Start – End	Observed	Mr(expt)	Mr(calc)	ppm	M	Score	Expect	Rank	U	Peptide
22659	43 – 55	722.8624	1443.7102	1443.7106	-0.29	0	130	9.9e-14	1	U	K.VAGSQIADYLHDR.I
22660	43 – 55	722.8631	1443.7117	1443.7106	0.76	0	119	1.2e-12	1	U	K.VAGSQIADYLHDR.I
22661	43 – 55	722.8636	1443.7125	1443.7106	1.33	0	118	1.7e-12	1	U	K.VAGSQIADYLHDR.I
31116	63 – 77	860.9450	1719.8755	1719.8753	0.10	0	89	1.1e-09	1	U	K.GDINLEVMPESLLYK.T
31117	63 – 77	860.9464	1719.8782	1719.8753	1.71	0	70	1e-07	1	U	K.GDINLEVMPESLLYK.T

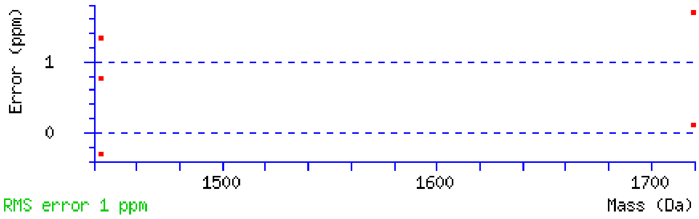

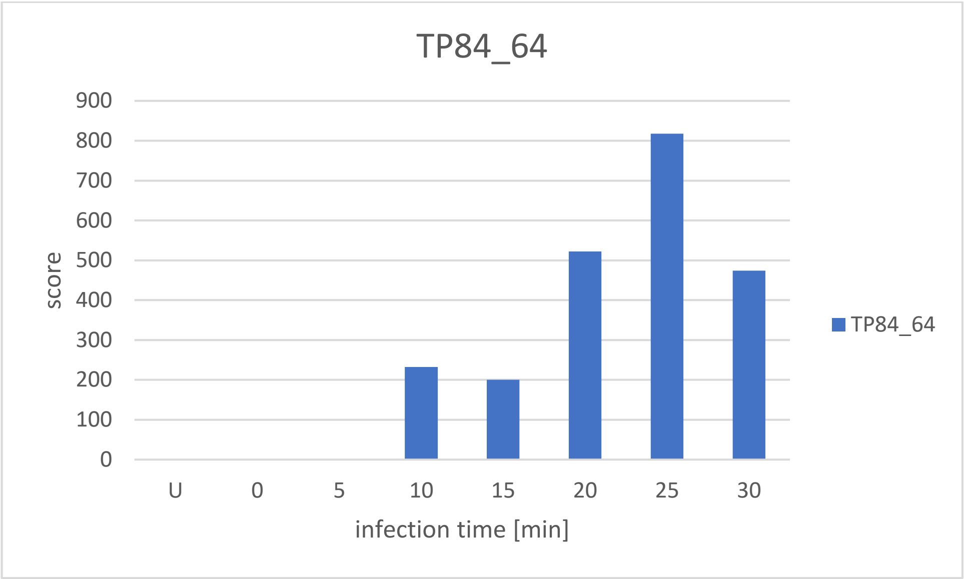

MASCOT Search Results

Protein View: TP84_66

Database: TP84
Score: 343
Nominal mass (M_r): 10393
Calculated pI: 5.92

Sequence similarity is available as an NCBI BLAST search of 38. against nr.

Search parameters

MS data file: O:\FA\02-luty2018\80202244zeb_czas20.raw
Enzyme: Trypsin: cuts C-term side of KR unless next residue is P.
Fixed modifications: Carbamidomethyl (C)
Variable modifications: Oxidation (M)

Protein sequence coverage: 22%

Matched peptides shown in ***bold red***.

```
 1 MDIREEIYQA QKEREEFEKA RAASMPDEVE RKIVELMNDP RIQAEVMAII
51 IHLKKQKRKD IEILTIGLMV TNMARSVMEQ AAQTKQKGE
```

Unformatted sequence string: 89 residues (for pasting into other applications).

Sort peptides by ◉ Residue Number ○ Increasing Mass ○ Decreasing Mass

Show predicted peptides also

Query	Start – End	Observed	Mr(expt)	Mr(calc)	ppm	M	Score	Expect	Rank	U	Peptide
11525	22 – 31	552.7569	1103.4993	1103.4917	6.90	0	67	2e-07	1	U	R.AASMPDEVER.K
11105	76 – 85	546.7746	1091.5346	1091.5281	5.95	0	58	1.5e-06	1	U	R.SVMEQAAQTK.Q
11106	76 – 85	546.7746	1091.5346	1091.5281	6.01	0	60	9.1e-07	1	U	R.SVMEQAAQTK.Q
11107	76 – 85	546.7746	1091.5347	1091.5281	6.03	0	61	8.7e-07	1	U	R.SVMEQAAQTK.Q
11109	76 – 85	546.7749	1091.5352	1091.5281	6.48	0	60	9.4e-07	1	U	R.SVMEQAAQTK.Q
11110	76 – 85	546.7749	1091.5352	1091.5281	6.50	0	61	8.6e-07	1	U	R.SVMEQAAQTK.Q
11111	76 – 85	546.7751	1091.5357	1091.5281	6.98	0	54	3.8e-06	1	U	R.SVMEQAAQTK.Q

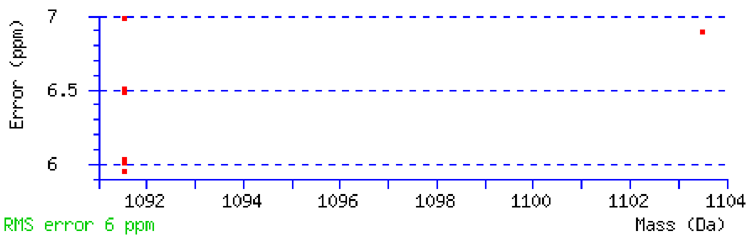

Mascot: http://www.matrixscience.com/

MASCOT Search Results

Protein View: TP84_66

Database: TP84
Score: 458
Nominal mass (M_r): 10393
Calculated pI: 5.92

Sequence similarity is available as an NCBI BLAST search of 38. against nr.

Search parameters

MS data file: O:\FA\02-luty2018\80202245zeb_czas25.raw
Enzyme: Trypsin: cuts C-term side of KR unless next residue is P.
Fixed modifications: Carbamidomethyl (C)
Variable modifications: Oxidation (M)

Protein sequence coverage: 22%

Matched peptides shown in ***bold red***.

```
 1 MDIREEIYQA QKEREEFEKA RAASMPDEVE RKIVELMNDP RIQAEVMAII
51 IHLKKQKRKD IEILTIGLMV TNMARSVMEQ AAQTKQKGE
```

Unformatted sequence string: 89 residues (for pasting into other applications).

Sort peptides by ◉ Residue Number ○ Increasing Mass ○ Decreasing Mass

Show predicted peptides also

Query	Start – End	Observed	Mr(expt)	Mr(calc)	ppm	M	Score	Expect	Rank	U	Peptide
7196	22 – 31	552.7536	1103.4927	1103.4917	0.90	0	88	1.5e-09	1	U	R.AASMPDEVER.K
7197	22 – 31	552.7538	1103.4931	1103.4917	1.26	0	77	2.1e-08	1	U	R.AASMPDEVER.K
7198	22 – 31	552.7553	1103.4960	1103.4917	3.94	0	80	9e-09	1	U	R.AASMPDEVER.K
6903	76 – 85	546.7717	1091.5289	1091.5281	0.79	0	77	2.1e-08	1	U	R.SVMEQAAQTK.Q
6904	76 – 85	546.7718	1091.5291	1091.5281	0.93	0	71	7.5e-08	1	U	R.SVMEQAAQTK.Q
6905	76 – 85	546.7719	1091.5292	1091.5281	1.06	0	71	7.3e-08	1	U	R.SVMEQAAQTK.Q
6906	76 – 85	546.7720	1091.5295	1091.5281	1.30	0	71	8e-08	1	U	R.SVMEQAAQTK.Q

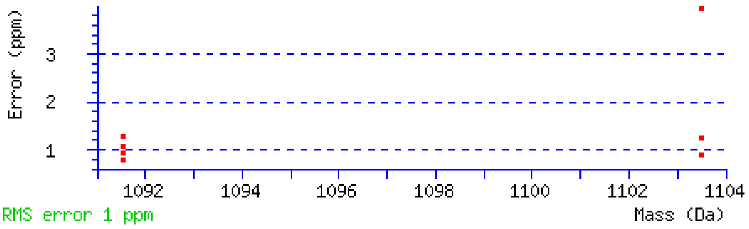

Mascot: http://www.matrixscience.com/

MASCOT Search Results

Protein View: TP84_66

Database: TP84
Score: 523
Nominal mass (M_r): 10393
Calculated pI: 5.92

Sequence similarity is available as an NCBI BLAST search of 38. against nr.

Search parameters

MS data file: O:\FA\02-luty2018\80202246zeb_czas30.raw
Enzyme: Trypsin: cuts C-term side of KR unless next residue is P.
Fixed modifications: Carbamidomethyl (C)
Variable modifications: Oxidation (M)

Protein sequence coverage: 22%

Matched peptides shown in ***bold red***.

```
 1 MDIREEIYQA QKEREEFEKA RAASMPDEVE RKIVELMNDP RIQAEVMAII
51 IHLKKQKRKD IEILTIGLMV TNMARSVMEQ AAQTKQKGE
```

Unformatted sequence string: 89 residues (for pasting into other applications).

Sort peptides by ◉ Residue Number ○ Increasing Mass ○ Decreasing Mass

Show predicted peptides also

Query	Start – End	Observed	Mr(expt)	Mr(calc)	ppm	M	Score	Expect	Rank	U	Peptide
9877	22 – 31	552.7536	1103.4926	1103.4917	0.86	0	83	5.1e-09	1	U	R.AASMPDEVER.K
9878	22 – 31	552.7537	1103.4928	1103.4917	1.04	0	84	3.7e-09	1	U	R.AASMPDEVER.K
9879	22 – 31	552.7540	1103.4933	1103.4917	1.49	0	84	3.7e-09	1	U	R.AASMPDEVER.K
9880	22 – 31	552.7541	1103.4937	1103.4917	1.84	0	76	2.5e-08	1	U	R.AASMPDEVER.K
9511	76 – 85	546.7715	1091.5285	1091.5281	0.36	0	58	1.6e-06	1	U	R.SVMEQAAQTK.Q
9512	76 – 85	546.7717	1091.5289	1091.5281	0.79	0	78	1.6e-08	1	U	R.SVMEQAAQTK.Q
9513	76 – 85	546.7718	1091.5290	1091.5281	0.88	0	71	8.8e-08	1	U	R.SVMEQAAQTK.Q
9514	76 – 85	546.7723	1091.5301	1091.5281	1.87	0	80	9.6e-09	1	U	R.SVMEQAAQTK.Q

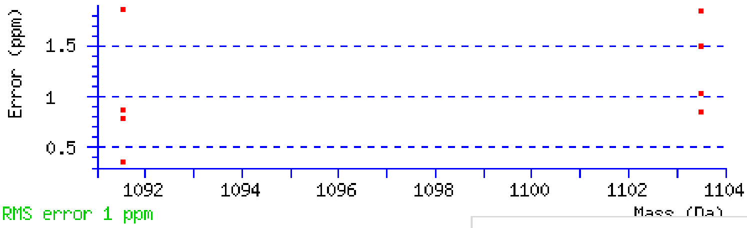

RMS error 1 ppm

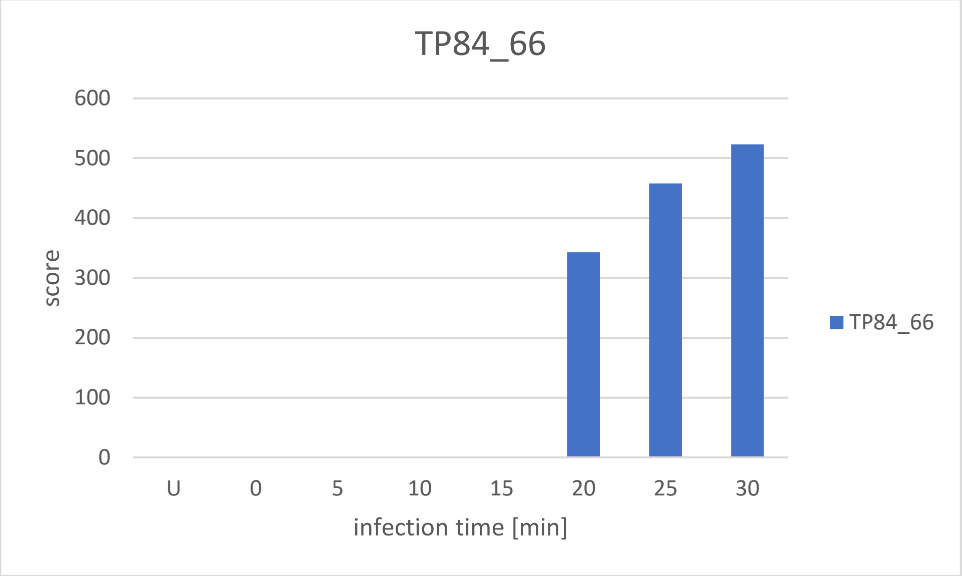

MASCOT Search Results

Protein View: TP84_68

**Database:**	TP84
**Score:**	715
**Nominal mass (M_r):**	38540
**Calculated pI:**	6.91

Sequence similarity is available as **an NCBI BLAST search of 40. against nr**.

Search parameters

**MS data file:**	O:\FA\02-luty2018\80202246zeb_czas30.raw
**Enzyme:**	Trypsin: cuts C-term side of KR unless next residue is P.
**Fixed modifications:**	Carbamidomethyl (C)
**Variable modifications:**	Oxidation (M)

Protein sequence coverage: 27%

Matched peptides shown in ***bold red***.

```
  1 MEFKLYKVAG YEEAIMSLRM SKGKYFSWER AKKIQHLVYA VTDHRGFIAP
 51 PQVYINNMRN LAAIEEDIKV DHQGKKGEIS GNYIRDVDEF KRLLALTLNN
101 AMGEHKHHTL MKYIDISFFT IGLHRGAQDD LDAHAIAFNN RITRYSTRLA
151 NIQETVLSEW YQDKIIPFEH AQHFVGIEWP MVIETDIGNF EYTPFGYIHE
201 NFNRISDENG LKKDVKRGLI PLSMASNALW KIDLFNLRYV YKMRSKLTKA
251 NPELKQGMEM LADQIEKHVP VFGPYFRYEL TDTGEWEHMN KVKTVTREEY
301 ELLKKIKKQM QQQGTVDVDL YRLQQEGE
```

Unformatted sequence string: **328 residues** (for pasting into other applications).

Sort peptides by ◉ Residue Number ○ Increasing Mass ○ Decreasing Mass

Show predicted peptides also

Query	Start – End	Observed	Mr(expt)	Mr(calc)	ppm	M	Score	Expect	Rank	U	Peptide
18939	8 – 19	669.8384	1337.6622	1337.6649	-1.98	0	111	7.9e-12	1	U	K.VAGYEEAIMSLR.M
18940	8 – 19	669.8388	1337.6631	1337.6649	-1.37	0	77	1.9e-08	1	U	K.VAGYEEAIMSLR.M
22934	34 – 45	363.6988	1450.7662	1450.7681	-1.30	0	52	6.7e-06	1	U	K.IQHLVYAVTDHR.G
22935	34 – 45	726.3913	1450.7680	1450.7681	-0.034	0	86	2.3e-09	1	U	K.IQHLVYAVTDHR.G
22936	34 – 45	726.3917	1450.7688	1450.7681	0.50	0	82	6.8e-09	1	U	K.IQHLVYAVTDHR.G
22938	34 – 45	484.5971	1450.7694	1450.7681	0.89	0	64	4.2e-07	1	U	K.IQHLVYAVTDHR.G
27979	46 – 59	810.4247	1618.8348	1618.8290	3.59	0	54	3.9e-06	1	U	R.GFIAPPQVYINNMR.N
25364	93 – 106	508.9451	1523.8136	1523.8130	0.40	0	64	3.9e-07	1	U	R.LLALTLNNAMGEHK.H
31273	126 – 141	864.4094	1726.8042	1726.8023	1.10	0	142	6.3e-15	1	U	R.GAQDDLDAHAIAFNNR.I
36289	149 – 164	646.3260	1935.9563	1935.9578	-0.77	0	61	8.1e-07	1	U	R.LANIQETVLSEWYQDK.I
3139	232 – 238	445.7584	889.5022	889.5021	0.10	0	52	6.2e-06	1	U	K.IDLFNLR.Y

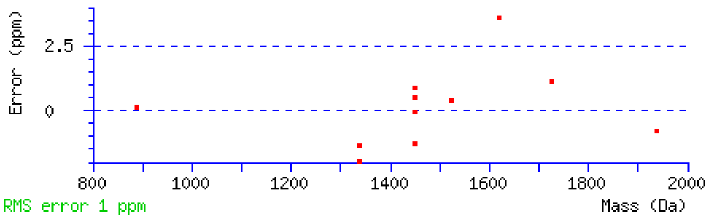

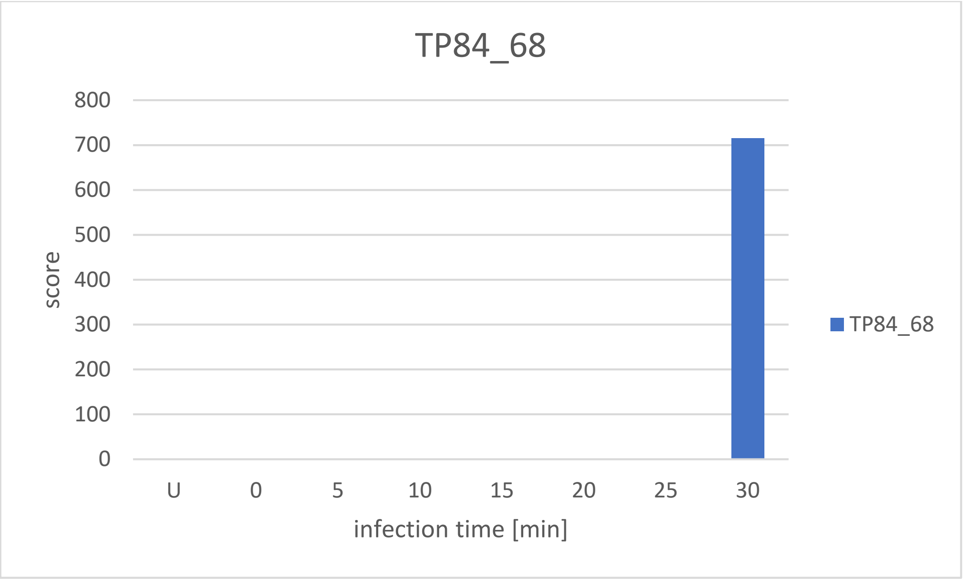
TP84_68
800
700
600
500
400
300
200
100
0
score
U
0
5
10
15
20
25
30
infection time [min]
TP84_68

MASCOT Search Results

Protein View: TP84_69

**Database:**	TP84
**Score:**	878
**Nominal mass (M_r):**	20065
**Calculated pI:**	5.67

Sequence similarity is available as **an NCBI BLAST search of 41. against nr**.

Search parameters

**MS data file:**	O:\FA\02-luty2018\80202246zeb_czas30.raw
**Enzyme:**	Trypsin: cuts C-term side of KR unless next residue is P.
**Fixed modifications:**	**Carbamidomethyl (C)**
**Variable modifications:**	**Oxidation (M)**

Protein sequence coverage: 15%

Matched peptides shown in ***bold red***.

```
  1 MVKVDERTIN LSDLFQKQKM LTDYVWQKHN IKKADLEKEK AAIICELWET
 51 ANELKSDGFK YWTDKKCDRE KTLEEIVDML HFYLQIGNIL GVVYEHYWIE
101 RRDTILDQIM AINWSLLMMD GPLTWAVSFA QYRGLVRMLG FDWDQDIIPA
151 YNRKFQENIA RQQRGY
```

Unformatted sequence string: **166 residues** (for pasting into other applications).

Sort peptides by ◉ Residue Number ○ Increasing Mass ○ Decreasing Mass

Show predicted peptides also

Query	Start – End	Observed	Mr(expt)	Mr(calc)	ppm	M	Score	Expect	Rank	U	Peptide
12657	8 – 17	589.8257	1177.6368	1177.6343	2.12	0	52	5.6e-06	1	U	R.TINLSDLFQK.Q
12658	8 – 17	589.8260	1177.6374	1177.6343	2.62	0	69	1.4e-07	1	U	R.TINLSDLFQK.Q
12659	8 – 17	589.8261	1177.6376	1177.6343	2.79	0	63	5.3e-07	1	U	R.TINLSDLFQK.Q
12660	8 – 17	589.8262	1177.6379	1177.6343	3.12	0	63	5.3e-07	1	U	R.TINLSDLFQK.Q
36468	138 – 153	977.4587	1952.9028	1952.9091	-3.23	0	128	1.6e-13	1	U	R.MLGFDWDQDIIPAYNR.K
36469	138 – 153	977.4587	1952.9029	1952.9091	-3.15	0	113	5.2e-12	1	U	R.MLGFDWDQDIIPAYNR.K
36470	138 – 153	977.4591	1952.9036	1952.9091	-2.82	0	130	9.2e-14	1	U	R.MLGFDWDQDIIPAYNR.K
36471	138 – 153	977.4598	1952.9051	1952.9091	-2.01	0	134	4.1e-14	1	U	R.MLGFDWDQDIIPAYNR.K
36472	138 – 153	977.4612	1952.9078	1952.9091	-0.64	0	128	1.6e-13	1	U	R.MLGFDWDQDIIPAYNR.K
36473	138 – 153	977.4613	1952.9081	1952.9091	-0.51	0	115	3.1e-12	1	U	R.MLGFDWDQDIIPAYNR.K

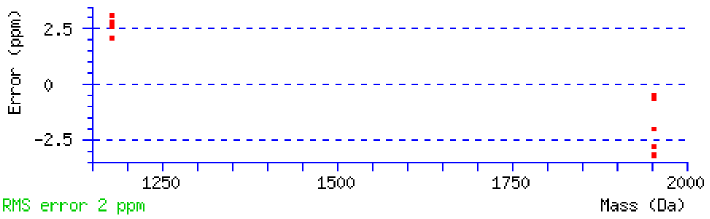

RMS error 2 ppm

Mascot: http://www.matrixscience.com/

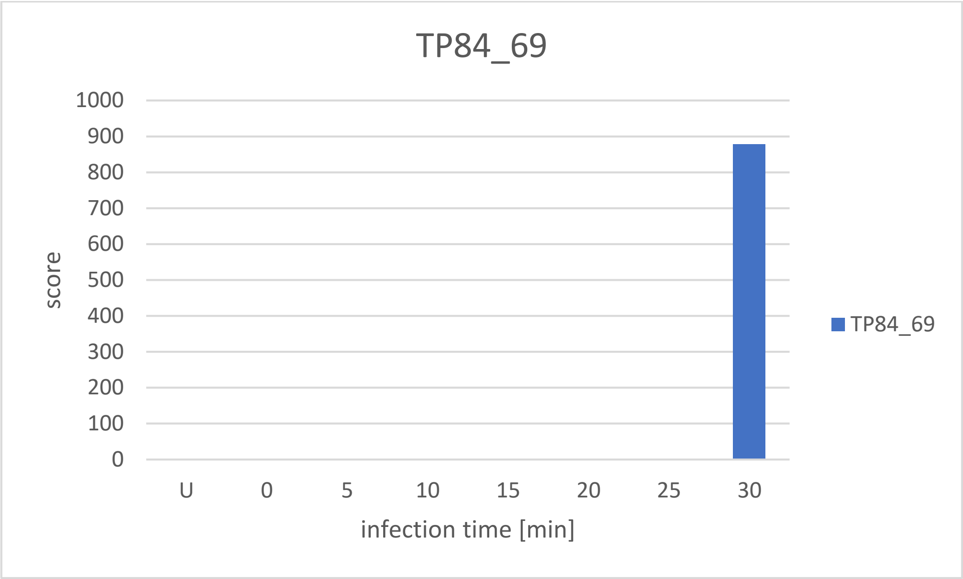

TP84_69
1000
900
800
700
600
500
400
300
200
100
0
score
U
0
5
10
15
20
25
30
infection time [min]
TP84_69

MASCOT Search Results

Protein View: TP84_79

Database: TP84
Score: 329
Nominal mass (M_r): 13449
Calculated pI: 7.82

Sequence similarity is available as an NCBI BLAST search of 52. against nr.

Search parameters

MS data file: O:\FA\02-luty2018\80202243zeb_czas15.raw
Enzyme: Trypsin: cuts C-term side of KR unless next residue is P.
Fixed modifications: Carbamidomethyl (C)
Variable modifications: Oxidation (M)

Protein sequence coverage: 23%

Matched peptides shown in ***bold red***.

```
  1 MAKTTTQFQY HSGVGDATSY IHTSEDYMNV YVTVELGRVG TWETEAWCKL
 51 ALQRYENGAW KTIATAQGYA ATGQNLNRTF SNISNVMEKP MRVKVDLYAN
101 SSYSDYVQTV YTKQWIR
```

Unformatted sequence string: 117 residues (for pasting into other applications).

Sort peptides by ◉ Residue Number ○ Increasing Mass ○ Decreasing Mass

Show predicted peptides also

Query	Start – End	Observed	Mr(expt)	Mr(calc)	ppm	M	Score	Expect	Rank	U	Peptide
11679	39 – 49	683.8082	1365.6018	1365.6023	-0.36	0	80	1.1e-08	1	U	R.VGTWETEAWCK.L
19787	62 – 78	583.9655	1748.8746	1748.8805	-3.42	0	60	9.8e-07	1	U	K.TIATAQGYAATGQNLNR.T
19788	62 – 78	875.4461	1748.8776	1748.8805	-1.70	0	23	0.0055	1	U	K.TIATAQGYAATGQNLNR.T
19789	62 – 78	583.9668	1748.8785	1748.8805	-1.16	0	61	8.1e-07	1	U	K.TIATAQGYAATGQNLNR.T
19790	62 – 78	875.4488	1748.8830	1748.8805	1.42	0	157	1.8e-16	1	U	K.TIATAQGYAATGQNLNR.T

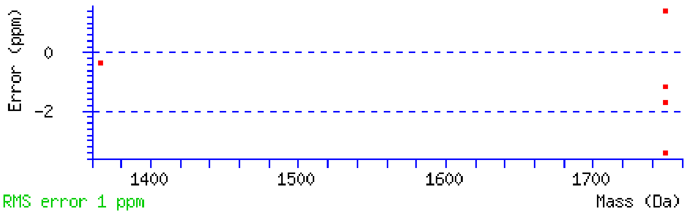

Mascot: http://www.matrixscience.com/

MASCOT Search Results

Protein View: TP84_79

**Database:**	TP84
**Score:**	1140
**Nominal mass (M_r):**	13449
**Calculated pI:**	7.82

Sequence similarity is available as **an NCBI BLAST search of 52. against nr**.

Search parameters

**MS data file:**	O:\FA\02-luty2018\80202244zeb_czas20.raw
**Enzyme:**	Trypsin: cuts C-term side of KR unless next residue is P.
**Fixed modifications:**	**Carbamidomethyl (C)**
**Variable modifications:**	**Oxidation (M)**

Protein sequence coverage: 26%

Matched peptides shown in ***bold red***.

```
  1 MAKTTTQFQY HSGVGDATSY IHTSEDYMNV YVTVELGRVG TWETEAWCKL
 51 ALQRYENGAW KTIATAQGYA ATGQNLNRTF SNISNVMEKP MRVKVDLYAN
101 SSYSDYVQTV YTKQWIR
```

Unformatted sequence string: **117 residues** (for pasting into other applications).

Sort peptides by ◉ Residue Number ○ Increasing Mass ○ Decreasing Mass

Show predicted peptides also

Query	Start – End	Observed	Mr(expt)	Mr(calc)	ppm	M	Score	Expect	Rank	U	Peptide
32668	62 – 78	875.4505	1748.8865	1748.8805	3.40	0	149	1.2e-15	1	U	K.TIATAQGYAATGQNLNR.T
32669	62 – 78	875.4509	1748.8873	1748.8805	3.85	0	155	3.2e-16	1	U	K.TIATAQGYAATGQNLNR.T
32670	62 – 78	583.9697	1748.8874	1748.8805	3.90	0	67	2.2e-07	1	U	K.TIATAQGYAATGQNLNR.T
32671	62 – 78	875.4511	1748.8875	1748.8805	4.01	0	161	8.7e-17	1	U	K.TIATAQGYAATGQNLNR.T
32672	62 – 78	583.9701	1748.8886	1748.8805	4.62	0	63	5.5e-07	1	U	K.TIATAQGYAATGQNLNR.T
32675	62 – 78	583.9704	1748.8893	1748.8805	5.02	0	51	8e-06	1	U	K.TIATAQGYAATGQNLNR.T
32676	62 – 78	583.9704	1748.8895	1748.8805	5.10	0	70	1e-07	1	U	K.TIATAQGYAATGQNLNR.T
32678	62 – 78	875.4531	1748.8916	1748.8805	6.35	0	145	2.9e-15	1	U	K.TIATAQGYAATGQNLNR.T
29974	79 – 92	827.4096	1652.8046	1652.8014	1.93	0	97	1.9e-10	1	U	R.TFSNISNVMEKPMR.V
29975	79 – 92	551.9442	1652.8107	1652.8014	5.60	0	75	3.5e-08	1	U	R.TFSNISNVMEKPMR.V
29976	79 – 92	551.9443	1652.8112	1652.8014	5.93	0	79	1.2e-08	1	U	R.TFSNISNVMEKPMR.V
29977	79 – 92	827.4134	1652.8123	1652.8014	6.60	0	76	2.7e-08	1	U	R.TFSNISNVMEKPMR.V
30414	79 – 92	557.2755	1668.8047	1668.7963	5.03	0	60	9.9e-07	1	U	R.TFSNISNVMEKPMR.V + Oxidation (M)
30415	79 – 92	557.2762	1668.8067	1668.7963	6.21	0	62	5.8e-07	1	U	R.TFSNISNVMEKPMR.V + Oxidation (M)

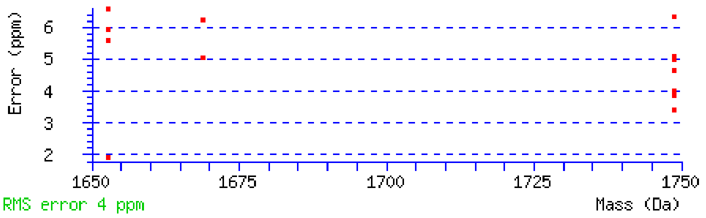

Mascot: **http://www.matrixscience.com/**

MASCOT Search Results

Protein View: TP84_79

Database: TP84
Score: 2462
Nominal mass (M_r): 13449
Calculated pI: 7.82

Sequence similarity is available as **an NCBI BLAST search of 52. against nr**.

Search parameters

MS data file: O:\FA\02-luty2018\80202245zeb_czas25.raw
Enzyme: Trypsin: cuts C-term side of KR unless next residue is P.
Fixed modifications: Carbamidomethyl (C)
Variable modifications: Oxidation (M)

Protein sequence coverage: 52%

Matched peptides shown in ***bold red***.

```
  1 MAKTTTQFQY HSGVGDATSY IHTSEDYMNV YVTVELGRVG TWETEAWCKL
 51 ALQRYENGAW KTIATAQGYA ATGQNLNRTF SNISNVMEKP MRVKVDLYAN
101 SSYSDYVQTV YTKQWIR
```

Unformatted sequence string: **117 residues** (for pasting into other applications).

Sort peptides by ◉ Residue Number ○ Increasing Mass ○ Decreasing Mass

Show predicted peptides also

Query	Start – End	Observed	Mr(expt)	Mr(calc)	ppm	M	Score	Expect	Rank	U	Peptide
14118	39 – 49	683.8071	1365.5996	1365.6023	-1.97	0	90	9.8e-10	1	U	R.VGTWETEAWCK.L
14119	39 – 49	683.8074	1365.6002	1365.6023	-1.56	0	95	3.2e-10	1	U	R.VGTWETEAWCK.L
14120	39 – 49	683.8076	1365.6007	1365.6023	-1.23	0	94	4e-10	1	U	R.VGTWETEAWCK.L
14121	39 – 49	683.8086	1365.6026	1365.6023	0.22	0	88	1.8e-09	1	U	R.VGTWETEAWCK.L
23493	62 – 78	875.4418	1748.8690	1748.8805	-6.62	0	113	5.6e-12	1	U	K.TIATAQGYAATGQNLNR.T
23495	62 – 78	583.9655	1748.8746	1748.8805	-3.42	0	58	1.6e-06	1	U	K.TIATAQGYAATGQNLNR.T
23496	62 – 78	875.4455	1748.8764	1748.8805	-2.37	0	112	5.9e-12	1	U	K.TIATAQGYAATGQNLNR.T
23497	62 – 78	875.4459	1748.8773	1748.8805	-1.85	0	93	5e-10	1	U	K.TIATAQGYAATGQNLNR.T
23498	62 – 78	583.9665	1748.8776	1748.8805	-1.67	0	68	1.6e-07	1	U	K.TIATAQGYAATGQNLNR.T
23499	62 – 78	875.4467	1748.8789	1748.8805	-0.91	0	152	6.7e-16	1	U	K.TIATAQGYAATGQNLNR.T
23500	62 – 78	875.4469	1748.8792	1748.8805	-0.74	0	152	6.5e-16	1	U	K.TIATAQGYAATGQNLNR.T
23501	62 – 78	875.4470	1748.8794	1748.8805	-0.64	0	158	1.6e-16	1	U	K.TIATAQGYAATGQNLNR.T
23502	62 – 78	875.4472	1748.8798	1748.8805	-0.42	0	161	7.7e-17	1	U	K.TIATAQGYAATGQNLNR.T
23503	62 – 78	875.4472	1748.8798	1748.8805	-0.42	0	161	8.4e-17	1	U	K.TIATAQGYAATGQNLNR.T
23504	62 – 78	875.4475	1748.8804	1748.8805	-0.098	0	153	5.2e-16	1	U	K.TIATAQGYAATGQNLNR.T
23505	62 – 78	583.9674	1748.8804	1748.8805	-0.061	0	90	1e-09	1	U	K.TIATAQGYAATGQNLNR.T
23506	62 – 78	583.9674	1748.8805	1748.8805	-0.027	0	78	1.6e-08	1	U	K.TIATAQGYAATGQNLNR.T
23507	62 – 78	875.4476	1748.8807	1748.8805	0.074	0	154	3.6e-16	1	U	K.TIATAQGYAATGQNLNR.T
23508	62 – 78	583.9677	1748.8814	1748.8805	0.49	0	88	1.7e-09	1	U	K.TIATAQGYAATGQNLNR.T
23509	62 – 78	583.9678	1748.8817	1748.8805	0.68	0	83	5.1e-09	1	U	K.TIATAQGYAATGQNLNR.T
23511	62 – 78	583.9680	1748.8823	1748.8805	0.99	0	83	4.7e-09	1	U	K.TIATAQGYAATGQNLNR.T
21073	79 – 92	827.4067	1652.7989	1652.8014	-1.50	0	103	5.4e-11	1	U	R.TFSNISNVMEKPMR.V
21074	79 – 92	827.4070	1652.7994	1652.8014	-1.22	0	113	5e-12	1	U	R.TFSNISNVMEKPMR.V
21075	79 – 92	551.9417	1652.8033	1652.8014	1.16	0	90	9.5e-10	1	U	R.TFSNISNVMEKPMR.V
21462	79 – 92	557.2732	1668.7977	1668.7963	0.82	0	61	7.5e-07	1	U	R.TFSNISNVMEKPMR.V + Oxidation (M)
31052	95 – 113	1108.5281	2215.0416	2215.0321	4.32	0	97	2.1e-10	1	U	K.VDLYANSSYSDYVQTVYTK.Q

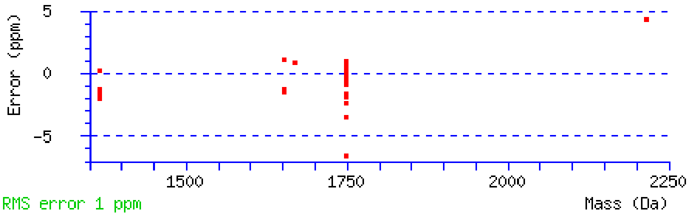

Mascot: http://www.matrixscience.com/

MASCOT Search Results

Protein View: TP84_79

Database: TP84
Score: 3398
Nominal mass (M_r): 13449
Calculated pI: 7.82

Sequence similarity is available as **an NCBI BLAST search of 52. against nr**.

Search parameters

MS data file: O:\FA\02-luty2018\80202246zeb_czas30.raw
Enzyme: Trypsin: cuts C-term side of KR unless next residue is P.
Fixed modifications: Carbamidomethyl (C)
Variable modifications: Oxidation (M)

Protein sequence coverage: 58%

Matched peptides shown in ***bold red***.

```
  1 MAKTTTQFQY HSGVGDATSY IHTSEDYMNV YVTVELGRVG TWETEAWCKL
 51 ALQRYENGAW KTIATAQGYA ATGQNLNRTF SNISNVMEKP MRVKVDLYAN
101 SSYSDYVQTV YTKQWIR
```

Unformatted sequence string: **117 residues** (for pasting into other applications).

Sort peptides by ◉ Residue Number ○ Increasing Mass ○ Decreasing Mass

Show predicted peptides also

Query	Start – End	Observed	Mr(expt)	Mr(calc)	ppm	M	Score	Expect	Rank	U	Peptide
19924	39 – 49	683.8054	1365.5963	1365.6023	-4.39	0	81	8.8e-09	1	U	R.VGTWETEAWCK.L
19925	39 – 49	683.8055	1365.5964	1365.6023	-4.33	0	80	9.5e-09	1	U	R.VGTWETEAWCK.L
19926	39 – 49	683.8060	1365.5975	1365.6023	-3.52	0	80	9.6e-09	1	U	R.VGTWETEAWCK.L
19927	39 – 49	683.8066	1365.5987	1365.6023	-2.66	0	84	4.3e-09	1	U	R.VGTWETEAWCK.L
19928	39 – 49	683.8069	1365.5992	1365.6023	-2.31	0	83	4.6e-09	1	U	R.VGTWETEAWCK.L
19929	39 – 49	683.8071	1365.5995	1365.6023	-2.05	0	94	4.2e-10	1	U	R.VGTWETEAWCK.L
19930	39 – 49	683.8072	1365.5998	1365.6023	-1.87	0	94	4.1e-10	1	U	R.VGTWETEAWCK.L
19931	39 – 49	683.8073	1365.6001	1365.6023	-1.66	0	87	1.8e-09	1	U	R.VGTWETEAWCK.L
19932	39 – 49	683.8073	1365.6001	1365.6023	-1.65	0	84	4.1e-09	1	U	R.VGTWETEAWCK.L
19933	39 – 49	683.8075	1365.6005	1365.6023	-1.37	0	78	1.8e-08	1	U	R.VGTWETEAWCK.L
2584	55 – 61	434.2032	866.3919	866.3922	-0.39	0	56	2.4e-06	1	U	R.YENGAWK.T
31814	62 – 78	875.4442	1748.8739	1748.8805	-3.80	0	62	6e-07	1	U	K.TIATAQGYAATGQNLNR.T
31816	62 – 78	875.4449	1748.8753	1748.8805	-2.99	0	112	6.6e-12	1	U	K.TIATAQGYAATGQNLNR.T
31817	62 – 78	875.4455	1748.8764	1748.8805	-2.38	0	78	1.6e-08	1	U	K.TIATAQGYAATGQNLNR.T
31818	62 – 78	875.4460	1748.8775	1748.8805	-1.73	0	155	3.4e-16	1	U	K.TIATAQGYAATGQNLNR.T
31819	62 – 78	875.4461	1748.8777	1748.8805	-1.60	0	148	1.5e-15	1	U	K.TIATAQGYAATGQNLNR.T
31820	62 – 78	875.4462	1748.8778	1748.8805	-1.57	0	152	6.6e-16	1	U	K.TIATAQGYAATGQNLNR.T
31821	62 – 78	875.4464	1748.8781	1748.8805	-1.37	0	157	1.9e-16	1	U	K.TIATAQGYAATGQNLNR.T
31822	62 – 78	875.4464	1748.8782	1748.8805	-1.34	0	161	8.9e-17	1	U	K.TIATAQGYAATGQNLNR.T
31823	62 – 78	875.4464	1748.8783	1748.8805	-1.26	0	131	7.1e-14	1	U	K.TIATAQGYAATGQNLNR.T
31824	62 – 78	583.9669	1748.8788	1748.8805	-0.97	0	56	2.6e-06	1	U	K.TIATAQGYAATGQNLNR.T
31825	62 – 78	875.4468	1748.8790	1748.8805	-0.90	0	155	3.3e-16	1	U	K.TIATAQGYAATGQNLNR.T
31826	62 – 78	875.4470	1748.8794	1748.8805	-0.65	0	142	7e-15	1	U	K.TIATAQGYAATGQNLNR.T
31827	62 – 78	583.9674	1748.8804	1748.8805	-0.078	0	97	1.8e-10	1	U	K.TIATAQGYAATGQNLNR.T
31828	62 – 78	583.9674	1748.8805	1748.8805	-0.0097	0	93	5.2e-10	1	U	K.TIATAQGYAATGQNLNR.T
31829	62 – 78	875.4476	1748.8806	1748.8805	0.028	0	164	3.9e-17	1	U	K.TIATAQGYAATGQNLNR.T
31830	62 – 78	583.9679	1748.8818	1748.8805	0.71	0	86	2.6e-09	1	U	K.TIATAQGYAATGQNLNR.T
31831	62 – 78	583.9680	1748.8822	1748.8805	0.95	0	66	2.3e-07	1	U	K.TIATAQGYAATGQNLNR.T
28828	79 – 92	827.4054	1652.7963	1652.8014	-3.09	0	106	2.4e-11	1	U	R.TFSNISNVMEKPMR.V
28829	79 – 92	827.4055	1652.7965	1652.8014	-3.00	0	105	3.5e-11	1	U	R.TFSNISNVMEKPMR.V
28830	79 – 92	827.4057	1652.7968	1652.8014	-2.80	0	107	2.1e-11	1	U	R.TFSNISNVMEKPMR.V
28831	79 – 92	827.4061	1652.7977	1652.8014	-2.26	0	106	2.7e-11	1	U	R.TFSNISNVMEKPMR.V
28832	79 – 92	827.4072	1652.7998	1652.8014	-0.96	0	103	5.6e-11	1	U	R.TFSNISNVMEKPMR.V
28833	79 – 92	551.9408	1652.8005	1652.8014	-0.59	0	86	2.4e-09	1	U	R.TFSNISNVMEKPMR.V
28834	79 – 92	551.9409	1652.8009	1652.8014	-0.30	0	83	5.5e-09	1	U	R.TFSNISNVMEKPMR.V
29331	79 – 92	557.2724	1668.7955	1668.7963	-0.51	0	60	1.1e-06	1	U	R.TFSNISNVMEKPMR.V + Oxidation (M)
29332	79 – 92	835.4070	1668.7993	1668.7963	1.80	0	95	3.3e-10	1	U	R.TFSNISNVMEKPMR.V + Oxidation (M)
40422	95 – 113	1108.5224	2215.0302	2215.0321	-0.83	0	67	2e-07	1	U	K.VDLYANSSYSDYVQTVYTK.Q
40423	95 – 113	1108.5322	2215.0498	2215.0321	8.02	0	61	7.3e-07	1	U	K.VDLYANSSYSDYVQTVYTK.Q

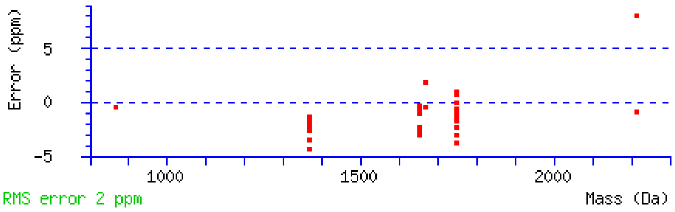

Mascot: http://www.matrixscience.com/

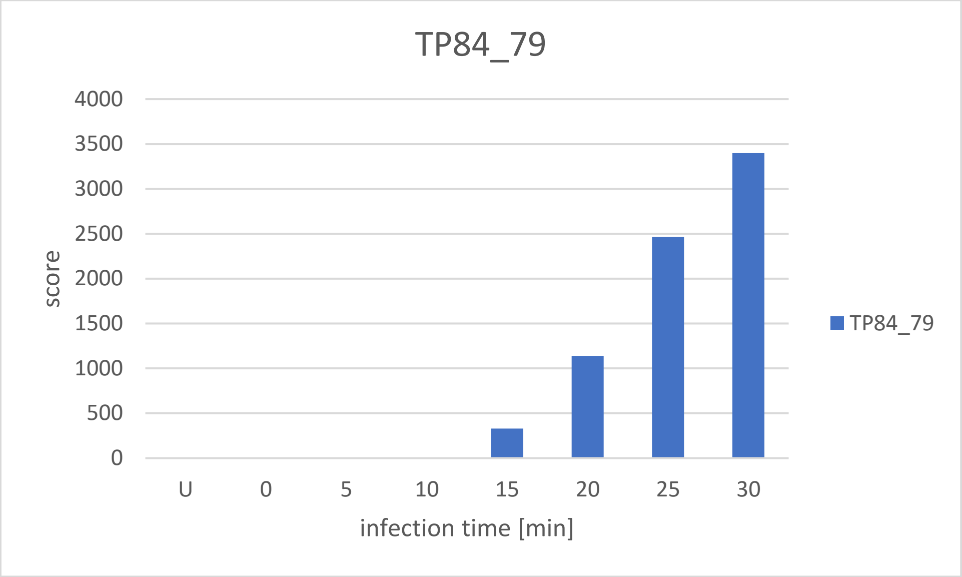

MASCOT Search Results

Protein View: TP84_80

Database: TP84
Score: 166
Nominal mass (M_r): 13948
Calculated pI: 6.82

Sequence similarity is available as an NCBI BLAST search of 53. against nr.

Search parameters

MS data file: O:\FA\02-luty2018\80202244zeb_czas20.raw
Enzyme: Trypsin: cuts C-term side of KR unless next residue is P.
Fixed modifications: Carbamidomethyl (C)
Variable modifications: Oxidation (M)

Protein sequence coverage: 9%

Matched peptides shown in ***bold red***.

```
  1 MGDMATTSKA IELNDNFTYG GTGYLNTSED YMNVYATWET PYNTYVVYASM
 51 TLQRYEDGVW KNIETKGAYA YYTHQERKHA NVQFTNIAKK GTPMRVKLNL
101 HDGNNPNSPS MQTAYSYSWT R
```

Unformatted sequence string: 121 residues (for pasting into other applications).

Sort peptides by ◉ Residue Number ○ Increasing Mass ○ Decreasing Mass

Show predicted peptides also

Query	Start - End	Observed	Mr(expt)	Mr(calc)	ppm	M	Score	Expect	Rank	U	Peptide
16826	79 - 89	621.8364	1241.6583	1241.6517	5.32	0	92	6.2e-10	1	U	K.HANVQFTNIAK.K
16827	79 - 89	621.8366	1241.6586	1241.6517	5.60	0	86	2.3e-09	1	U	K.HANVQFTNIAK.K

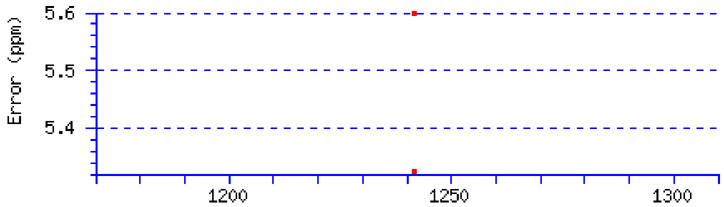

RMS error 5 ppm

Mascot: http://www.matrixscience.com/

MASCOT Search Results

Protein View: TP84_80

Database: TP84
Score: 511
Nominal mass (M_r): 13948
Calculated pI: 6.82

Sequence similarity is available as an NCBI BLAST search of 53. against nr.

Search parameters

MS data file: O:\FA\02-luty2018\80202245zeb_czas25.raw
Enzyme: Trypsin: cuts C-term side of KR unless next residue is P.
Fixed modifications: Carbamidomethyl (C)
Variable modifications: Oxidation (M)

Protein sequence coverage: 38%

Matched peptides shown in ***bold red***.

```
  1 MGDMATTSKA IELNDNFTYG GTGYLNTSED YMNVYATWET PYNTYVYASM
 51 TLQRYEDGVW KNIETKGAYA YYTHQERKHA NVQFTNIAKK GTPMRVKLNL
101 HDGNNPNSPS MQTAYSYSWT R
```

Unformatted sequence string: 121 residues (for pasting into other applications).

Sort peptides by ◉ Residue Number ○ Increasing Mass ○ Decreasing Mass

Show predicted peptides also

Query	Start - End	Observed	Mr(expt)	Mr(calc)	ppm	M	Score	Expect	Rank	U	Peptide
13924	67 - 77	453.5421	1357.6043	1357.6051	-0.56	0	55	3.2e-06	1	U	K.GAYAYYTHQER.K
13928	67 - 77	453.5424	1357.6053	1357.6051	0.14	0	57	2.1e-06	1	U	K.GAYAYYTHQER.K
13929	67 - 77	453.5426	1357.6059	1357.6051	0.59	0	59	1.3e-06	1	U	K.GAYAYYTHQER.K
13930	67 - 77	679.8106	1357.6067	1357.6051	1.18	0	97	2.1e-10	1	U	K.GAYAYYTHQER.K
14377	78 - 89	457.5896	1369.7470	1369.7466	0.28	1	59	1.2e-06	1	U	R.KHANVQFTNIAK.K
14378	78 - 89	457.5896	1369.7471	1369.7466	0.33	1	52	5.6e-06	1	U	R.KHANVQFTNIAK.K
14379	78 - 89	457.5897	1369.7473	1369.7466	0.48	1	55	3.3e-06	1	U	R.KHANVQFTNIAK.K
10813	79 - 89	621.8331	1241.6516	1241.6517	-0.023	0	98	1.5e-10	1	U	K.HANVQFTNIAK.K
35225	98 - 121	918.4150	2752.2233	2752.2299	-2.42	0	83	4.9e-09	1	U	K.LNLHDGNNPNSPSMQTAYSYSWTR.-

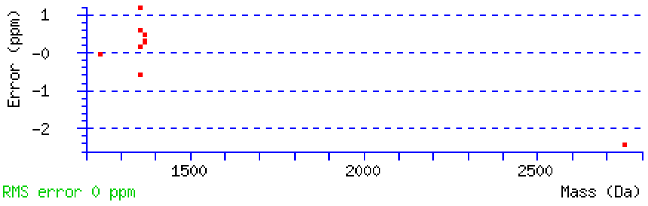

Mascot: http://www.matrixscience.com/

MASCOT Search Results

Protein View: TP84_80

**Database:**	TP84
**Score:**	1229
**Nominal mass (M_r):**	13948
**Calculated pI:**	6.82

Sequence similarity is available as an NCBI BLAST search of 53. against nr.

Search parameters

**MS data file:**	O:\FA\02-luty2018\80202246zeb_czas30.raw
**Enzyme:**	Trypsin: cuts C-term side of KR unless next residue is P.
**Fixed modifications:**	Carbamidomethyl (C)
**Variable modifications:**	Oxidation (M)

Protein sequence coverage: 19%

Matched peptides shown in ***bold red***.

```
  1 MGDMATTSKA IELNDNFTYG GTGYLNTSED YMNVYATWET PYNTYVYASM
 51 TLQRYEDGVW KNIETKGAYA YYTHQERKHA NVQFTNIAKK GTPMRVKLNL
101 HDGNNPNSPS MQTAYSYSWT R
```

Unformatted sequence string: 121 residues (for pasting into other applications).

Sort peptides by ◉ Residue Number ○ Increasing Mass ○ Decreasing Mass

Show predicted peptides also

Query	Start – End	Observed	Mr(expt)	Mr(calc)	ppm	M	Score	Expect	Rank	U	Peptide
19644	67 – 77	679.8091	1357.6036	1357.6051	-1.07	0	103	5.2e-11	1	U	K.GAYAYYTHQER.K
19645	67 – 77	679.8093	1357.6041	1357.6051	-0.75	0	97	1.9e-10	1	U	K.GAYAYYTHQER.K
19646	67 – 77	679.8094	1357.6043	1357.6051	-0.56	0	103	5.2e-11	1	U	K.GAYAYYTHQER.K
19647	67 – 77	453.5421	1357.6044	1357.6051	-0.54	0	69	1.3e-07	1	U	K.GAYAYYTHQER.K
19648	67 – 77	679.8095	1357.6045	1357.6051	-0.43	0	103	5.3e-11	1	U	K.GAYAYYTHQER.K
19649	67 – 77	453.5421	1357.6045	1357.6051	-0.41	0	90	9.5e-10	1	U	K.GAYAYYTHQER.K
19650	67 – 77	453.5422	1357.6049	1357.6051	-0.14	0	90	9.4e-10	1	U	K.GAYAYYTHQER.K
19651	67 – 77	453.5423	1357.6051	1357.6051	0.033	0	90	9.4e-10	1	U	K.GAYAYYTHQER.K
19652	67 – 77	679.8099	1357.6052	1357.6051	0.060	0	97	1.8e-10	1	U	K.GAYAYYTHQER.K
19653	67 – 77	453.5425	1357.6056	1357.6051	0.39	0	90	9.5e-10	1	U	K.GAYAYYTHQER.K
20224	78 – 89	685.8818	1369.7490	1369.7466	1.73	1	88	1.7e-09	1	U	R.KHANVQFTNIAK.K
15148	79 – 89	621.8331	1241.6517	1241.6517	0.041	0	101	8.8e-11	1	U	K.HANVQFTNIAK.K
15149	79 – 89	621.8332	1241.6518	1241.6517	0.15	0	98	1.6e-10	1	U	K.HANVQFTNIAK.K
15150	79 – 89	621.8332	1241.6519	1241.6517	0.19	0	96	2.8e-10	1	U	K.HANVQFTNIAK.K
15152	79 – 89	621.8338	1241.6530	1241.6517	1.06	0	96	2.3e-10	1	U	K.HANVQFTNIAK.K

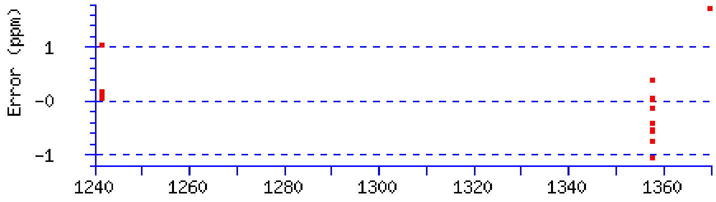

RMS error 0 ppm

Mascot: http://www.matrixscience.com/

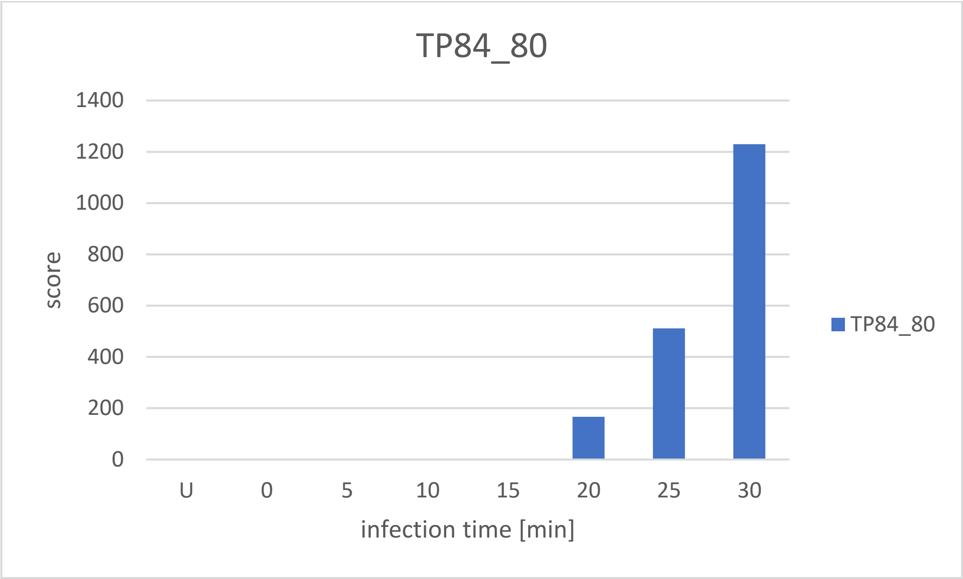

TP84_80
1400
1200
1000
800
600
400
200
0
score
U
0
5
10
15
20
25
30
infection time [min]
TP84_80
