# Supplementary material for: Sequence, genome organization, annotation and proteomics of the thermophilic, 47.7-kb Geobacillus stearothermophilus bacteriophage TP-84 and its classification in the new Tp84virus genus
Source: PLoS One. 2018 Apr 6;13(4):e0195449. doi: 10.1371/journal.pone.0195449 (PMC5889276; doi:10.1371/journal.pone.0195449)
Supplement: S1 Table — (DOC) [file pone.0195449.s007.doc]

Putative regulatory elements in TP-84 DNA

1. Promoters

| coordinates | upstream | -35 |  | -10 | downstream |
| --- | --- | --- | --- | --- | --- |
| 5255..25284 | atattttttaaaaatcatcg | ttgaca | ttgtccacgatcgcatga | tagtat | aaaggcaaca |
| 7242..7271 | gacaactgaatggaaaaata | ttcact | ttttagccgaatgtttgg | tataat | aacgataaag |
| 25056..25083 | aattggtatcattaaaagtc | atgaca | aaactgacgatcagtga | tattat | atgaagtgcc |
| 36613..36642 | atcctgggaacaaaagaggg | gtgaca | aatctgacatccctaaat | tataaa | aaataccgta |
| 36865..36893 | acaattttttcagttttttg | tttaca | gtttataaacaataagc | tataat | gaaagtacca |
|  |  |  |  |  |  |
| Consensus |  | TTGACA | N15-18 | TATAAT |  |

N.B. the upstream region of several of these putative promoters in unusually AT-rich

1. Rho-independent terminators

| coordinates | up-stem | loop | down-stem | tail | Initial ΔG (kcal/mol) * |
| --- | --- | --- | --- | --- | --- |
| 9739..9772 | gggatagac | acatgatcta | gtctatccc | tttttt | -14.3 |
| 12160..12192 | ggatagggaac | ataat | gatccctatcc | tttttt | -15.5 |
| 25180..25214 | gatccgccatcc | aaac | ggatagcggatc | ttttttt | -17.8 |
| 25893..25919 | ggcgaat | aaaaaa | attcgcc | atttttt | -10.3 |
| 29593..29629 | ggcactga | aaaaaa | tcagtgcc | ttttttattttt | -13.70 |
| 33363..33397 | ggtcgttcttg | gatcaga | caggaacggcc | ttttttgtt | -17.7 |
| 33616..33643 | gccaggg | atgat | ccctggc | ttttttatt | -15.2 |
| 46251..46275 | ggccagg | ggaaa | cctggtc | tttttt | -12.8 |
| 47132..47171 ** | ggacatcctgga | cattt | tccctggatgtcc | atttttt | -17.5 |
| 47611..47645 | catcctggac | aaaata | gtctagggtg | tcttttttt | -14.8 |

* calculated using The mfold Web Server (<http://unafold.rna.albany.edu/?q=mfold/RNA-Folding-Form>)

** the stem contains a bubble
